# Morphometric and topographic data of small and medium size landforms in the Northern Circumpolar Region of Mars

**DOI:** 10.1016/j.dib.2022.108417

**Published:** 2022-06-24

**Authors:** Marina Sánchez-Bayton, Miguel Herraiz, Patrick Martin, Beatriz Sánchez-Cano, Erwan Tréguier, Akos Kereszturi

**Affiliations:** a Department of Physics of the Earth and Astrophysics, Universidad Complutense de Madrid (UCM), Madrid, Spain; b Instituto de Matemática Interdisciplinar (IMI), Madrid, Spain; c ESAC (European Space Astronomy Centre), ESA (European Space Agency), Villanueva de la Cañada, Spain; d School of Physics and Astronomy, University of Leicester, Leicester, United Kingdom; e Research Centre for Astronomy and Earth Sciences, Konkoly Thege Miklos Astronomical Institute, Hungary; f European Astrobiology Institute, Hungary

**Keywords:** Mars Northern polar region, Volcanic landforms, Impact craters, Morphometric and topographic data, Scandia Cavi, Olympia Undae, Mars Express, Mars Reconnaissance Orbiter

## Abstract

A substantial dataset containing topographic landforms at Olympia Undae and Scandia Cavi in the Northern circumpolar region of Mars was created by Sanchez-Bayton et al. (2022) [1]. This dataset contains the essential morphometric parameters of 200 small and medium-size landforms. In particular, it includes cratered, non-cratered, and complex irregular structures. Experimental Data Records (EDR) were obtained from the Mars Express, Mars Reconnaissance Orbiter, and Mars Global Surveyor missions, and the analysed dataset was produced thanks to the Java Mission-planning and Analysis for Remote Sensing (JMARS) software. This dataset constitutes a significant improvement in the characterisation of the small and medium-size topographic structures in the Northern circumpolar region of Mars and it contributes towards better understanding of the Northern circumpolar area. This dataset is of great value for modellers and other studies of the Martian surface processes, related to volcanic structures, aeolian processes driving to erosion or deposition, sublimation and subglacial processes, and several impact events.

## Specifications Table

**Table:** 

Subject	Space and Planetary Science
Specific subject area	Geophysics of the Northern circumpolar region of Mars
Type of data	Files Images
How the data were acquired	Data presented here are geophysical morphometric products from orbital images and topographic profiles of the 200 small and medium-size geological landforms analysed in [1] at the Northern circumpolar region of Mars. In particular, data come from latitudes 72°−80° N and 150°−230° E, which includes a small part of Olympia Undae (68.500 km2) and a large area of Scandia Cavi (302.810 km2). Reduced Data Records (RDR) from satellite data include 221 nadir images of level 3 (photometrically normalised) from the High Resolution Stereo Camera (HRSC) [2,3] onboard Mars Express, 51 from the High Resolution Imaging Science Experiment (HiRISE) [4] and 2015 from the Context Camera (CTX) [5] onboard Mars Reconnaissance Orbiter. Analysed data include elevation and slope topographic profiles, topographic maps, and 2D/3D views of the 200 landforms from digital elevation and terrain models (DEM/DTM) obtained from data from the Mars Orbiter Laser Altimeter (MOLA) [6] onboard Mars Global Surveyor. These profiles are acquired with the JMARS (Java Mission-planning and Analysis for Remote Sensing) Open Access software [7], which is a geospatial information system that provides data-analysis tools for Mars missions.
Data format	Reduced Data Records (RDR) Analysed
Description of data collection	RDR data acquired and analysed from the surface of Mars using the space missions Mars Express, Mars Reconnaissance Orbiter and Mars Global Surveyor. The RDR datasets are obtained from the NASA Planetary Data System and the ESA Planetary Science Archive. It consists on images taken by these three spacecraft at Mars, as well as elevation profiles of the topography of Mars taken from orbit with the instrument MOLA on Mars Global Surveyor. The analysed dataset contains topographic information together with processed orbital images using the software JMARS [7].
Data source location	Location: Mars. Scandia Cavi and Olympia Undae regions of the Martian Northern polar area.RDR data source: - HRSC images: http://archives.esac.esa.int/psa/ftp/MARS-EXPRESS/HRSC/ - MOLA dataset: https://pds-geosciences.wustl.edu/mgs/mgs-m-mola-1-aedr-l0-v1/mgsl_1xxx/data/ - HiRISE and CTX images: https://pds-imaging.jpl.nasa.gov/volumes/mro.html Analysed dataset: - https://leicester.figshare.com/articles/dataset/Small_and_medium_size_landforms_in_Scandia_Cavi_and_Olympia_Undae_/16822300 - Supplementary information file
Data accessibility	Experimental Data Records are publically available at the NASA Planetary Data System and the ESA Planetary Science Archive: - HRSC images: http://archives.esac.esa.int/psa/ftp/MARS-EXPRESS/HRSC/ - MOLA dataset: https://pds-geosciences.wustl.edu/mgs/mgs-m-mola-1-aedr-l0-v1/mgsl_1xxx/data/ - HiRISE and CTX images: https://pds-imaging.jpl.nasa.gov/volumes/mro.html
	Analysed data (RDR) are available with the article and data repository.Data identification number: 10.25392/leicester.data.16822300.v1Direct URL to data [8]: https://leicester.figshare.com/articles/dataset/Small_and_medium_size_landforms_in_Scandia_Cavi_and_Olympia_Undae_/16822300. There is also a Supplementary information file to this article
Related research article	M. Sánchez-Bayton, M. Herraiz, P. Martin, B. Sánchez-Cano, E. Tréguier, A. Kereszturi, Morphological analyses of small and medium size landforms in Scandia Cavi and Olympia Undae, Northern Circumpolar Region of Mars, Planetary and Space Science. 2021, 210, 105389, ISSN 0032–0633, https://doi.org/10.1016/j.pss.2021.105389.

## Value of the Data

• This dataset contributes towards a better understanding and characterization of the Northern circumpolar area of Mars, particularly Scandia Cavi and Olympia Undae terrains.
• This dataset characterises small and medium-size topographic structures most of them not studied before in the Northern polar region of Mars, including cratered, non-cratered, and complex irregular structures.
• Several of the structures involve subsurface processes such as volcanic activity, aeolian processes driving to erosion or deposition, sublimation and subglacial processes, and several impact events.

## 1 Data Description

All the files and images presented here contain the details and morphological data of the 200 small-medium size structures of [1]. In particular, this dataset contains files, and 3D topographic views in this document as described hereafter:

### 1.1 Data files

#### 1.1.1 File “NorthPole_Landforms.jmars”

**Fig. 1:**
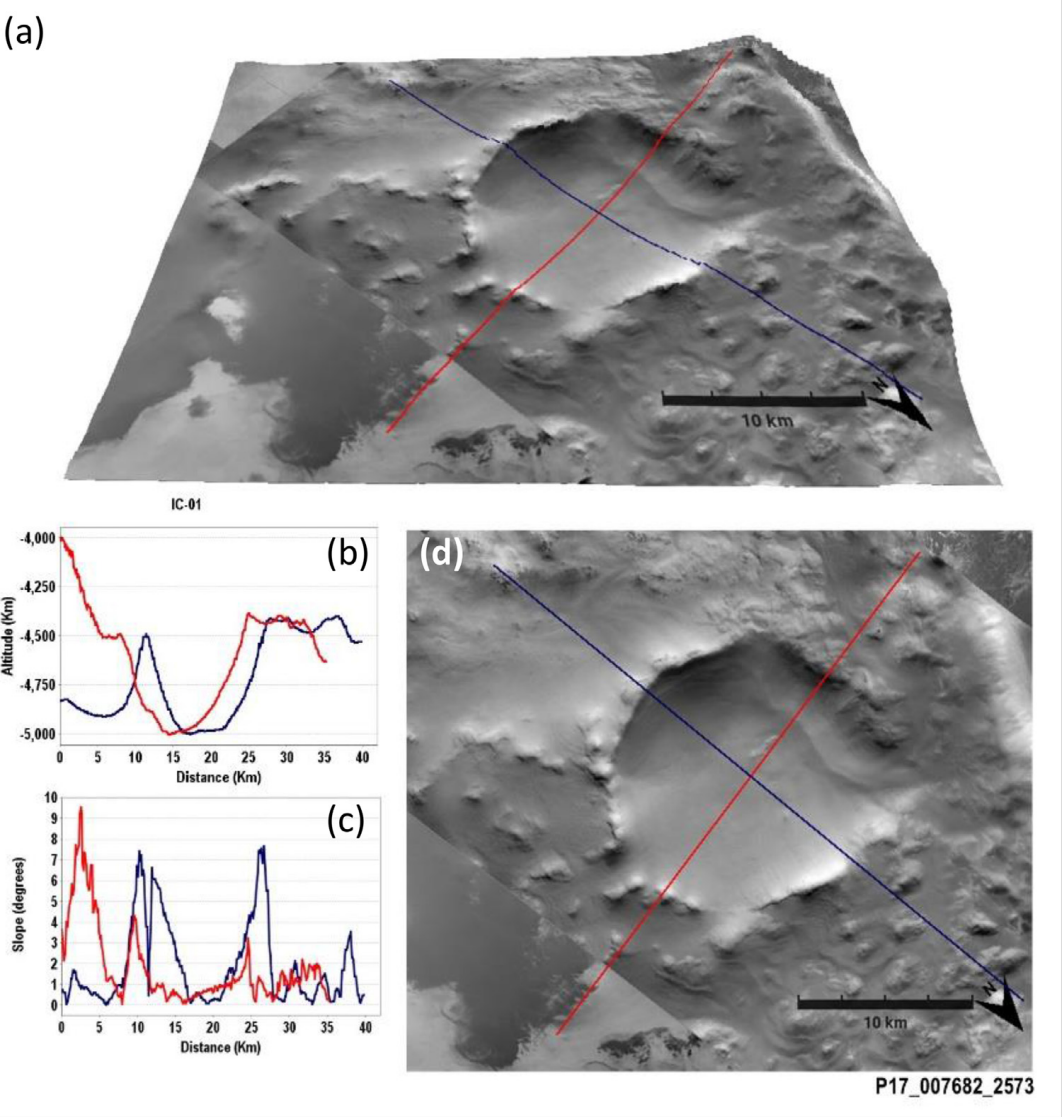
Structure IC-01 as example of analysis performed and stored in the supplementary material. (a) 3D view from JMARS where the altimetry from MOLA and CTX images have been over-plotted. Two profiles are drawn that correspond to the data archived in [8]. (b-c) Elevation and slope of the profiles in panel (a), respectively. (d) Same as in (a) but in 2D.

File to be opened with the Open Access Software JMARS [7]. It contains a geographic map of the Northern Polar region of Mars with the 200 analysed structures. This map was used to produce Figs. 1 and 5 in [1]. The contour of each of the structures is marked with a black solid line, and their areas with different colours. In particular, green is used for Cratered Cones (CC), orange for Impact Craters (IC), yellow for Undifferentiated Craters (UC), light pink for Simple Domes (SD), purple for Peaked Domes (PD), and light blue for Irregular Structures (IS). Each structure has the same identification number than in [1]. The latitude and longitude of each of the structures is available. Moreover, each user is allowed to add additional layers to this map for further studies. The number of available layers in JMARS is large, including magnetic field data, surface and surface radar data or topographic data amongst many other possibilities.

#### 1.1.2 File “Landforms.csv”

This is a general file that contains the length of several profiles that cross a structure as well as the longitude, latitude, bent slopes and altimetry of those profiles for every structure. This file has been created with the intention of facilitating access to the altimetry and slope data of all structures in a single file.

#### 1.1.3 Files in folders named “CC, IC, IS, PD, SD and UC”

These folders contain 1208 files that contain the individual profiles performed for each structure. In particular, those named "ZZ-XX-Y", where ZZ is the name of the folder (or type of structure), XX is the number that identifies the given structure, and Y is the number that identifies the altimetry and bent slope profiles over each structure. It contains the length, longitude, latitude, bent slopes and elevation 0above 59° N of each profile per structure. Those named as "ZZ-XX-K", where ZZ is the name of the folder (or type of structure), XX is the number that identifies the structure, and K is a letter that identifies different altimetry profiles over each structure. It contains the length, longitude, latitude, elevation and shaded relief. MOLA High Resolution Shaded Relief Maps are very high resolution topographic shade maps of Mars that are produced from the MOLA Science Team. Polar views are in a polar stereographic projection and have a resolution of 300 or 600 m. The grayscale maps are gradients from 30° clockwise from the top of the rectangle, then normalized by an arctangent function, and finally, merged with topographic colour contours to produce images using the Generic Mapping Tools (GMT). We note that Shape files (.shp) are not included but can be downloaded from the JMARS map file (Section 1.1)”.

### 1.2 3D topographic views

Fig. 1 contains an example of 3D view analysis performed to the data for a single structure. The same 3D view for the 200 structures presented in [1] can be found in the Supplementary material. For each of the structures, panel (a) shows a 3D view from JMARS where the altimetry from MOLA and either HRSC, HiRISE or CTX images have been over-plotted. Two profiles are drawn that correspond to the data archived in [8]. The 3D views in JMARS are created using the standard deviation mode of the dataset to draw the vertical scaling. They also have a 10 vertical exaggeration and 128 pixels per degree (PPD). Panels (b) and (c) show the MOLA elevation and HRSC MOLA blended DEM slope (200 m) [9] of the profiles in panel (a), respectively. Panel (d) shows the same as panel (a) but in 2D.

## 2 Experimental Design, Materials and Methods

Data presented here are geophysical morphometric products derived from orbital images and topographic profiles of the 200 small and medium-size geological landforms analysed in [1] in the Northern circumpolar region of Mars. 23 structures are situated in Olympia Undae and 177 in Scandia Cavi.

The first step consisted of selecting the structures for study and corroborate that enough data was available using the DEM/DTM data in JMARS. This included topographic observations and high quality orbital images. Then, structures with and without craters were separated and a grid of MOLA topographic elevation profiles were retrieved from each structure using JMARS as a blend of the DEM/DTM data derived from MOLA and orbital images data. Using all these retrieval profiles in a gridding and surface mapping software as well as in JMARS and MatLab, 2D and 3D views of each landform were created, which provided the main geomorphological characteristics of the structures, such as the shortest and longest crater diameters, crater depths, shortest and longest basal diameters and heights from the base of the structures. From the analysis of all these parameters, the 6 different categories of structures were created, as described and discussed in [1].

## Ethics Statements

Ethics statements are not required for the presented data. This work did not consider the use of human, animal subjects or social media sources.

## CRediT Author Statement

**Marina Sánchez-Bayton:** Conceptualization, Methodology, Formal analysis, Software; **Miguel Herraiz:** Conceptualization, Supervision, Validation, Writing – review & editing; **Patrick Martin:** Supervision, Validation, Writing – review & editing; **Beatriz Sánchez-Cano:** Supervision, Validation, Data Curation, Writing – review & editing; **Erwan Tréguier:** Validation, Writing – review & editing; **Akos Kereszturi:** Validation, Writing – review & editing.

## Declaration of Competing Interest

The authors declare that they have no known competing financial interests or personal relationships that could have appeared to influence the work reported in this paper.

## Data Availability

Small and medium size landforms in Scandia Cavi and Olympia Undae (Original data) (Figshare).

## Supplementary materials

Image, application 1

## References

[1] Sánchez-Bayton M., Herraiz M., Martin P., Sánchez-Cano B., Tréguier E., Kereszturi A. (2022). Morphological analyses of small and medium size landforms in Scandia Cavi and Olympia Undae, Northern Circumpolar Region of Mars. Planet. Space Sci..

[2] Neukum G., Jaumann R., Wilson A. (2004). Mars Express: The scientific Payload.

[3] Neukum J.G., Jaumann R., Hoffmann H., Hauber E., Head J.W., Basilevsky A.T., Ivanov B.A., Werner S.C., Van Gasselt S., Murray J.B., McCord T. (2004). Recent and episodic volcanic and glacial activity on Mars revealed by the High Resolution Stereo Camera. Nature.

[4] McEwen A.S., Banks M.E., Baugh N., Becker K., Boyd A. (2010). The High Resolution Imaging Science Experiment (HiRISE) during MRO's Primary Science Phase (PSP). Icarus.

[5] Malin M., Bell J., Cantor B., Caplinger M., Calvin W., Clancy T., Edgett K., Edwards L., Haberle R., James P., Lee S., Ravine M., Thomas P., Wolff M. (2007). Contex Camera Investigation on board the Mars Reconnaissance Orbiter. J. Geophys. Res..

[6] Smith D.E., Zuber M., Frey H., Garvin J., Head J., Muhleman D., Pettengill G., Phillips R., Solomon S., Zwally H., Banerdt W., Duxbury T., Golombek M., Lemoine F., Neumann G., Rowlands D., Aharonson O., Ford P., Ivanov A., Johnson C., McGovern P., Abshire J., Afzal R., Sun X. (2001). Mars Orbiter Laser Altimeter: experiment summary after the first year of global mapping of Mars. J. Geophys. Res..

[7] Christensen, P.R.; Engle, E.; Anwar, S.; Dickenshied, S.; Noss, D.; Gorelick, N.; Weiss-Malik, M.; JMARS – A Planetary GIS, http://adsabs.harvard.edu/abs/2009AGUFMIN22A..06C.

[8] Herraiz Miguel, Sanchez-Bayton Marina, Martin Patrick, Sanchez-Cano Beatriz, Tréguier Erwan, Kereszturi Akos (2021).

[9] Fergason, R.L., Hare, T.M., & Laura, J. (2018). HRSC and MOLA Blended Digital Elevation Model at 200 m v2. Astrogeology PDS Annex, U.S. Geological Survey. http://bit.ly/HRSC_MOLA_Blend_v0.

