# Supplementary material for: Morphometric and topographic data of small and medium size landforms in the Northern Circumpolar Region of Mars
Source: Data Brief. 2022 Jun 24;43:108417. doi: 10.1016/j.dib.2022.108417 (PMC9260444; doi:10.1016/j.dib.2022.108417)
Supplement: Supplementary file 1 [file mmc1.pdf]

Supplementary material for article in *Data in Brief*

Morphometric and topographic data of small and medium size landforms in the Northern Circumpolar Region of Mars.

Marina Sánchez-Bayton[1(*)], Miguel Herraiz[1,2], Patrick Martin[3], Beatriz Sánchez-Cano[4(*)], Erwan Tréguier[5], Akos Kereszturi[6,7]

[1] Department of Physics of the Earth and Astrophysics, Universidad Complutense de Madrid (UCM),
Madrid, Spain

[2] Instituto de Matemática Interdisciplinar (IMI), Madrid, Spain

[3] ESAC (European Space Astronomy Centre), ESA (European Space Agency), Villanueva de la Cañada,
Spain

[4] School of Physics and Astronomy, University of Leicester, Leicester, UK

[5] Formerly at ESAC (European Space Astronomy Centre), ESA (European Space Agency), Villanueva de
la Cañada, Spain

[6] Research Centre for Astronomy and Earth Sciences, Konkoly Thege Miklos Astronomical Institute,
Hungary

[7] European Astrobiology Institute, Hungary

Content of this file:

This file contains 3D view analysis performed to the 200 structures, following the description presented in the paper for Figure 1. For each of the structures, panel (a) shows a 3D view from JMARS where the altimetry from MOLA and either HRSC, HiRISE or CTX images have been over-plotted. Two profiles are drawn that correspond to the data archived in [8]. The 3D views in JMARS are created using the standard deviation mode of the dataset to draw the vertical scaling. They also have a 10 vertical exaggeration and 128 pixels per degree (PPD). Panels (b) and (c) show the MOLA altitude and HRSC MOLA blended DEM slope (200m) [9] of the profiles in panel (a), respectively. Panel (d) shows the same as panel (a) but in 2D.

CC-01

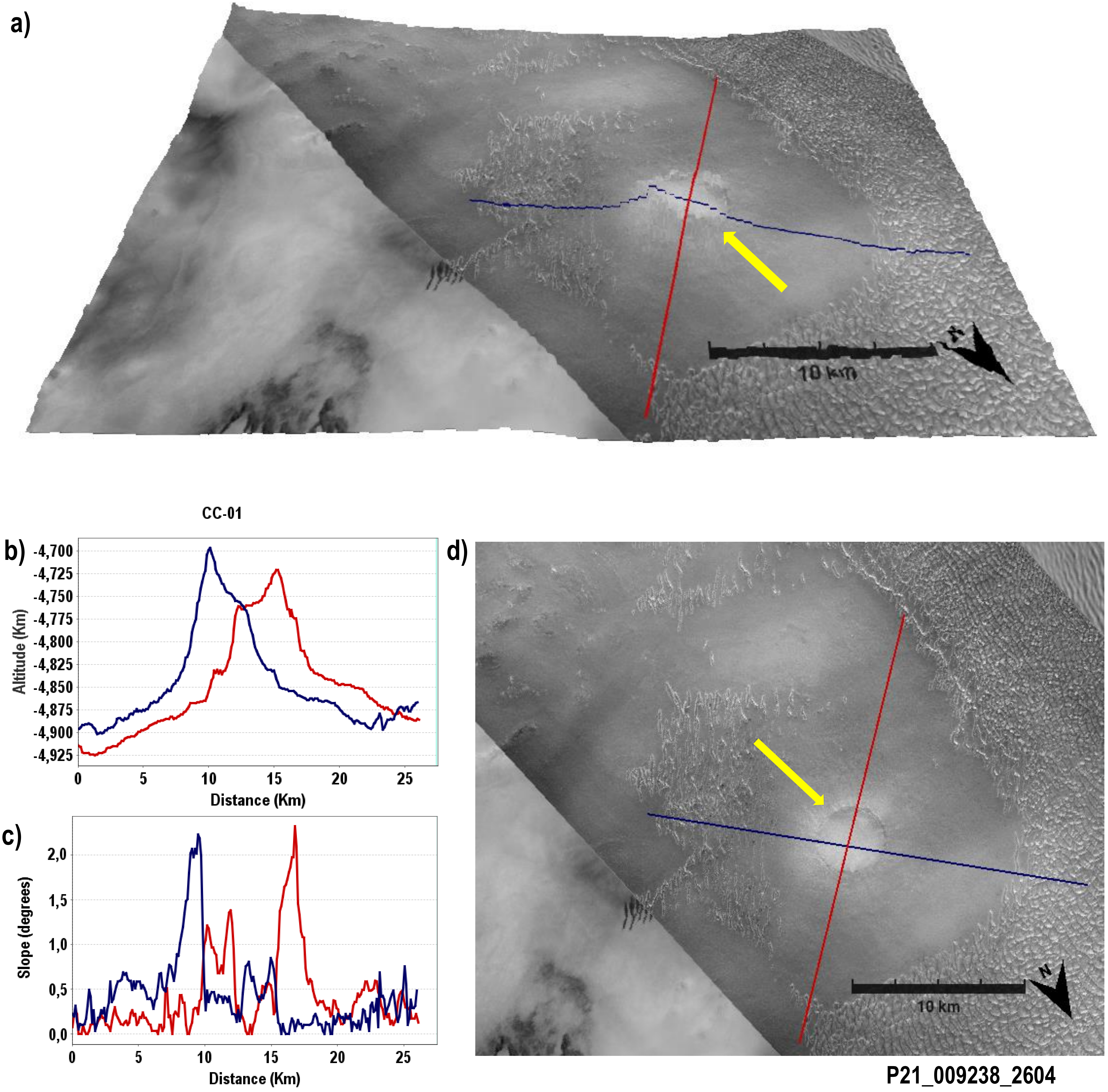

a)
10 km
CC-01
b)
Altitude (Km)
-4,700
-4,725
-4,750
-4,775
-4,800
-4,825
-4,850
-4,875
-4,900
-4,925
0
5
10
15
20
25
Distance (Km)
c)
Slope (degrees)
2,0
1,5
1,0
0,5
0,0
0
5
10
15
20
25
Distance (Km)
d)
10 km
N
P21_009238_2604

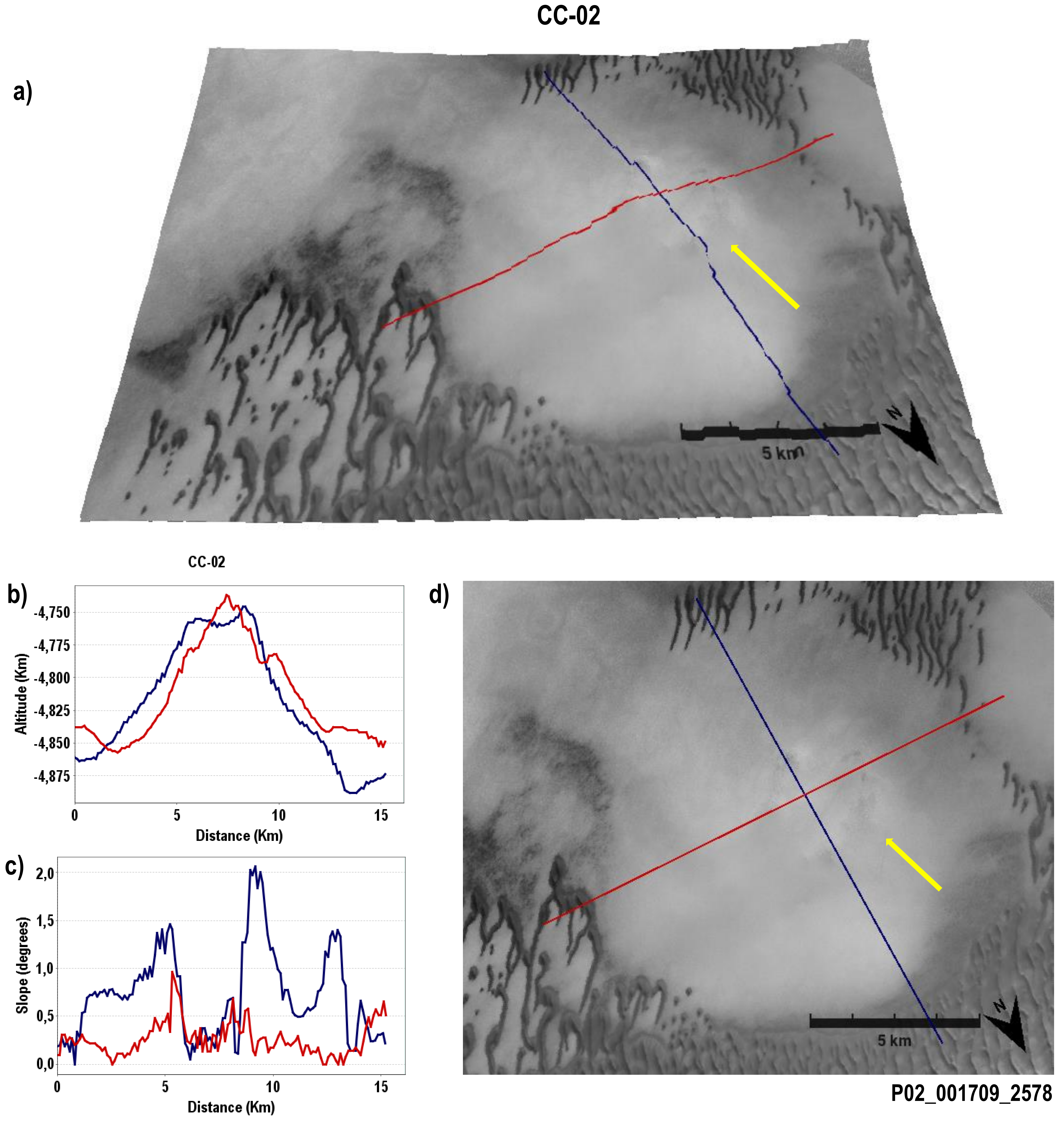
CC-02
a)
5 km
N
CC-02
b)
Altitude (Km)
-4,750
-4,775
-4,800
-4,825
-4,850
-4,875
0
5
10
15
Distance (Km)
c)
Slope (degrees)
2,0
1,5
1,0
0,5
0,0
0
5
10
15
Distance (Km)
d)
5 km
N
P02_001709_2578

CC-03

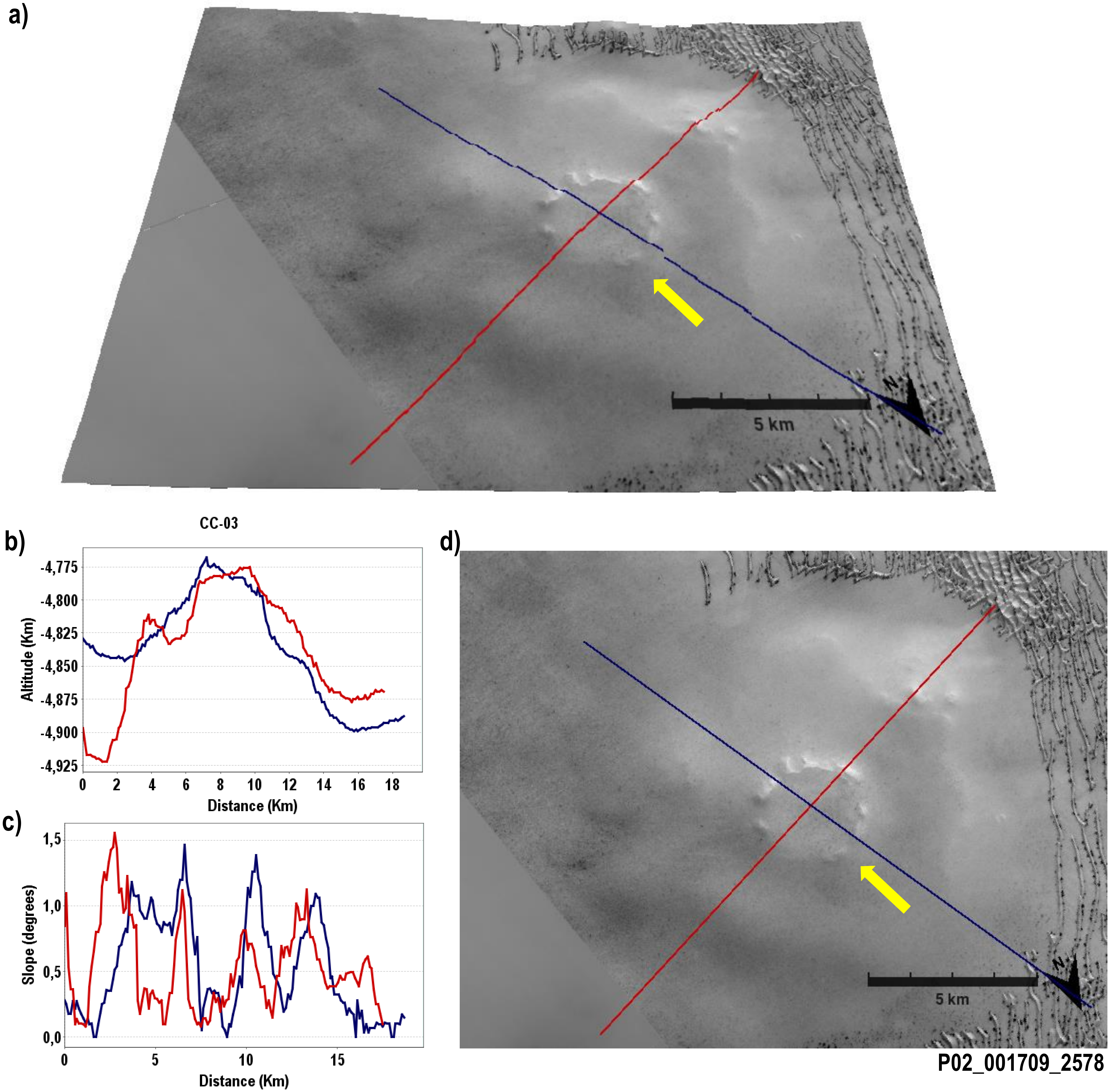

P02_001709_2578

CC-04

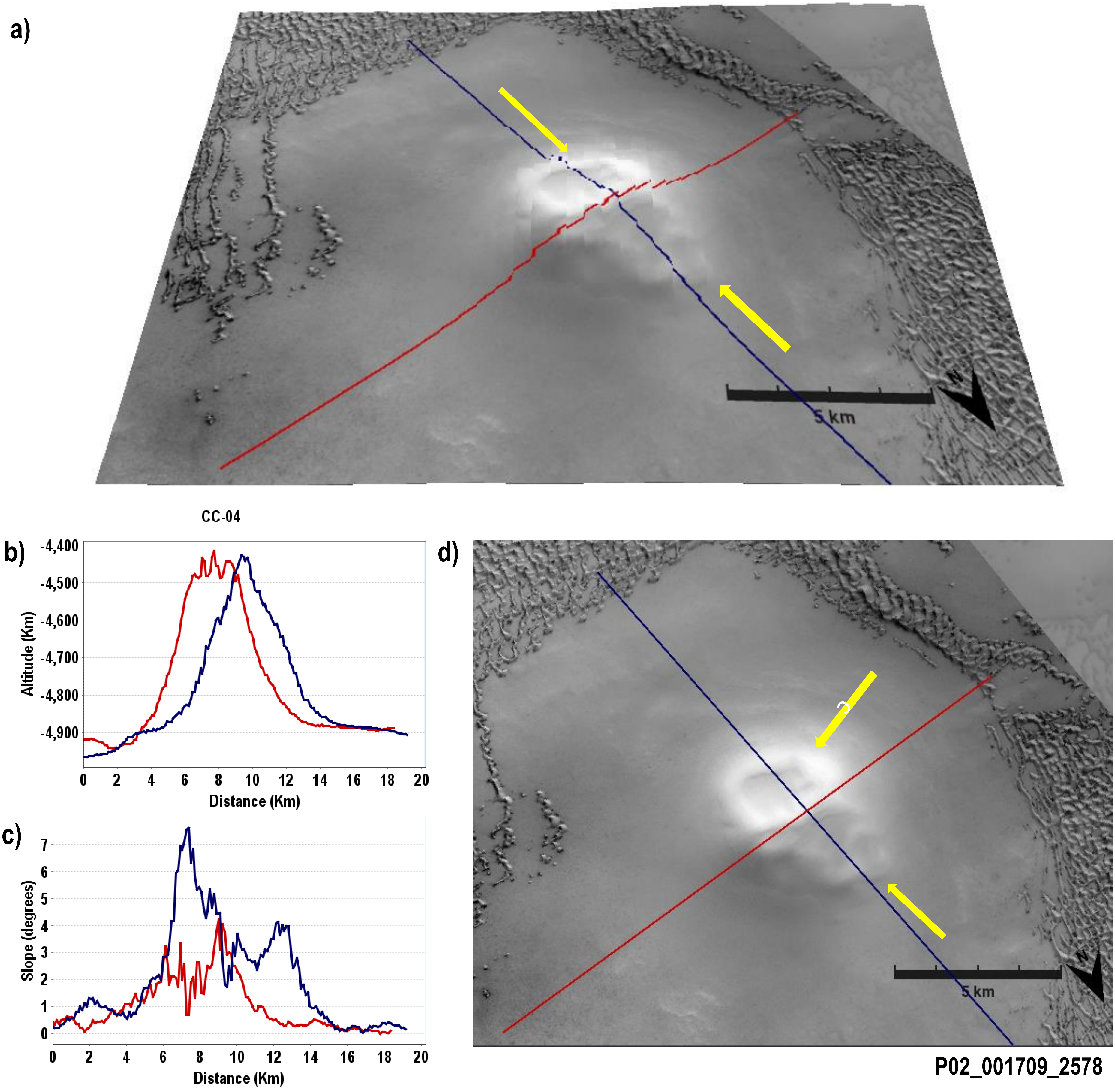

P02_001709_2578

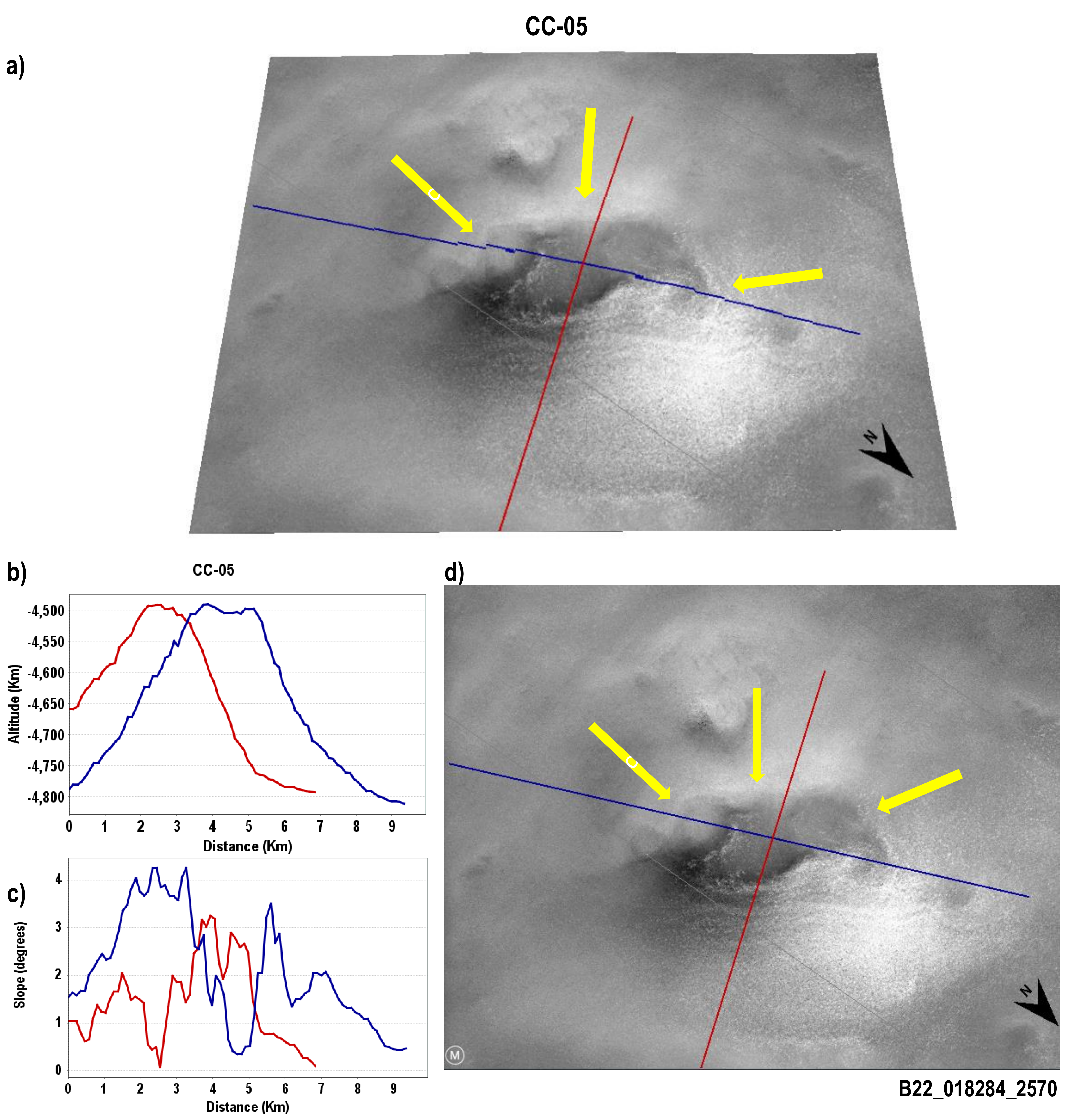
CC-05
a)
N
b)
CC-05
-4,500
-4,550
-4,600
-4,650
-4,700
-4,750
-4,800
Altitude (Km)
0 1 2 3 4 5 6 7 8 9
Distance (Km)
c)
4
3
2
1
0
Slope (degrees)
0 1 2 3 4 5 6 7 8 9
Distance (Km)
d)
N
B22_018284_2570

CC-06

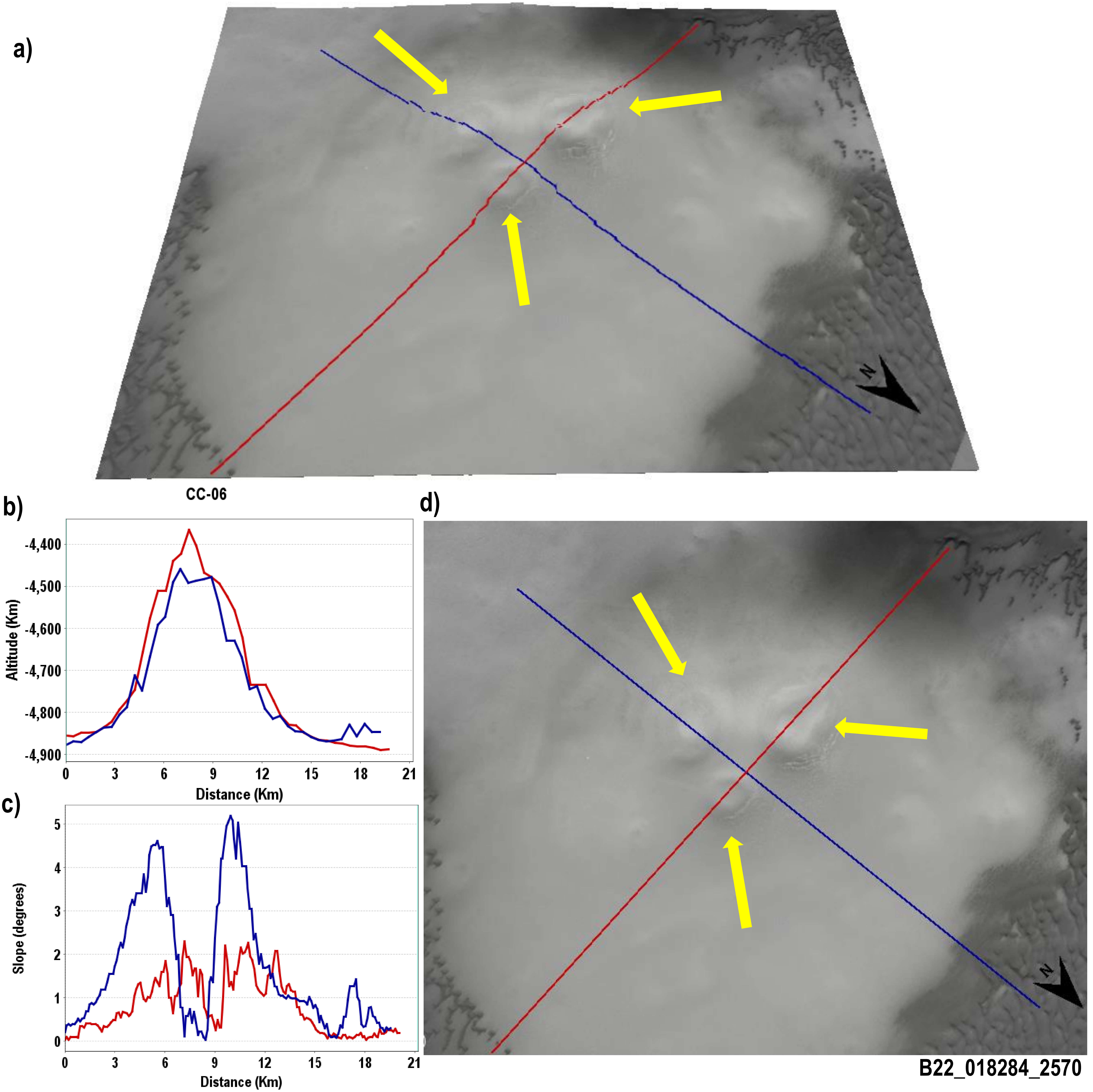

B22_018284_2570

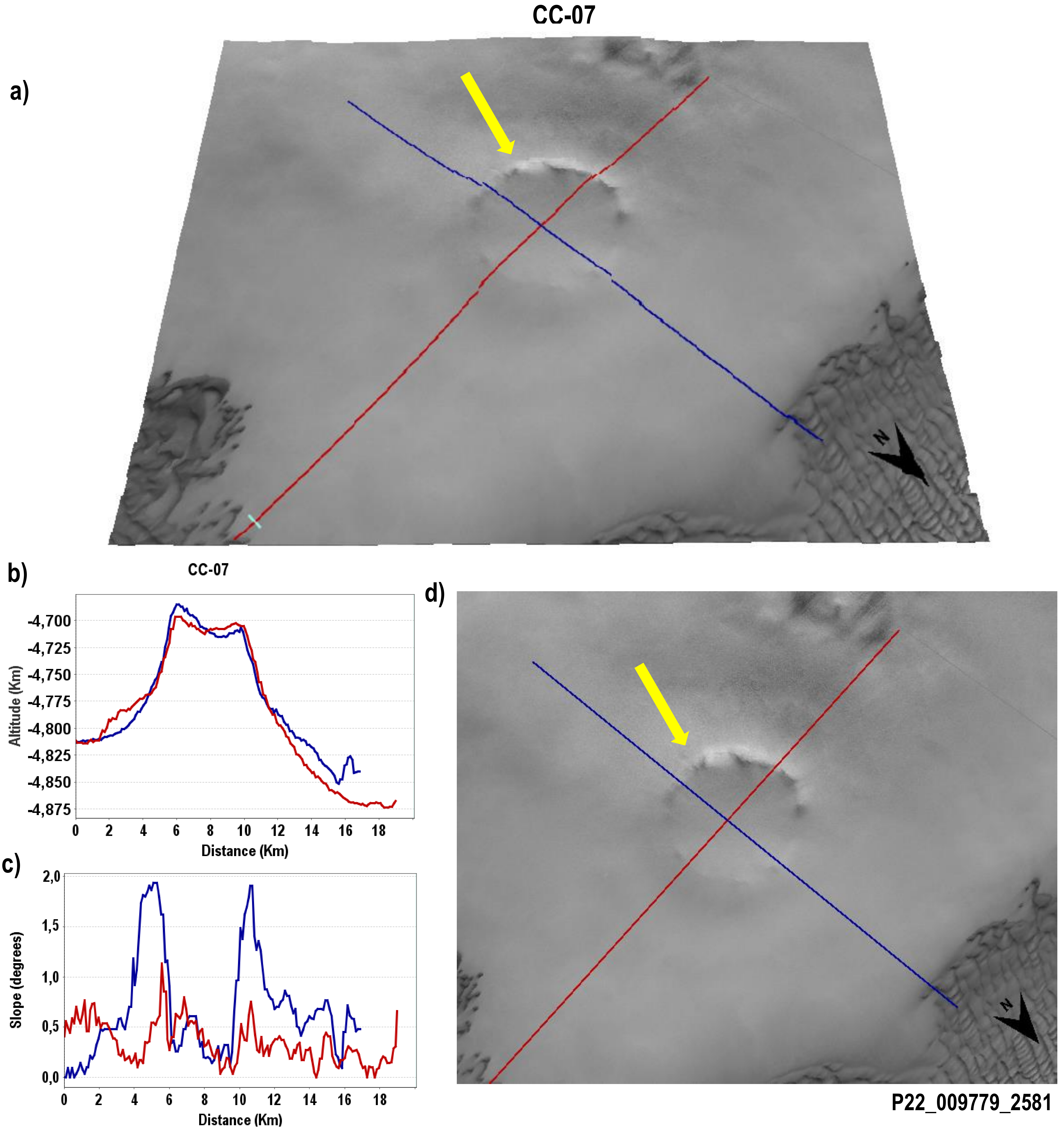
CC-07
a)
N
b)
CC-07
Altitude (Km)
-4,700
-4,725
-4,750
-4,775
-4,800
-4,825
-4,850
-4,875
0 2 4 6 8 10 12 14 16 18
Distance (Km)
c)
Slope (degrees)
2,0
1,5
1,0
0,5
0,0
0 2 4 6 8 10 12 14 16 18
Distance (Km)
d)
N
P22_009779_2581

CC-08

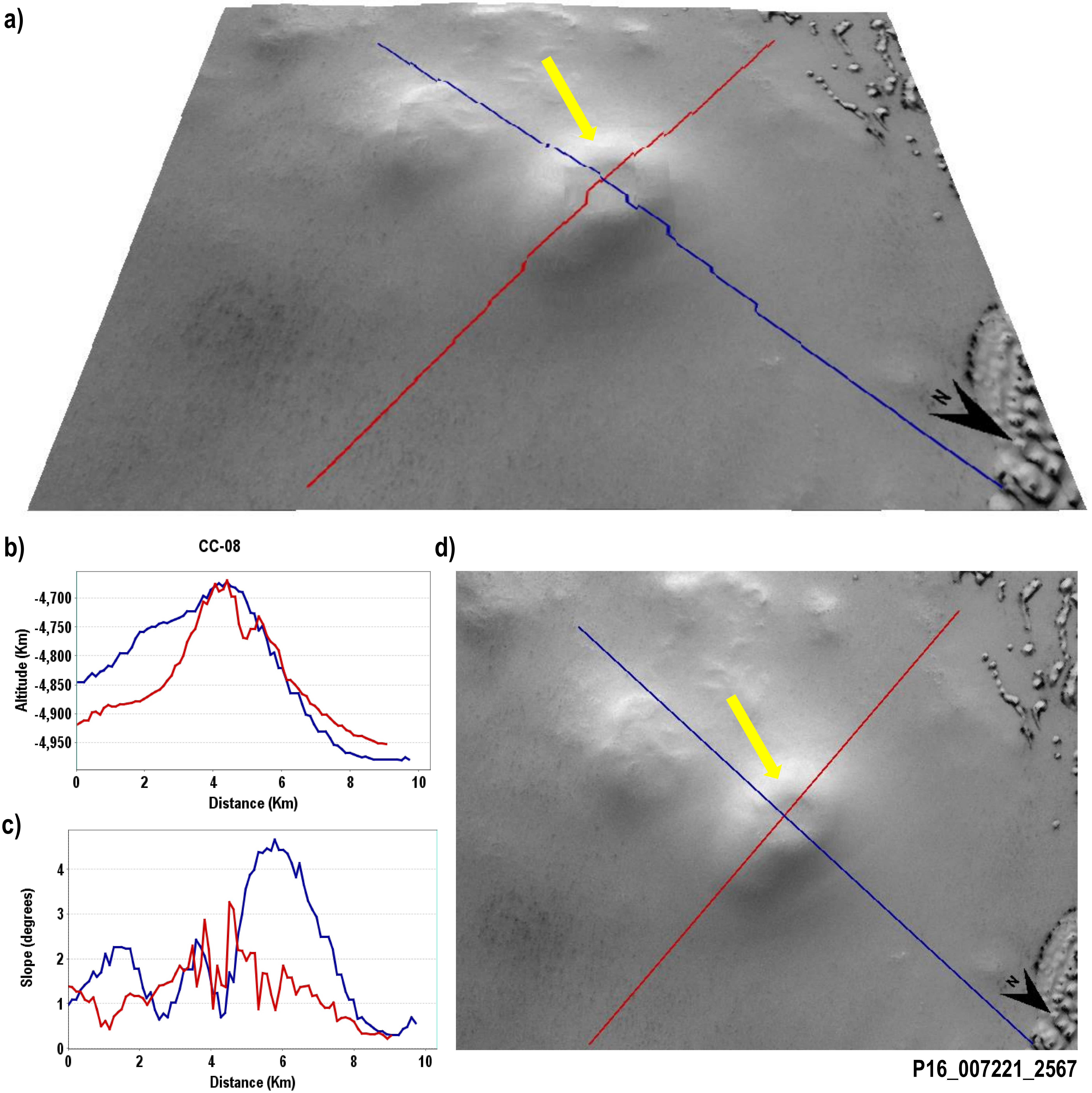
a)
N
b)
CC-08
Altitude (Km)
-4,700
-4,750
-4,800
-4,850
-4,900
-4,950
0
2
4
6
8
10
Distance (Km)
c)
Slope (degrees)
0
1
2
3
4
0
2
4
6
8
10
Distance (Km)
d)
N
P16_007221_2567

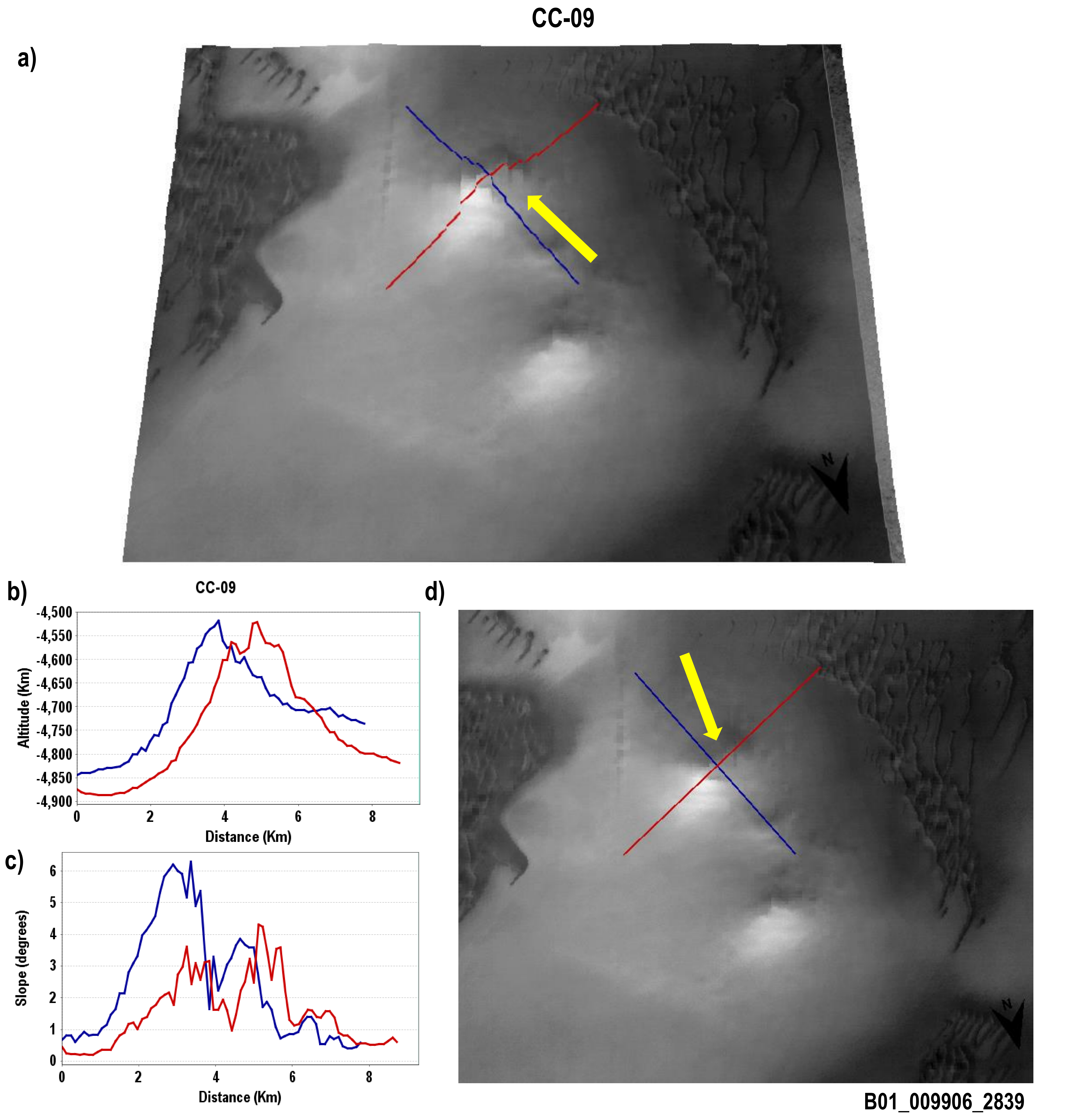
CC-09
a)
b)
CC-09
Altitude (Km)
-4,500
-4,550
-4,600
-4,650
-4,700
-4,750
-4,800
-4,850
-4,900
0
2
4
6
8
Distance (Km)
c)
Slope (degrees)
6
5
4
3
2
1
0
0
2
4
6
8
Distance (Km)
d)
N
B01_009906_2839

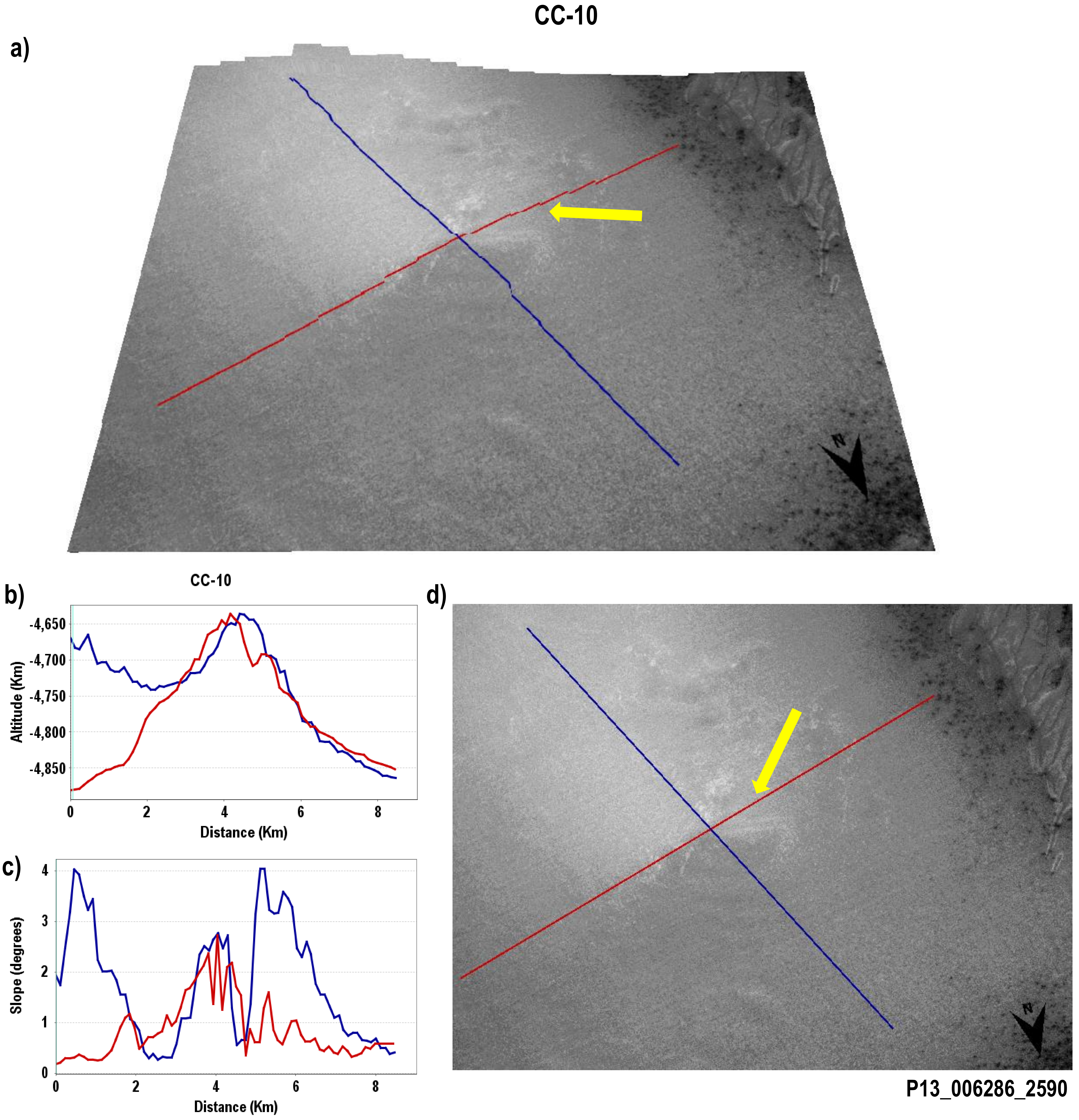
CC-10
a)
N
b)
CC-10
Altitude (Km)
-4,650
-4,700
-4,750
-4,800
-4,850
0
2
4
6
8
Distance (Km)
c)
Slope (degrees)
4
3
2
1
0
0
2
4
6
8
Distance (Km)
d)
N
P13_006286_2590

CC-11

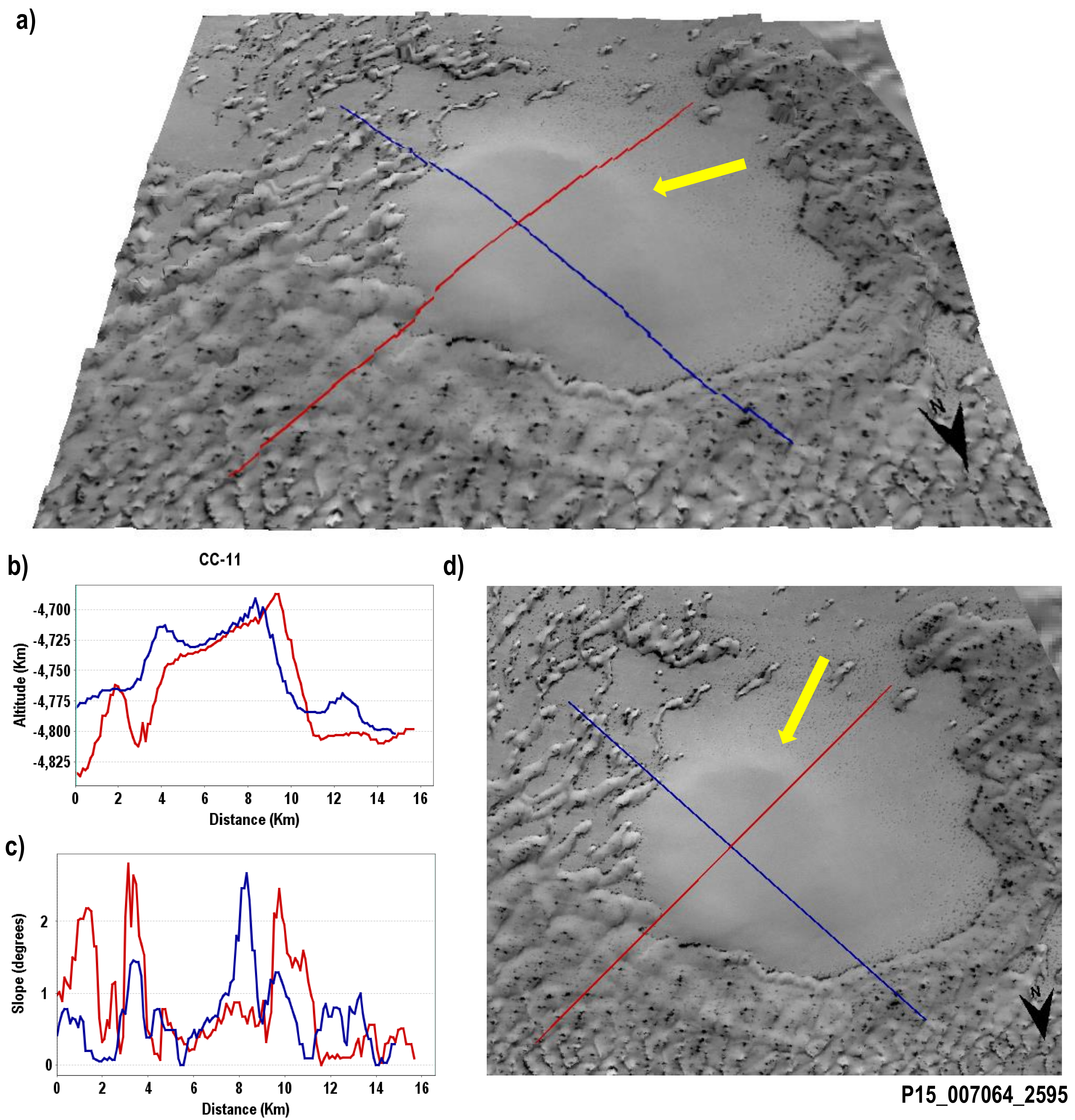

a)
N
b)
CC-11
Altitude (Km)
-4,700
-4,725
-4,750
-4,775
-4,800
-4,825
0 2 4 6 8 10 12 14 16
Distance (Km)
c)
Slope (degrees)
0
1
2
0 2 4 6 8 10 12 14 16
Distance (Km)
d)
N
P15_007064_2595

CC-12

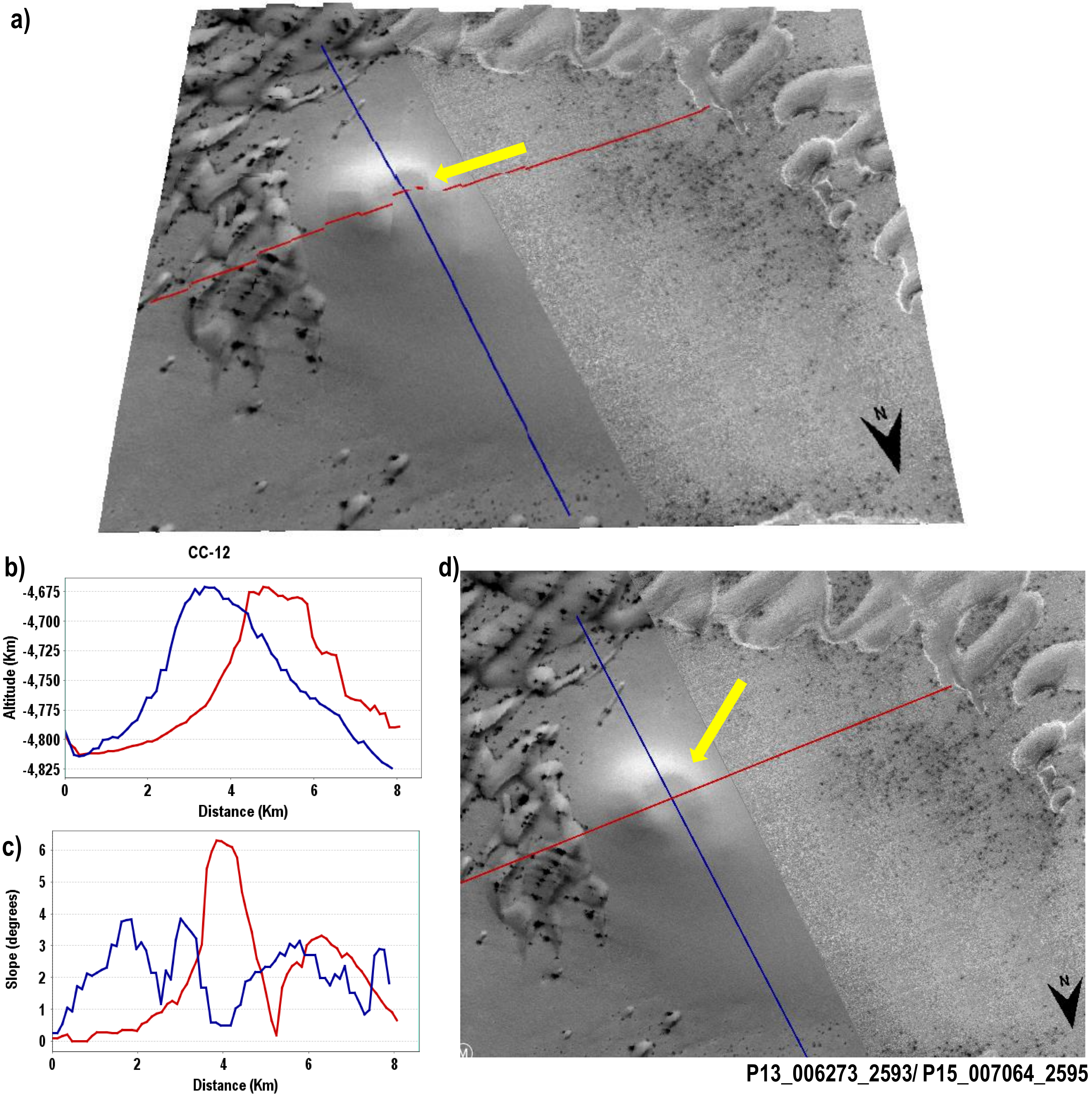
a)
N
CC-12
b)
Altitude (Km)
-4,675
-4,700
-4,725
-4,750
-4,775
-4,800
-4,825
0
2
4
6
8
Distance (Km)
c)
Slope (degrees)
6
5
4
3
2
1
0
0
2
4
6
8
Distance (Km)
d)
N
P13_006273_2593/ P15_007064_2595

CC-13

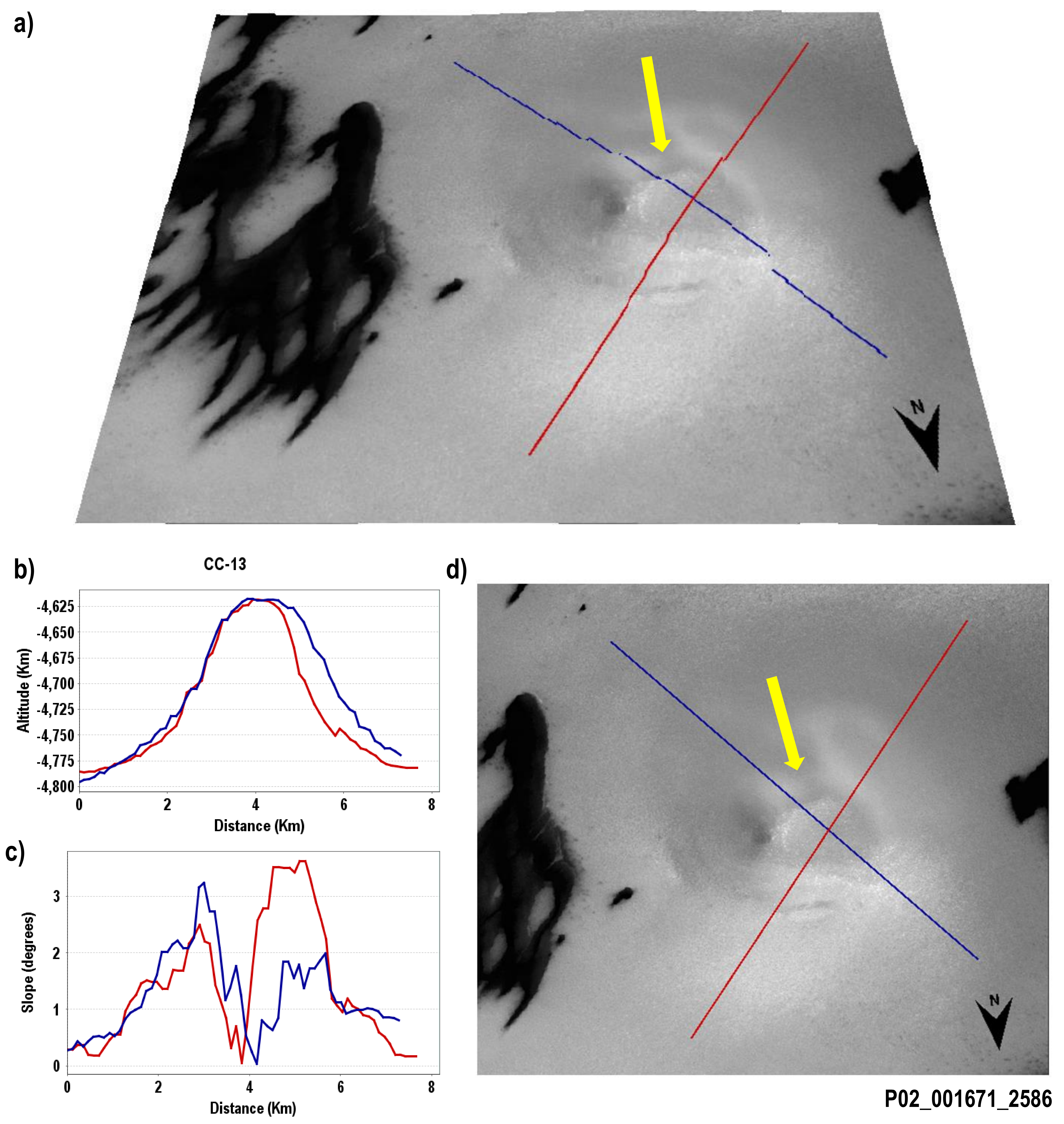
a)
N
b)
CC-13
-4,625
-4,650
-4,675
-4,700
-4,725
-4,750
-4,775
-4,800
Altitude (Km)
0
2
4
6
8
Distance (Km)
c)
3
2
1
0
Slope (degrees)
0
2
4
6
8
Distance (Km)
d)
N
P02_001671_2586

CC-14

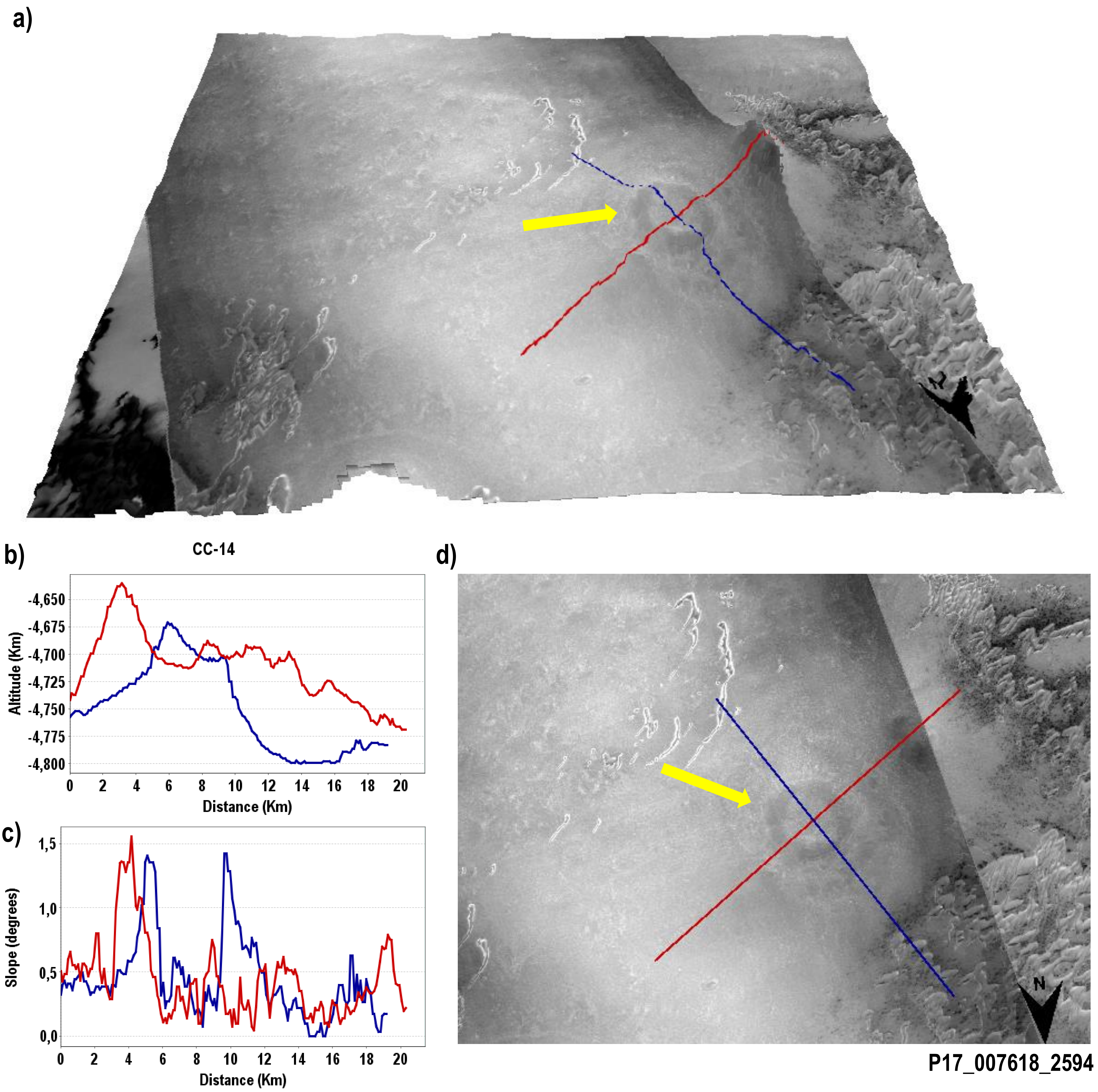

a)
b)
CC-14
Altitude (Km)
-4,650
-4,675
-4,700
-4,725
-4,750
-4,775
-4,800
Distance (Km)
0 2 4 6 8 10 12 14 16 18 20
c)
Slope (degrees)
1,5
1,0
0,5
0,0
Distance (Km)
0 2 4 6 8 10 12 14 16 18 20
d)
N
P17_007618_2594

CC-15

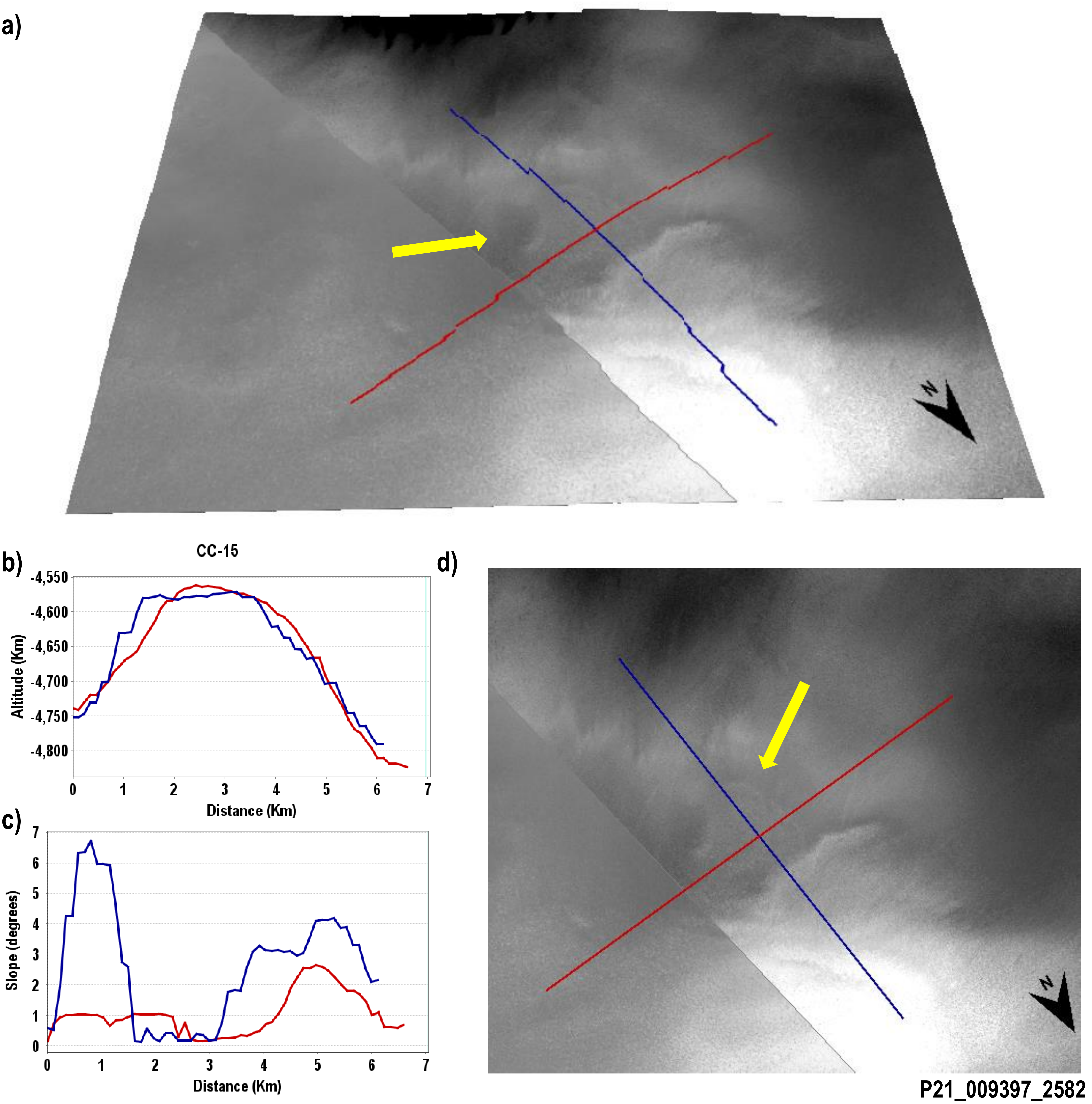
a)
b)
CC-15
Altitude (Km)
-4,550
-4,600
-4,650
-4,700
-4,750
-4,800
0 1 2 3 4 5 6 7
Distance (Km)
c)
Slope (degrees)
0 1 2 3 4 5 6 7
Distance (Km)
d)
N
P21_009397_2582

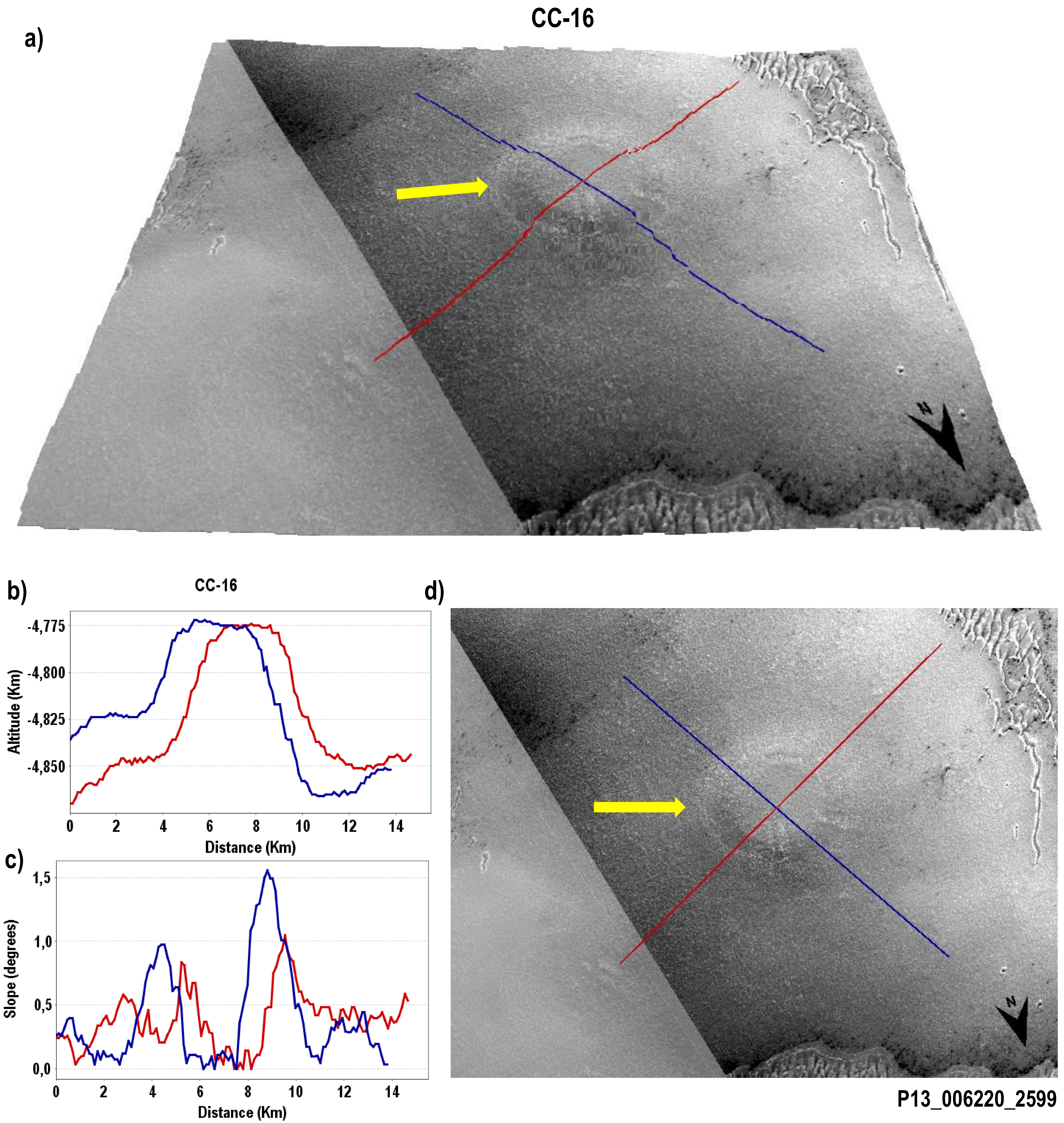
CC-16
a)
N
b)
CC-16
-4,775
-4,800
-4,825
-4,850
Altitude (Km)
0
2
4
6
8
10
12
14
Distance (Km)
c)
1,5
1,0
0,5
0,0
Slope (degrees)
0
2
4
6
8
10
12
14
Distance (Km)
d)
N
P13_006220_2599

CC-17

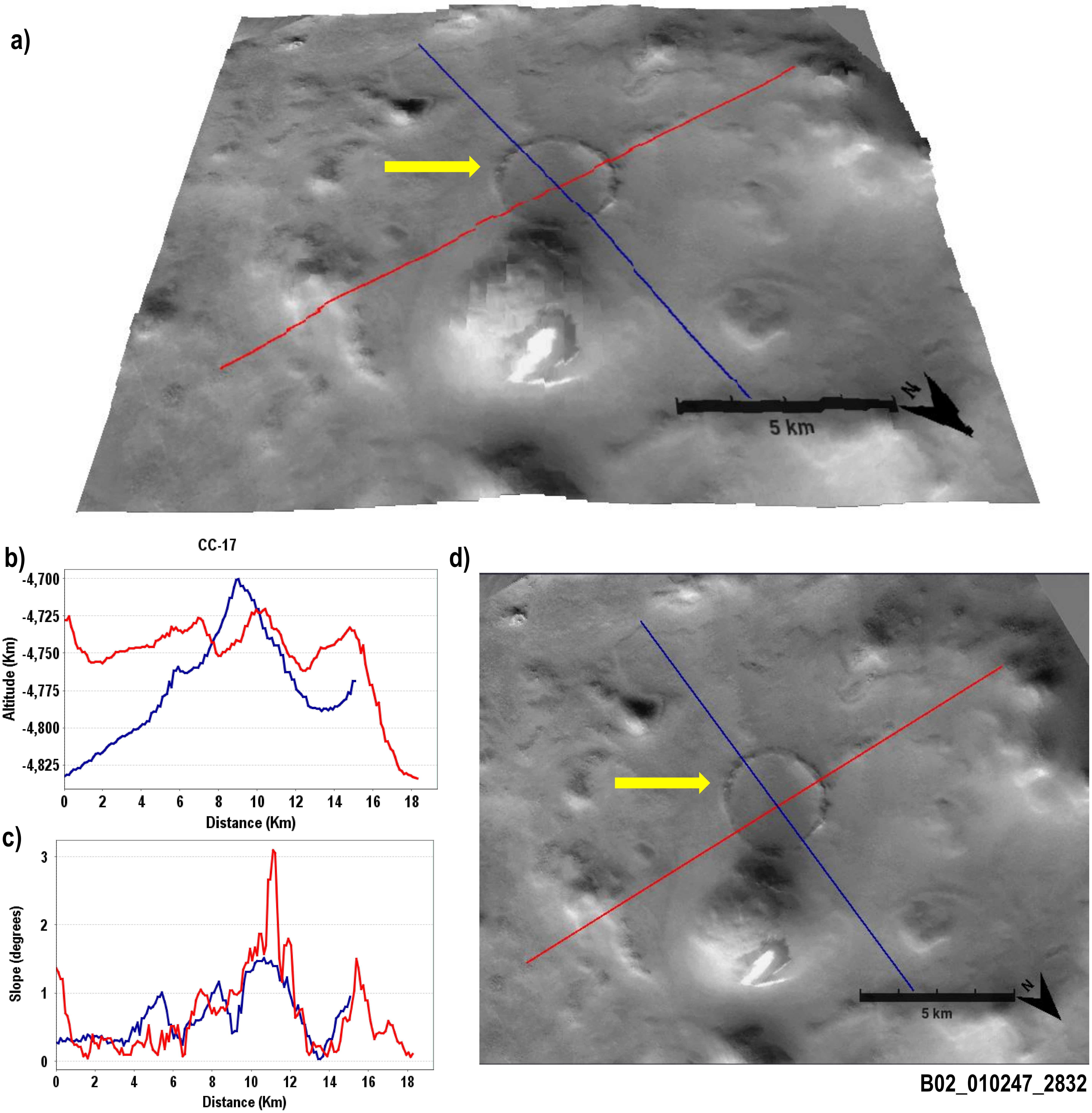

B02_010247_2832

CC-18

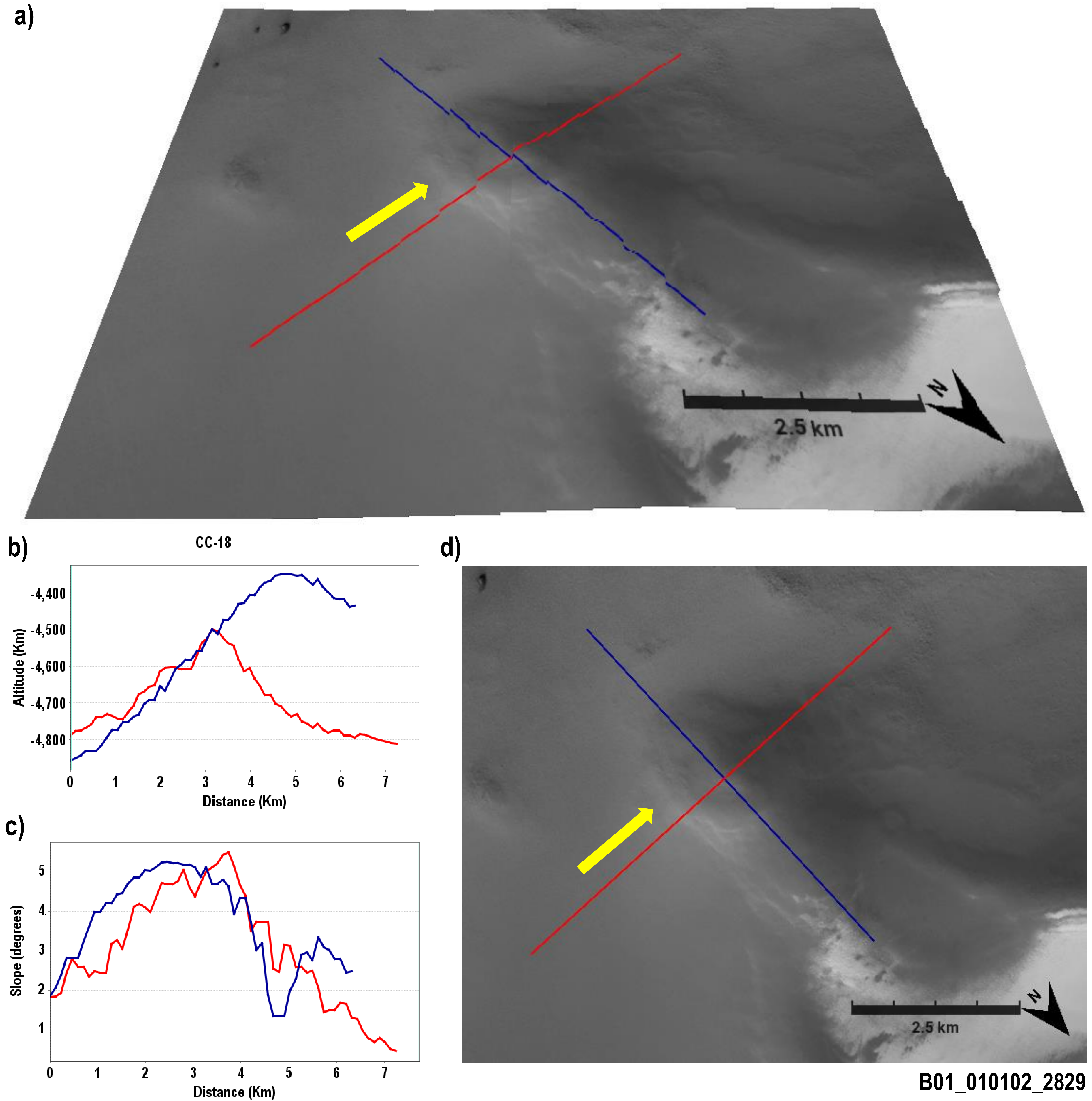
a)
2.5 km
N
b)
CC-18
Altitude (Km)
-4,400
-4,500
-4,600
-4,700
-4,800
Distance (Km)
0 1 2 3 4 5 6 7
c)
Slope (degrees)
5
4
3
2
1
Distance (Km)
0 1 2 3 4 5 6 7
d)
2.5 km
N
B01_010102_2829

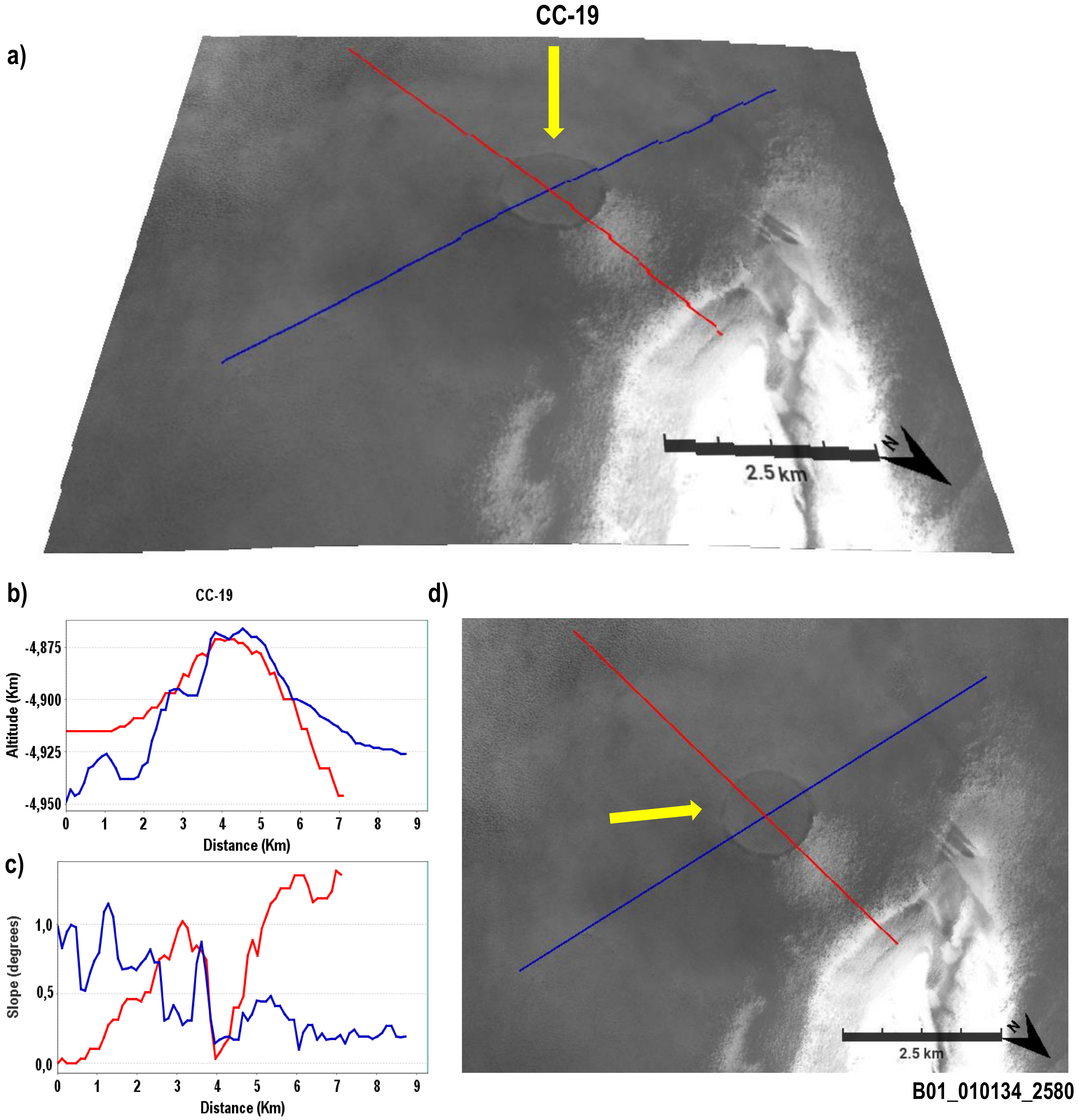
CC-19
a)
2.5 km
N
b)
CC-19
Altitude (Km)
-4,875
-4,900
-4,925
-4,950
Distance (Km)
c)
Slope (degrees)
1,0
0,5
0,0
Distance (Km)
d)
2.5 km
N
B01_010134_2580

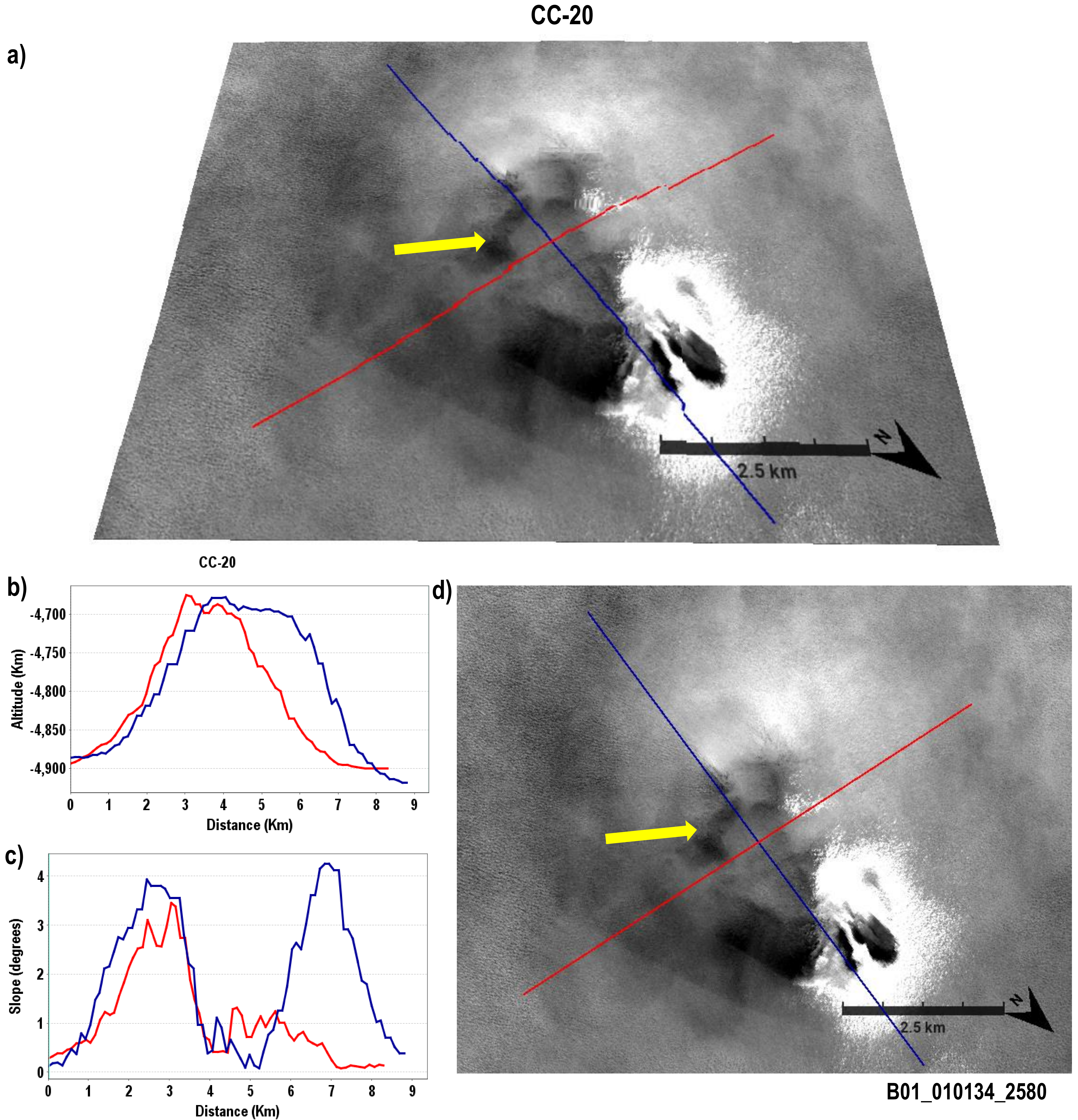
CC-20
a)
2.5 km
N
CC-20
b)
Altitude (Km)
-4,700
-4,750
-4,800
-4,850
-4,900
0 1 2 3 4 5 6 7 8 9
Distance (Km)
c)
Slope (degrees)
0 1 2 3 4
0 1 2 3 4 5 6 7 8 9
Distance (Km)
d)
2.5 km
N
B01_010134_2580

CC-21

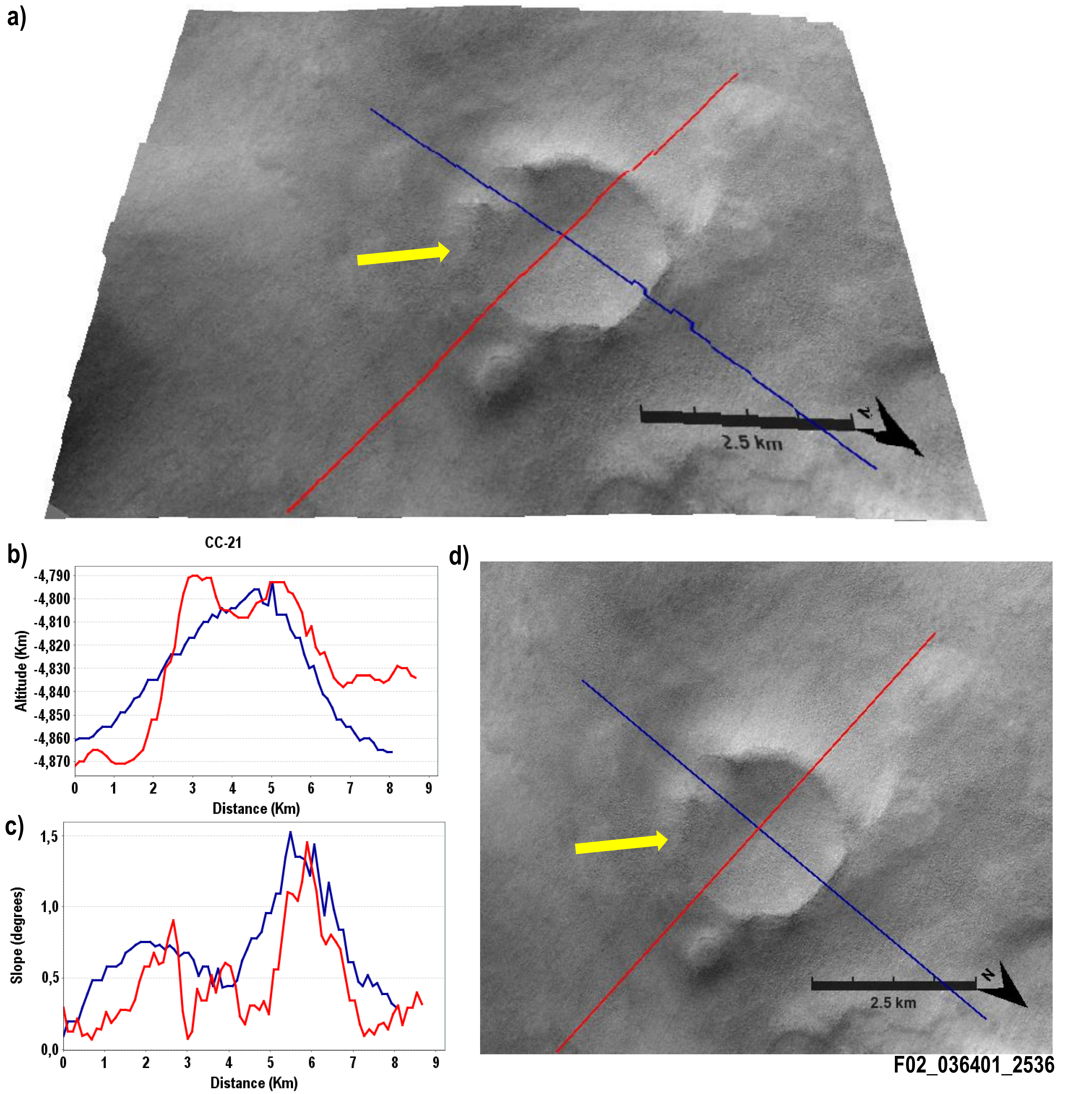
a)
2.5 km
b)
CC-21
Altitude (Km)
-4,790
-4,800
-4,810
-4,820
-4,830
-4,840
-4,850
-4,860
-4,870
Distance (Km)
0 1 2 3 4 5 6 7 8 9
c)
Slope (degrees)
1,5
1,0
0,5
0,0
Distance (Km)
0 1 2 3 4 5 6 7 8 9
d)
2.5 km
F02_036401_2536

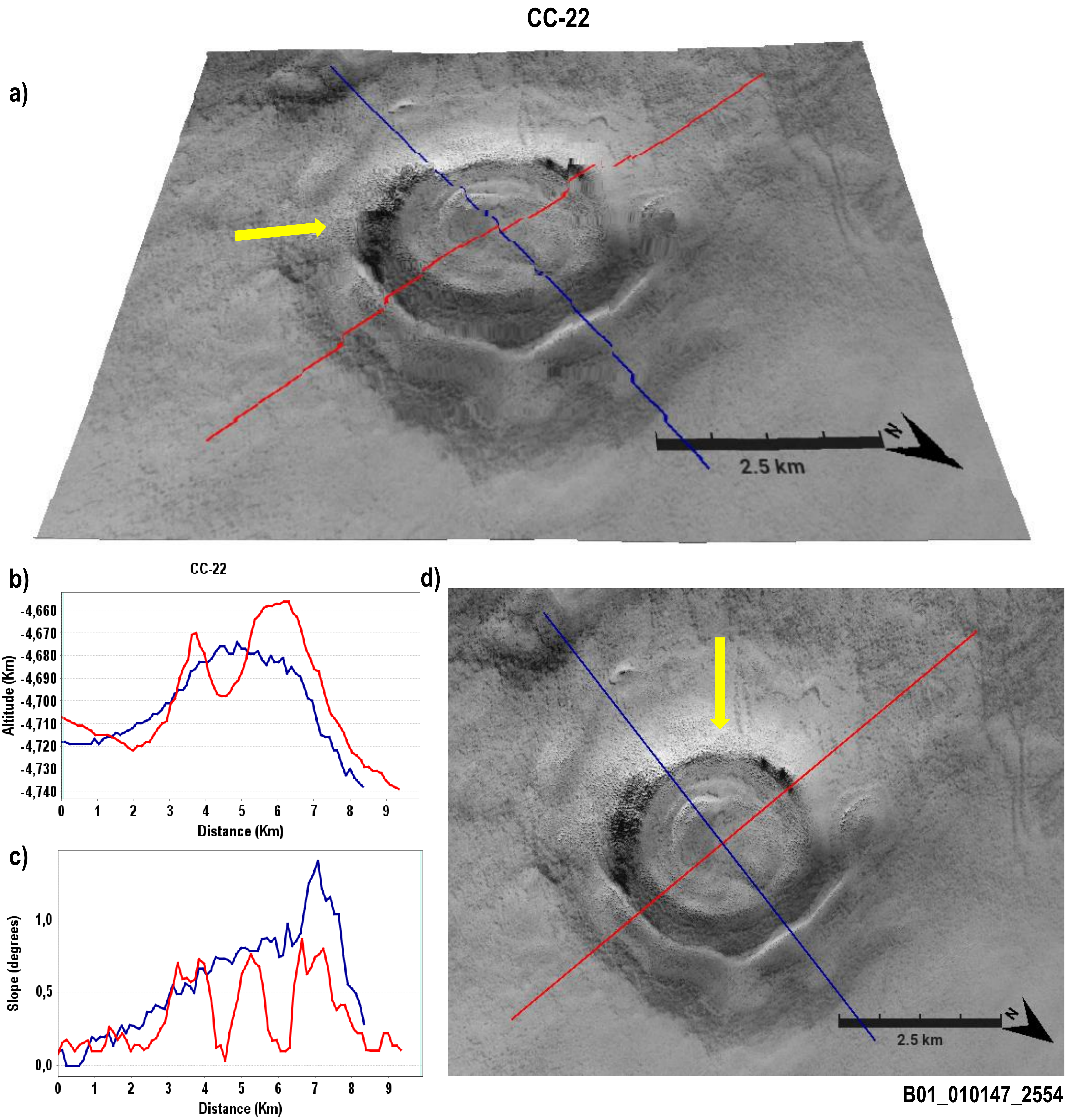
CC-22
a)
2.5 km
N
b)
CC-22
Altitude (Km)
-4,660
-4,670
-4,680
-4,690
-4,700
-4,710
-4,720
-4,730
-4,740
Distance (Km)
0 1 2 3 4 5 6 7 8 9
c)
Slope (degrees)
0,0
0,5
1,0
Distance (Km)
0 1 2 3 4 5 6 7 8 9
d)
2.5 km
N
B01_010147_2554

CC-23

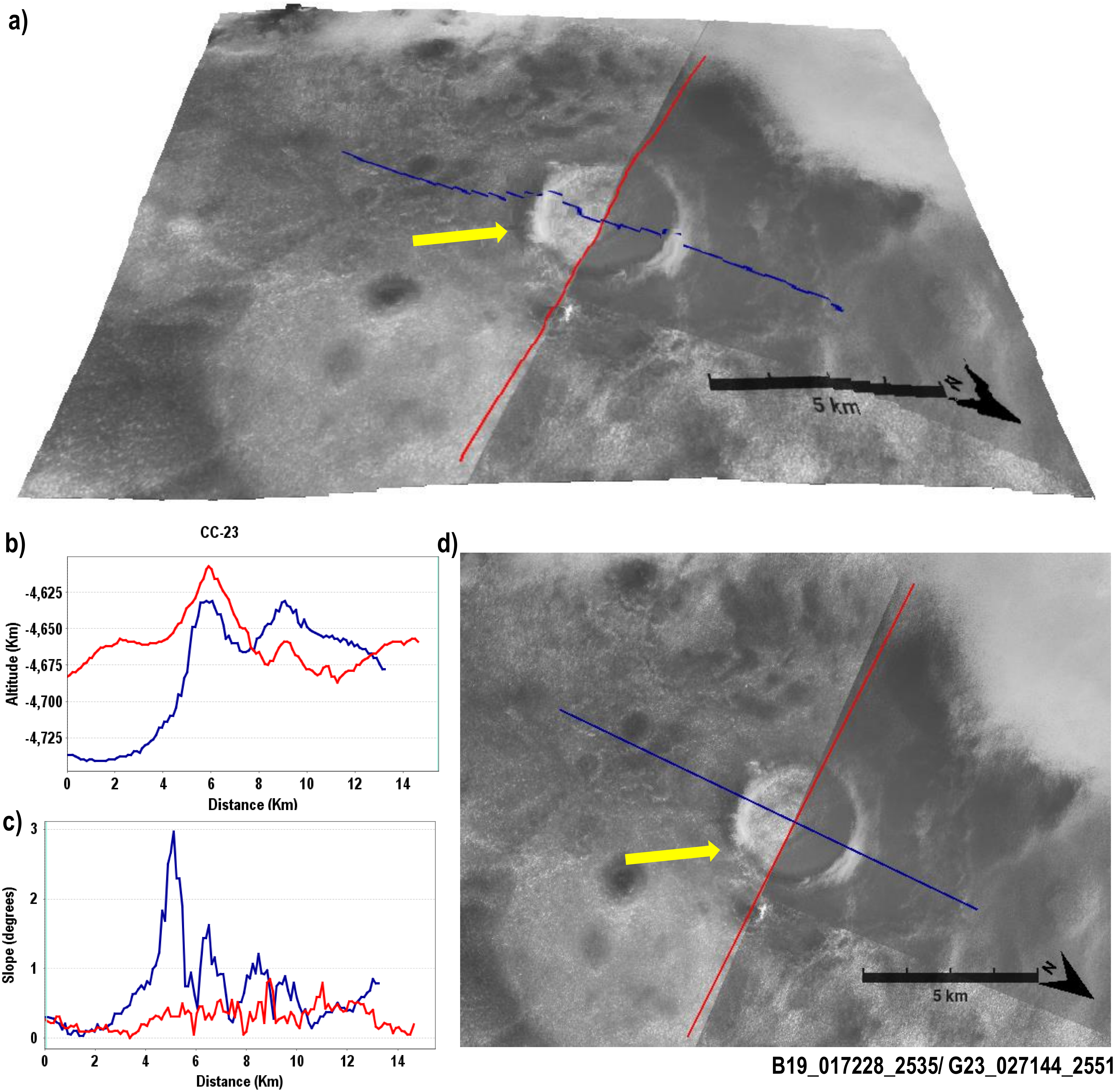

B19_017228_2535/ G23_027144_2551

CC-24

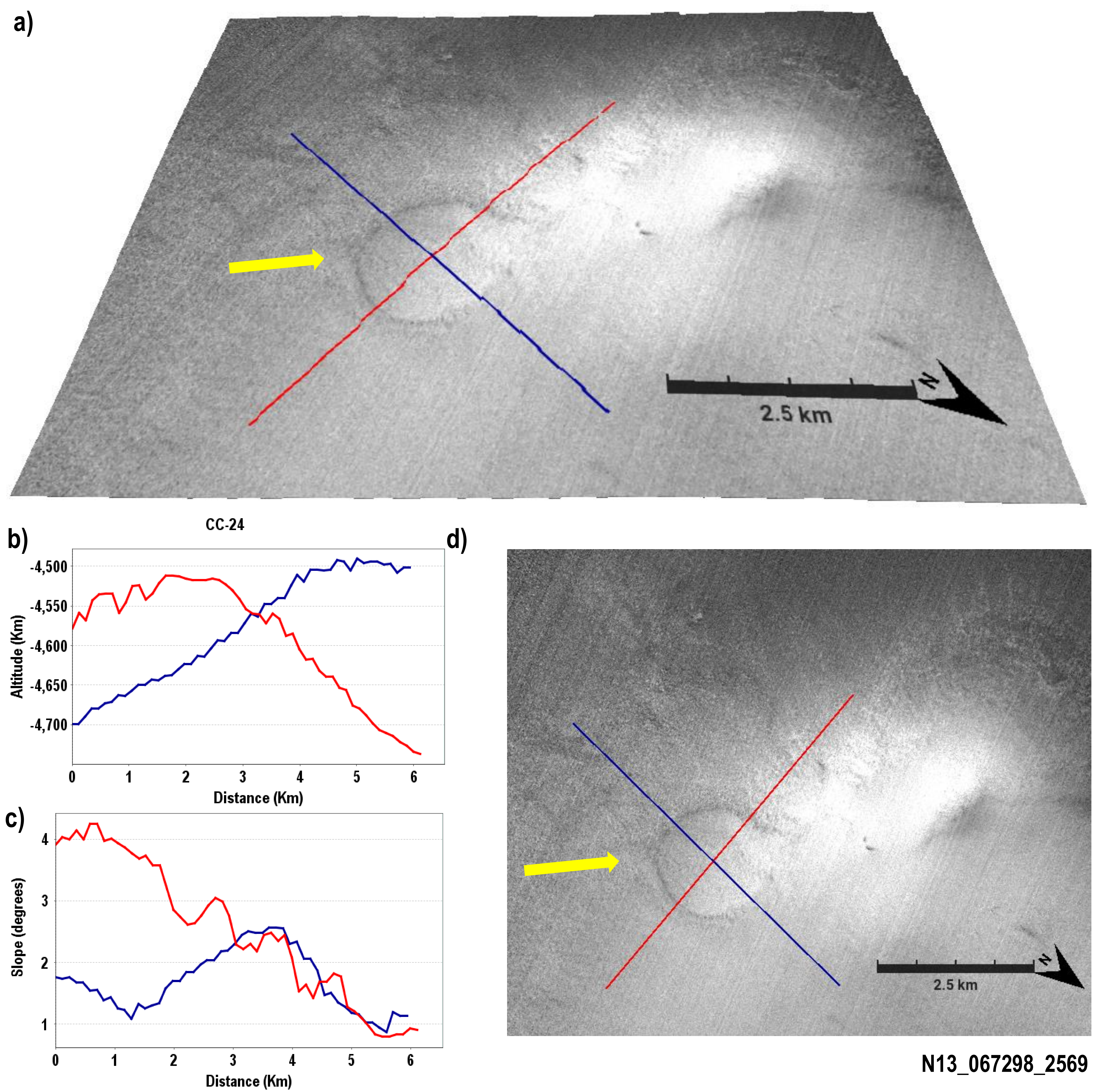

N13_067298_2569

CC-25

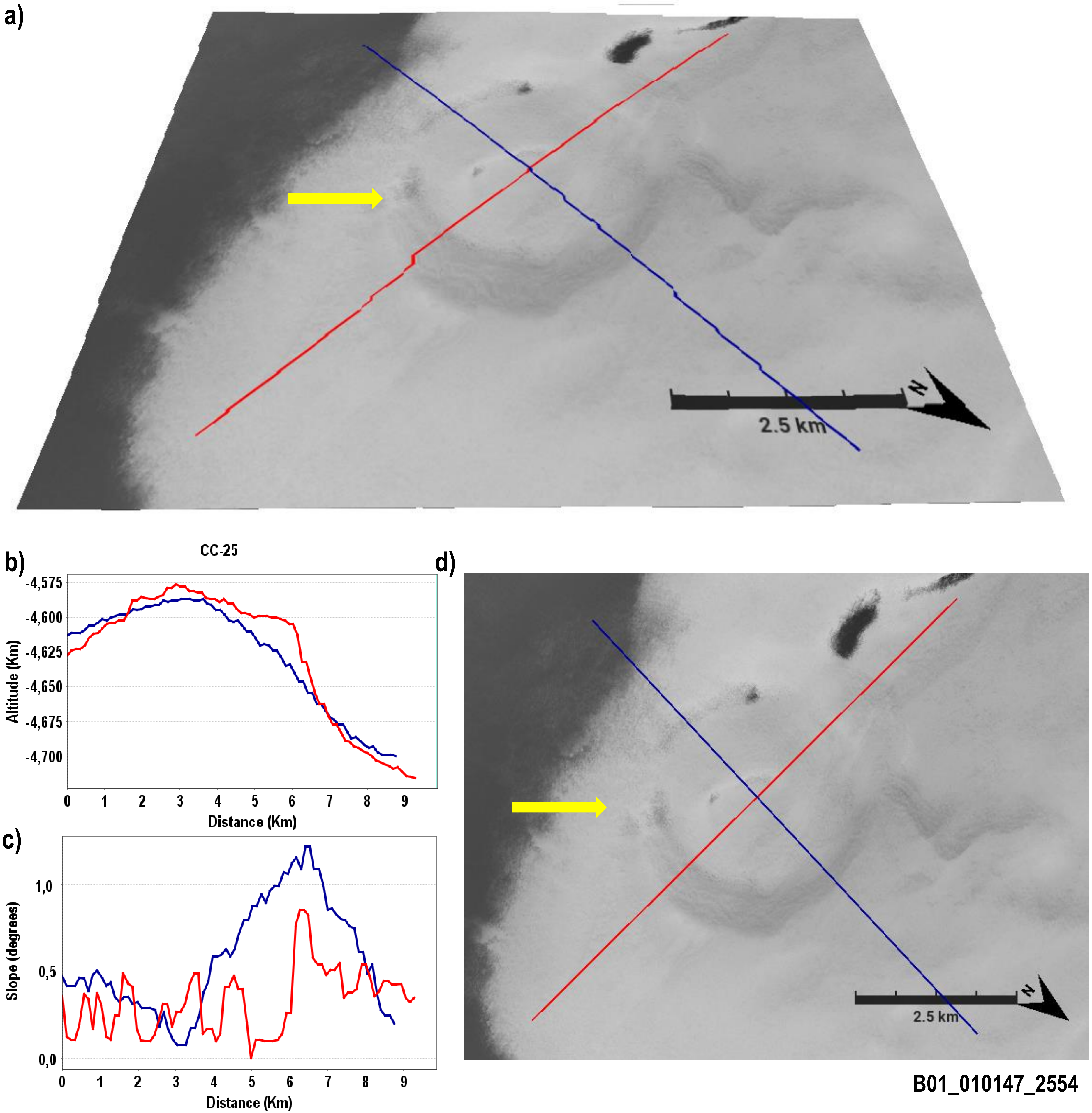
a)
2.5 km
N
b)
CC-25
-4,575
-4,600
-4,625
-4,650
-4,675
-4,700
Altitude (Km)
0 1 2 3 4 5 6 7 8 9
Distance (Km)
c)
1,0
0,5
0,0
Slope (degrees)
0 1 2 3 4 5 6 7 8 9
Distance (Km)
d)
2.5 km
N
B01_010147_2554

CC-26

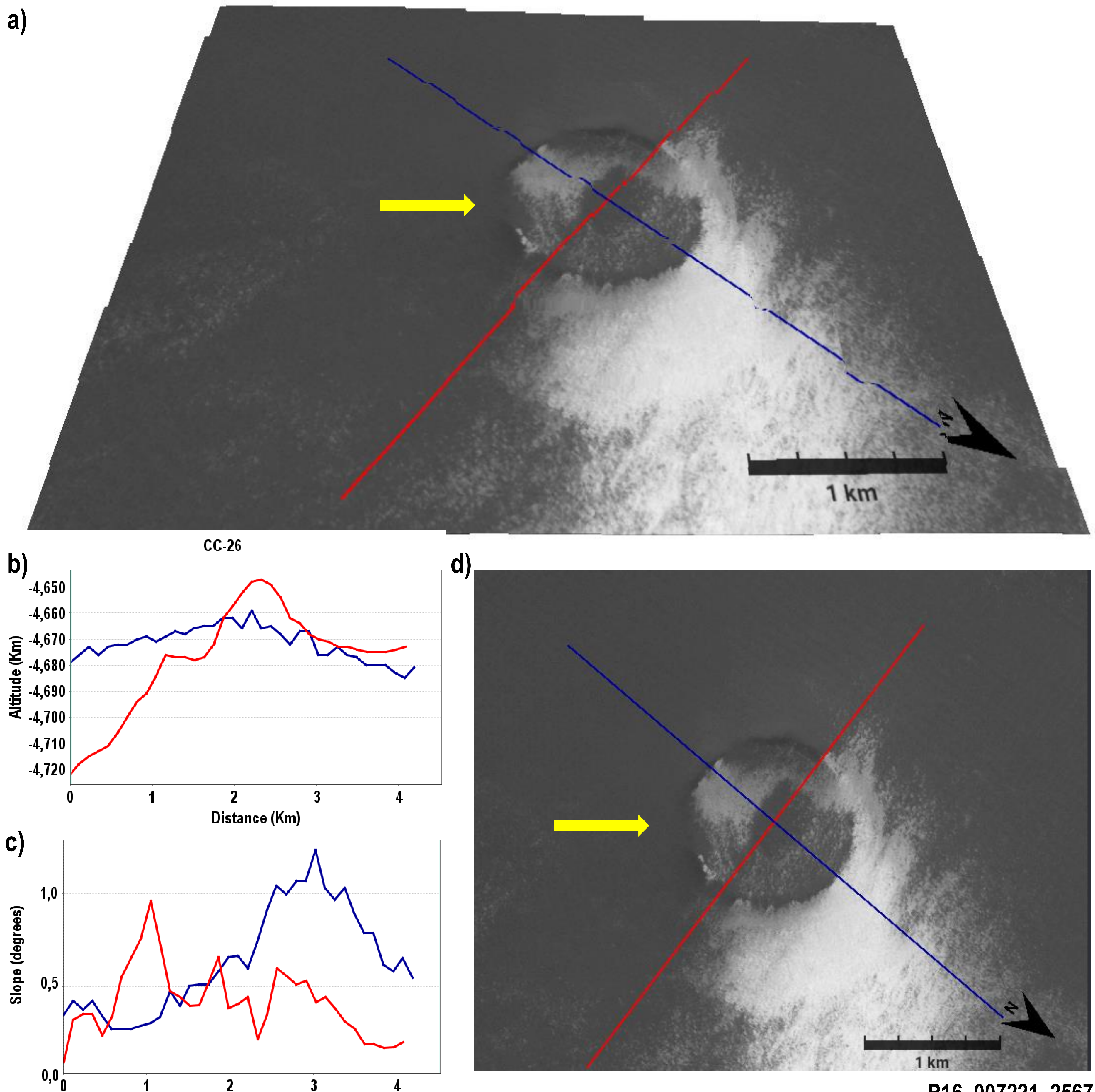

P16_007221_2567

CC-27

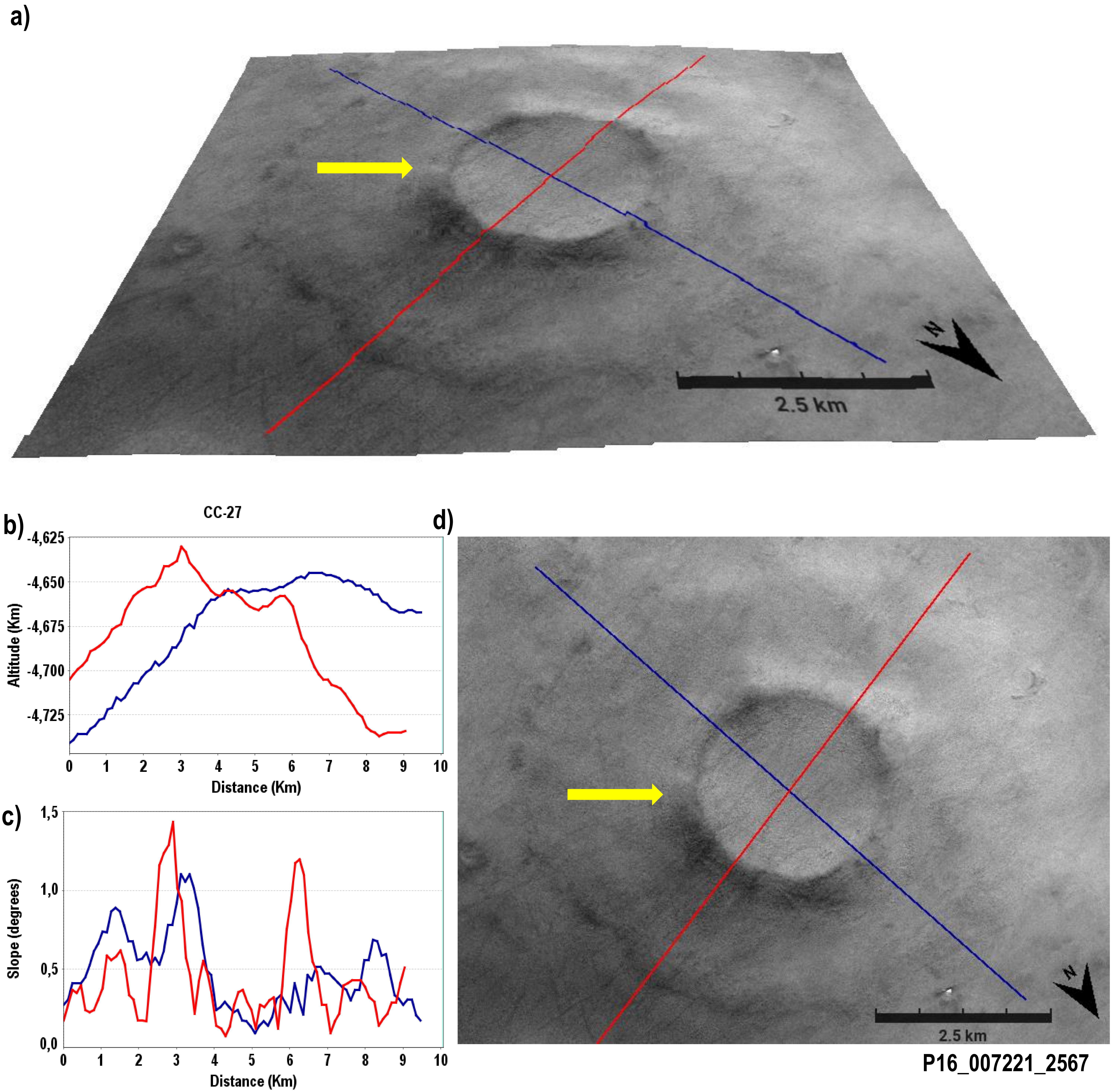
a)
2.5 km
N
b)
CC-27
-4,625
-4,650
-4,675
-4,700
-4,725
Altitude (Km)
0 1 2 3 4 5 6 7 8 9 10
Distance (Km)
c)
1,5
1,0
0,5
0,0
Slope (degrees)
0 1 2 3 4 5 6 7 8 9 10
Distance (Km)
d)
2.5 km
N
P16_007221_2567

CC-28

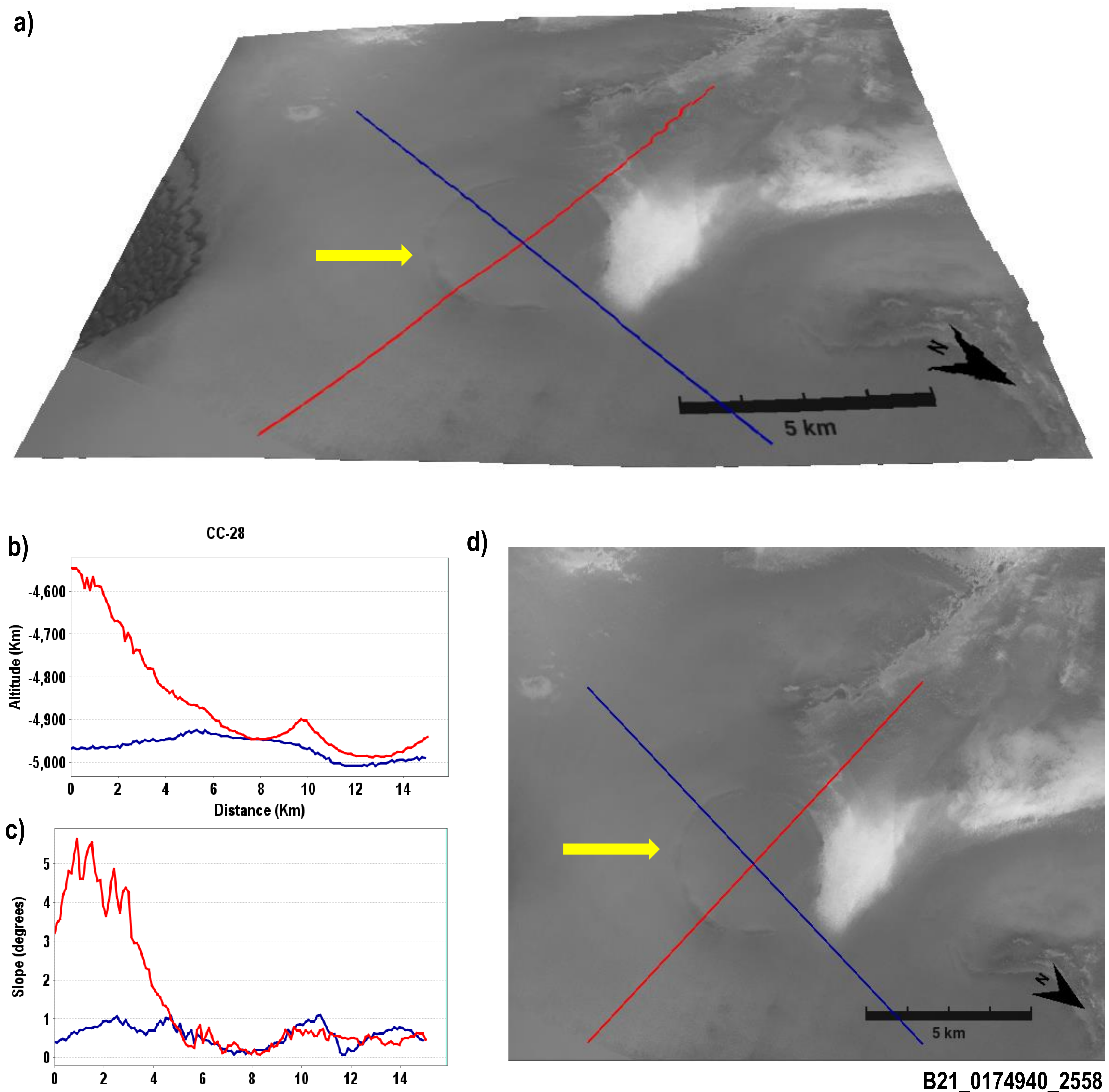

B21_0174940_2558

CC-29

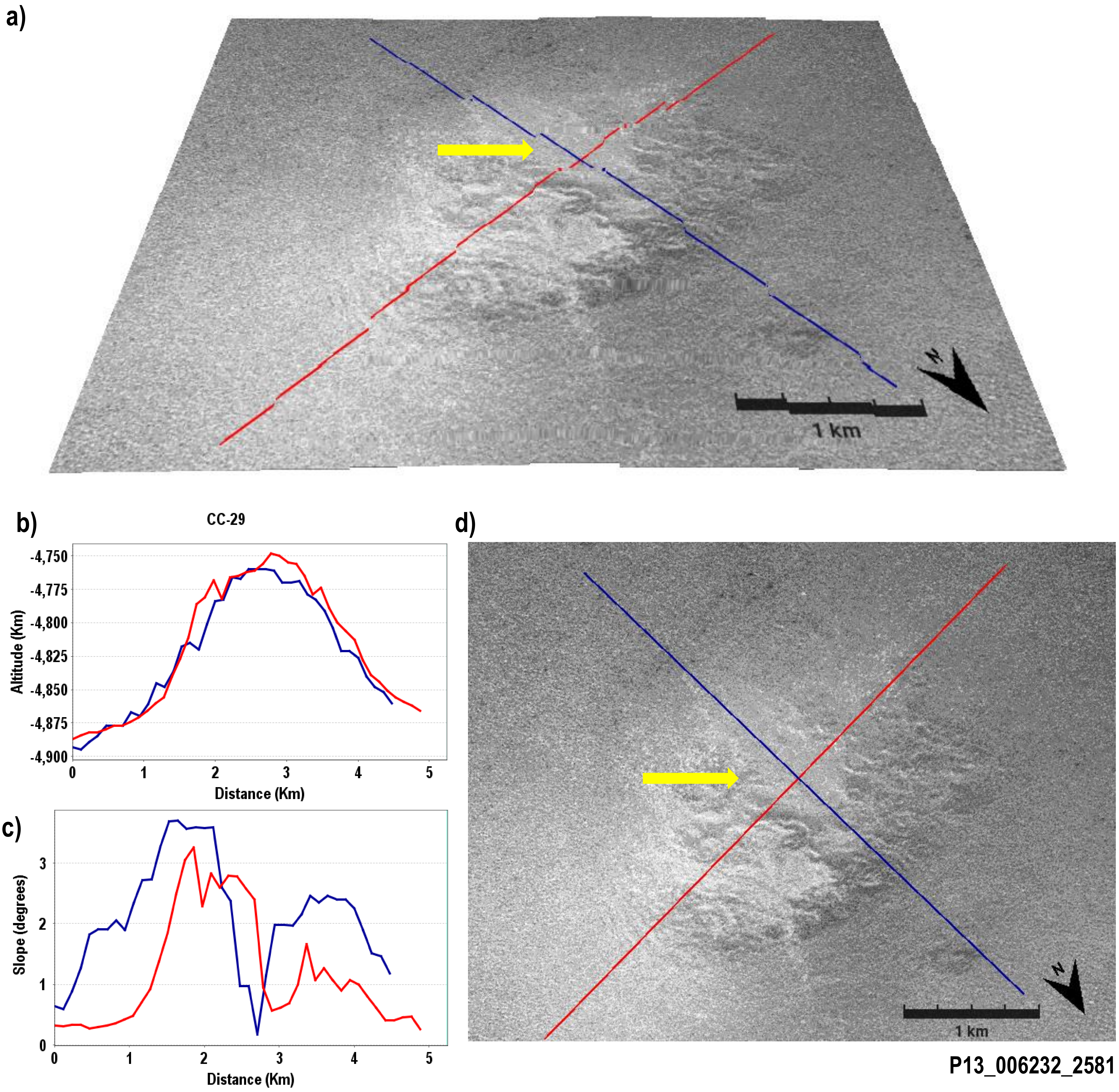
a)
1 km
N
b)
CC-29
Altitude (Km)
-4,750
-4,775
-4,800
-4,825
-4,850
-4,875
-4,900
Distance (Km)
0
1
2
3
4
5
c)
Slope (degrees)
0
1
2
3
Distance (Km)
d)
1 km
N
P13_006232_2581

CC-30

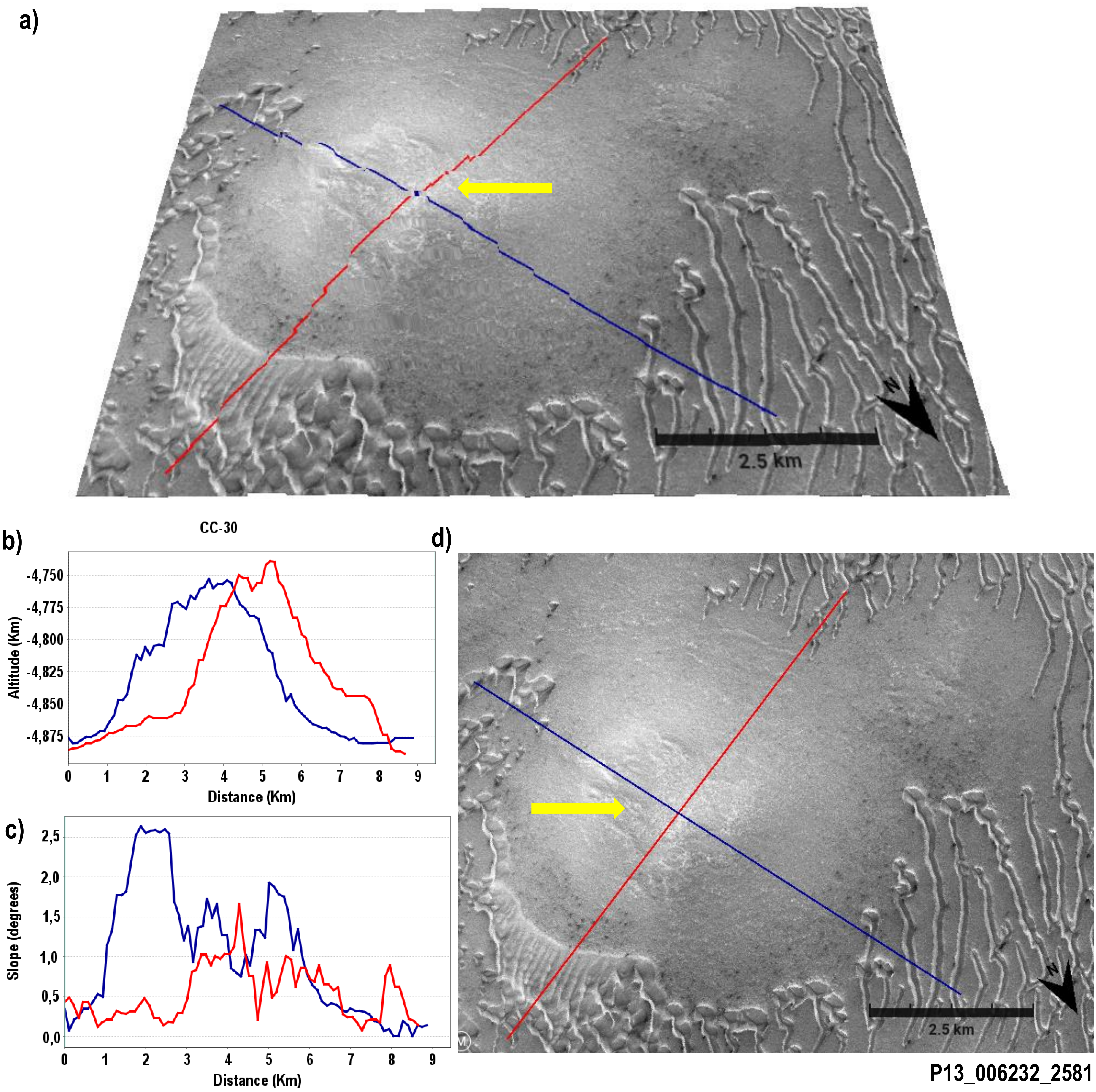
a)
2.5 km
N
b)
CC-30
Altitude (Km)
-4,750
-4,775
-4,800
-4,825
-4,850
-4,875
Distance (Km)
0
1
2
3
4
5
6
7
8
9
c)
Slope (degrees)
2,5
2,0
1,5
1,0
0,5
0,0
Distance (Km)
d)
2.5 km
N
P13_006232_2581

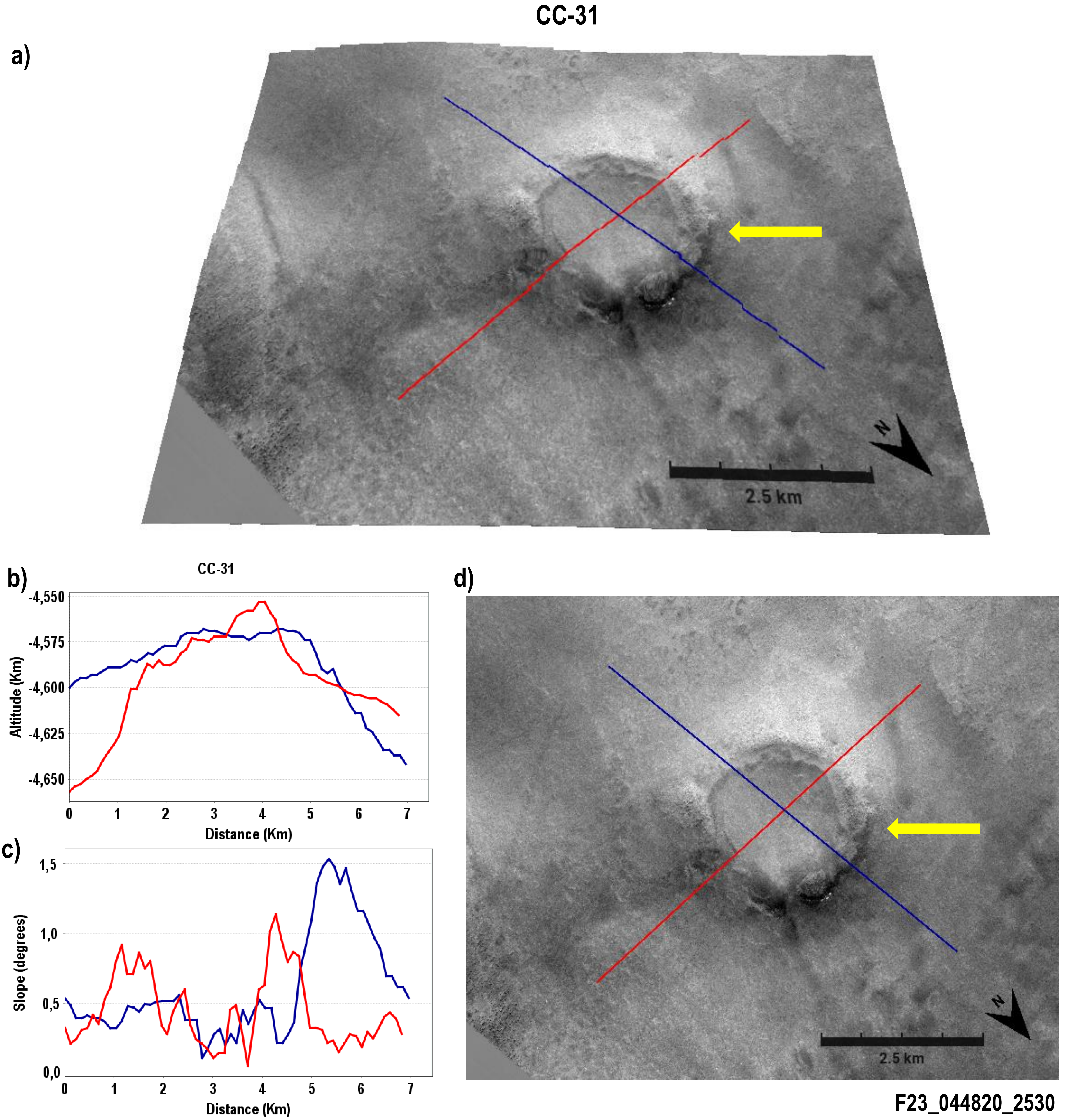
CC-31
a)
2.5 km
N
b)
CC-31
-4,550
-4,575
-4,600
-4,625
-4,650
Altitude (Km)
0 1 2 3 4 5 6 7
Distance (Km)
c)
1,5
1,0
0,5
0,0
Slope (degrees)
0 1 2 3 4 5 6 7
Distance (Km)
d)
2.5 km
N
F23_044820_2530

CC-32

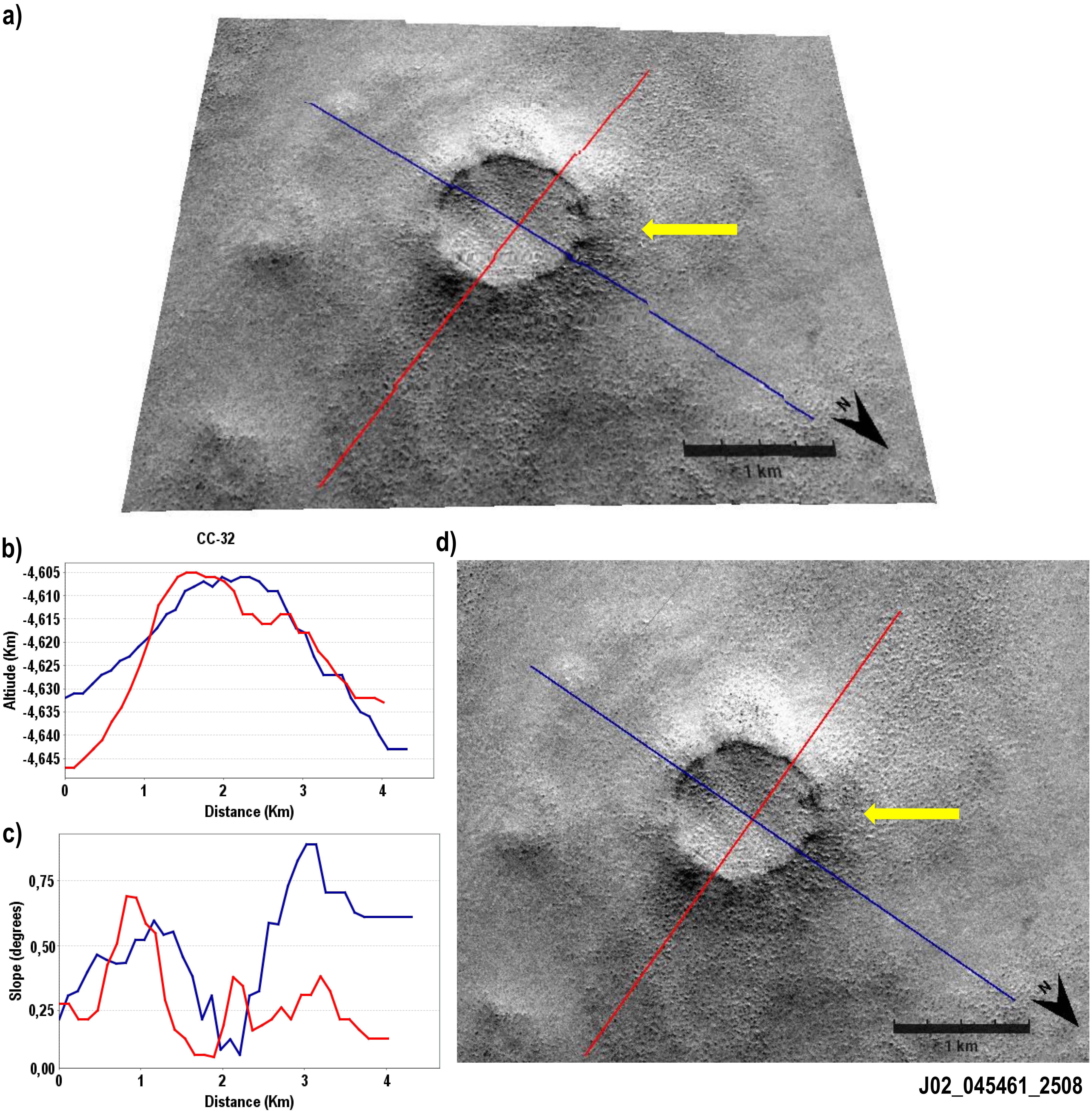
a)
1 km
N
b)
CC-32
Altiude (Km)
-4,605
-4,610
-4,615
-4,620
-4,625
-4,630
-4,635
-4,640
-4,645
0
1
2
3
4
Distance (Km)
c)
Slope (degrees)
0,75
0,50
0,25
0,00
0
1
2
3
4
Distance (Km)
d)
1 km
N
J02_045461_2508

CC-33

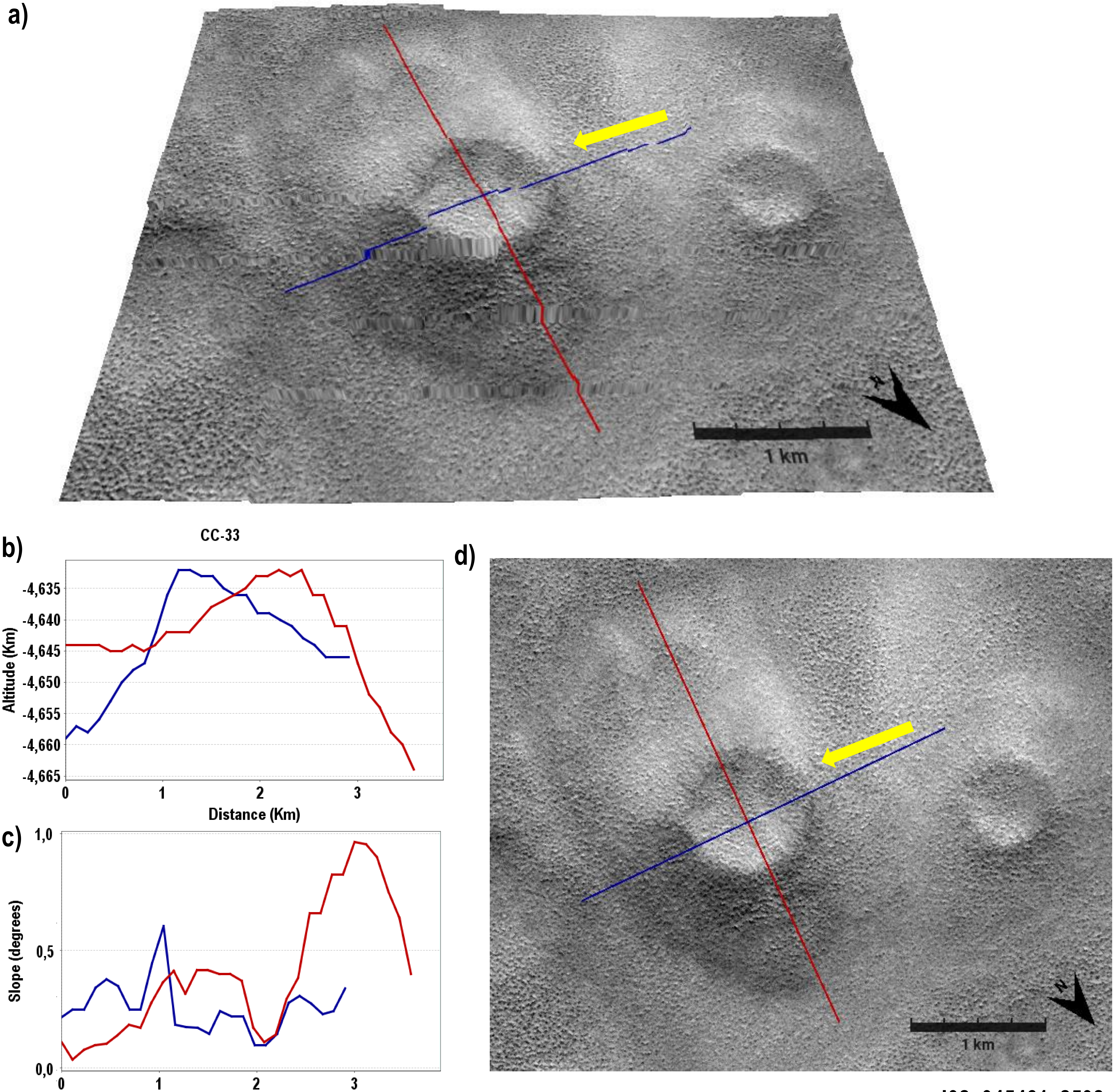

J02_045461_2508

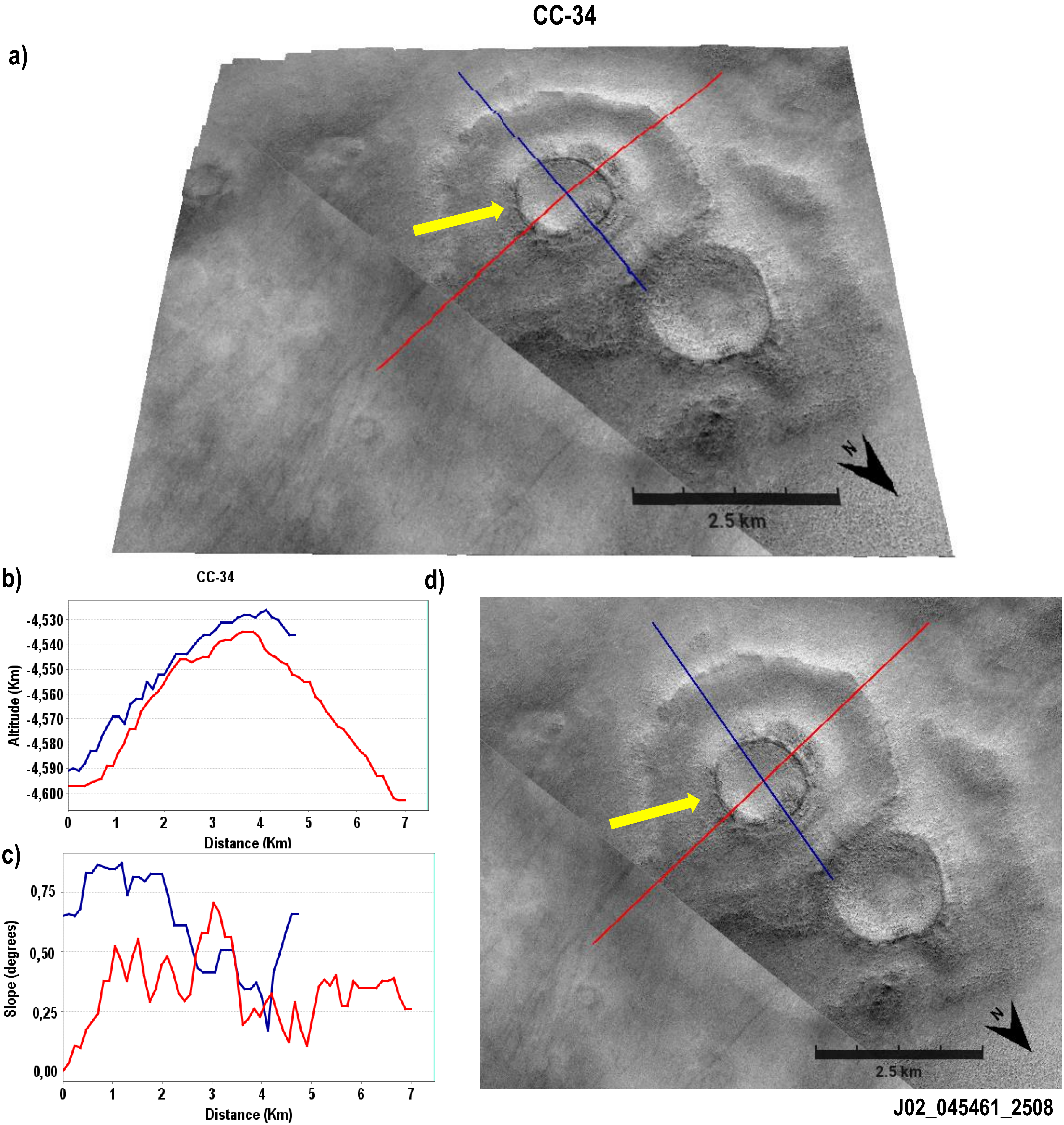

CC-34
a)
2.5 km
N
b)
CC-34
Altitude (Km)
-4,530
-4,540
-4,550
-4,560
-4,570
-4,580
-4,590
-4,600
Distance (Km)
0
1
2
3
4
5
6
7
c)
Slope (degrees)
0,75
0,50
0,25
0,00
Distance (Km)
d)
2.5 km
J02_045461_2508

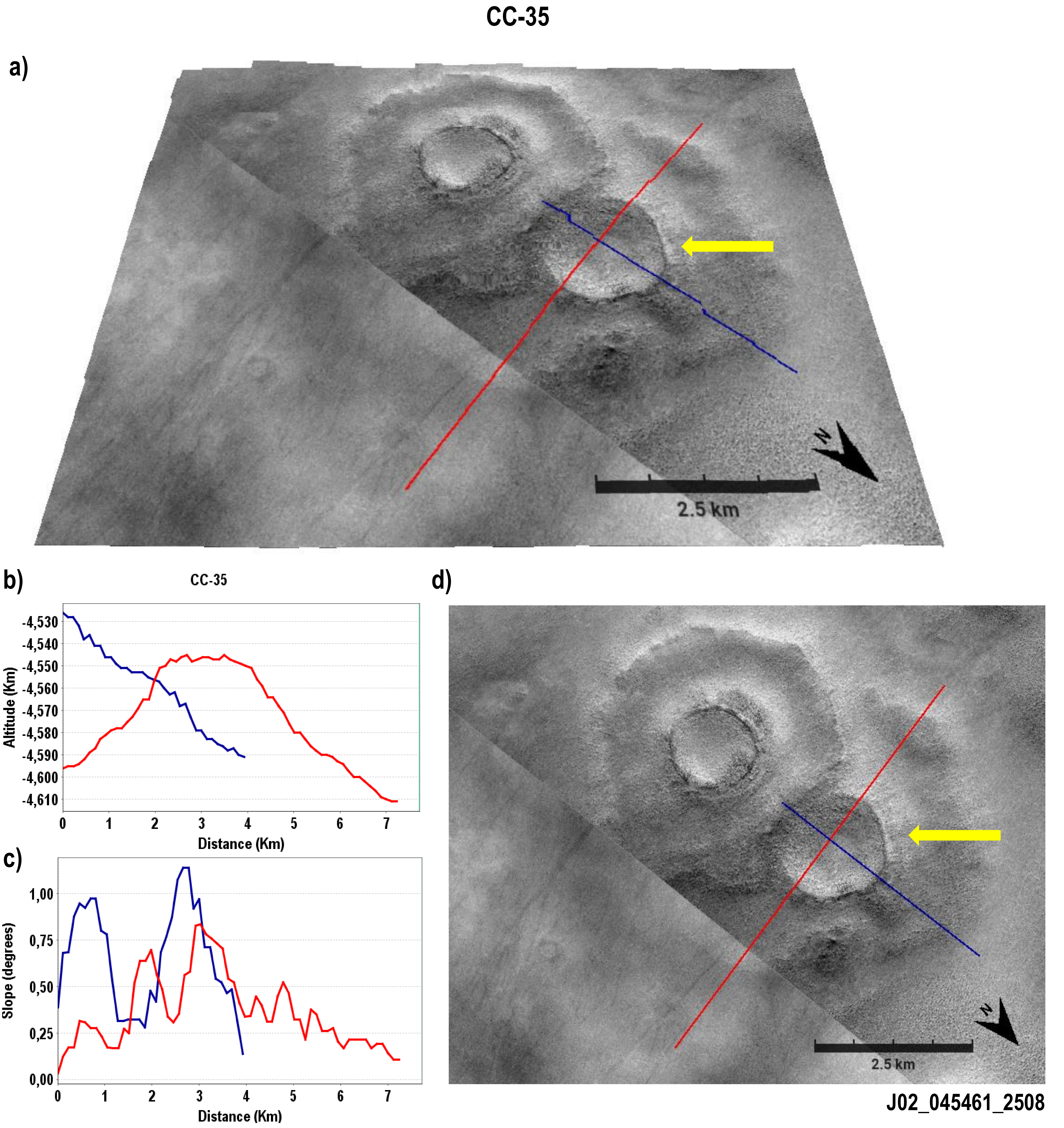

CC-35
a)
2.5 km
N
b)
CC-35
Altitude (Km)
-4,530
-4,540
-4,550
-4,560
-4,570
-4,580
-4,590
-4,600
-4,610
Distance (Km)
c)
Slope (degrees)
1,00
0,75
0,50
0,25
0,00
Distance (Km)
d)
2.5 km
N
J02_045461_2508

CC-36

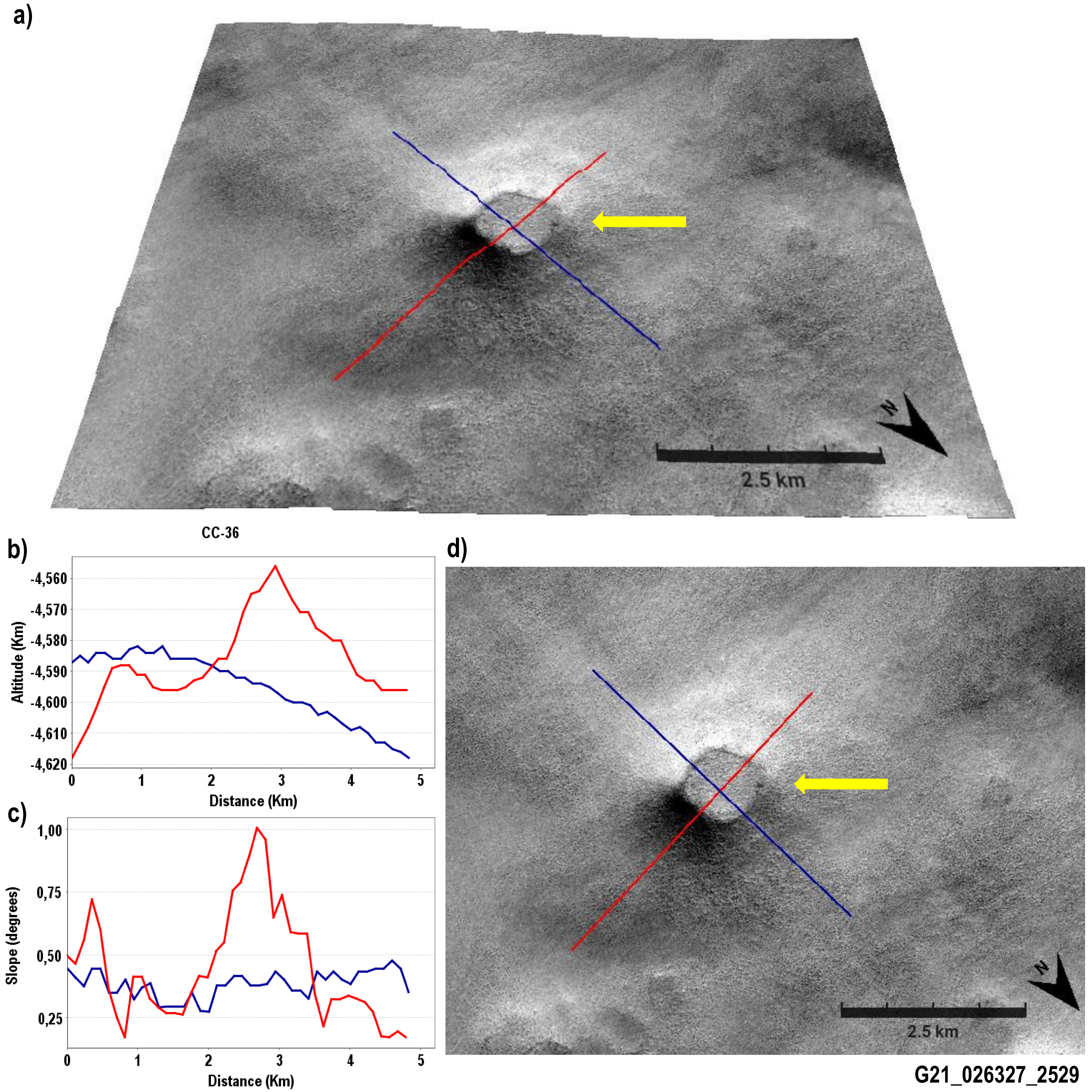
a)
N
2.5 km
CC-36
b)
-4,560
-4,570
-4,580
-4,590
-4,600
-4,610
-4,620
Altitude (Km)
0
1
2
3
4
5
Distance (Km)
c)
1,00
0,75
0,50
0,25
Slope (degrees)
0
1
2
3
4
5
Distance (Km)
d)
N
2.5 km
G21_026327_2529

CC-37

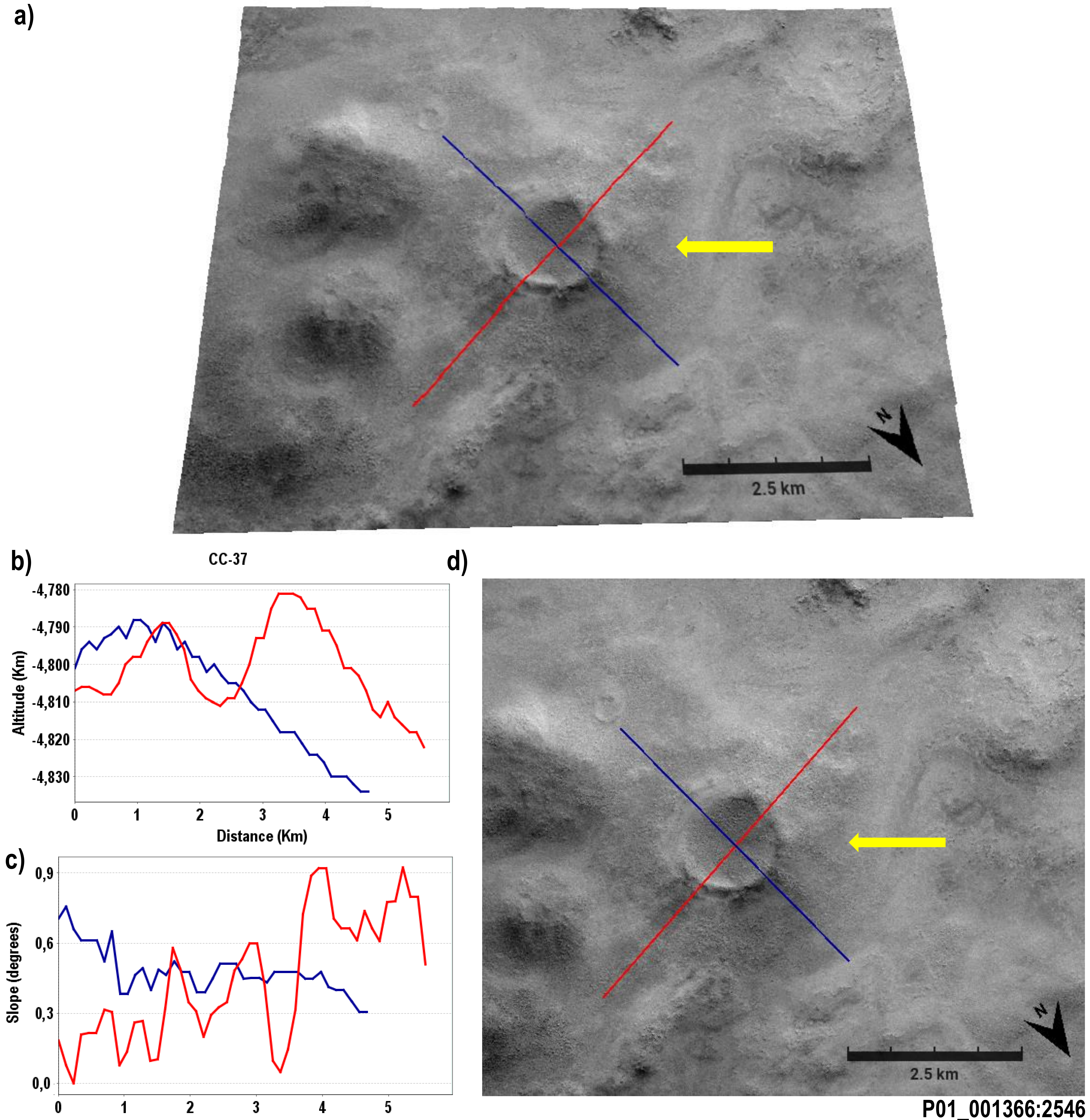

P01_001366:2546

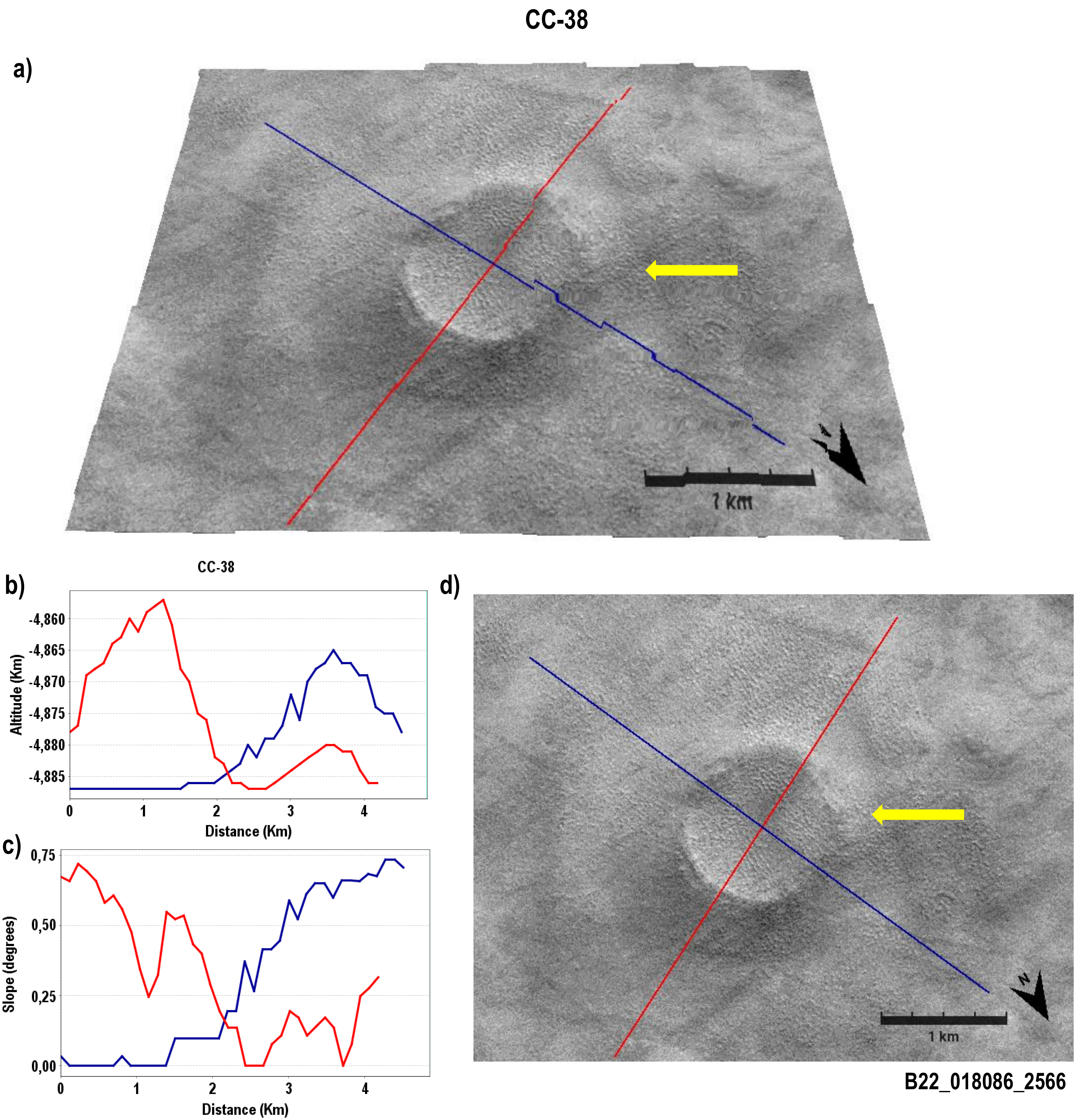
CC-38
a)
1 km
b)
CC-38
Altitude (Km)
-4,860
-4,865
-4,870
-4,875
-4,880
-4,885
0
1
2
3
4
Distance (Km)
c)
Slope (degrees)
0,75
0,50
0,25
0,00
0
1
2
3
4
Distance (Km)
d)
1 km
B22_018086_2566

CC-39

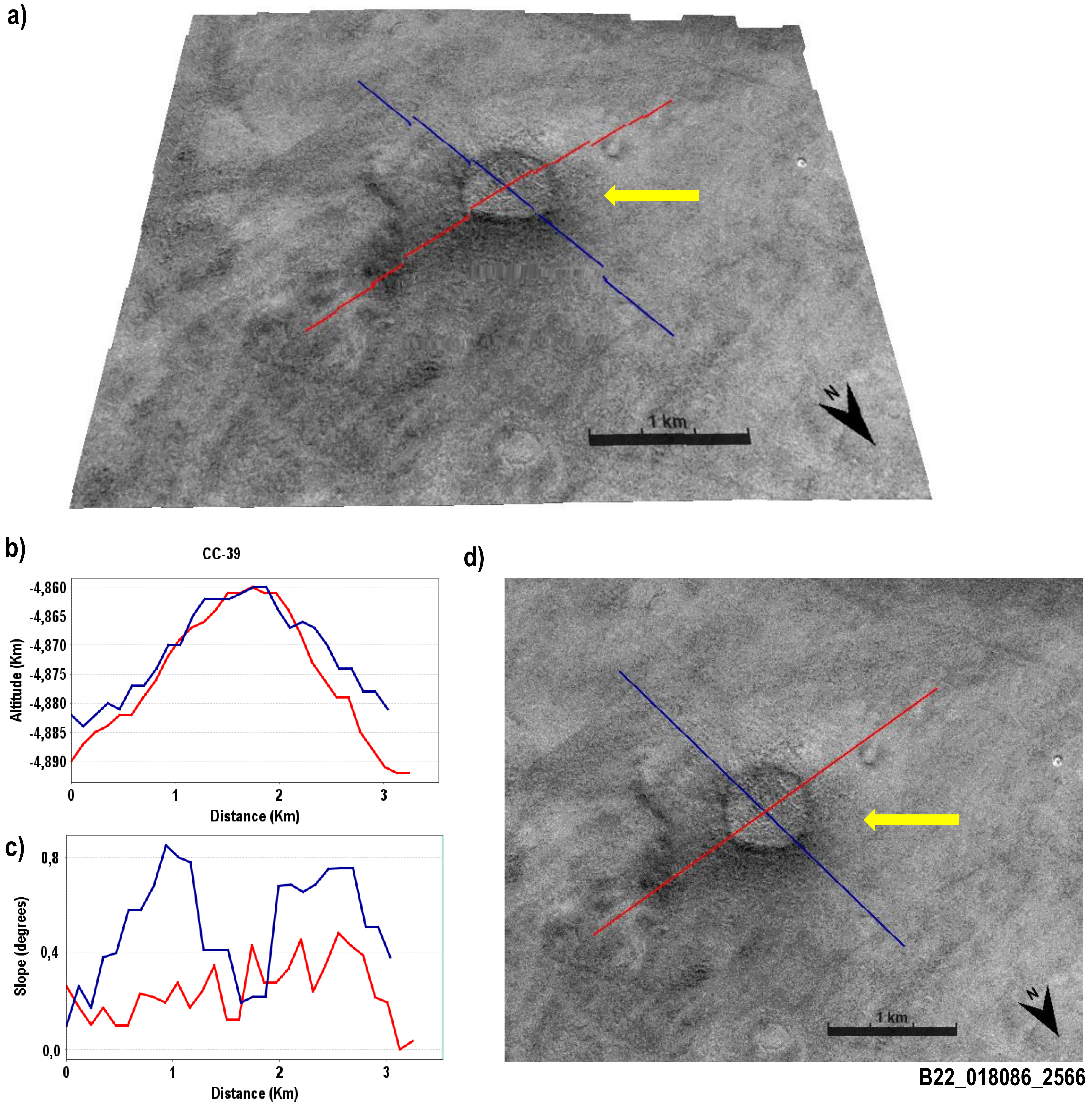
a)
1 km
N
b)
CC-39
Altitude (Km)
-4,860
-4,865
-4,870
-4,875
-4,880
-4,885
-4,890
Distance (Km)
0
1
2
3
c)
Slope (degrees)
0,8
0,4
0,0
Distance (Km)
d)
1 km
N
B22_018086_2566

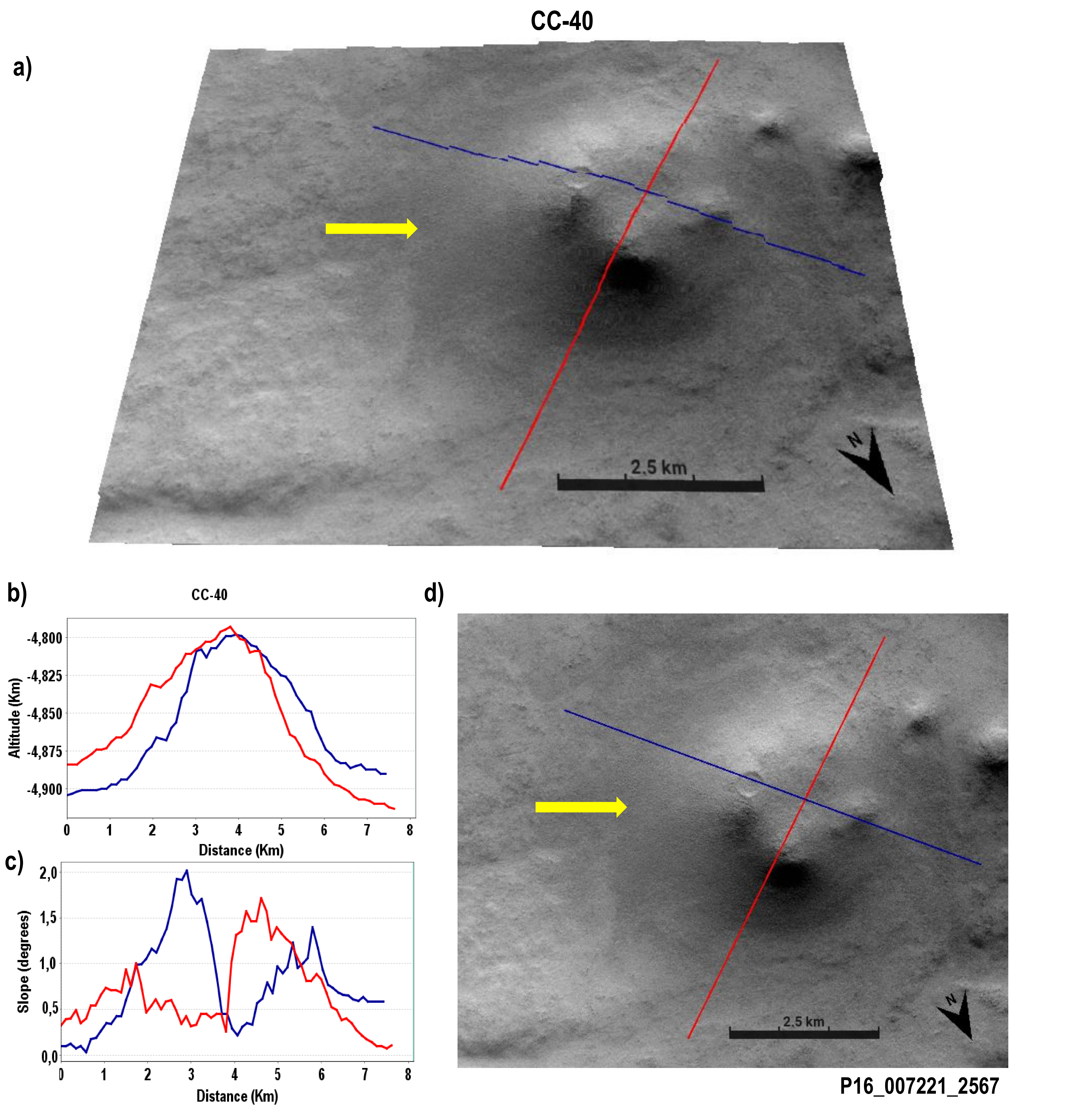
CC-40
a)
2,5 km
N
b)
CC-40
-4,800
-4,825
-4,850
-4,875
-4,900
Altitude (Km)
0 1 2 3 4 5 6 7 8
Distance (Km)
c)
2,0
1,5
1,0
0,5
0,0
Slope (degrees)
0 1 2 3 4 5 6 7 8
Distance (Km)
d)
2,5 km
N
P16_007221_2567

CC-41

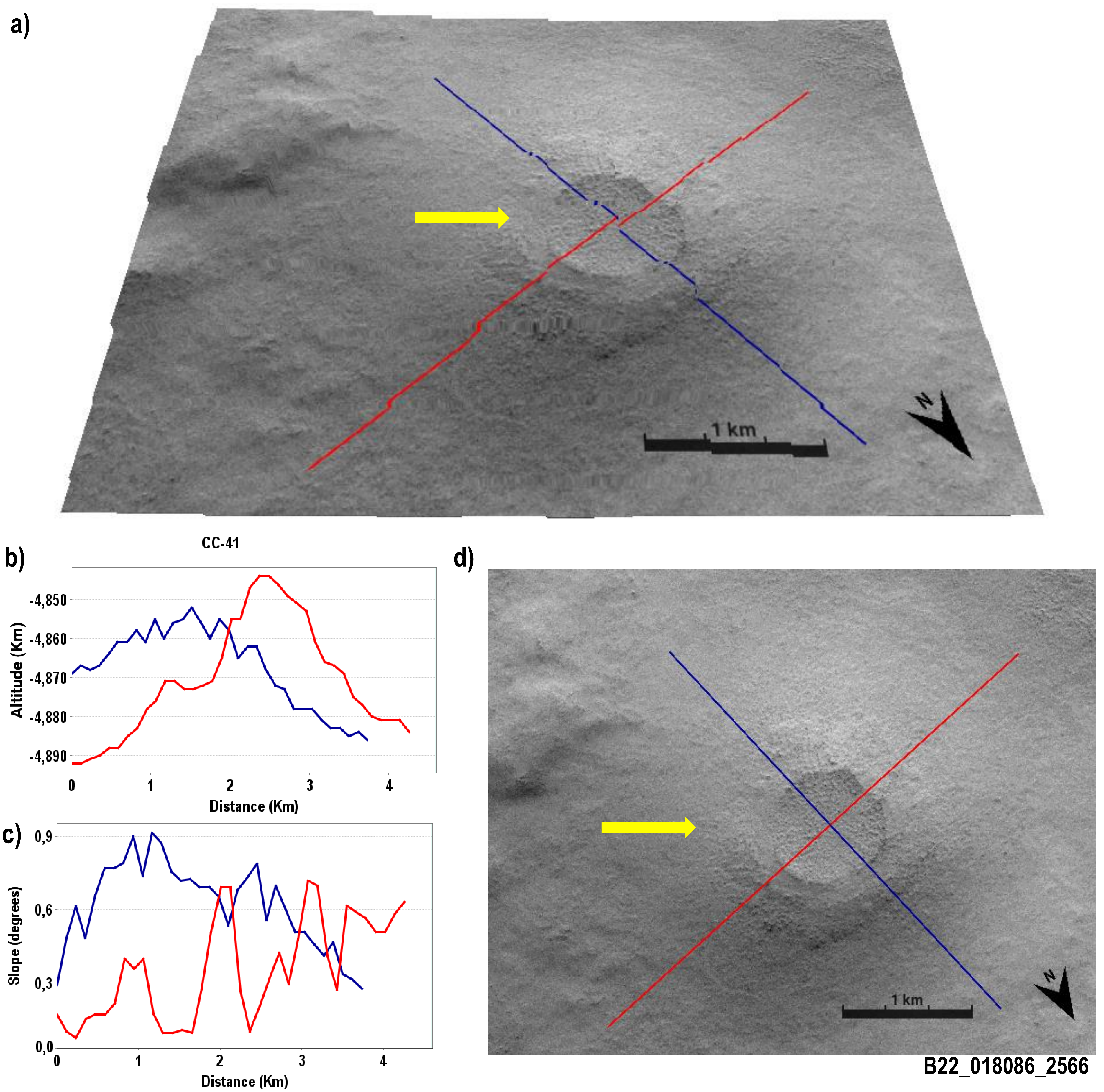
a)
1 km
N
b)
CC-41
Altitude (Km)
-4,850
-4,860
-4,870
-4,880
-4,890
Distance (Km)
0
1
2
3
4
c)
Slope (degrees)
0,9
0,6
0,3
0,0
Distance (Km)
d)
1 km
N
B22_018086_2566

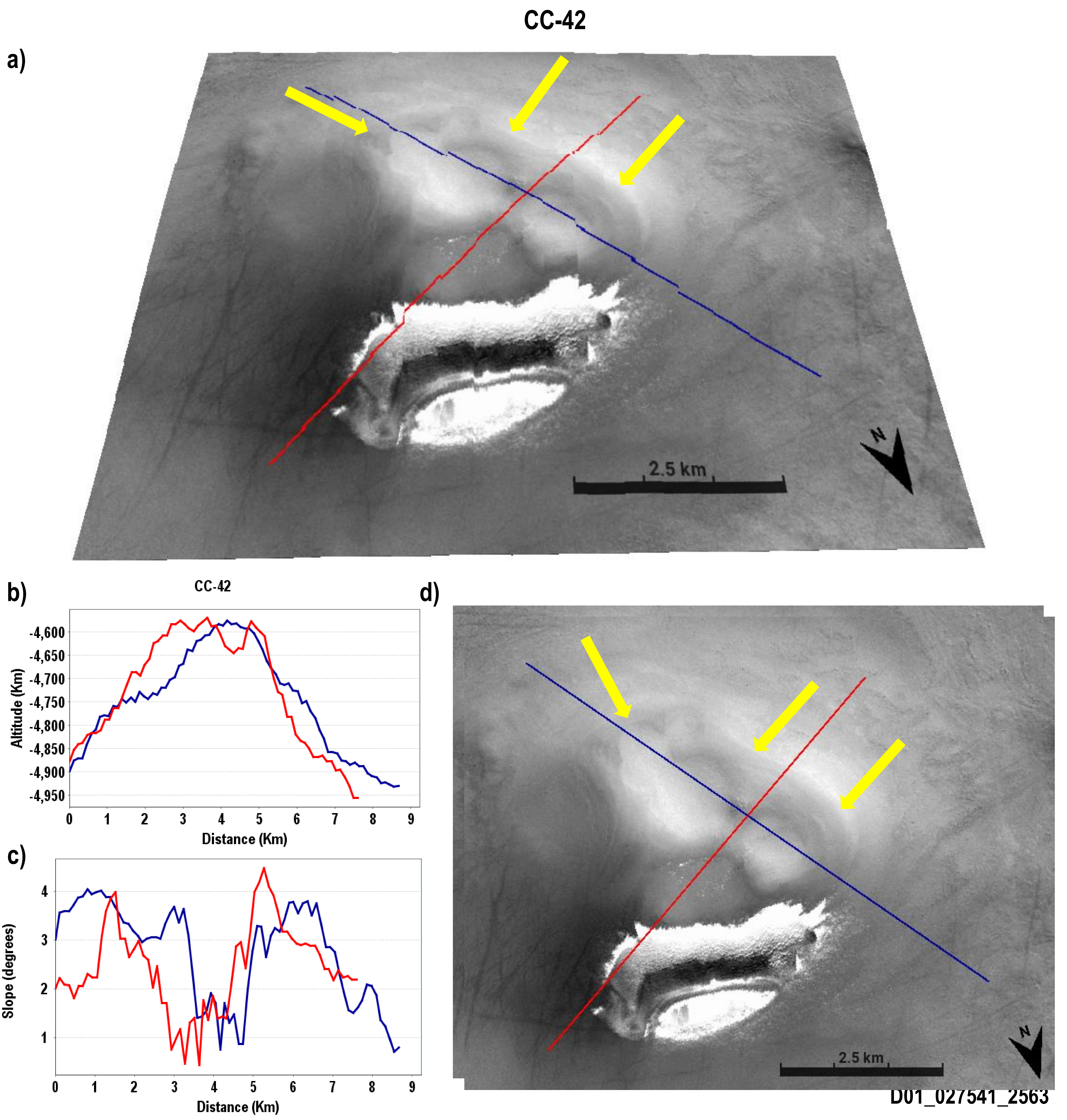
CC-42
a)
2.5 km
N
b)
CC-42
Altitude (Km)
Distance (Km)
c)
Slope (degrees)
Distance (Km)
d)
2.5 km
N
D01_027541_2563

CC-43

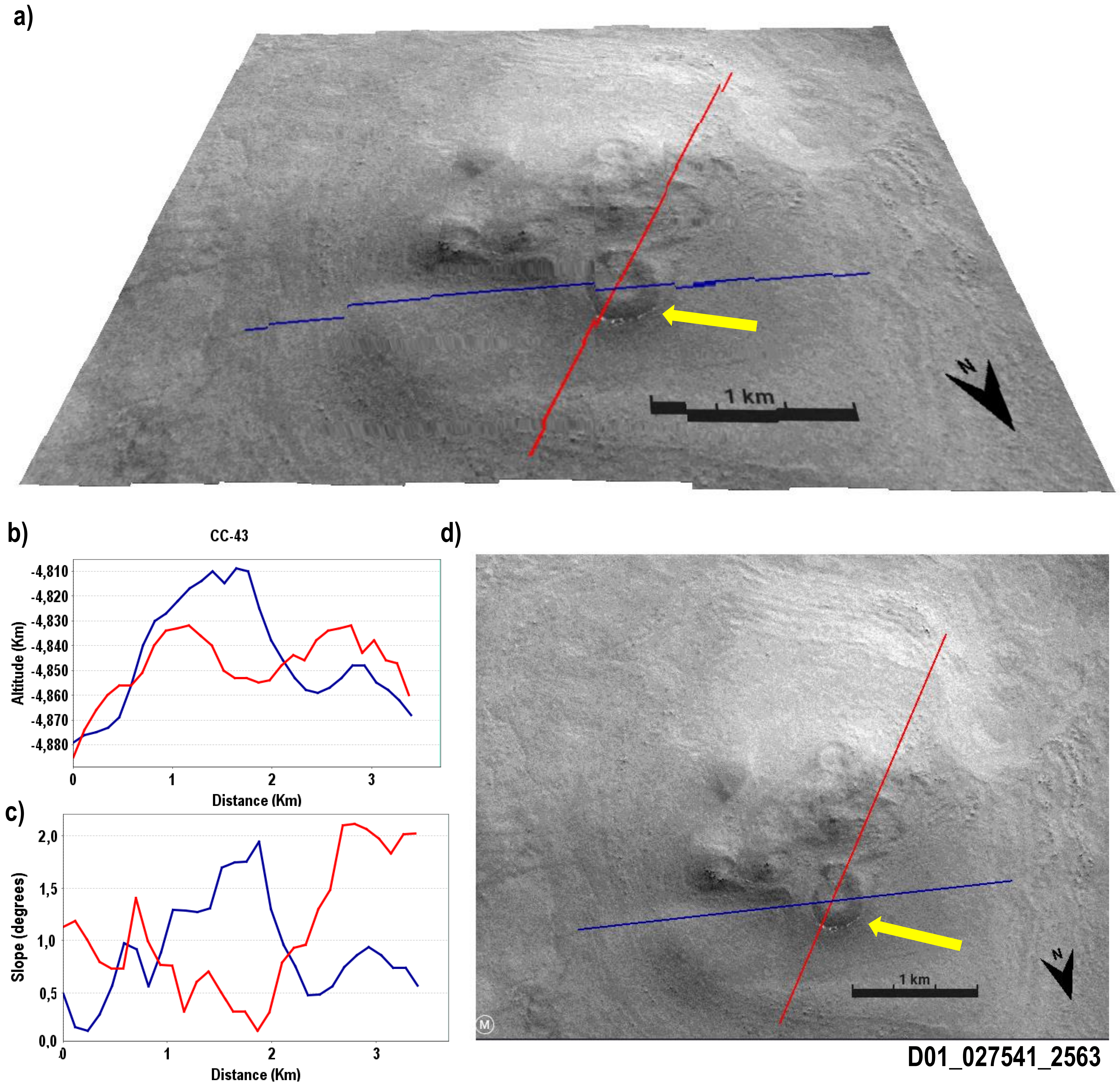
a)
1 km
N
b)
CC-43
Altitude (Km)
-4,810
-4,820
-4,830
-4,840
-4,850
-4,860
-4,870
-4,880
0
1
2
3
Distance (Km)
c)
Slope (degrees)
2,0
1,5
1,0
0,5
0,0
0
1
2
3
Distance (Km)
d)
1 km
N
D01_027541_2563

CC-44

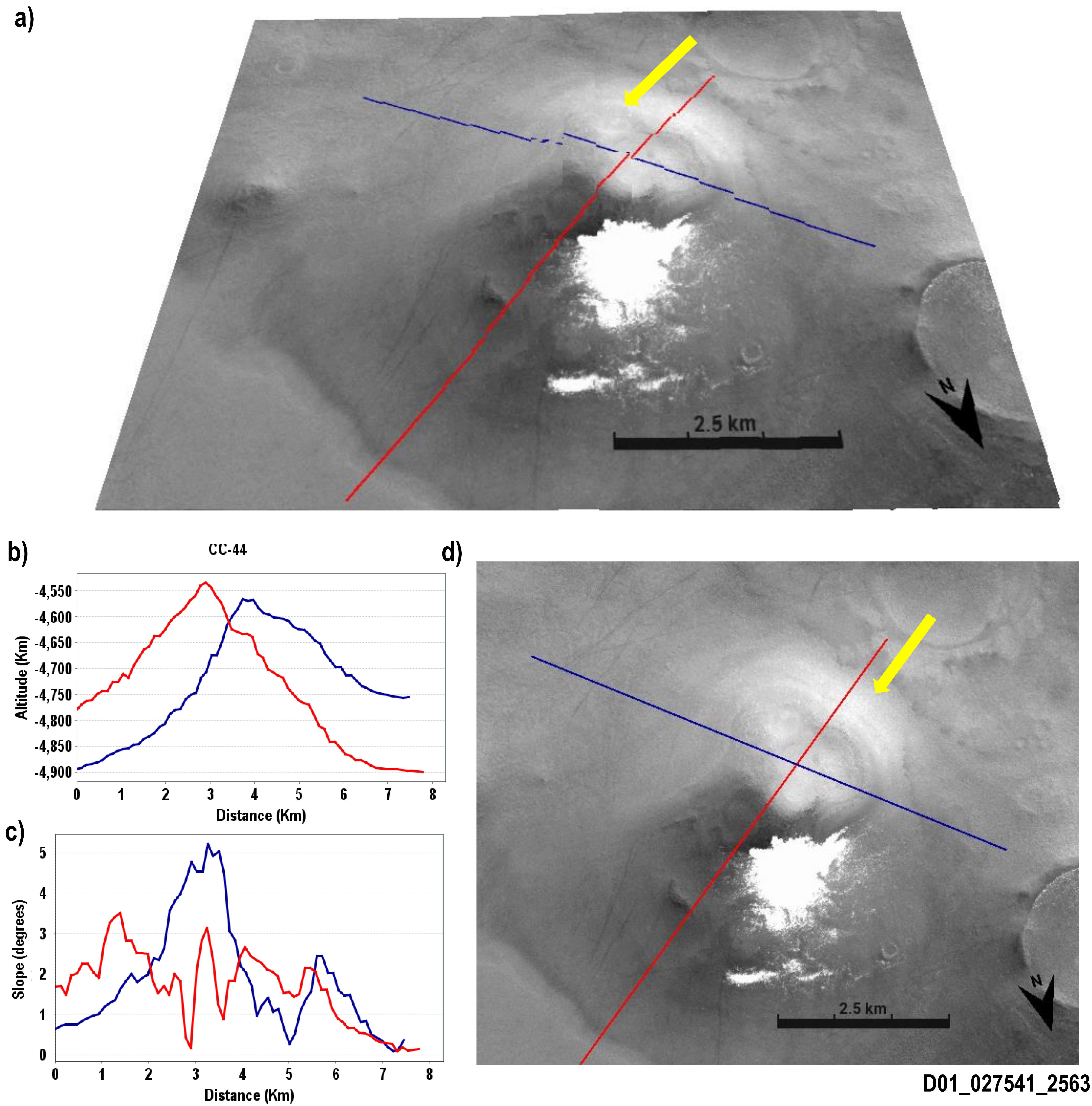

D01_027541_2563

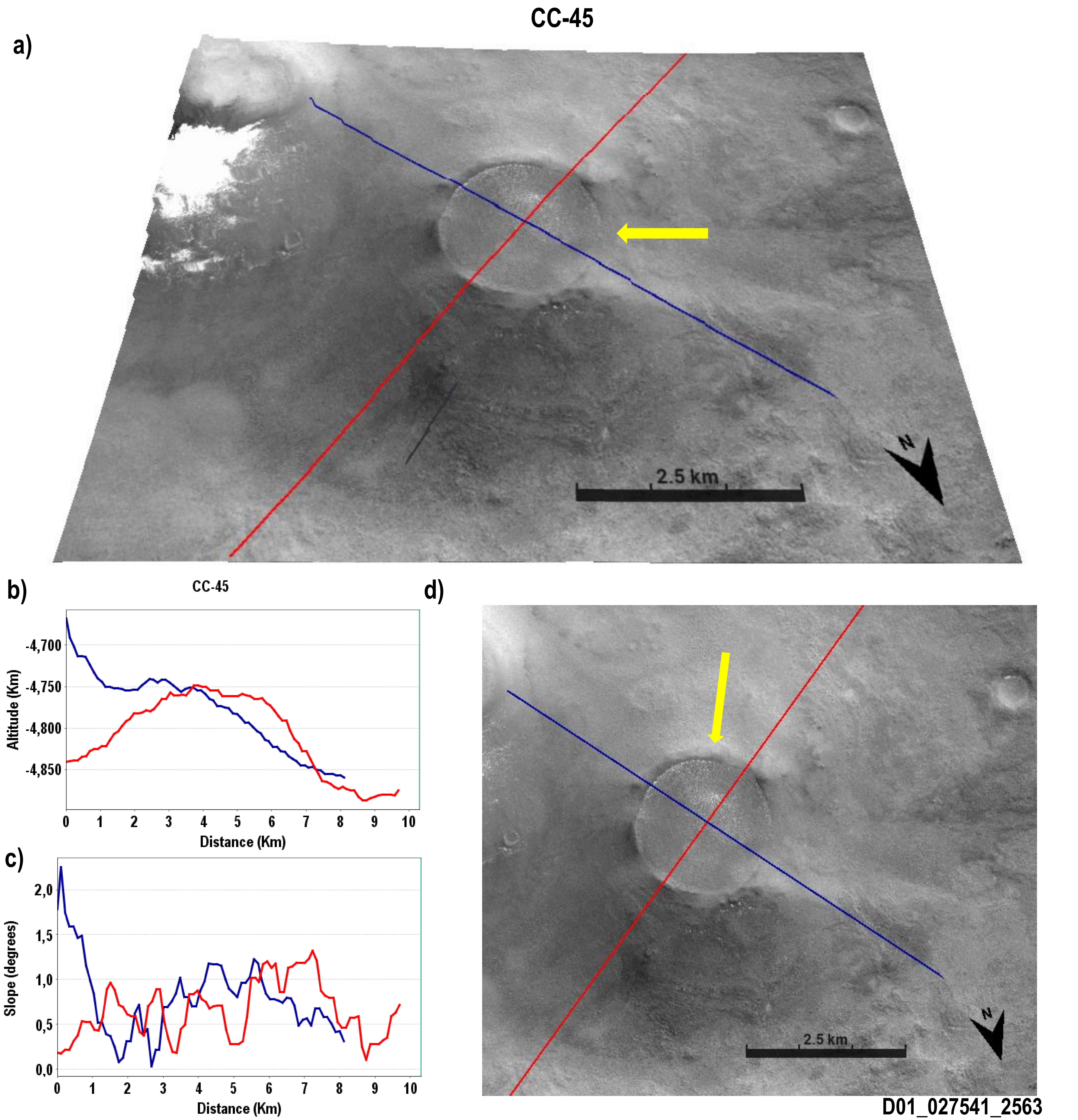

CC-45
a)
2.5 km
N
b)
CC-45
Altitude (Km)
-4,700
-4,750
-4,800
-4,850
0 1 2 3 4 5 6 7 8 9 10
Distance (Km)
c)
Slope (degrees)
2,0
1,5
1,0
0,5
0,0
0 1 2 3 4 5 6 7 8 9 10
Distance (Km)
d)
2.5 km
N
D01_027541_2563

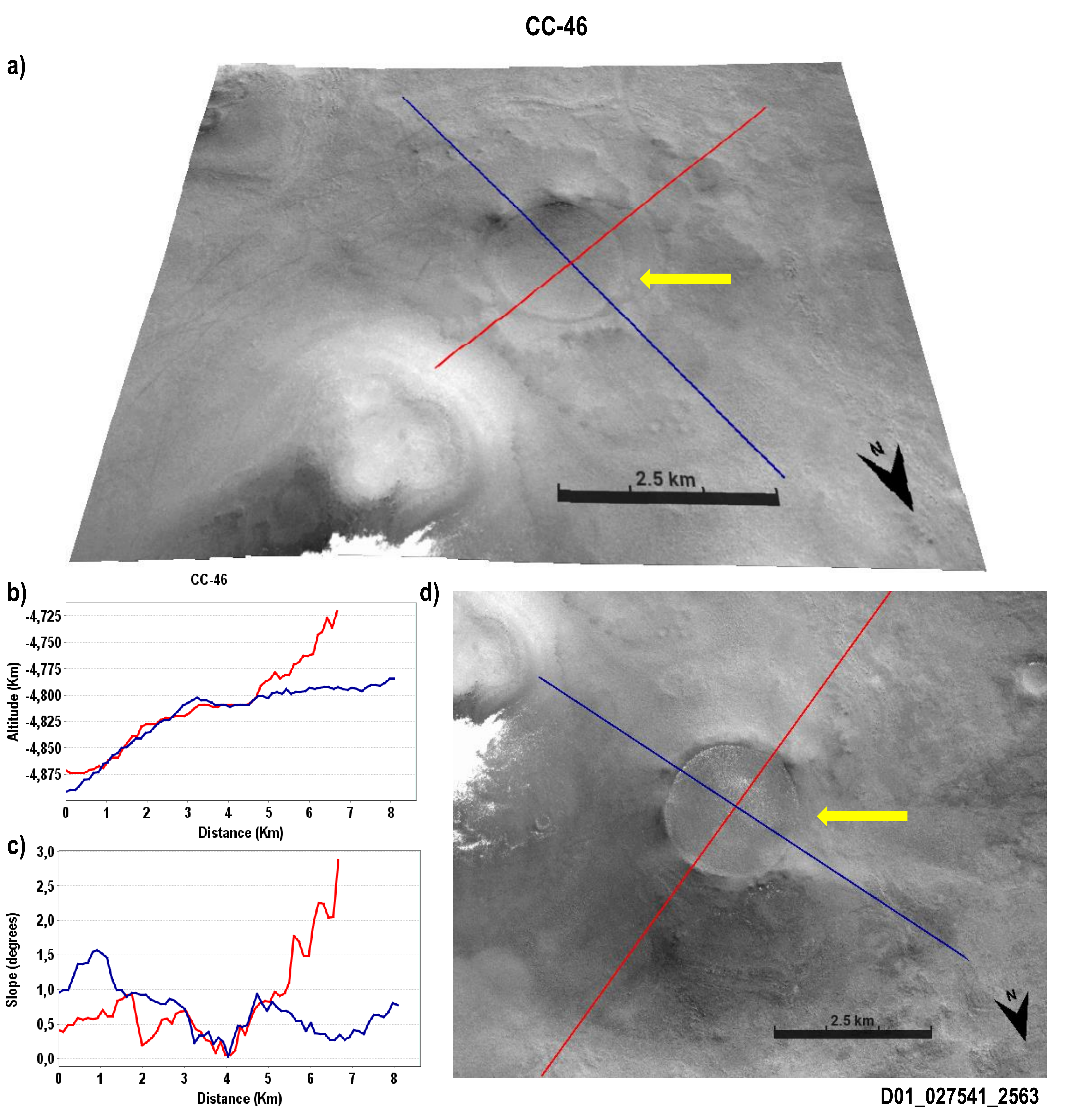
CC-46
a)
2.5 km
N
CC-46
b)
Altitude (Km)
-4,725
-4,750
-4,775
-4,800
-4,825
-4,850
-4,875
0 1 2 3 4 5 6 7 8
Distance (Km)
c)
Slope (degrees)
3,0
2,5
2,0
1,5
1,0
0,5
0,0
0 1 2 3 4 5 6 7 8
Distance (Km)
d)
2.5 km
N
D01_027541_2563

CC-47

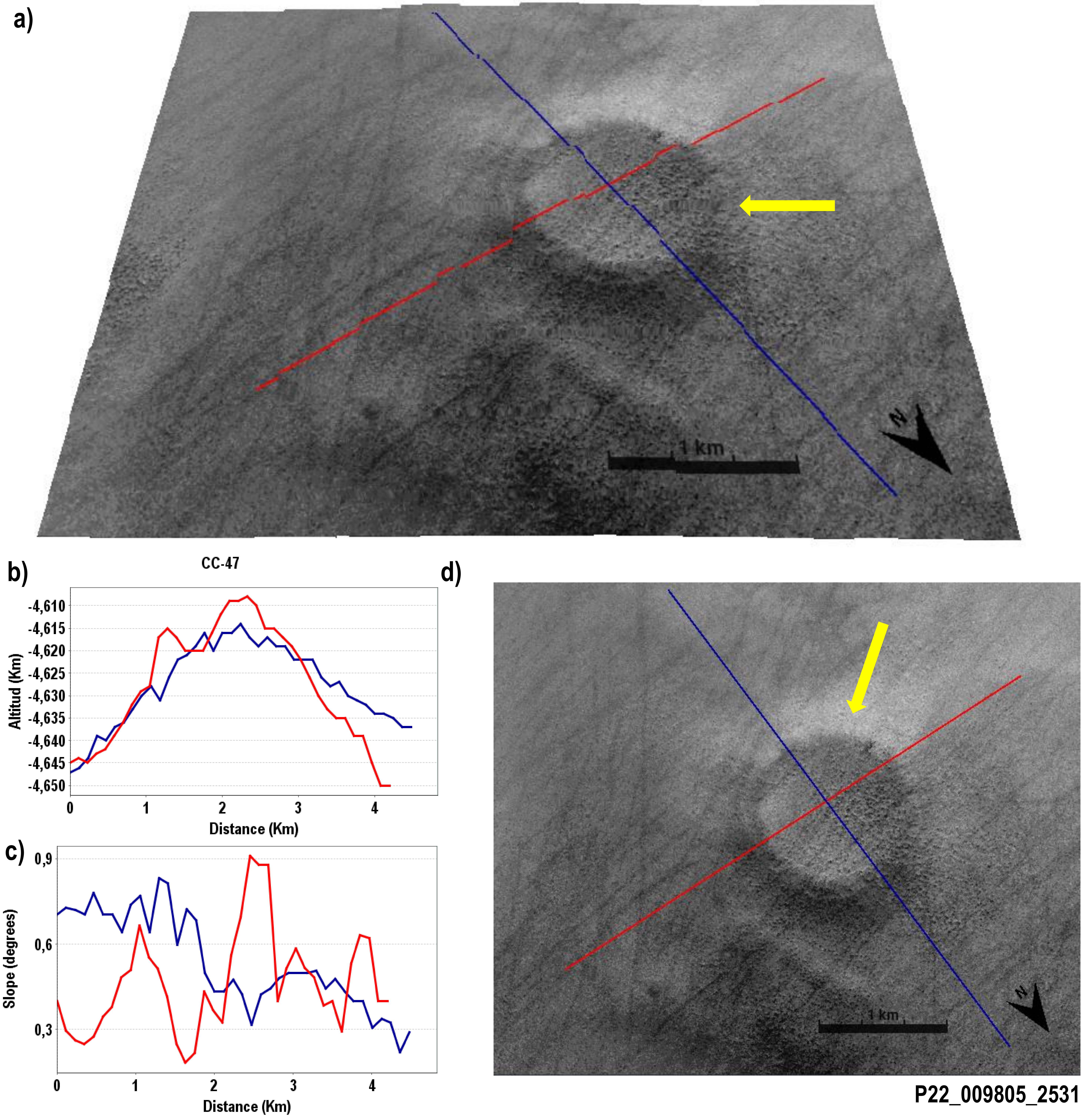

a)
1 km
N
b)
CC-47
-4,610
-4,615
-4,620
-4,625
-4,630
-4,635
-4,640
-4,645
-4,650
Altitud (Km)
0
1
2
3
4
Distance (Km)
c)
0,9
0,6
0,3
Slope (degrees)
0
1
2
3
4
Distance (Km)
d)
1 km
N
P22_009805_2531

CC-48

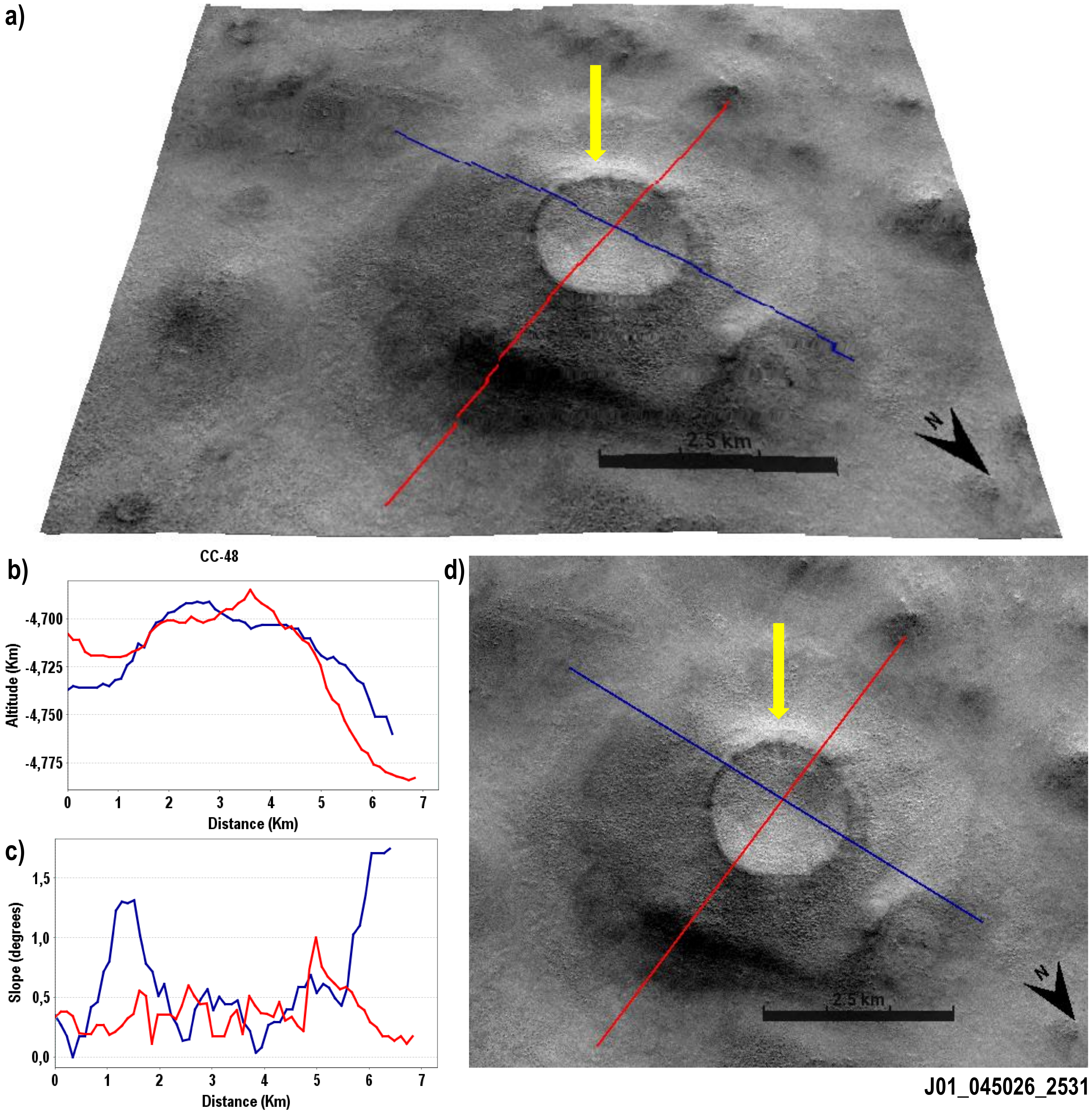

J01_045026_2531

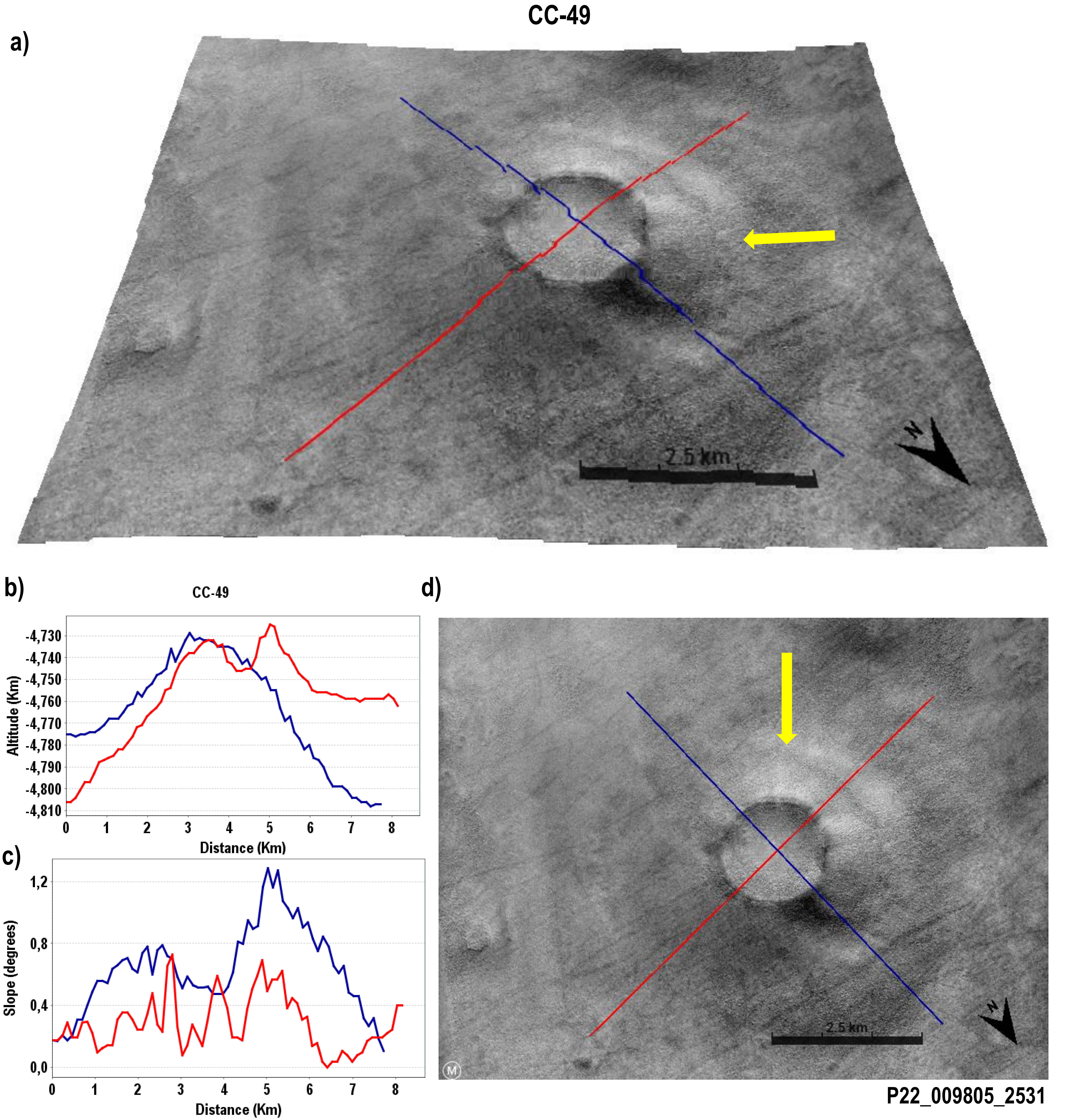
CC-49
a)
2.5 km
N
b)
CC-49
Altitude (Km)
-4,730
-4,740
-4,750
-4,760
-4,770
-4,780
-4,790
-4,800
-4,810
0 1 2 3 4 5 6 7 8
Distance (Km)
c)
Slope (degrees)
1,2
0,8
0,4
0,0
0 1 2 3 4 5 6 7 8
Distance (Km)
d)
2.5 km
N
P22_009805_2531

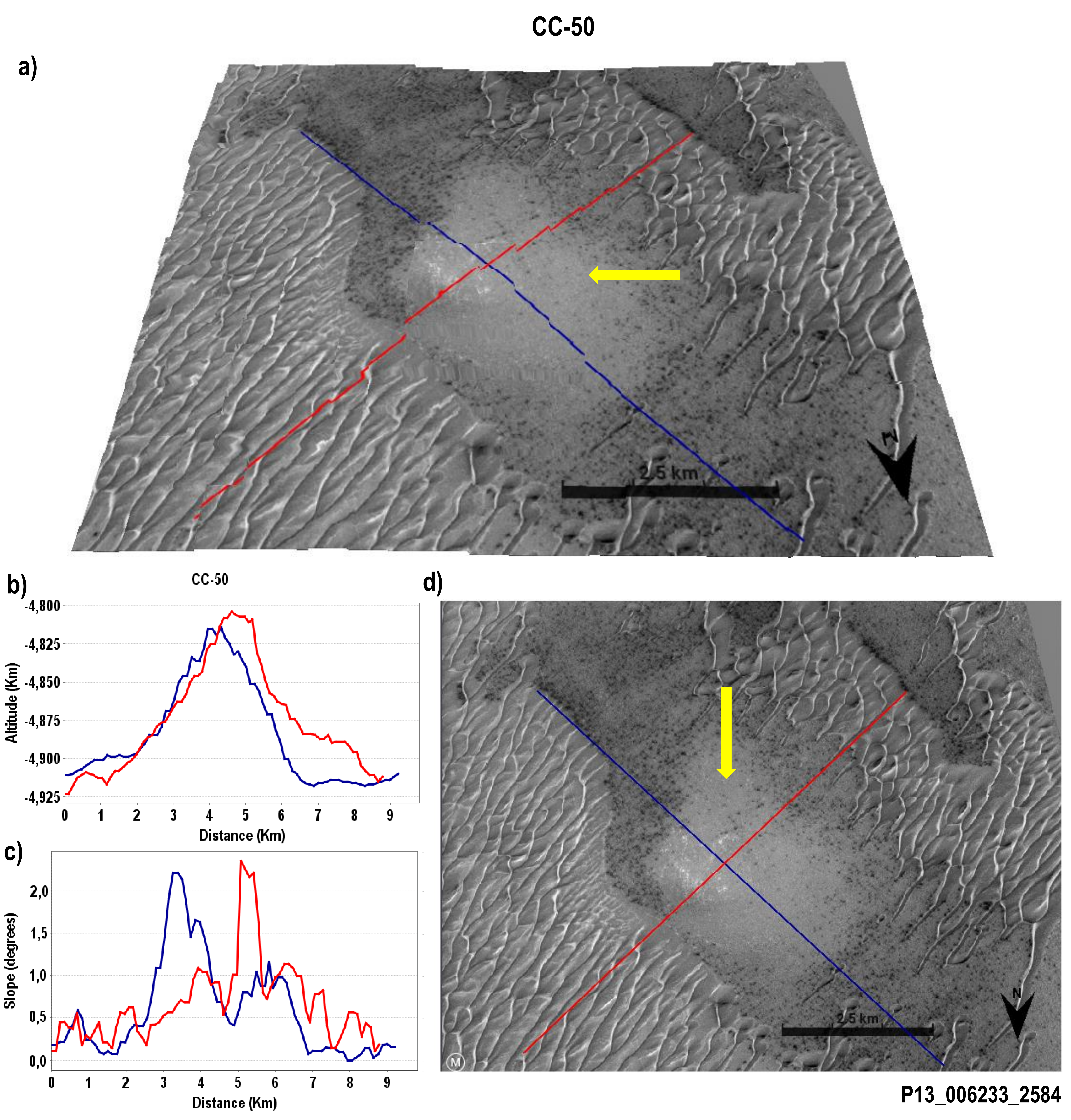
CC-50
a)
2.5 km
N
b)
CC-50
Altitude (Km)
-4,800
-4,825
-4,850
-4,875
-4,900
-4,925
0 1 2 3 4 5 6 7 8 9
Distance (Km)
c)
Slope (degrees)
2,0
1,5
1,0
0,5
0,0
0 1 2 3 4 5 6 7 8 9
Distance (Km)
d)
2.5 km
N
P13_006233_2584

CC-51

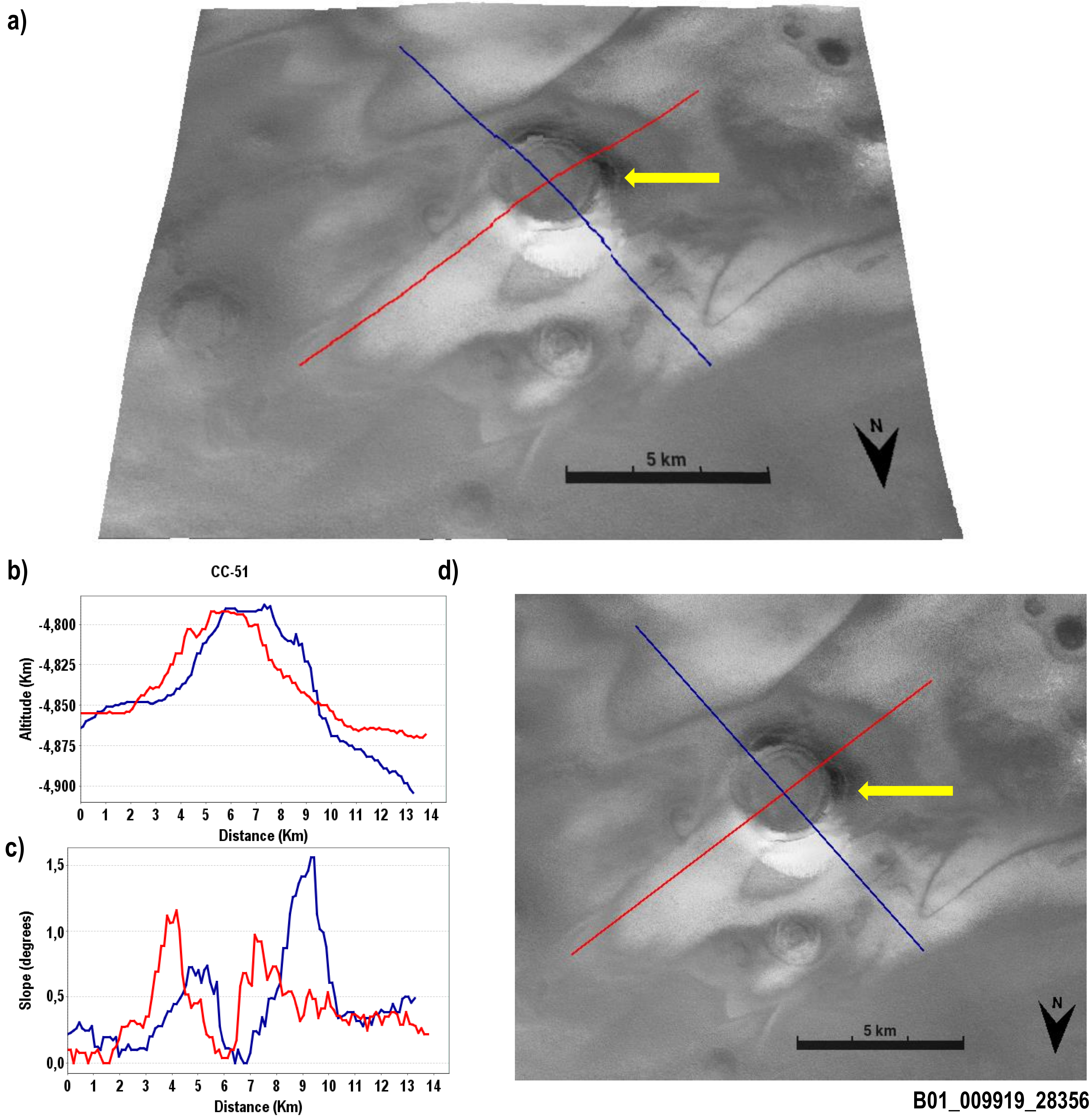

B01_009919_28356

CC-52

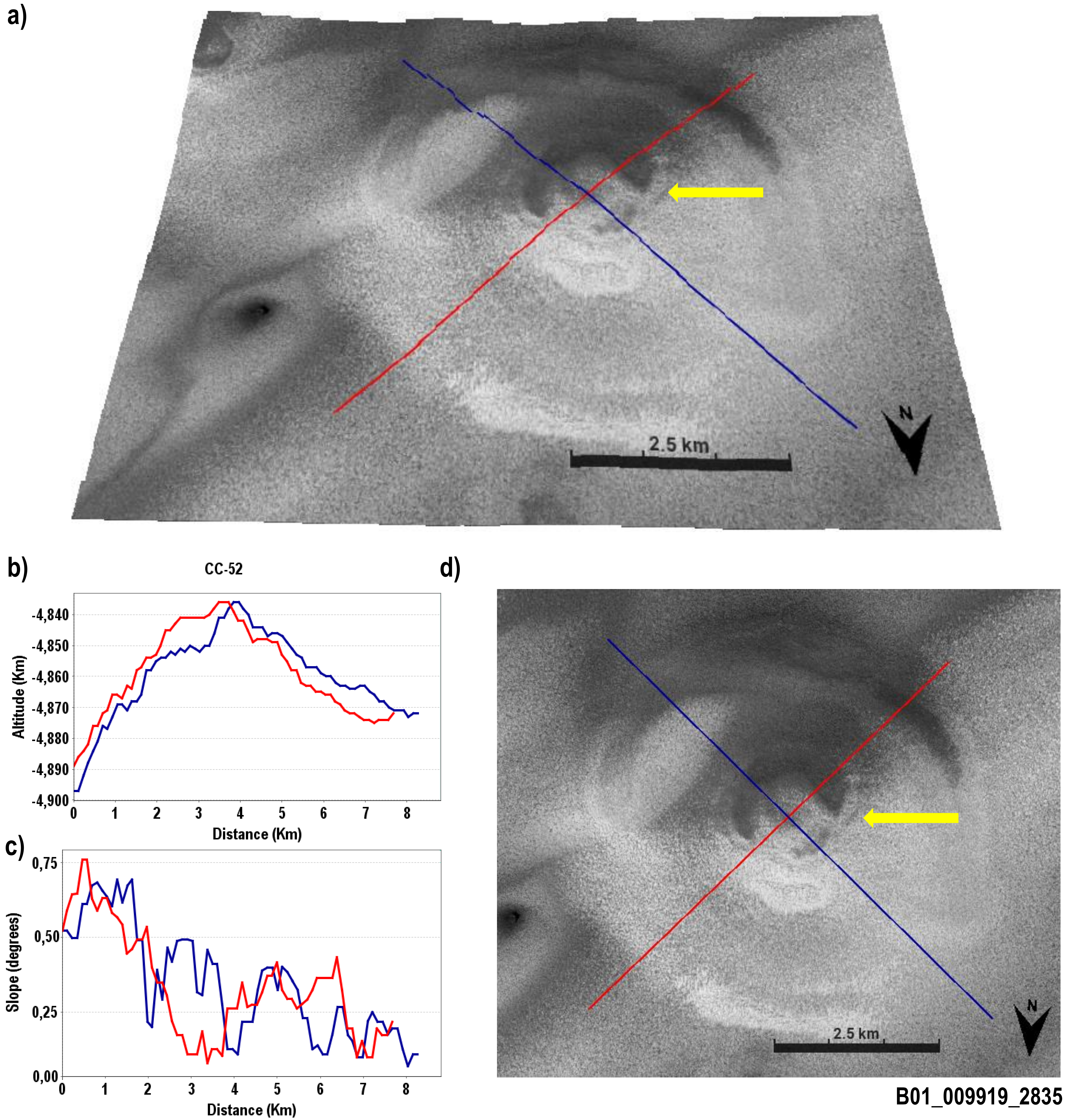

a)
2.5 km
N
b)
CC-52
Altitude (Km)
-4,840
-4,850
-4,860
-4,870
-4,880
-4,890
-4,900
0 1 2 3 4 5 6 7 8
Distance (Km)
c)
Slope (degrees)
0,75
0,50
0,25
0,00
0 1 2 3 4 5 6 7 8
Distance (Km)
d)
2.5 km
N
B01_009919_2835

CC-53

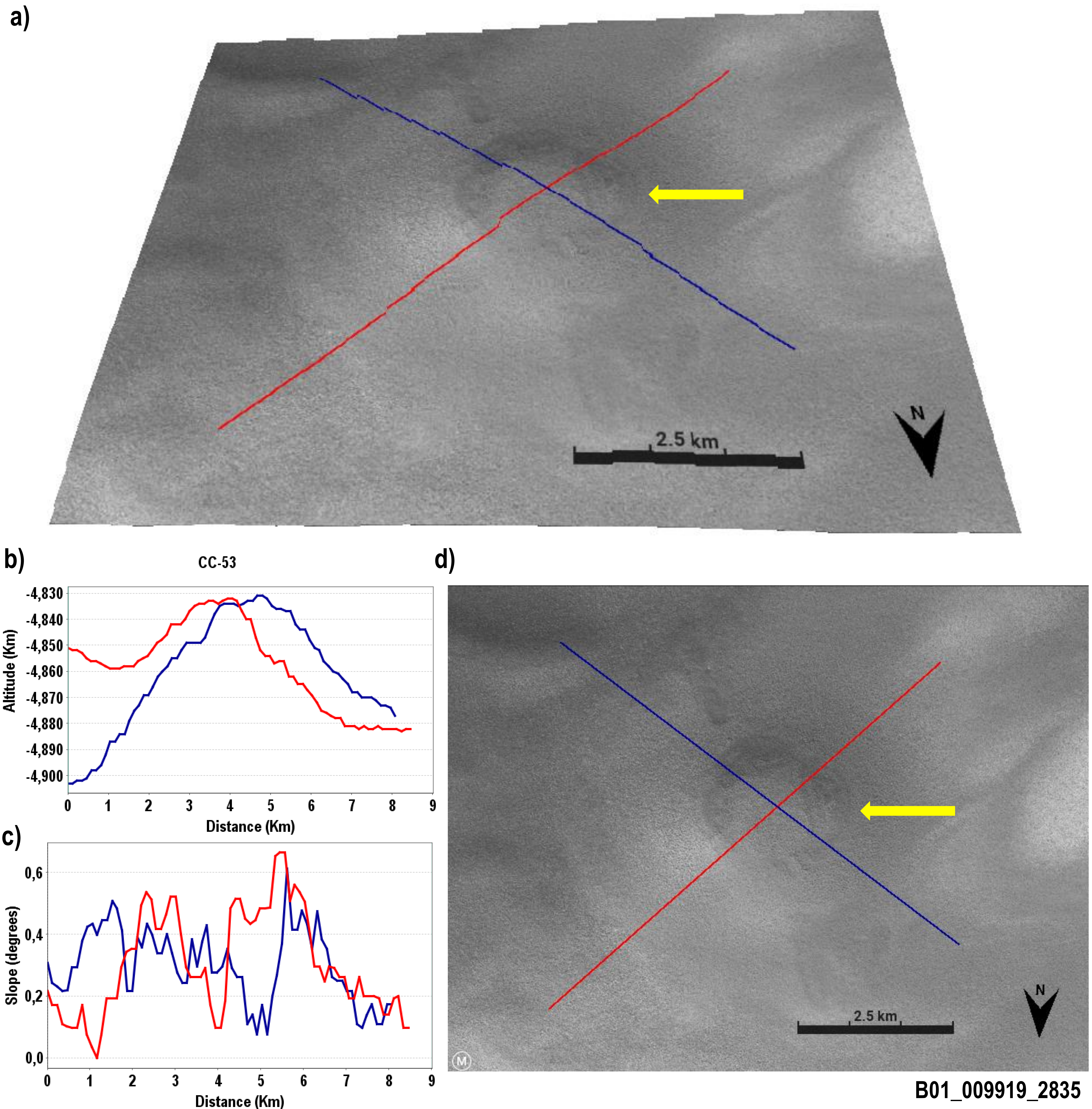

a)
2.5 km
N
b)
CC-53
-4,830
-4,840
-4,850
-4,860
-4,870
-4,880
-4,890
-4,900
Altitude (Km)
0 1 2 3 4 5 6 7 8 9
Distance (Km)
c)
0,6
0,4
0,2
0,0
Slope (degrees)
0 1 2 3 4 5 6 7 8 9
Distance (Km)
d)
2.5 km
N
B01_009919_2835

CC-54

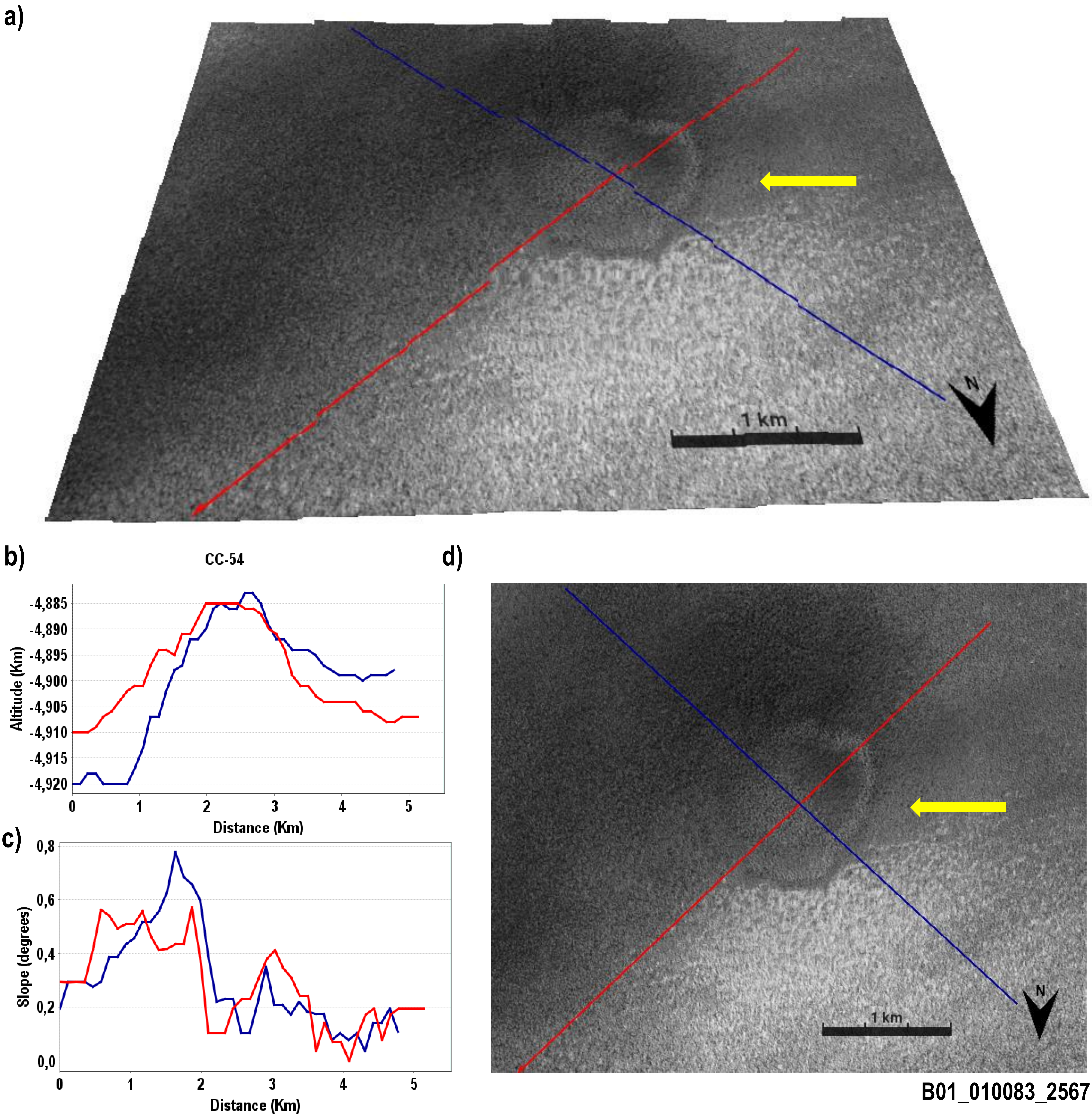

B01_010083_2567

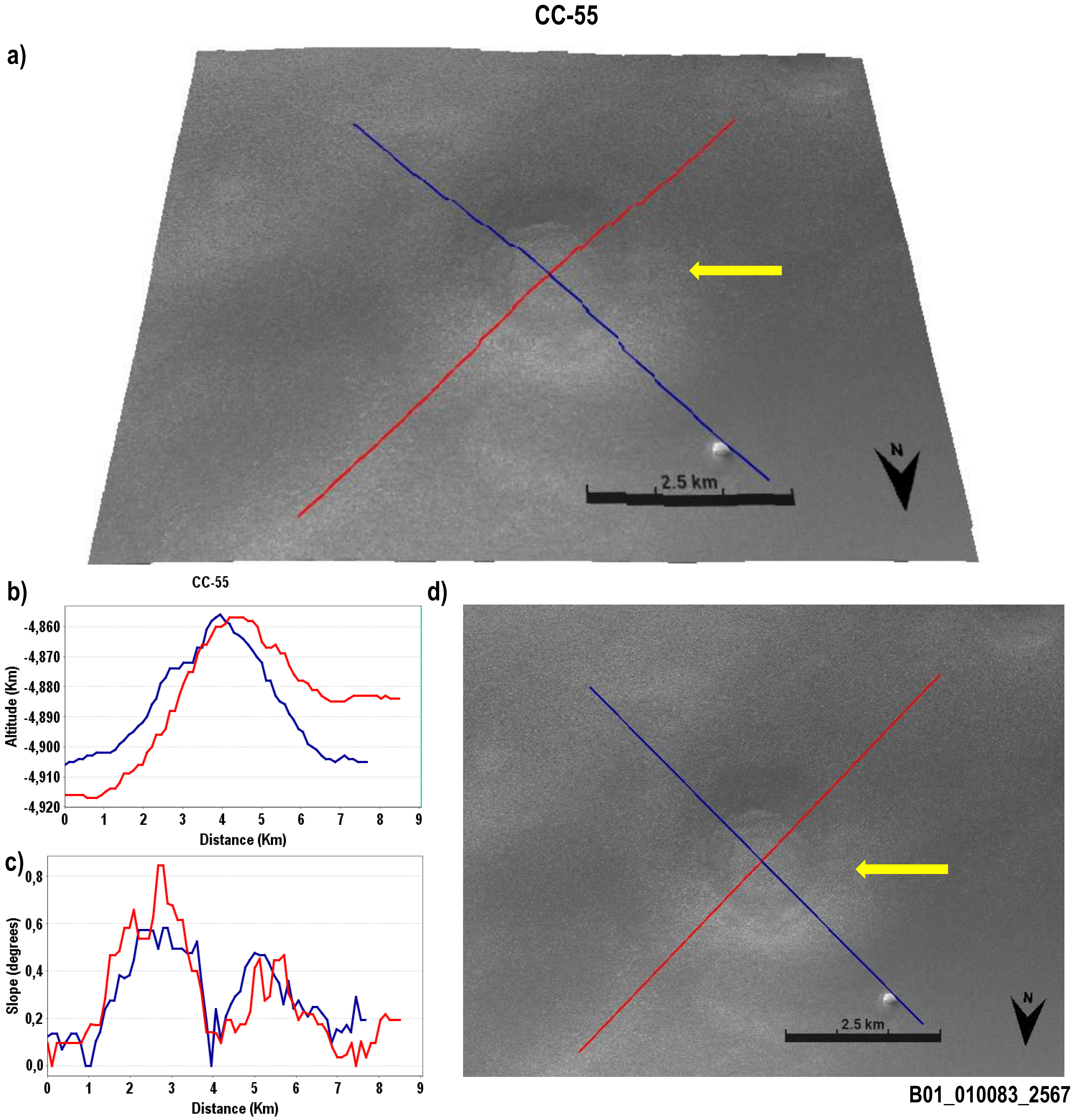
CC-55
a)
2.5 km
N
b)
CC-55
Altitude (Km)
-4,860
-4,870
-4,880
-4,890
-4,900
-4,910
-4,920
Distance (Km)
c)
Slope (degrees)
0,8
0,6
0,4
0,2
0,0
Distance (Km)
d)
2.5 km
N
B01_010083_2567

CC-56

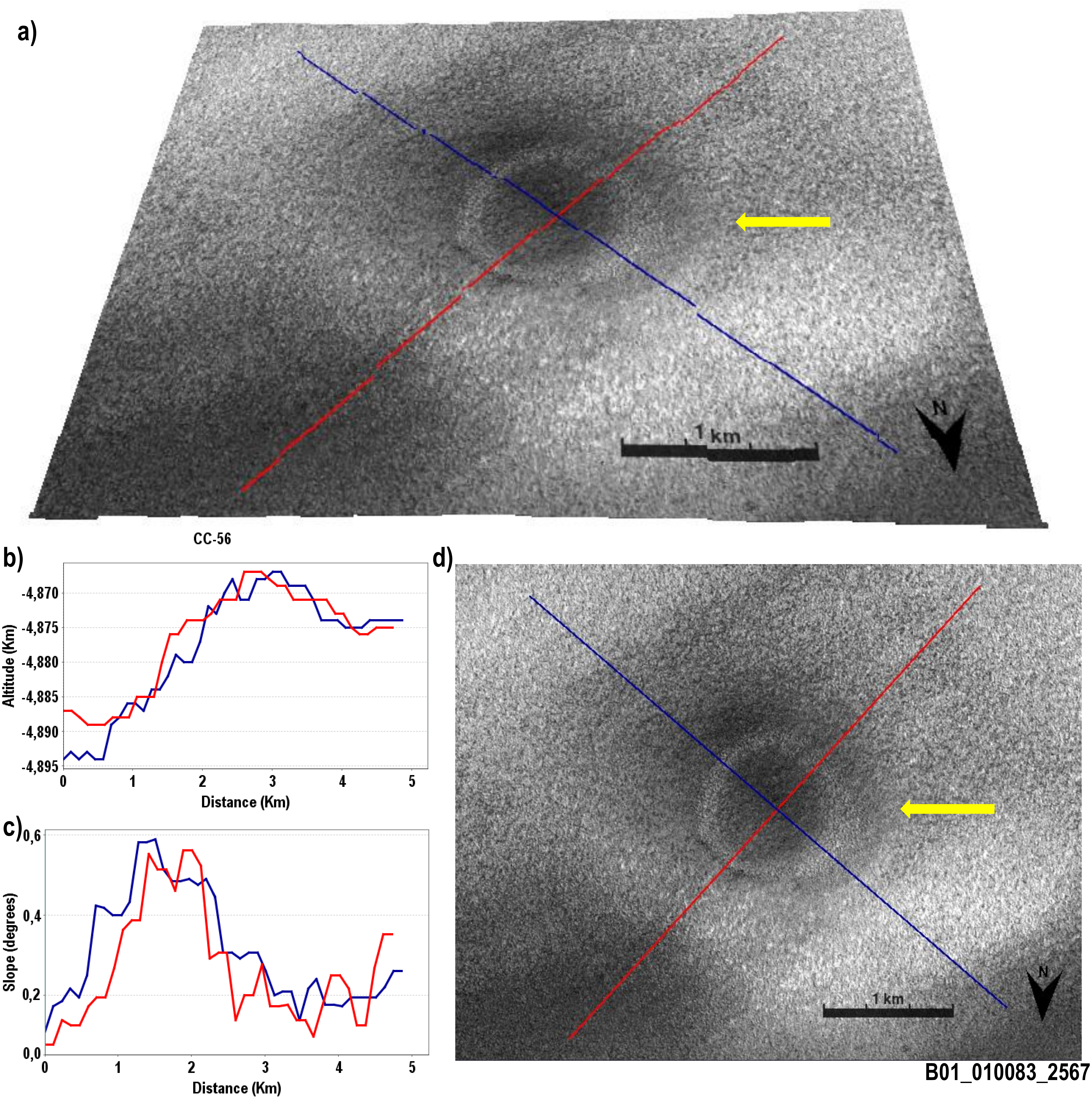

B01_010083_2567

CC-57

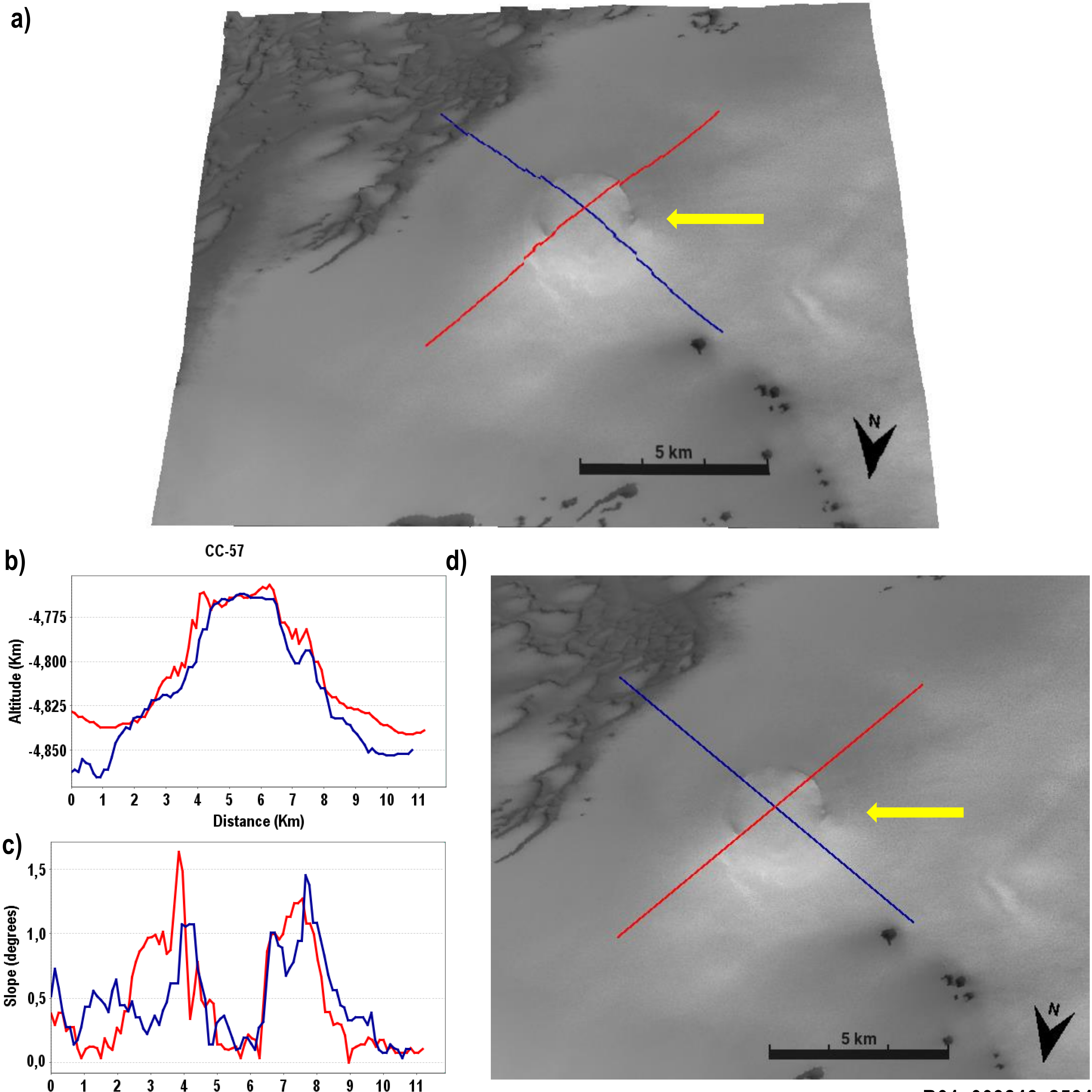

B01_009846_2564

CC-58

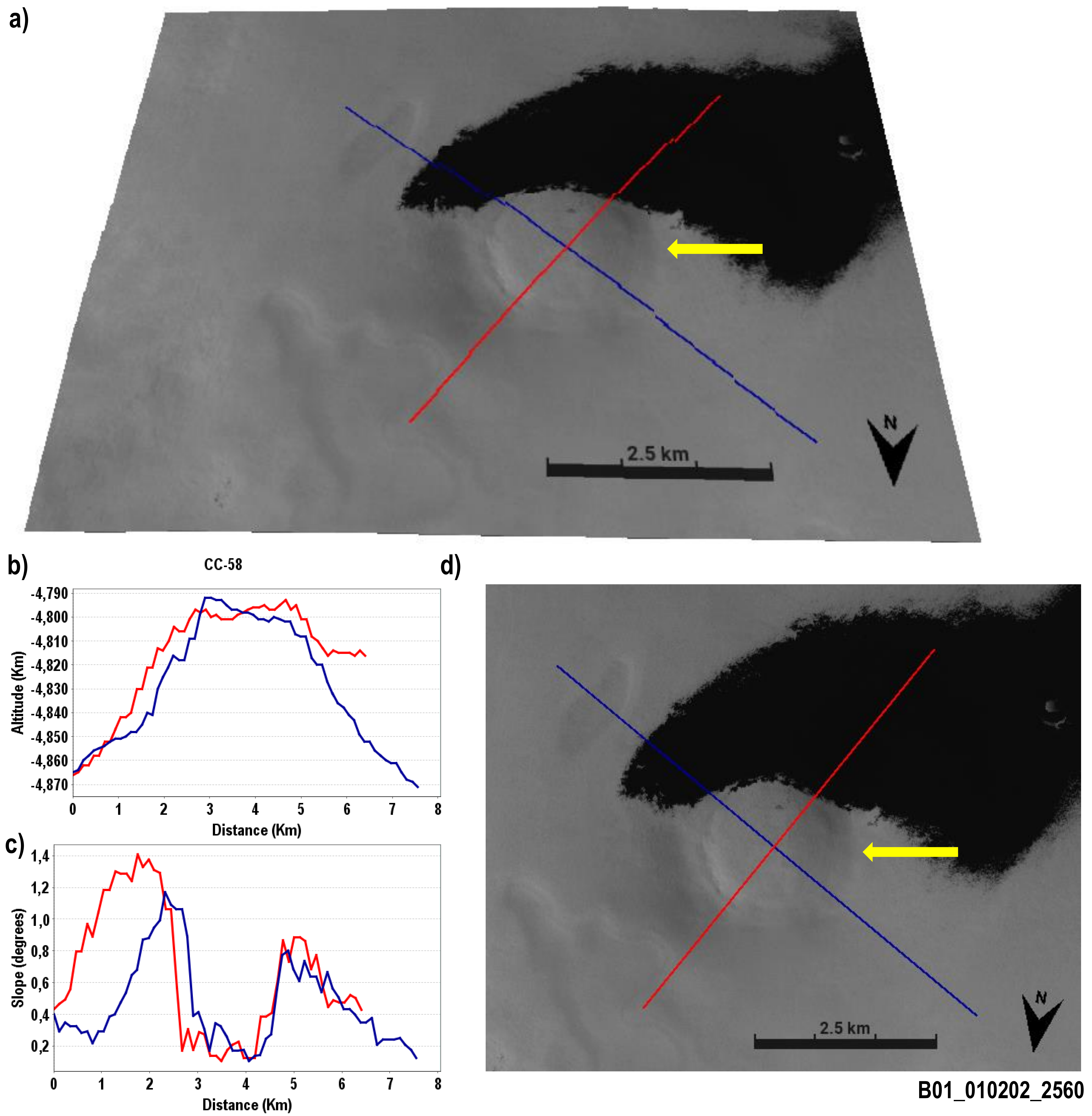
a)
2.5 km
N
b)
CC-58
Altitude (Km)
-4,790
-4,800
-4,810
-4,820
-4,830
-4,840
-4,850
-4,860
-4,870
0 1 2 3 4 5 6 7 8
Distance (Km)
c)
Slope (degrees)
1,4
1,2
1,0
0,8
0,6
0,4
0,2
0 1 2 3 4 5 6 7 8
Distance (Km)
d)
2.5 km
N
B01_010202_2560

CC-59

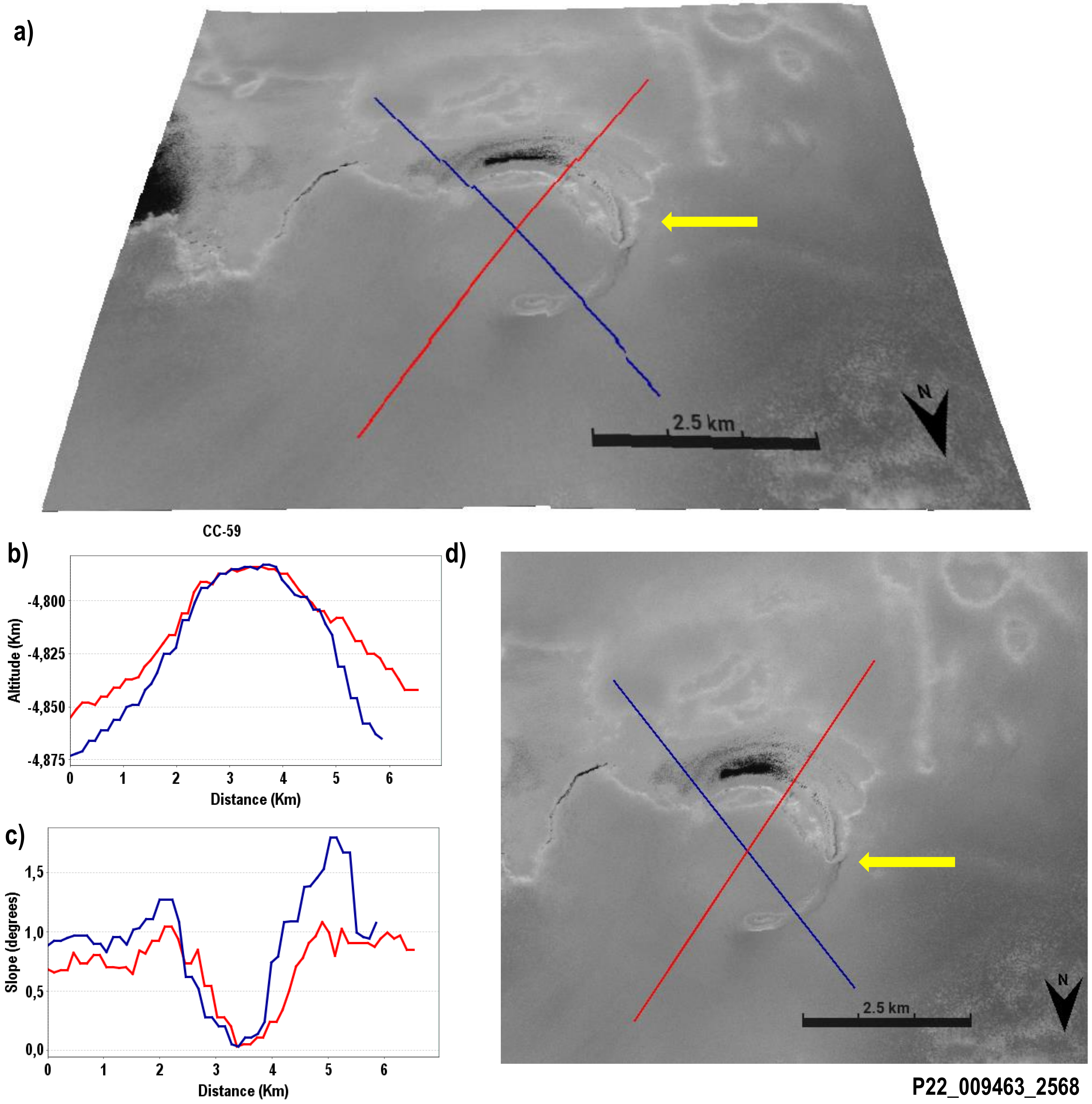

a)
2.5 km
N
CC-59
b)
Altitude (Km)
-4,800
-4,825
-4,850
-4,875
0 1 2 3 4 5 6
Distance (Km)
c)
Slope (degrees)
1,5
1,0
0,5
0,0
0 1 2 3 4 5 6
Distance (Km)
d)
2.5 km
N
P22_009463_2568

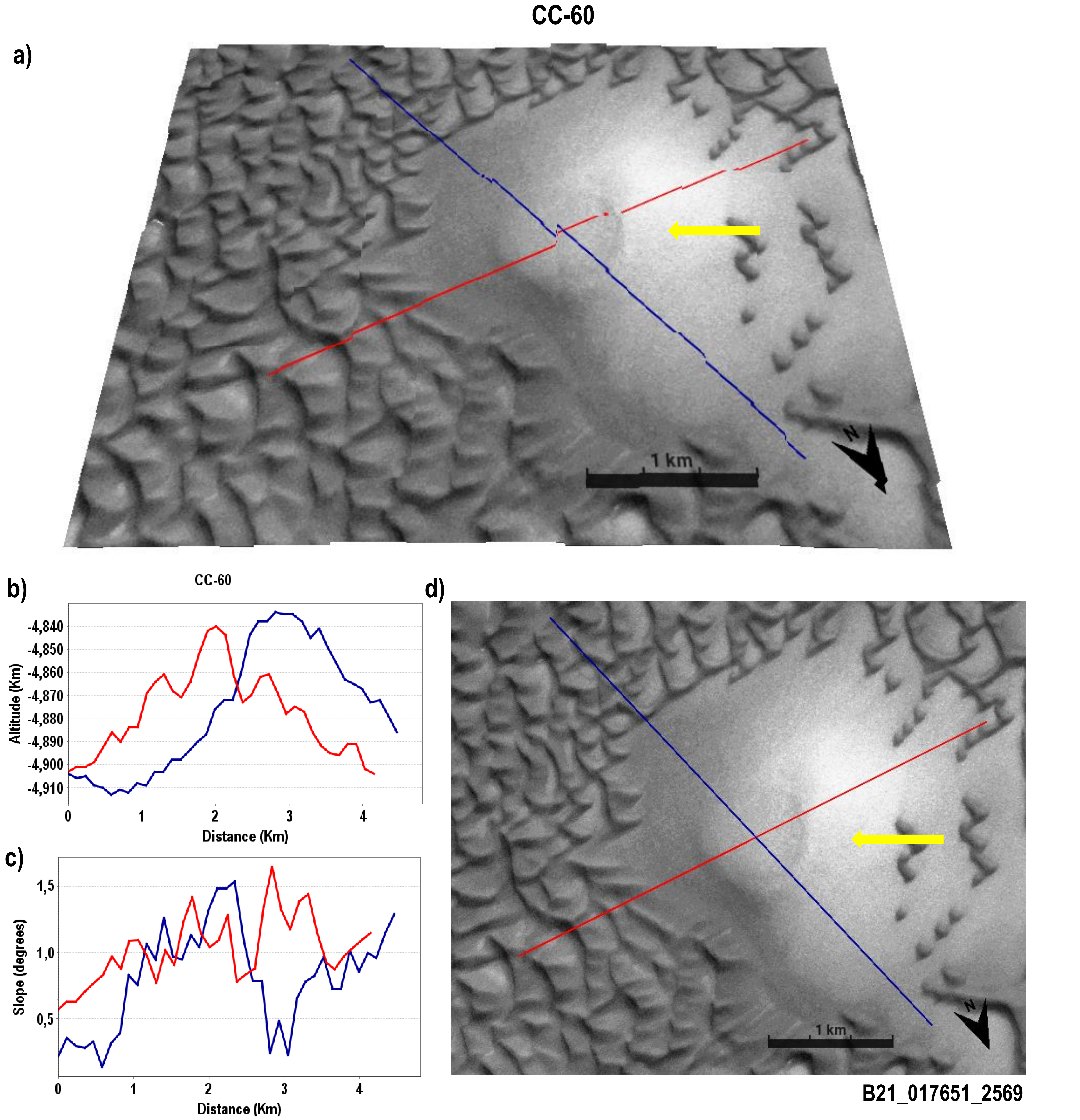
CC-60
a)
1 km
N
b)
CC-60
Altitude (Km)
-4,840
-4,850
-4,860
-4,870
-4,880
-4,890
-4,900
-4,910
0
1
2
3
4
Distance (Km)
c)
Slope (degrees)
1,5
1,0
0,5
0
1
2
3
4
Distance (Km)
d)
1 km
N
B21_017651_2569

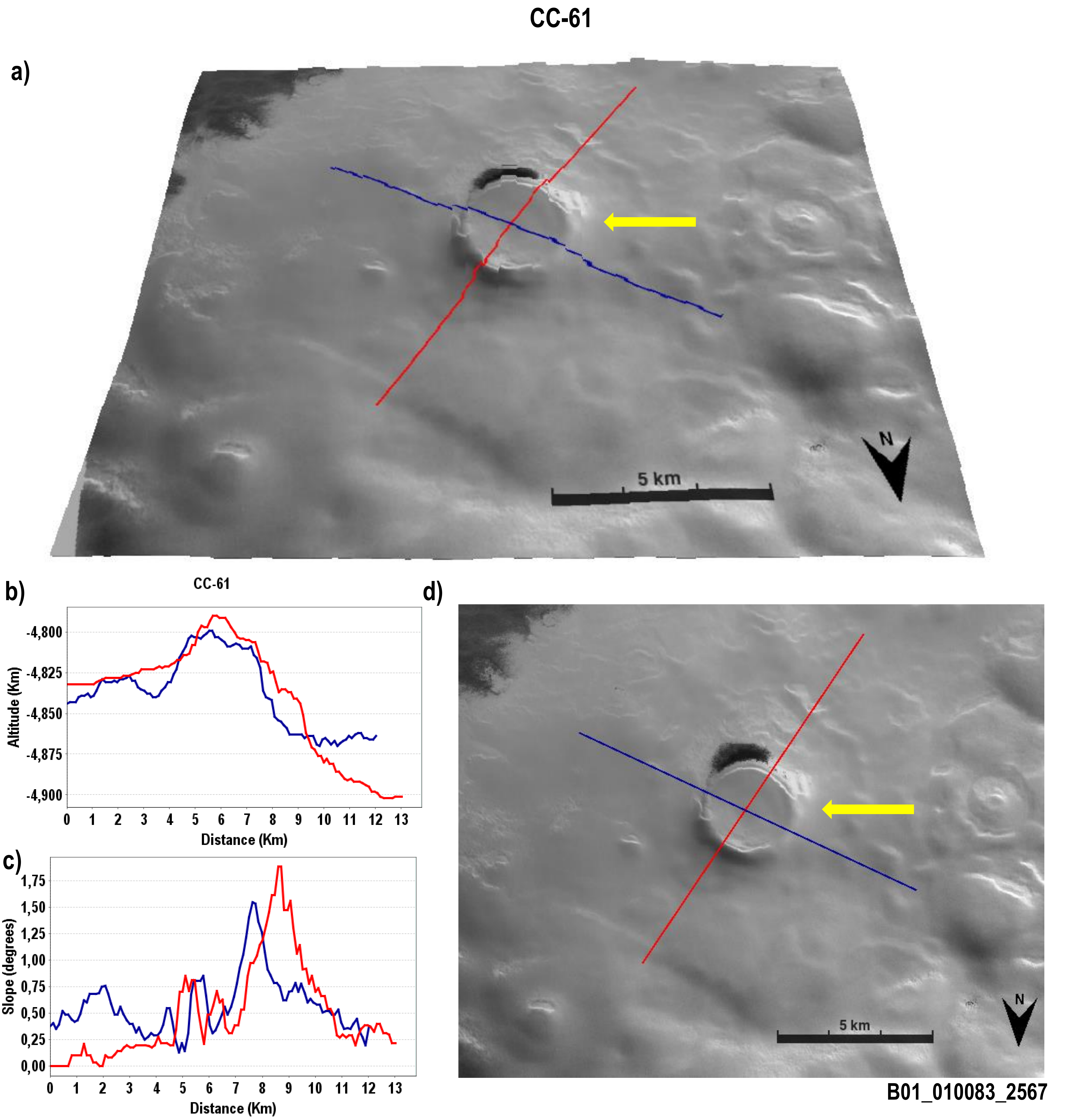

CC-61
a)
5 km
N
b)
CC-61
Altitude (Km)
-4,800
-4,825
-4,850
-4,875
-4,900
0 1 2 3 4 5 6 7 8 9 10 11 12 13
Distance (Km)
c)
Slope (degrees)
1,75
1,50
1,25
1,00
0,75
0,50
0,25
0,00
0 1 2 3 4 5 6 7 8 9 10 11 12 13
Distance (Km)
d)
5 km
N
B01_010083_2567

CC-62

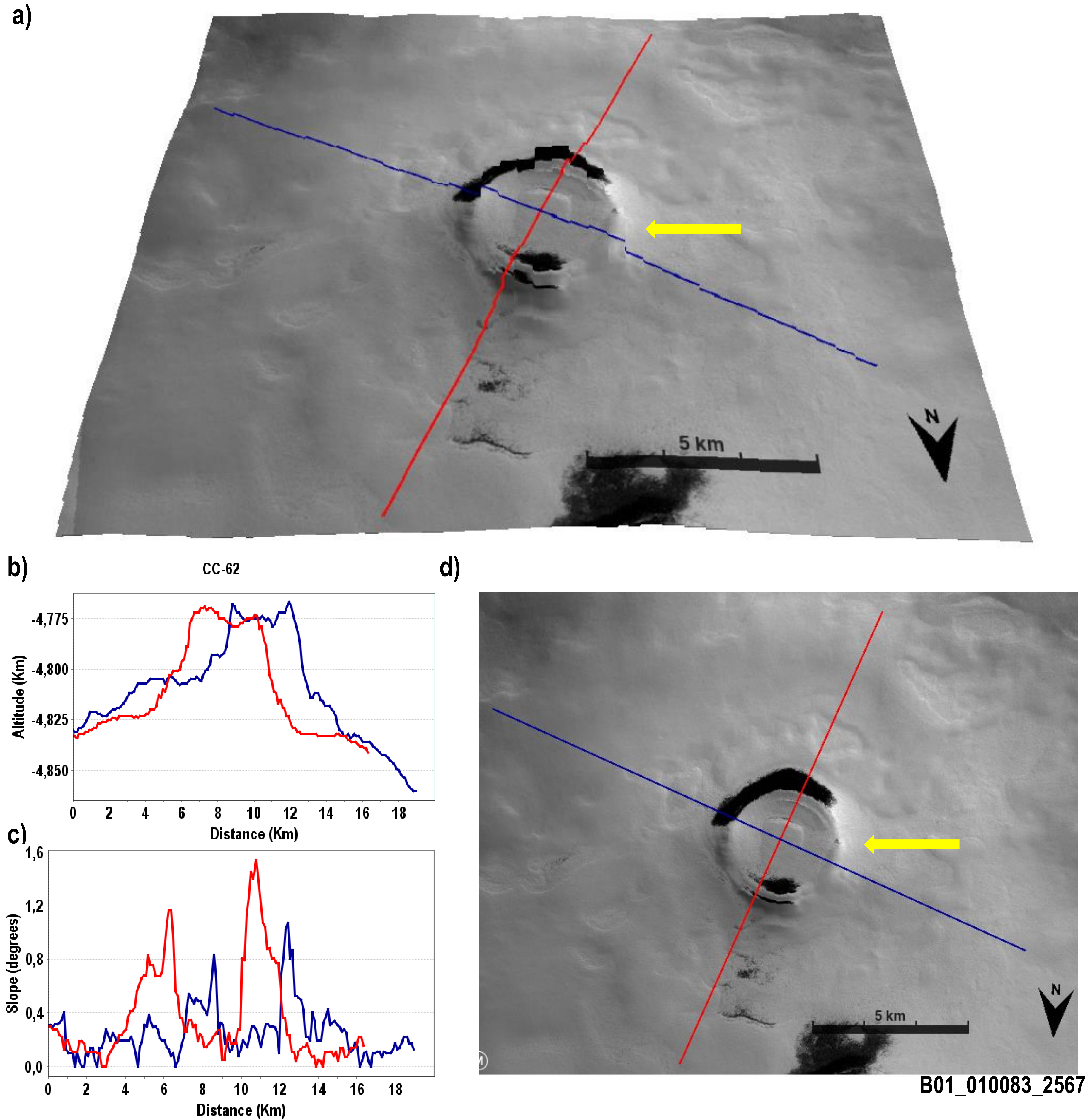

B01_010083_2567

CC-63

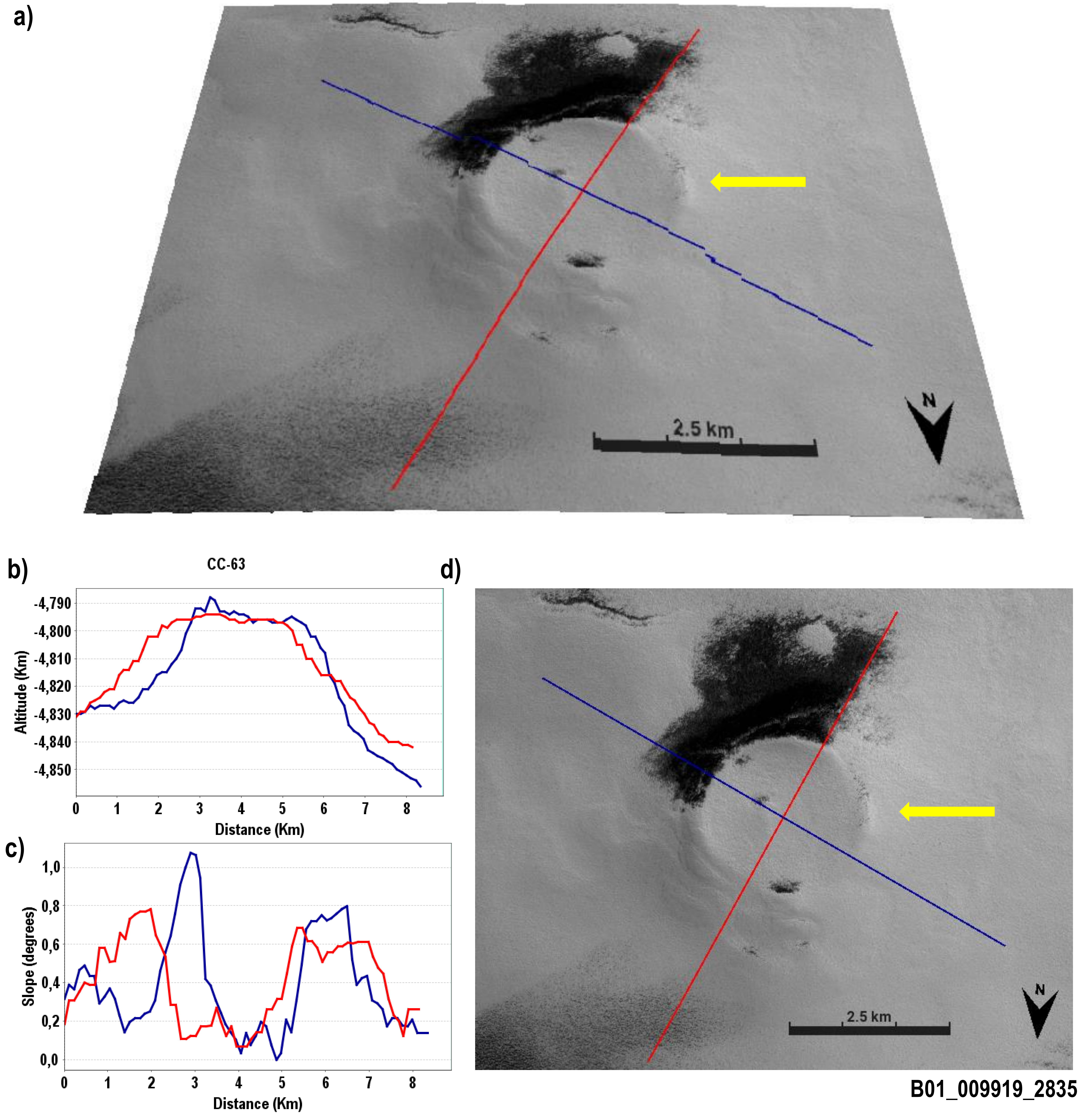

a)
2.5 km
N
b)
CC-63
Altitude (Km)
-4,790
-4,800
-4,810
-4,820
-4,830
-4,840
-4,850
Distance (Km)
0
1
2
3
4
5
6
7
8
c)
Slope (degrees)
1,0
0,8
0,6
0,4
0,2
0,0
Distance (Km)
d)
2.5 km
N
B01_009919_2835

CC-64

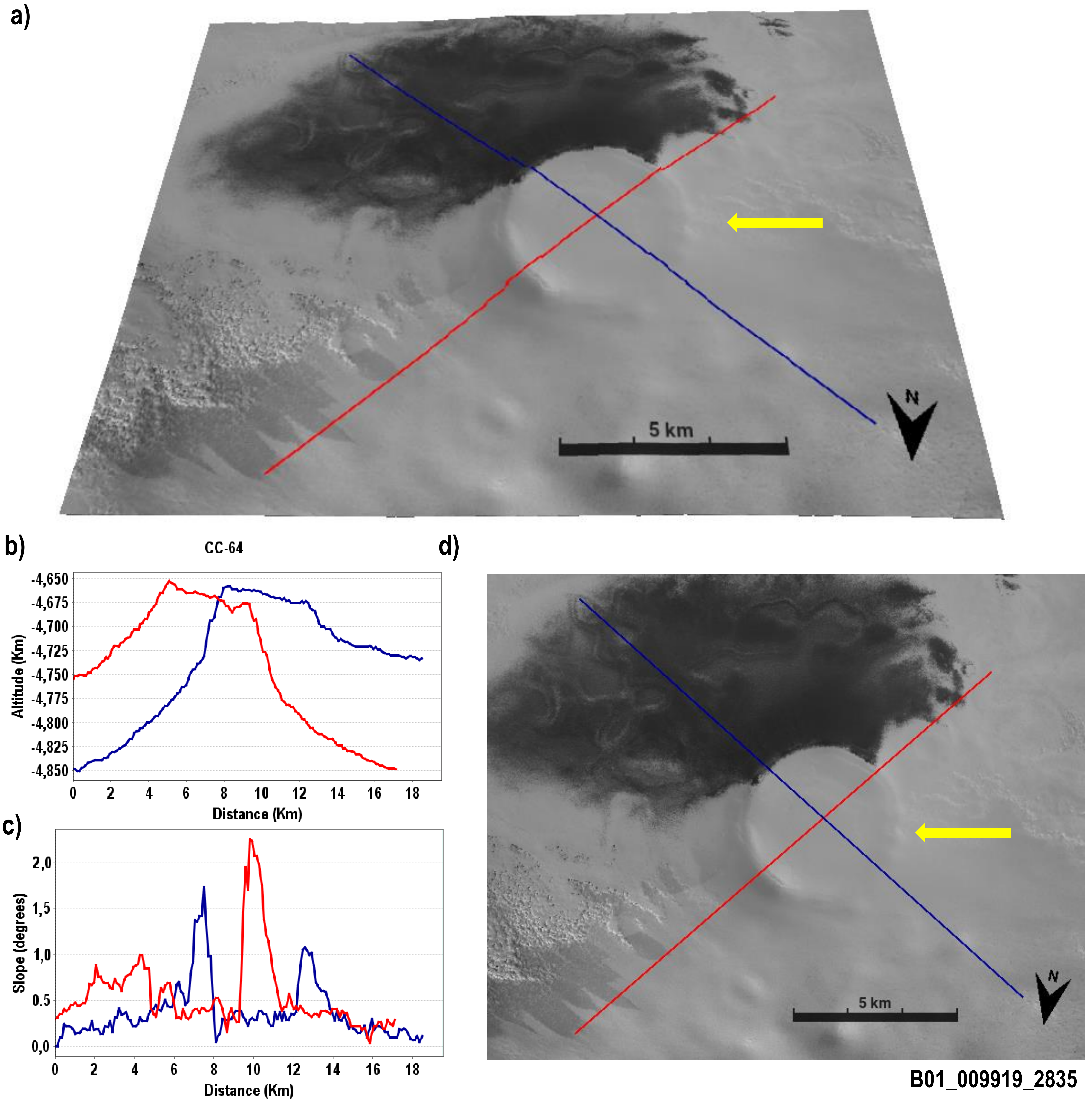
a)
5 km
N
b)
CC-64
Altitude (Km)
-4,650
-4,675
-4,700
-4,725
-4,750
-4,775
-4,800
-4,825
-4,850
Distance (Km)
0 2 4 6 8 10 12 14 16 18
c)
Slope (degrees)
0,0
0.5
1,0
1,5
2,0
Distance (Km)
0 2 4 6 8 10 12 14 16 18
d)
5 km
N
B01_009919_2835

CC-65

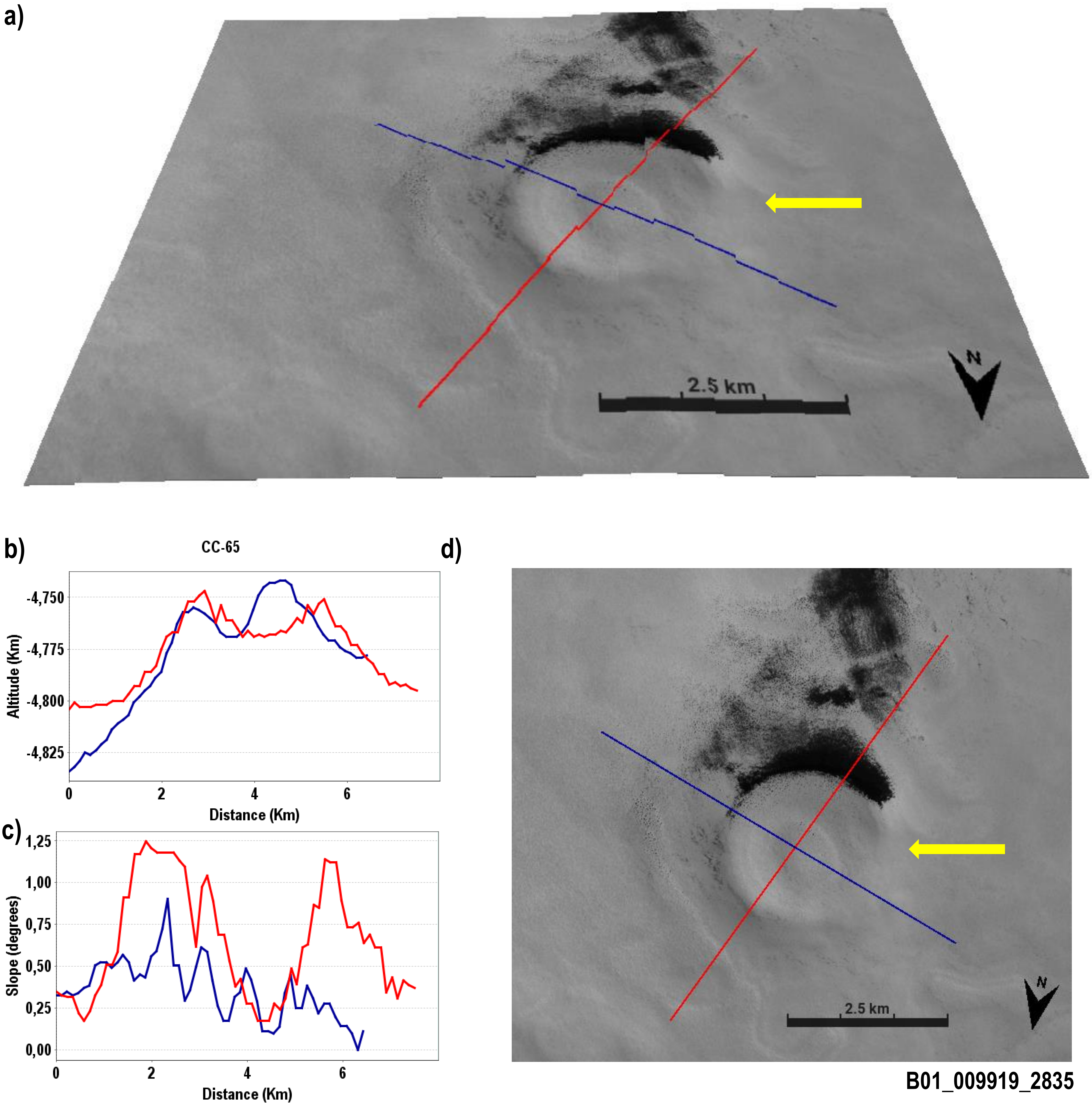

a)
2.5 km
N
b)
CC-65
-4,750
-4,775
-4,800
-4,825
Altitude (Km)
0
2
4
6
Distance (Km)
c)
1,25
1,00
0,75
0,50
0,25
0,00
Slope (degrees)
0
2
4
6
Distance (Km)
d)
2.5 km
N
B01_009919_2835

CC-66

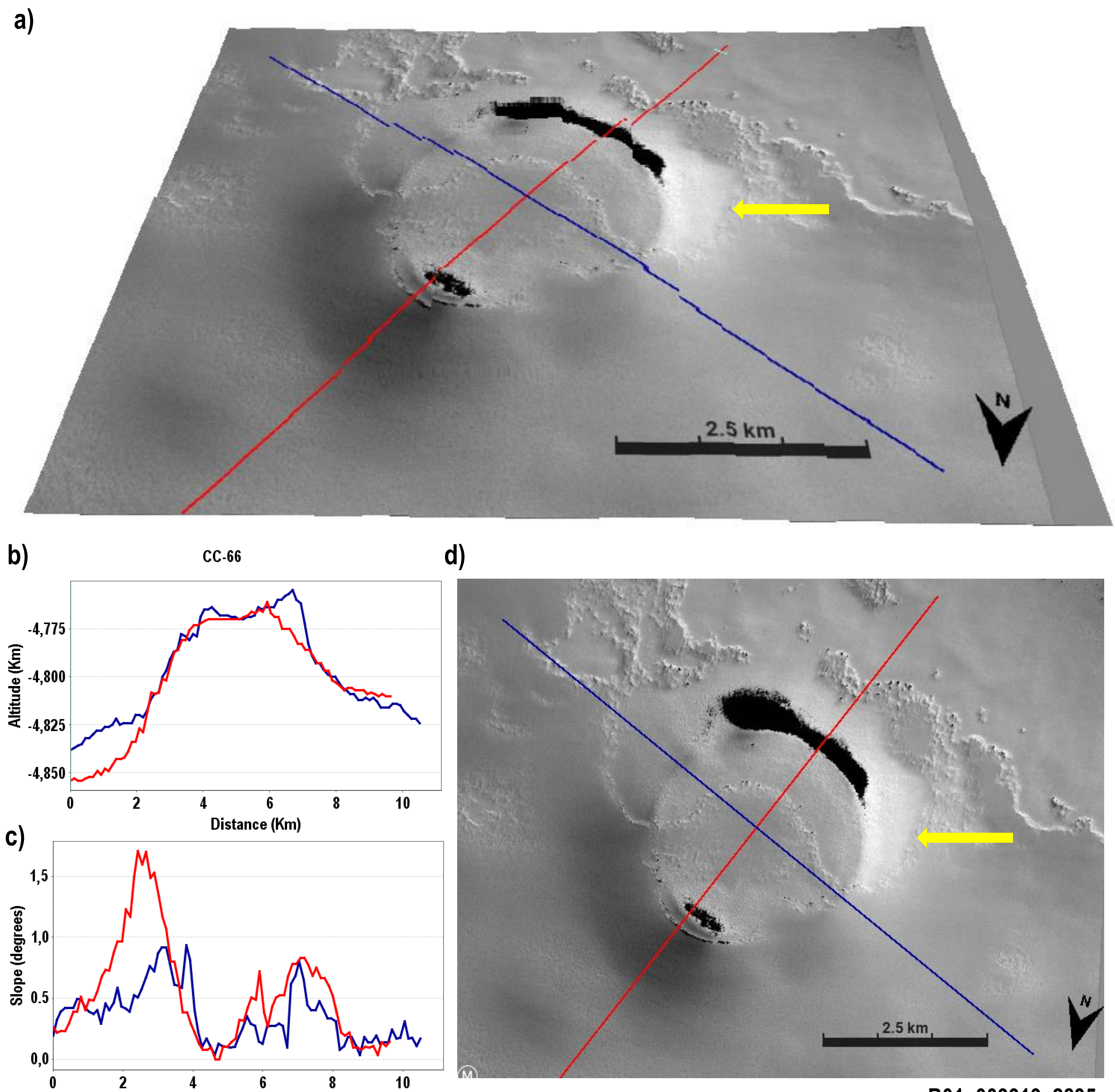

B01_009919_2835

CC-67

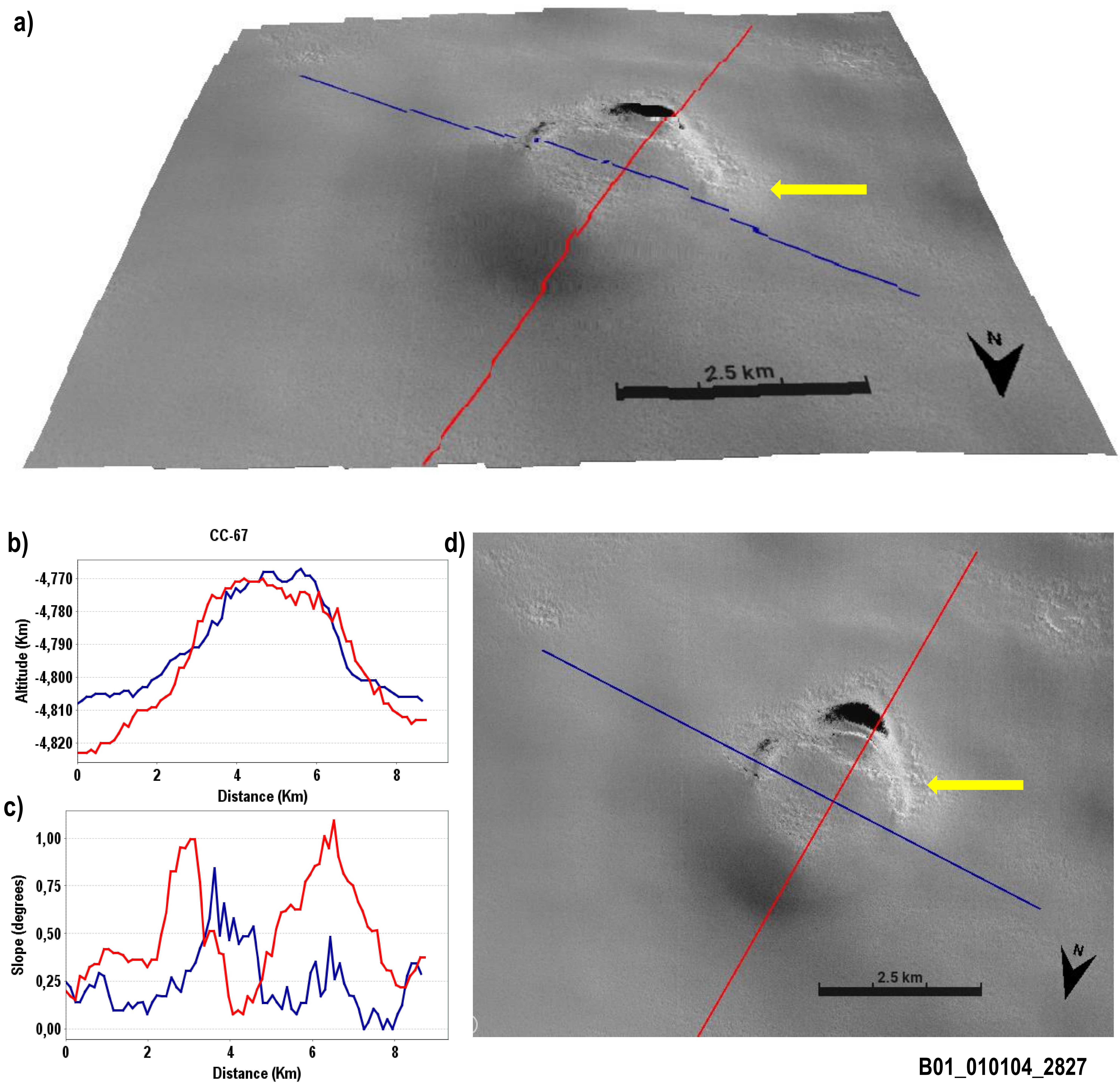

B01_010104_2827

CC-68

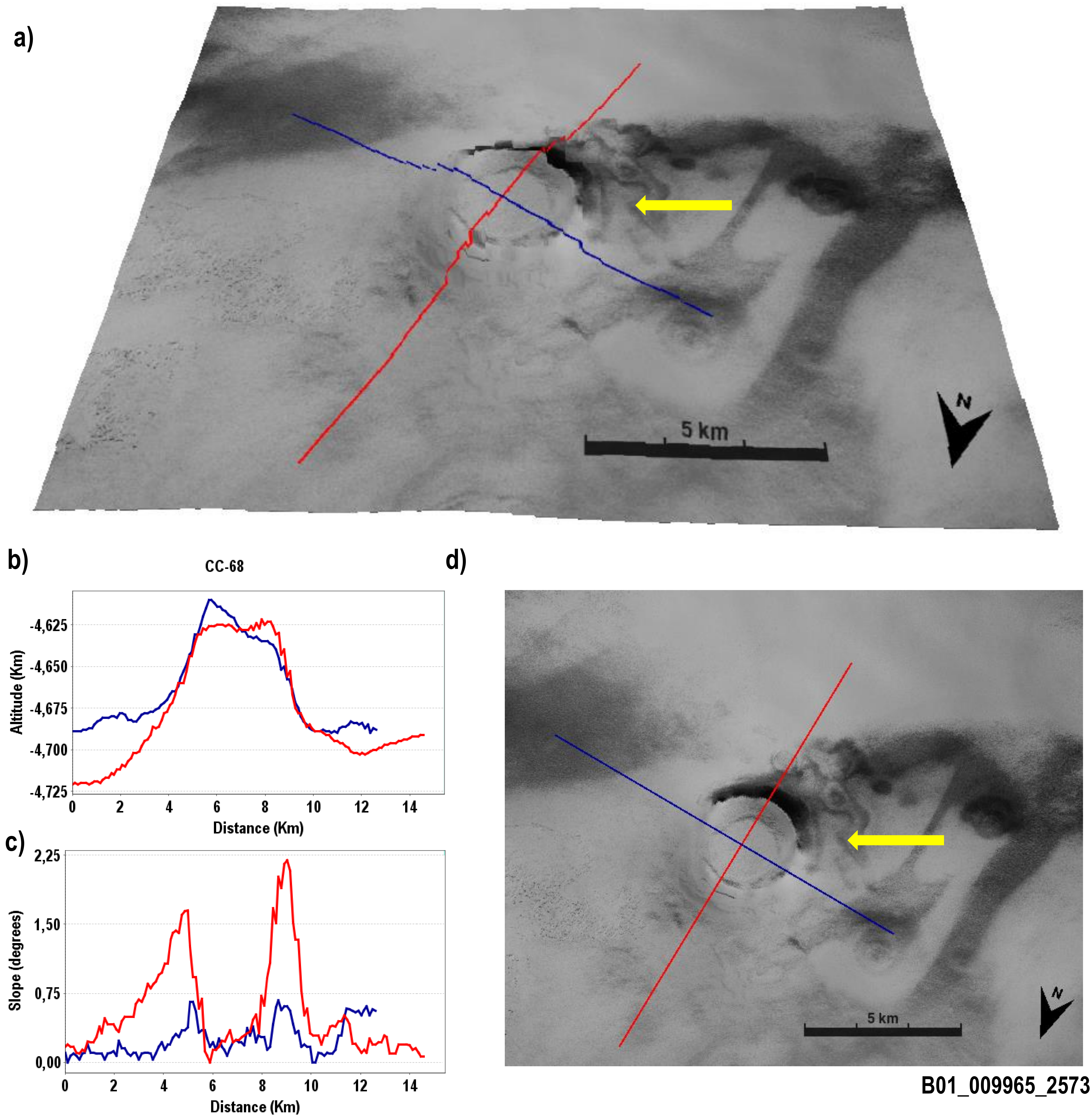

B01_009965_2573

CC-69

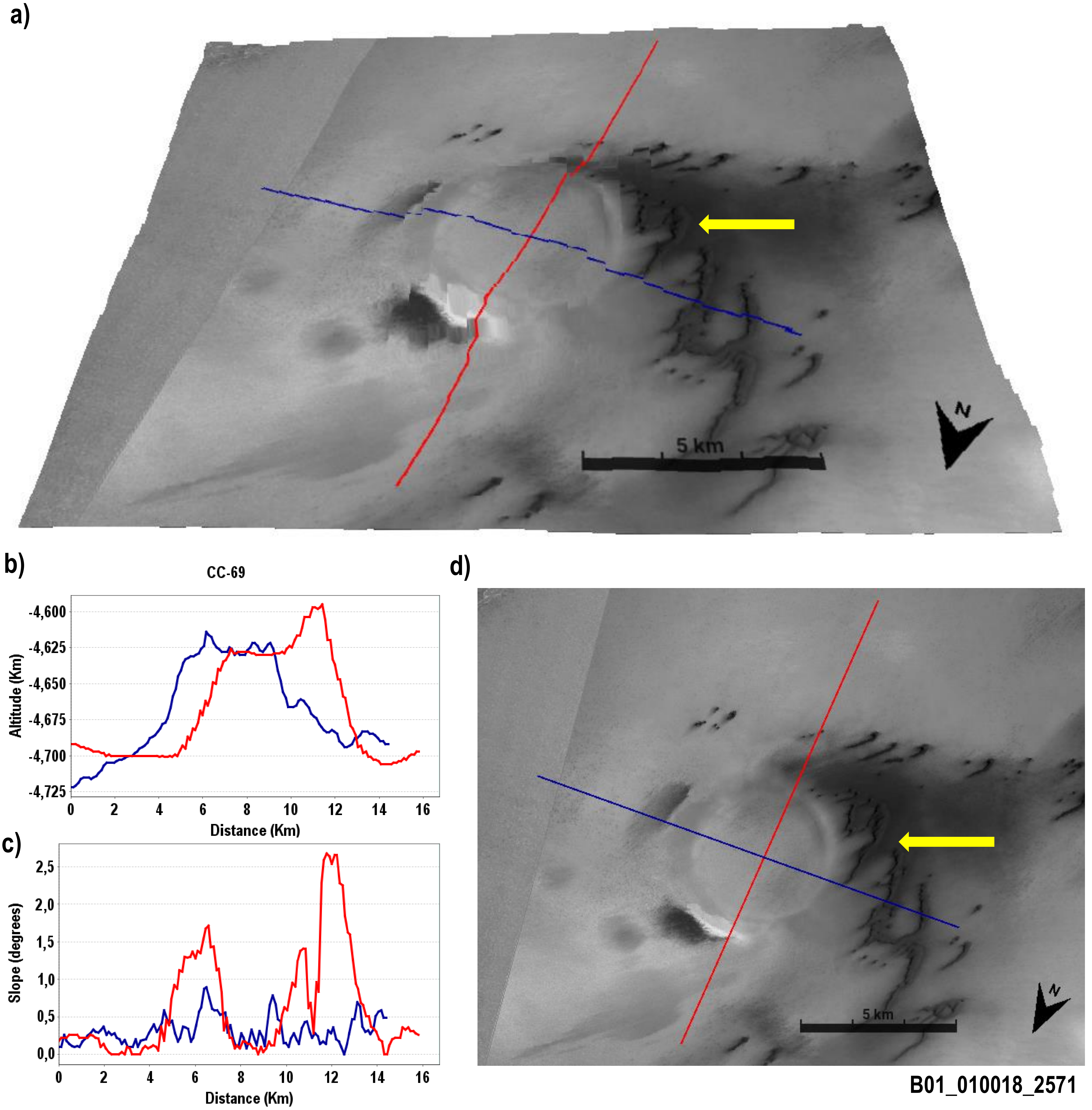

a)
5 km
N
b)
CC-69
-4,600
-4,625
-4,650
-4,675
-4,700
-4,725
Altitude (Km)
0
2
4
6
8
10
12
14
16
Distance (Km)
c)
2,5
2,0
1,5
1,0
0,5
0,0
Slope (degrees)
0
2
4
6
8
10
12
14
16
Distance (Km)
d)
5 km
N
B01_010018_2571

CC-70

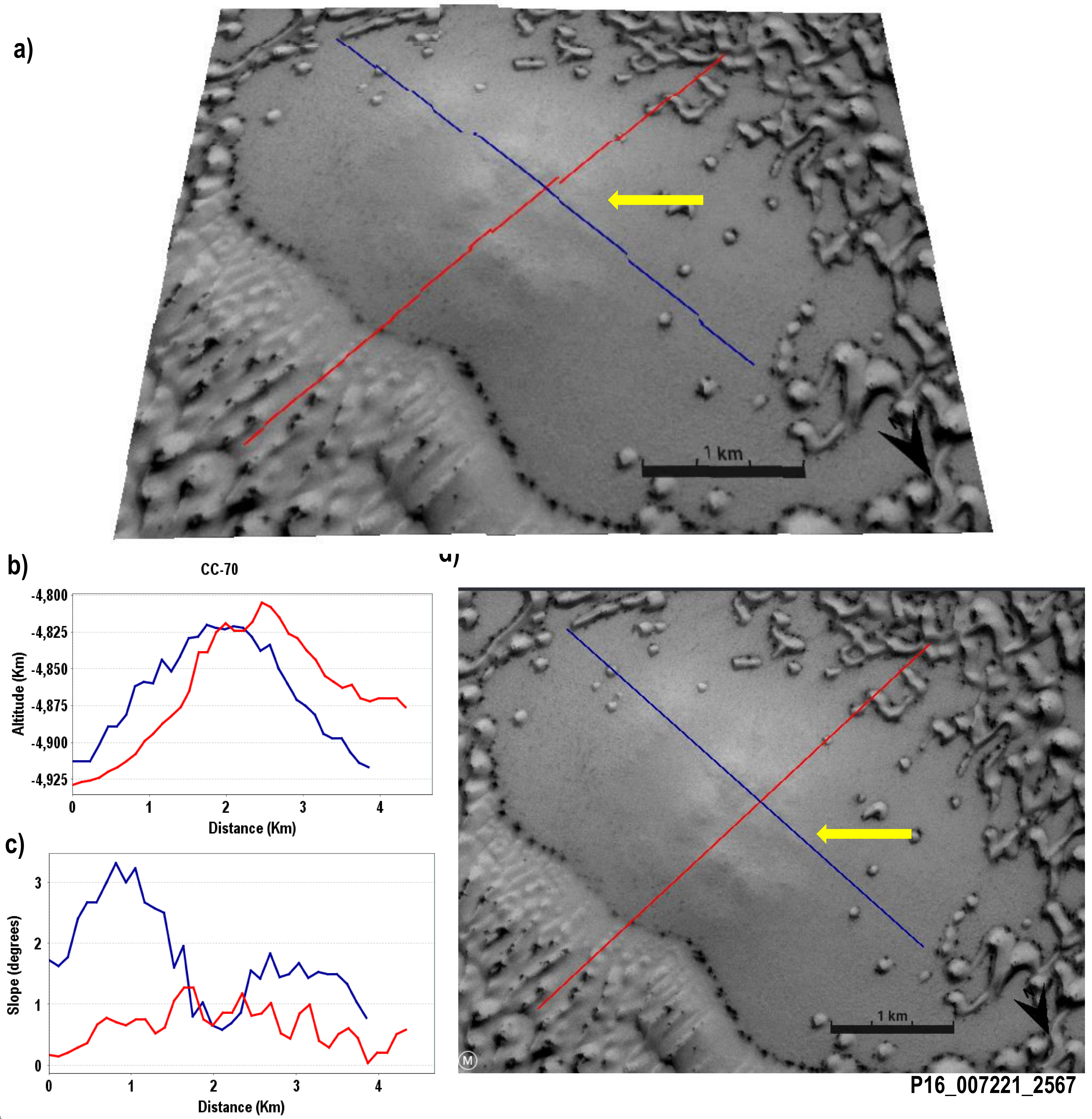
a)
1 km
b)
CC-70
-4,800
-4,825
-4,850
-4,875
-4,900
-4,925
Altitude (Km)
0
1
2
3
4
Distance (Km)
c)
3
2
1
0
Slope (degrees)
0
1
2
3
4
Distance (Km)
d)
1 km
P16_007221_2567

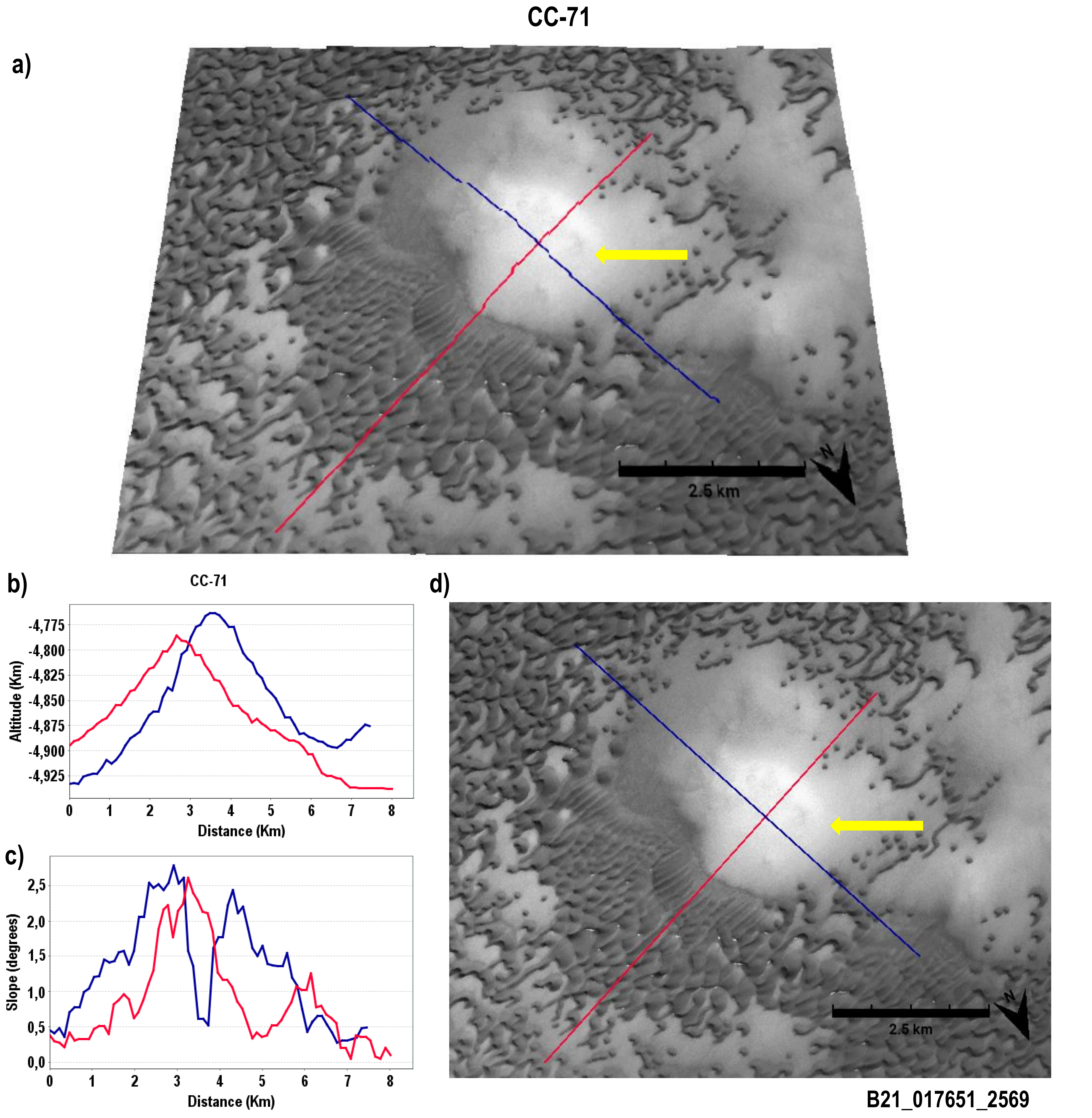
CC-71
a)
2.5 km
N
b)
CC-71
-4,775
-4,800
-4,825
-4,850
-4,875
-4,900
-4,925
Altitude (Km)
0
1
2
3
4
5
6
7
8
Distance (Km)
c)
2,5
2,0
1,5
1,0
0,5
0,0
Slope (degrees)
0
1
2
3
4
5
6
7
8
Distance (Km)
d)
2.5 km
N
B21_017651_2569

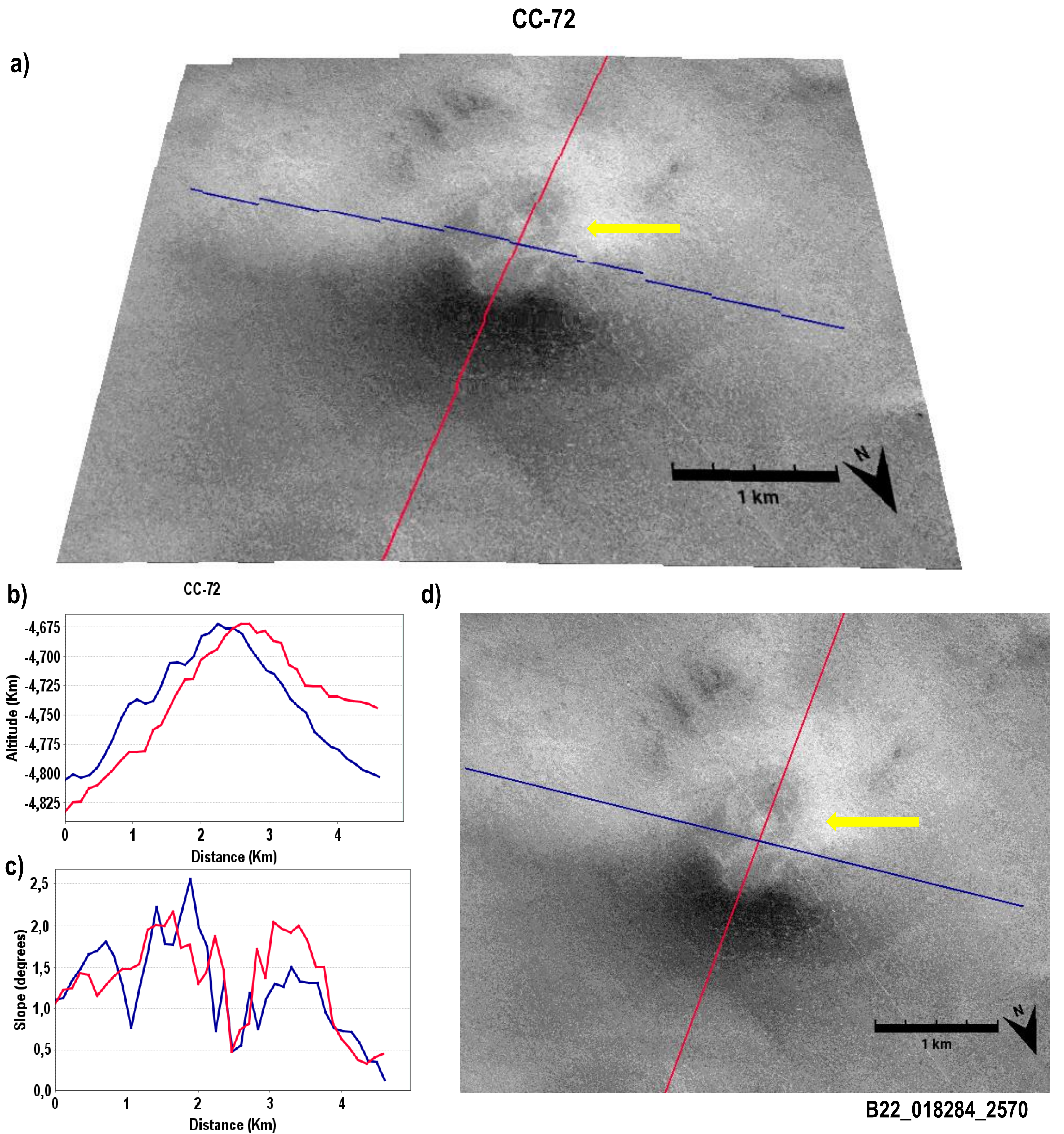
CC-72
a)
1 km
N
b)
CC-72
Altitude (Km)
-4,675
-4,700
-4,725
-4,750
-4,775
-4,800
-4,825
Distance (Km)
0
1
2
3
4
c)
Slope (degrees)
2,5
2,0
1,5
1,0
0,5
0,0
Distance (Km)
d)
1 km
N
B22_018284_2570

CC-73

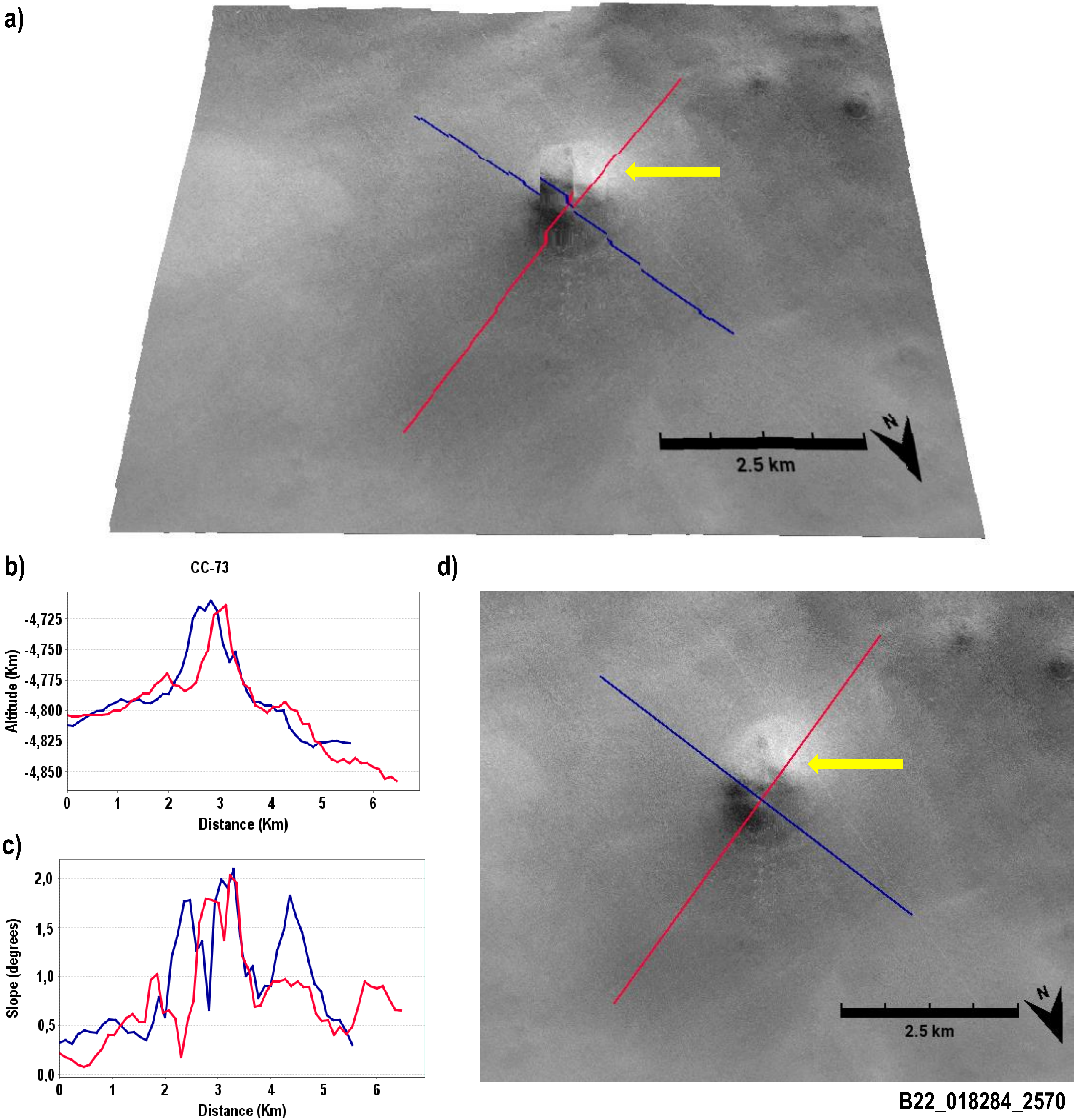
a)
2.5 km
N
b)
CC-73
Altitude (Km)
-4,725
-4,750
-4,775
-4,800
-4,825
-4,850
Distance (Km)
0
1
2
3
4
5
6
c)
Slope (degrees)
2,0
1,5
1,0
0,5
0,0
Distance (Km)
d)
2.5 km
N
B22_018284_2570

CC-74

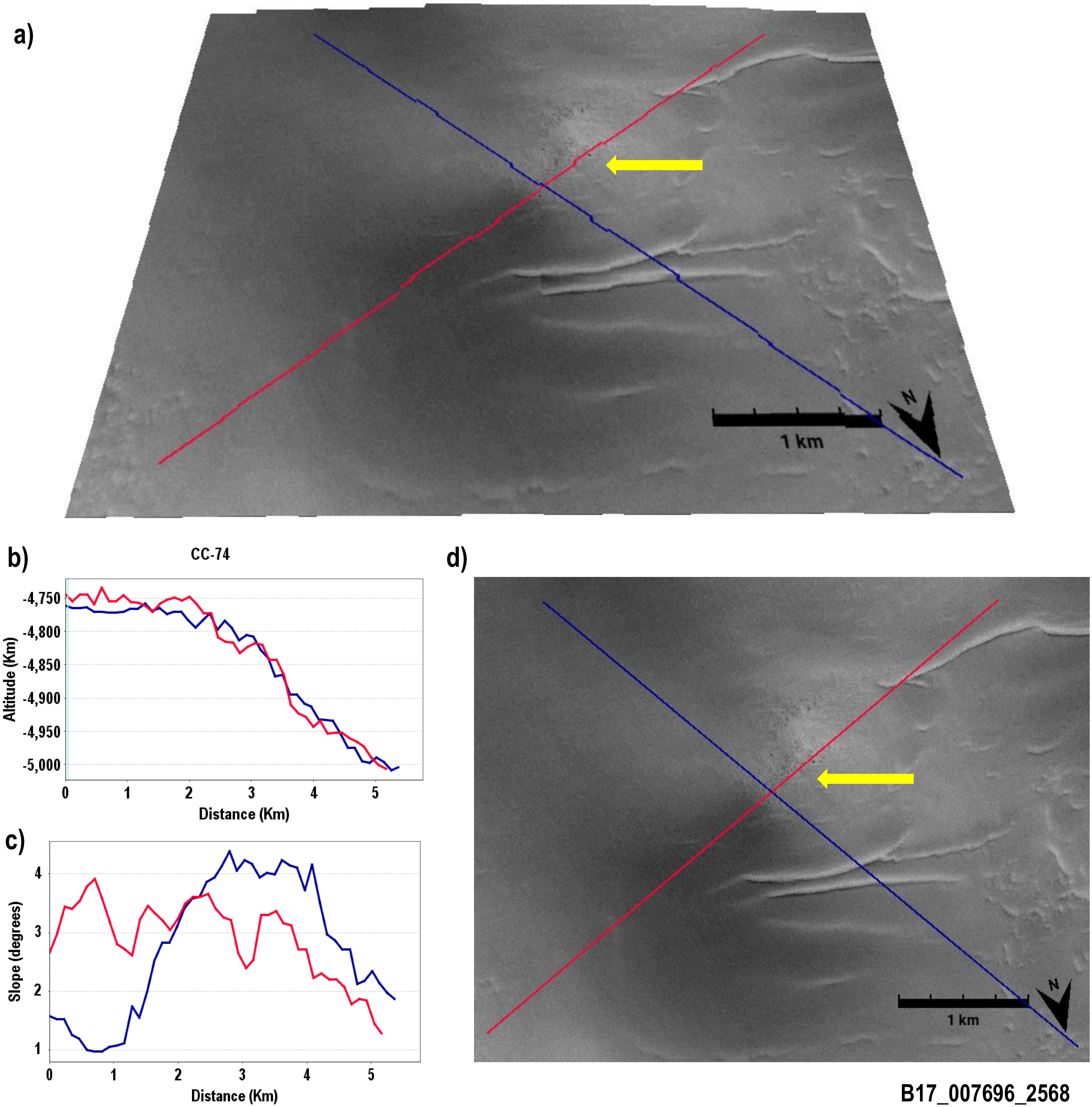

B17_007696_2568

CC-75

a)

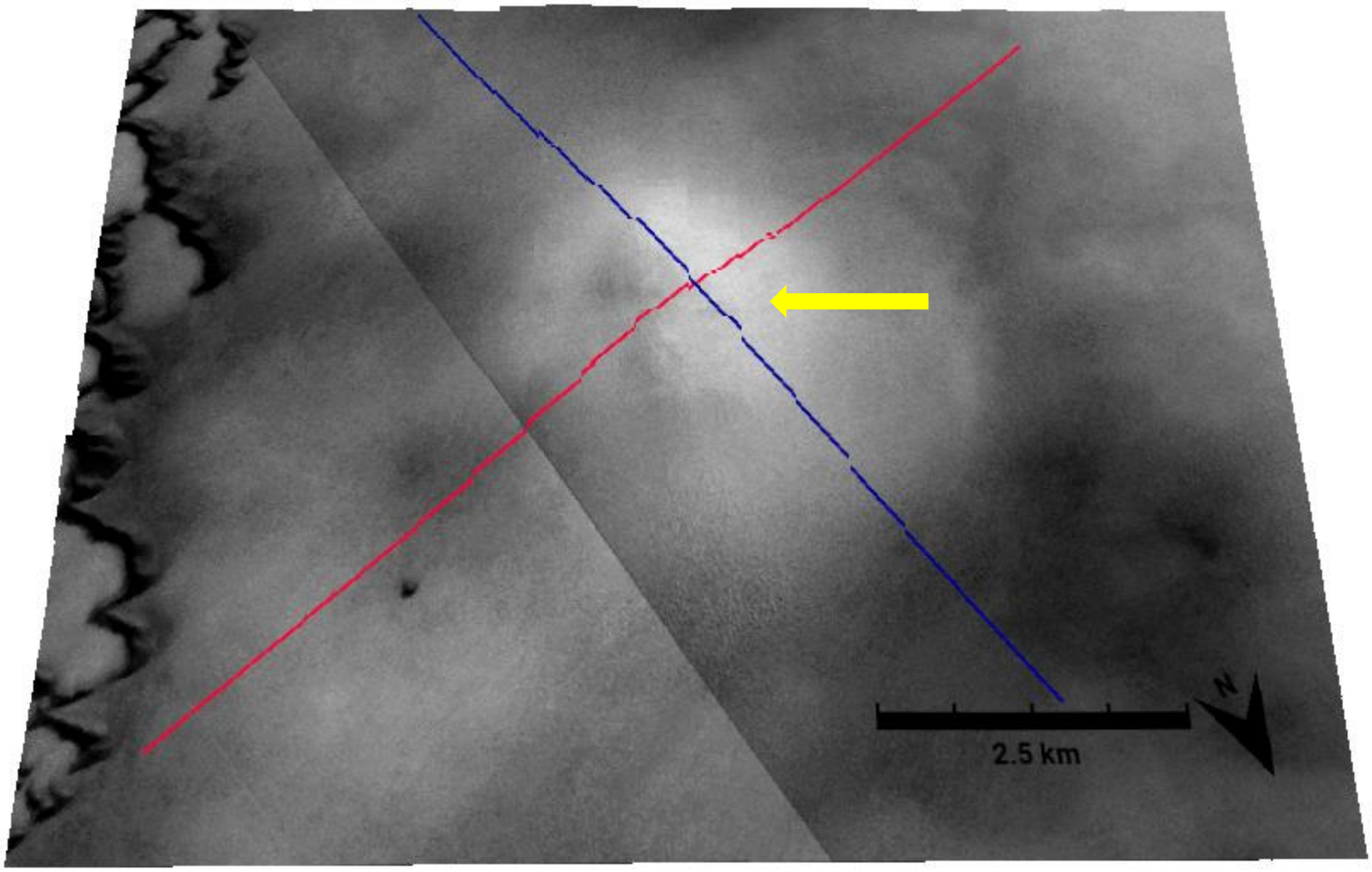

b)

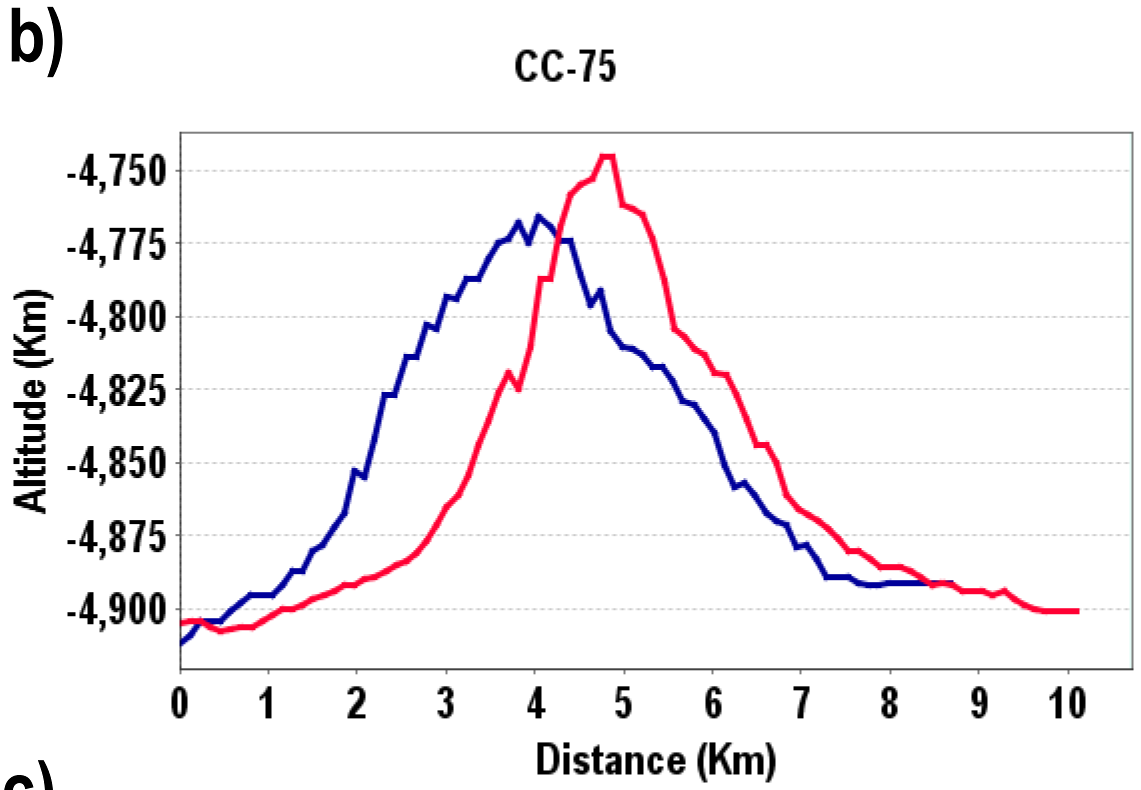

d)

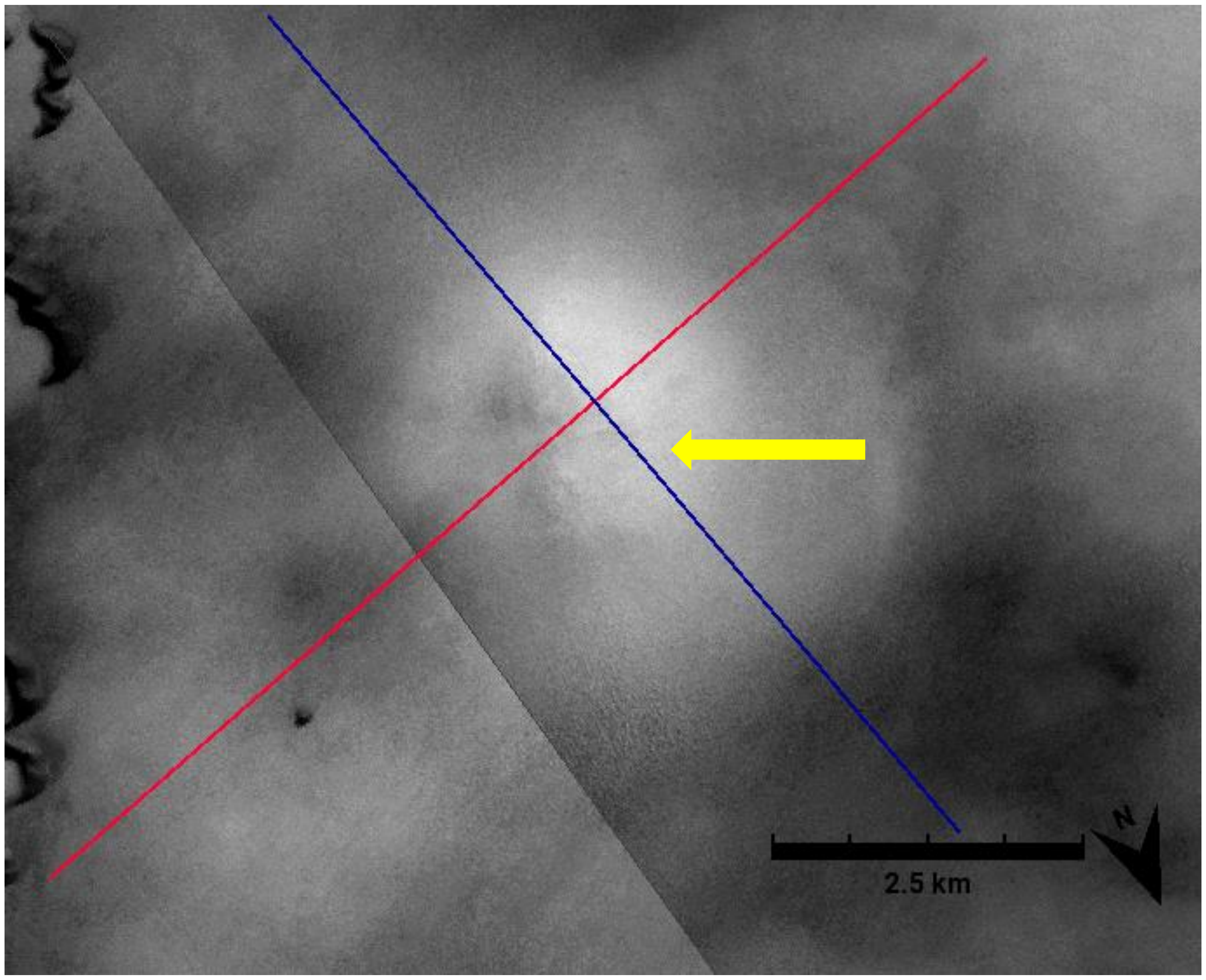

c)

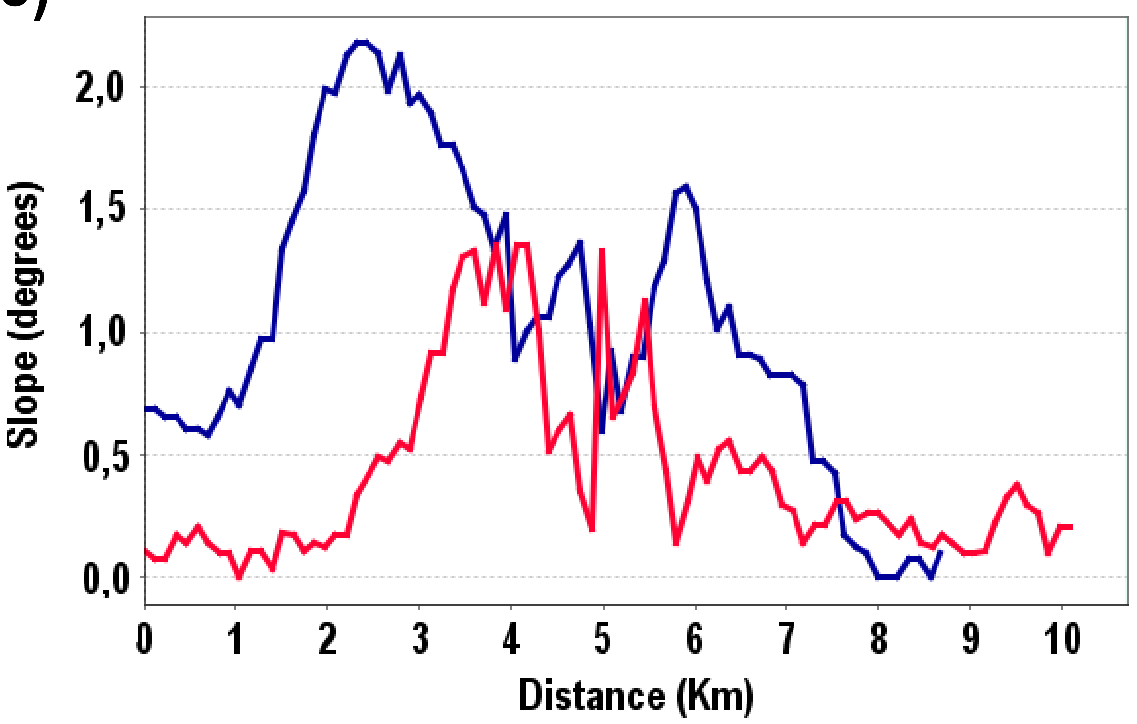

B22_018284_2570

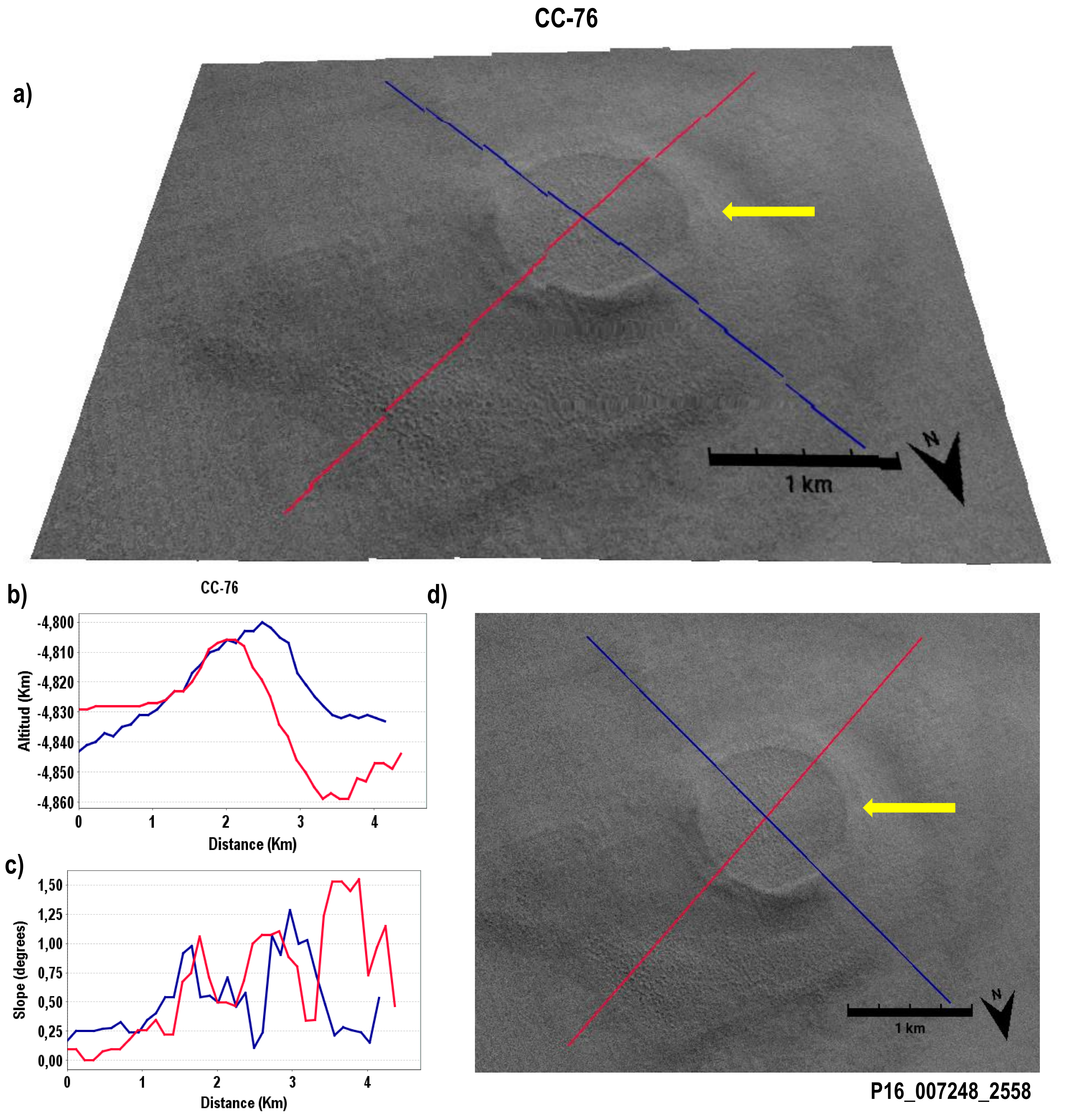
CC-76
a)
1 km
N
b)
CC-76
Altitud (Km)
-4,800
-4,810
-4,820
-4,830
-4,840
-4,850
-4,860
0
1
2
3
4
Distance (Km)
c)
Slope (degrees)
1,50
1,25
1,00
0,75
0,50
0,25
0,00
0
1
2
3
4
Distance (Km)
d)
1 km
N
P16_007248_2558

CC-77

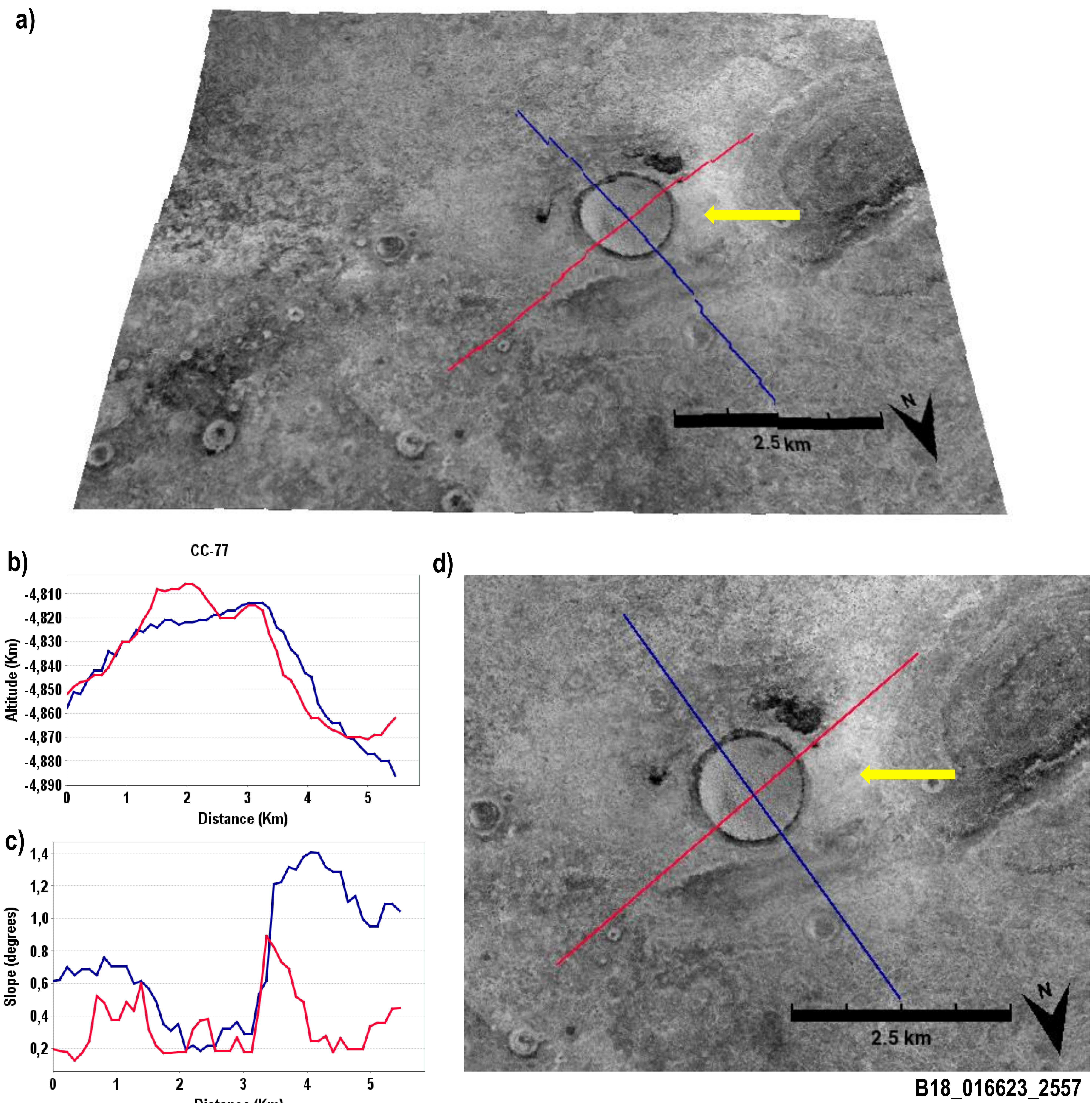

B18_016623_2557

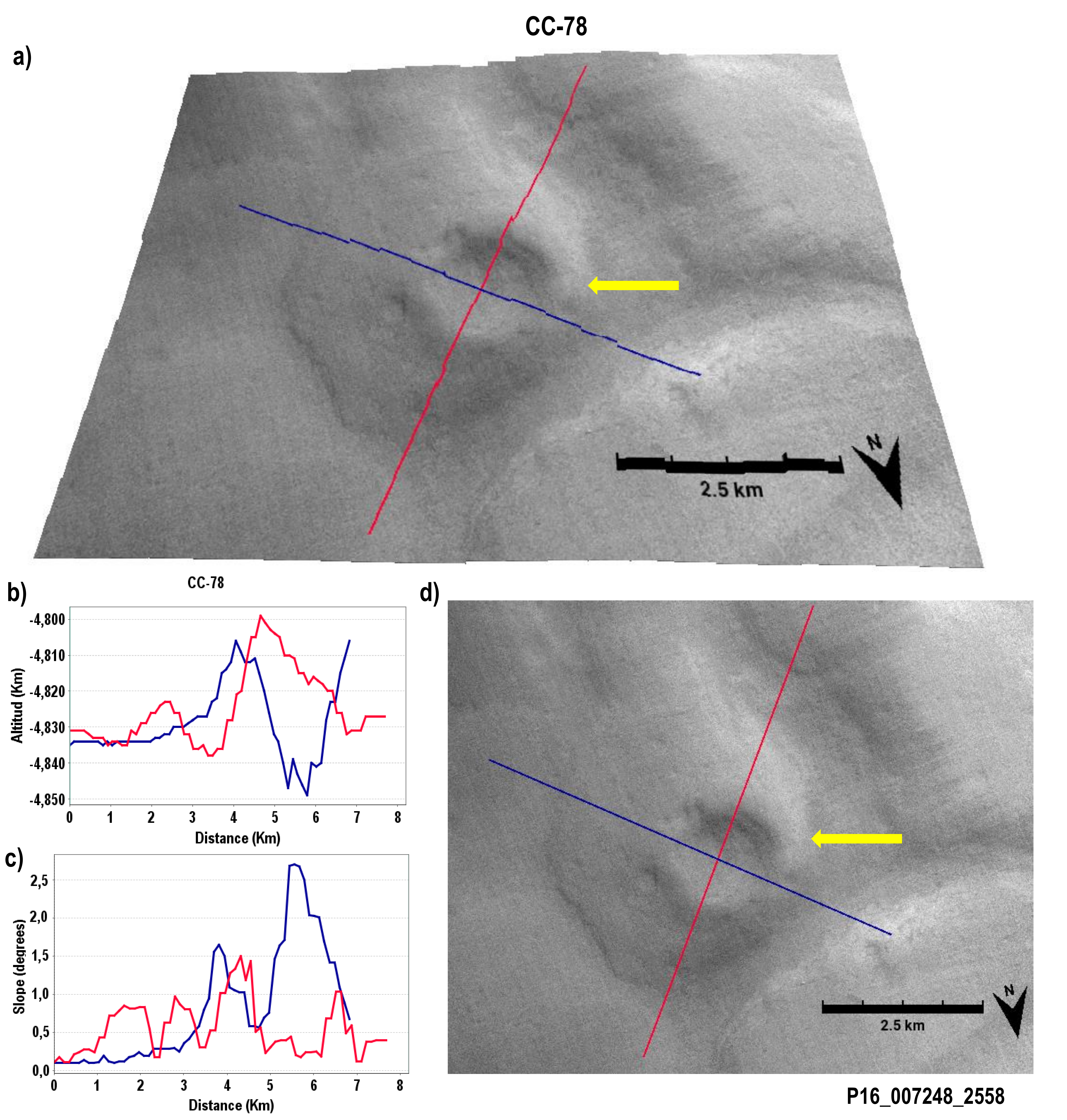
CC-78
a)
2.5 km
N
b)
CC-78
-4,800
-4,810
-4,820
-4,830
-4,840
-4,850
Altitud (Km)
0 1 2 3 4 5 6 7 8
Distance (Km)
c)
2,5
2,0
1,5
1,0
0,5
0,0
Slope (degrees)
0 1 2 3 4 5 6 7 8
Distance (Km)
d)
2.5 km
N
P16_007248_2558

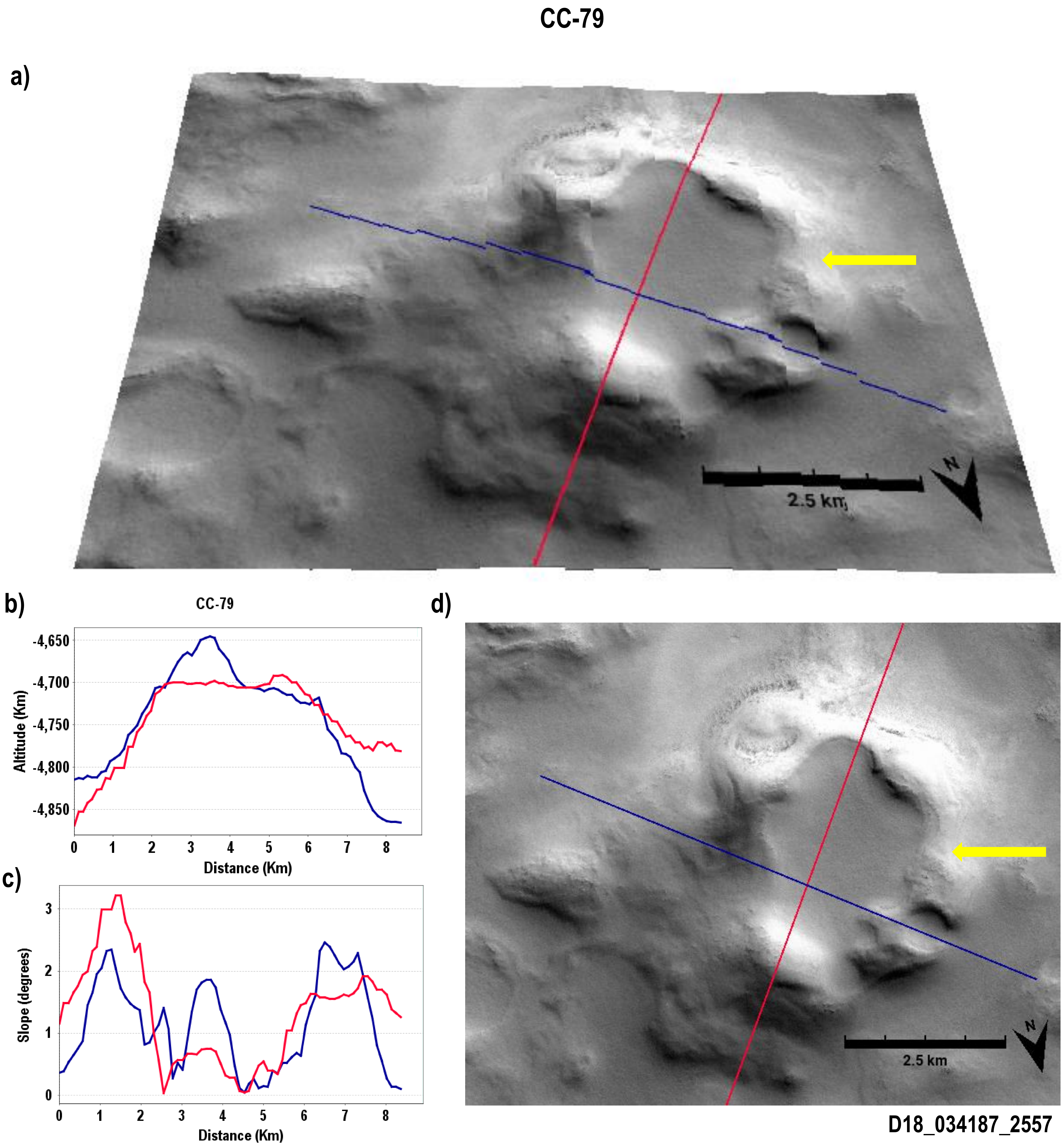
CC-79
a)
2.5 km
N
b)
CC-79
Altitude (Km)
-4,650
-4,700
-4,750
-4,800
-4,850
Distance (Km)
c)
Slope (degrees)
Distance (Km)
d)
2.5 km
N
D18_034187_2557

CC-80

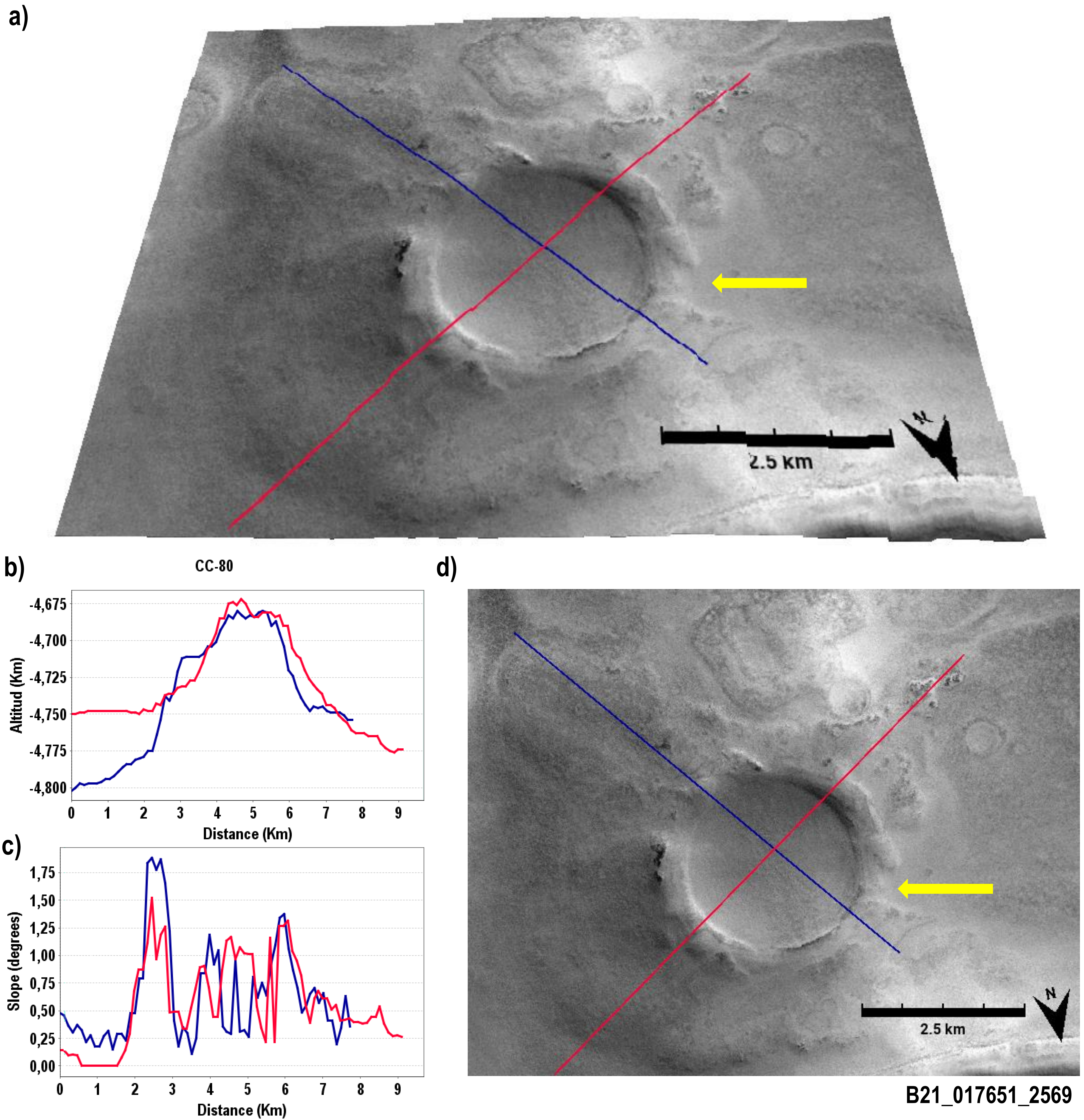

B21_017651_2569

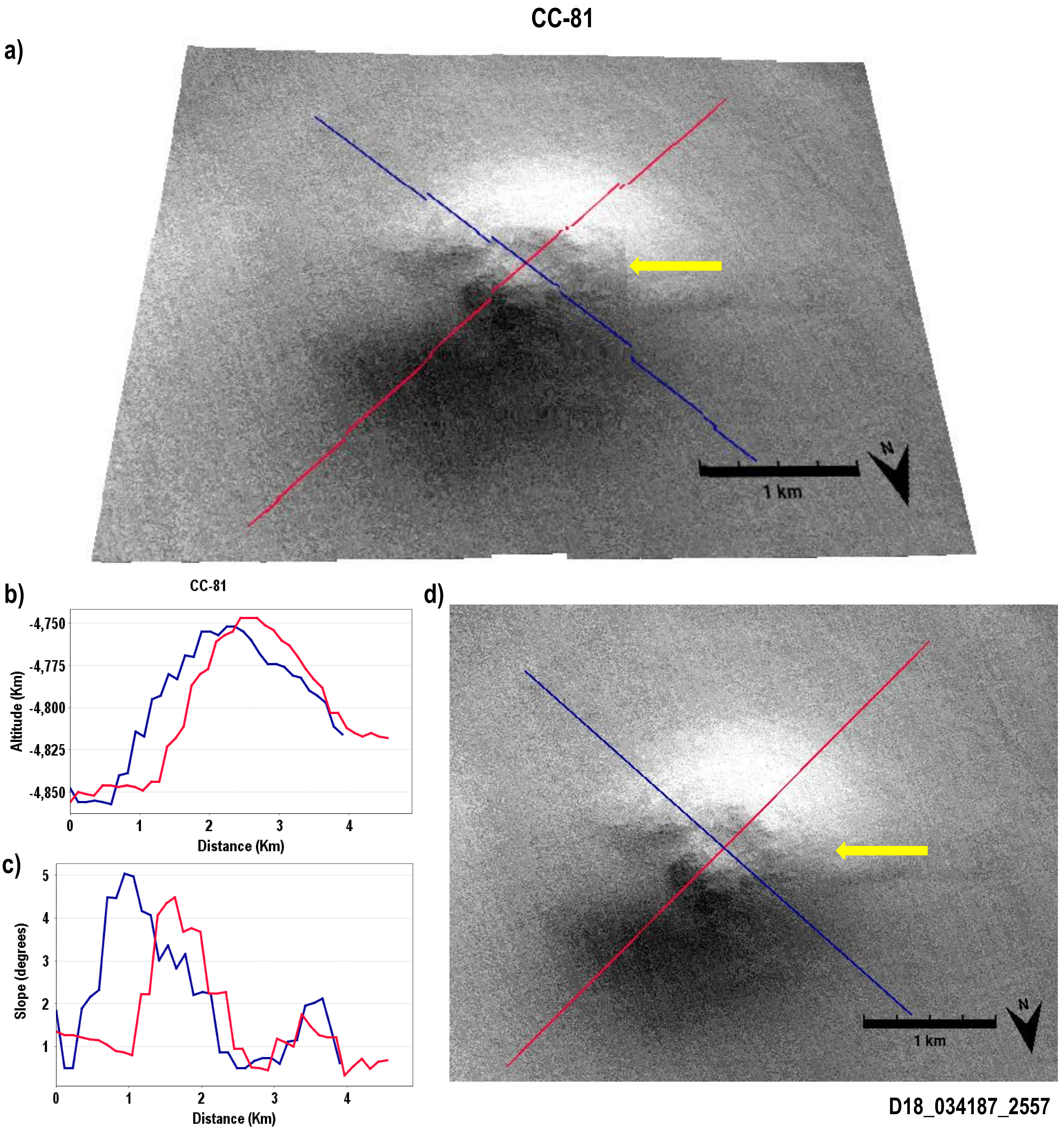
CC-81
a)
1 km
N
b)
CC-81
Altitude (Km)
-4,750
-4,775
-4,800
-4,825
-4,850
Distance (Km)
0
1
2
3
4
c)
Slope (degrees)
5
4
3
2
1
Distance (Km)
d)
1 km
N
D18_034187_2557

CC-82

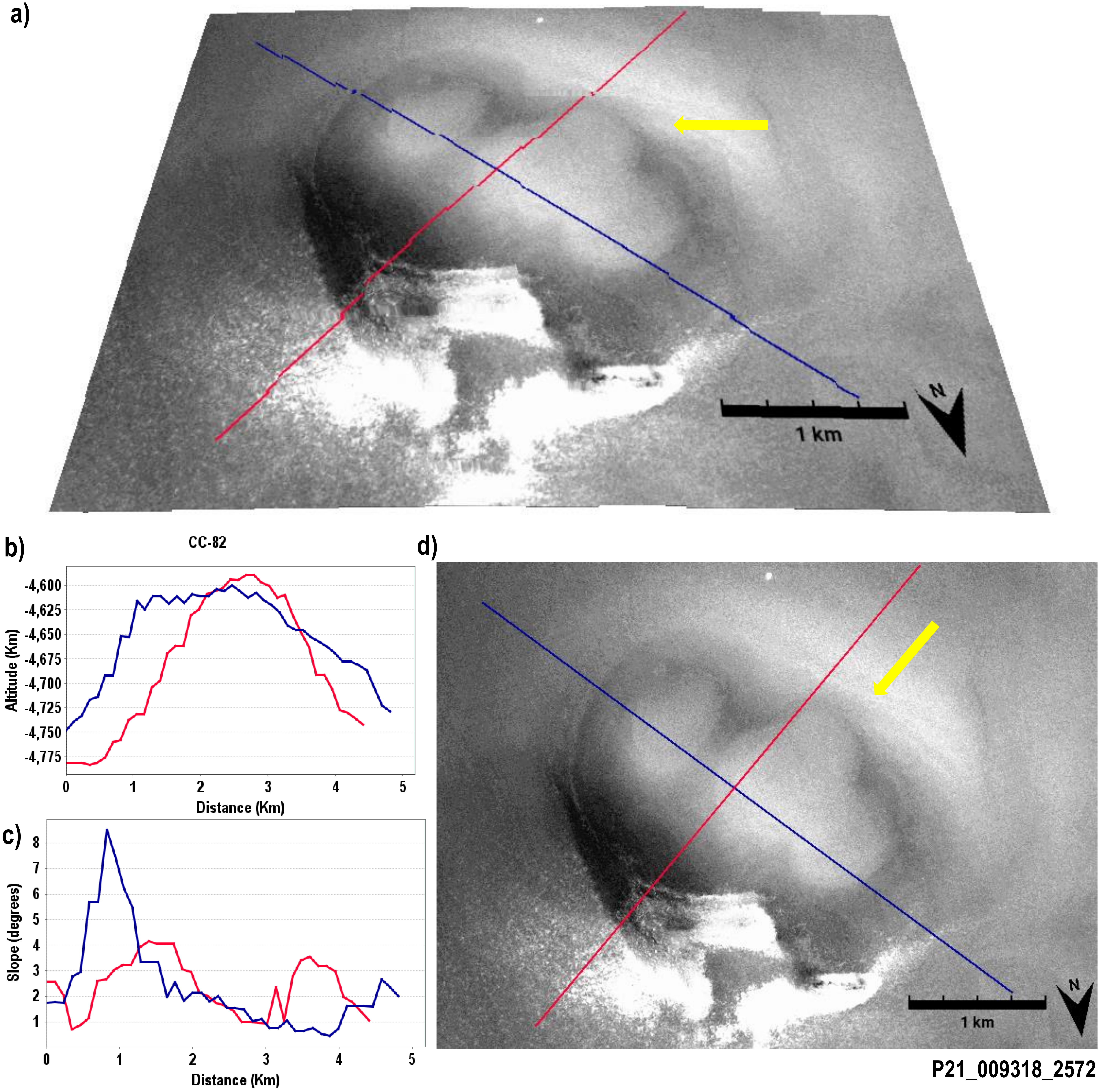
a)
1 km
N
b)
CC-82
Altitude (Km)
-4,600
-4,625
-4,650
-4,675
-4,700
-4,725
-4,750
-4,775
0
1
2
3
4
5
Distance (Km)
c)
Slope (degrees)
8
7
6
5
4
3
2
1
0
1
2
3
4
5
Distance (Km)
d)
1 km
N
P21_009318_2572

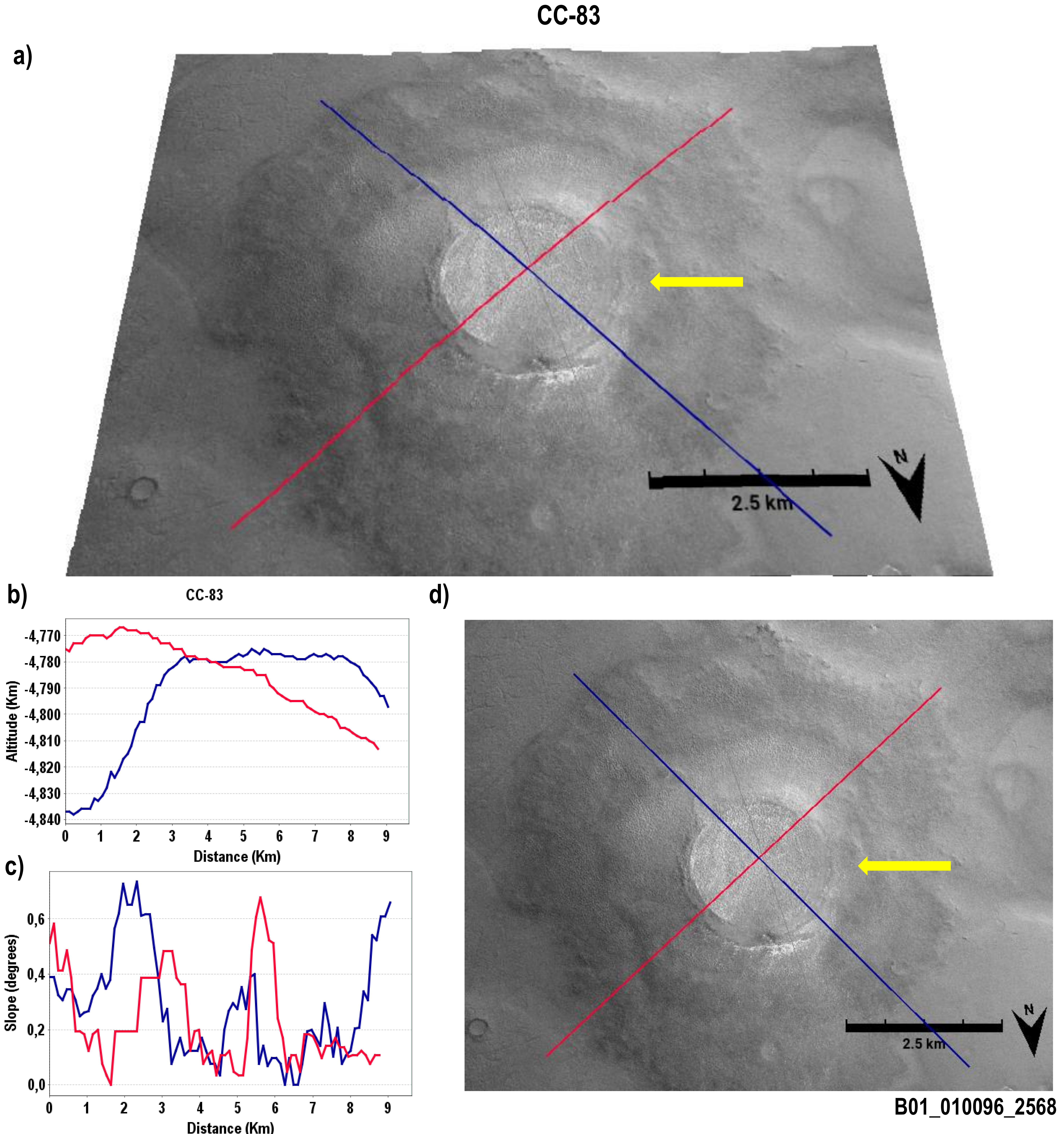

CC-83
a)
2.5 km
N
b)
CC-83
Altitude (Km)
-4,770
-4,780
-4,790
-4,800
-4,810
-4,820
-4,830
-4,840
0 1 2 3 4 5 6 7 8 9
Distance (Km)
c)
Slope (degrees)
0,6
0,4
0,2
0,0
0 1 2 3 4 5 6 7 8 9
Distance (Km)
d)
2.5 km
N
B01_010096_2568

CC-84

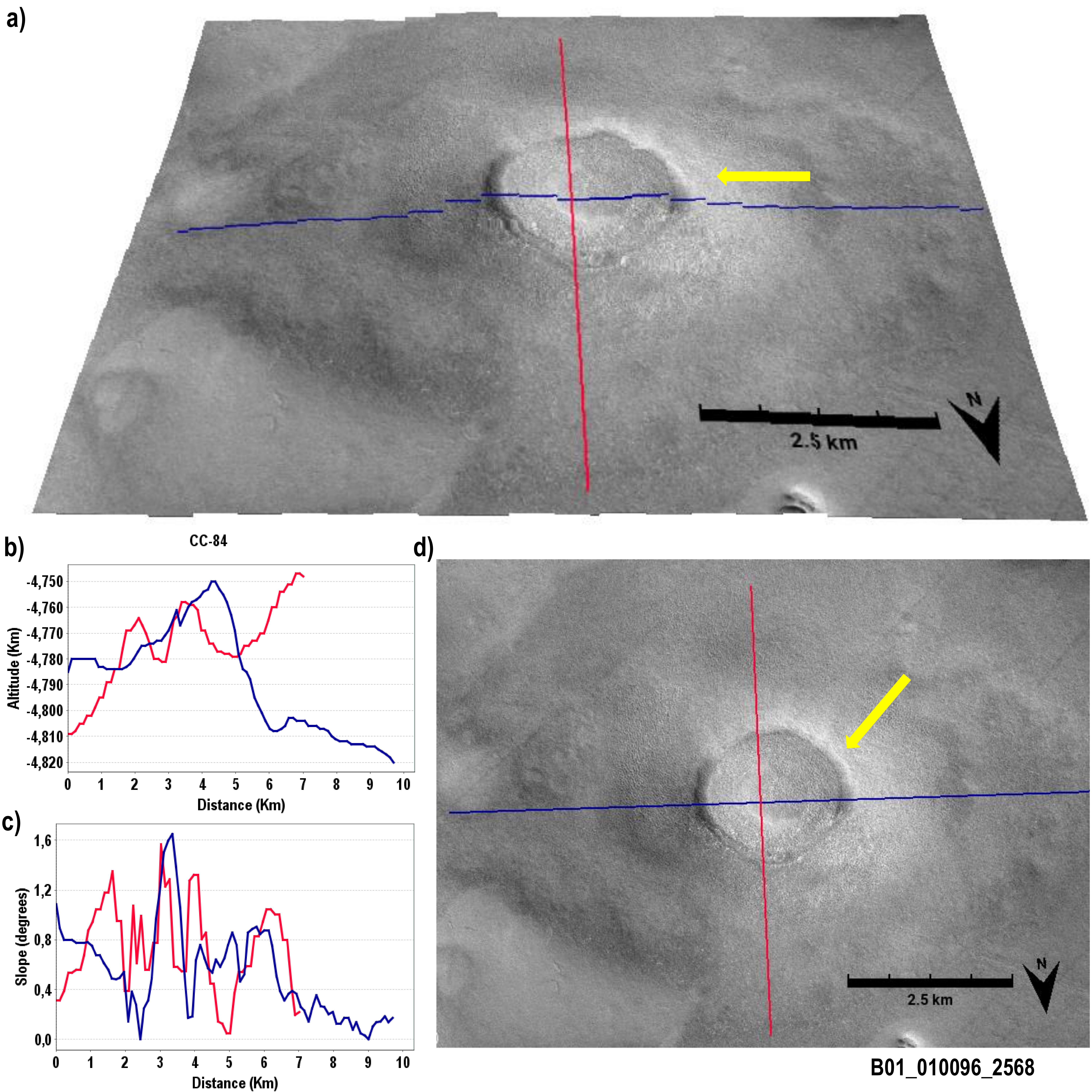
a)
2.5 km
N
b)
CC-84
-4,750
-4,760
-4,770
-4,780
-4,790
-4,800
-4,810
-4,820
Altitude (Km)
0 1 2 3 4 5 6 7 8 9 10
Distance (Km)
c)
1,6
1,2
0,8
0,4
0,0
Slope (degrees)
0 1 2 3 4 5 6 7 8 9 10
Distance (Km)
d)
2.5 km
N
B01_010096_2568

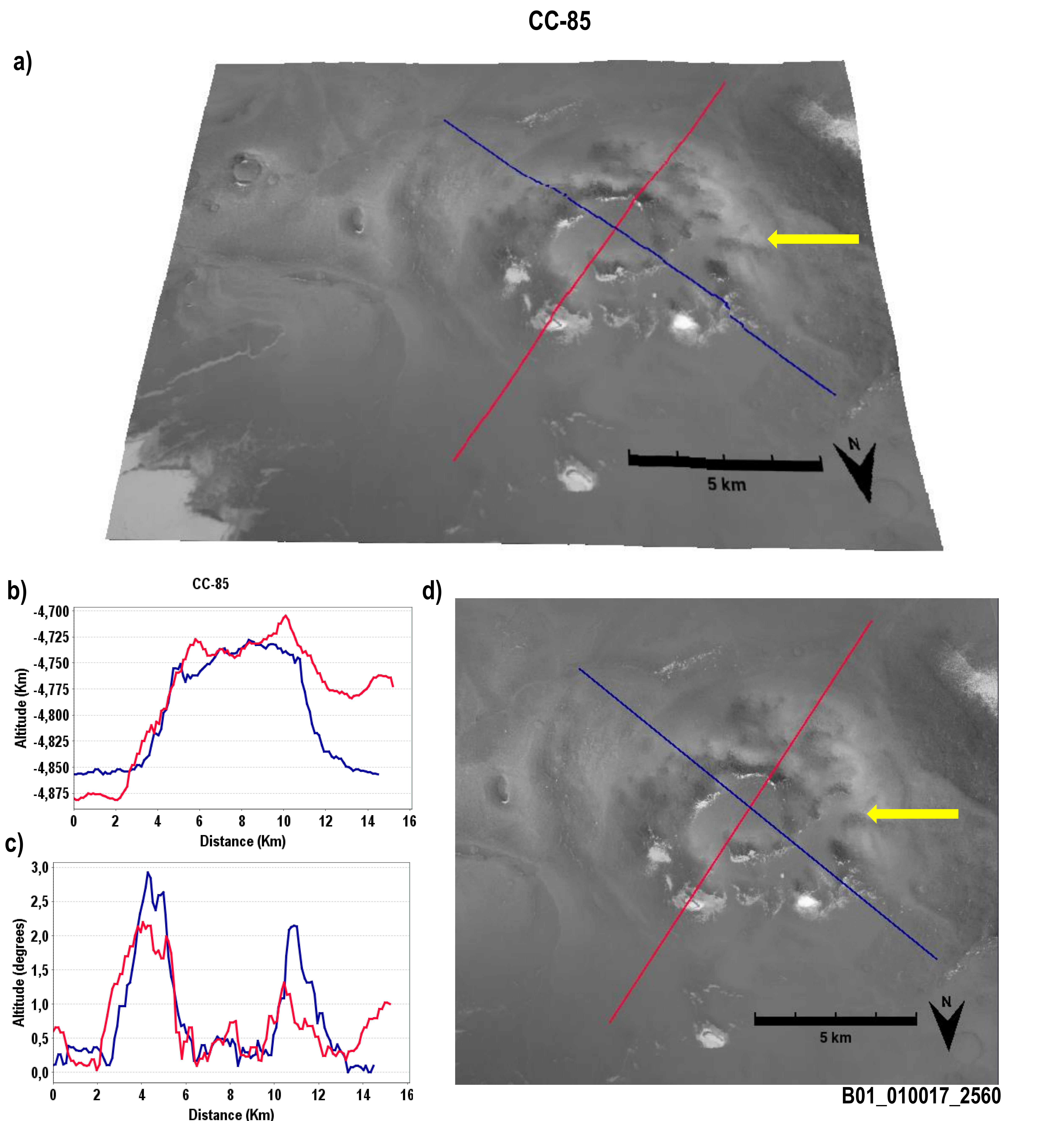
CC-85
a)
5 km
N
b)
CC-85
Altitude (Km)
-4,700
-4,725
-4,750
-4,775
-4,800
-4,825
-4,850
-4,875
0 2 4 6 8 10 12 14 16
Distance (Km)
c)
Altitude (degrees)
3,0
2,5
2,0
1,5
1,0
0,5
0,0
0 2 4 6 8 10 12 14 16
Distance (Km)
d)
5 km
N
B01_010017_2560

CC-86

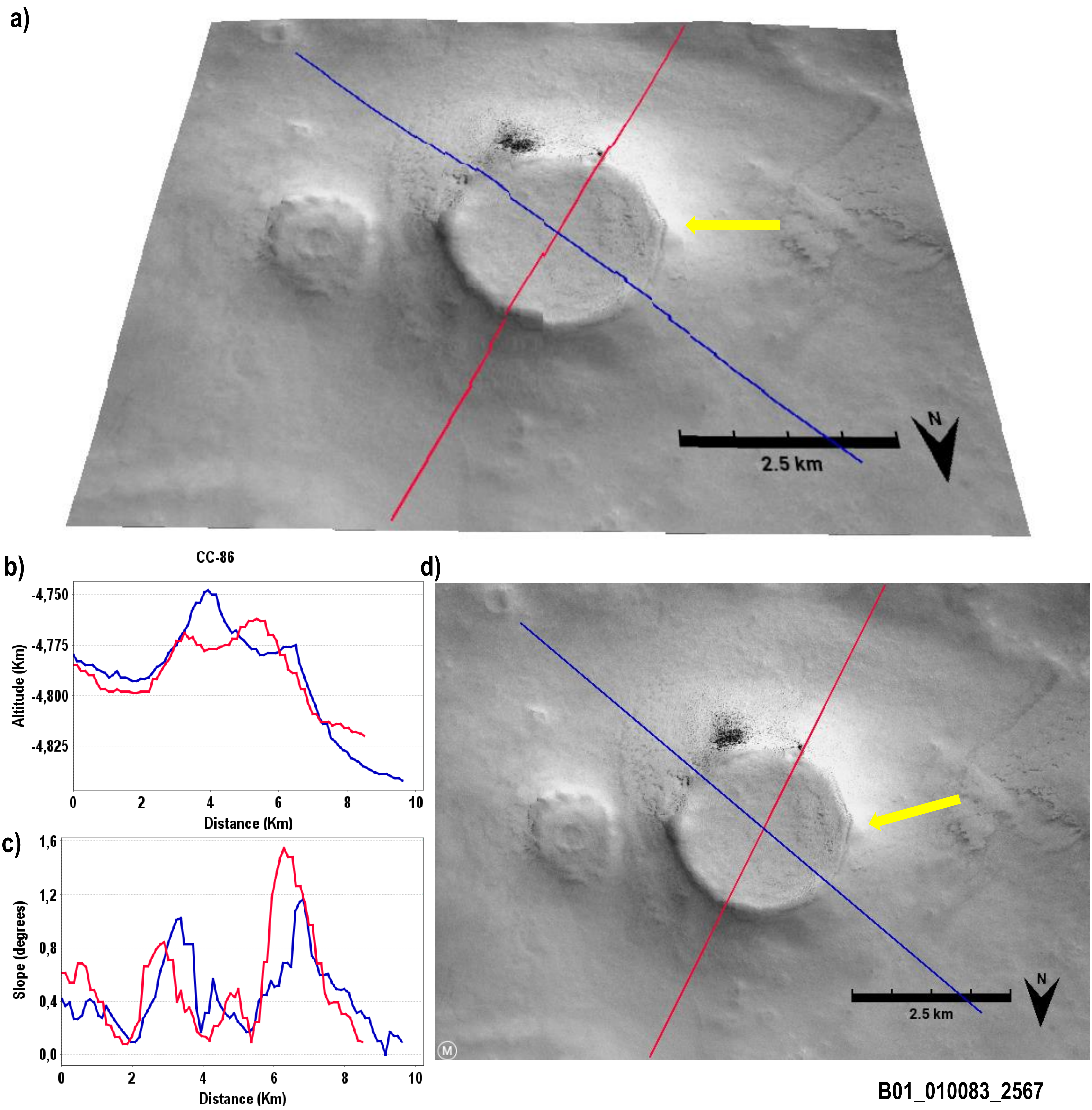

B01_010083_2567

CC-87

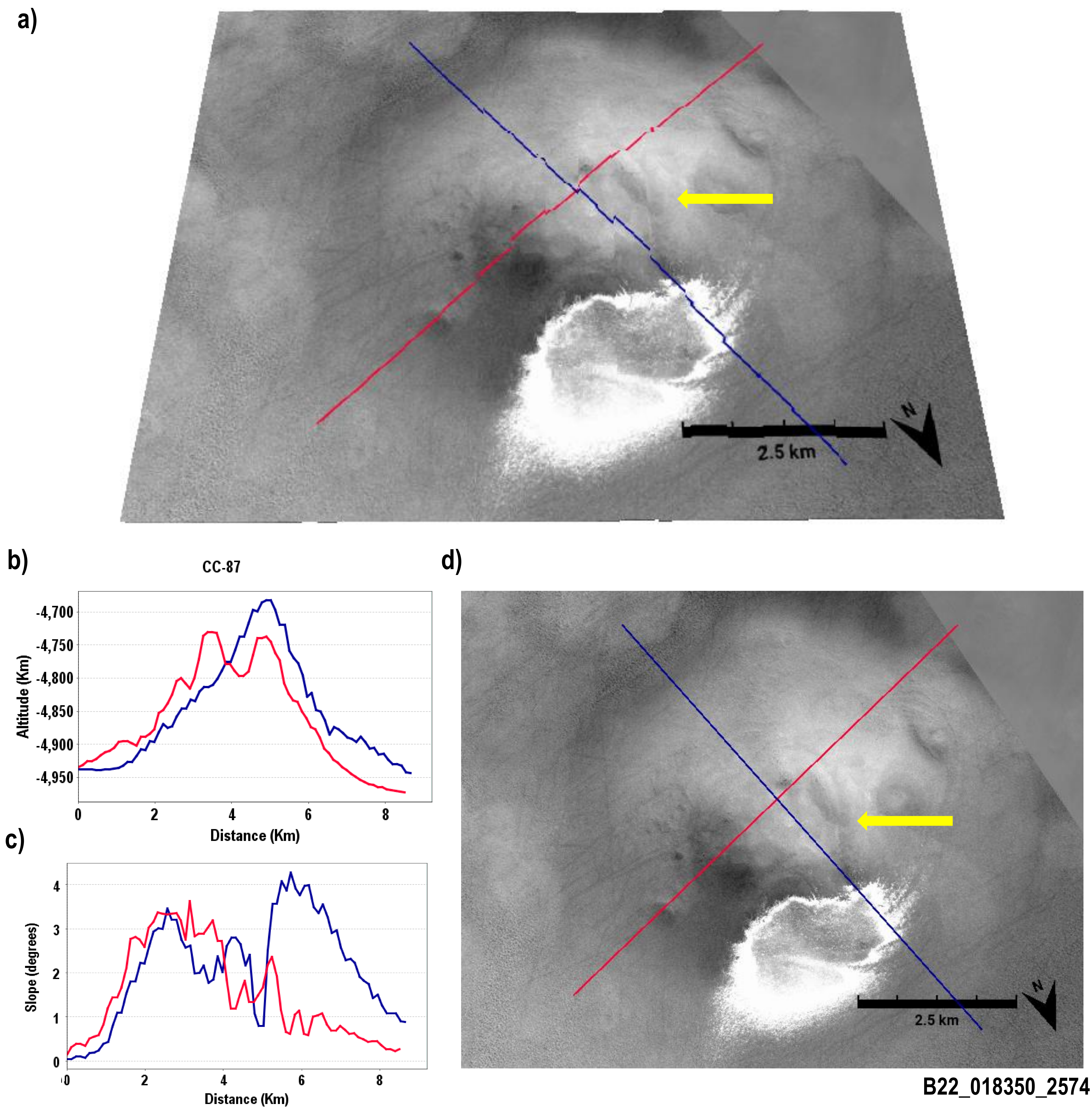

B22_018350_2574

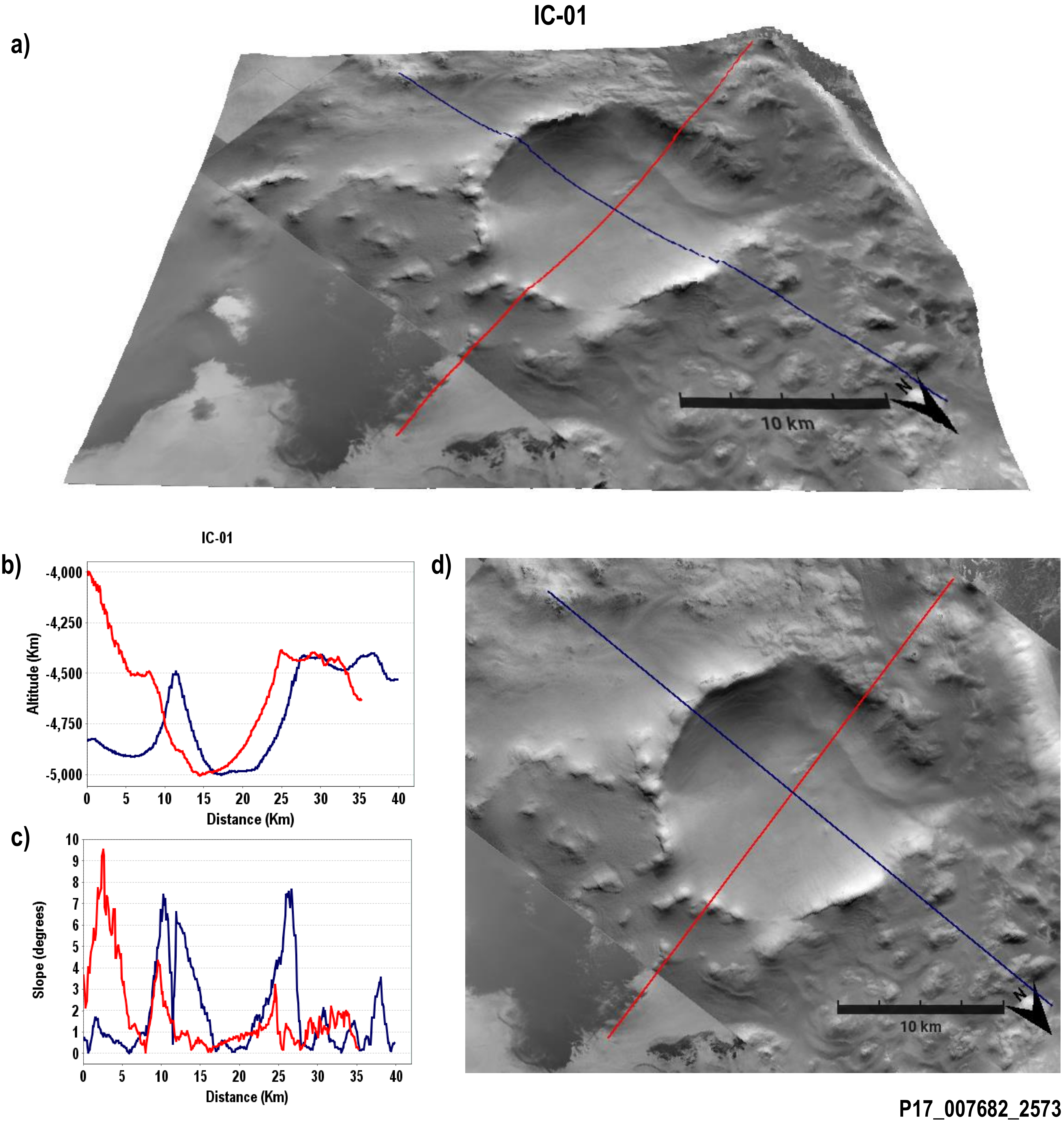
IC-01
a)
10 km
N
IC-01
b)
Altitude (Km)
-4,000
-4,250
-4,500
-4,750
-5,000
0 5 10 15 20 25 30 35 40
Distance (Km)
c)
Slope (degrees)
10 9 8 7 6 5 4 3 2 1 0
0 5 10 15 20 25 30 35 40
Distance (Km)
d)
10 km
N
P17_007682_2573

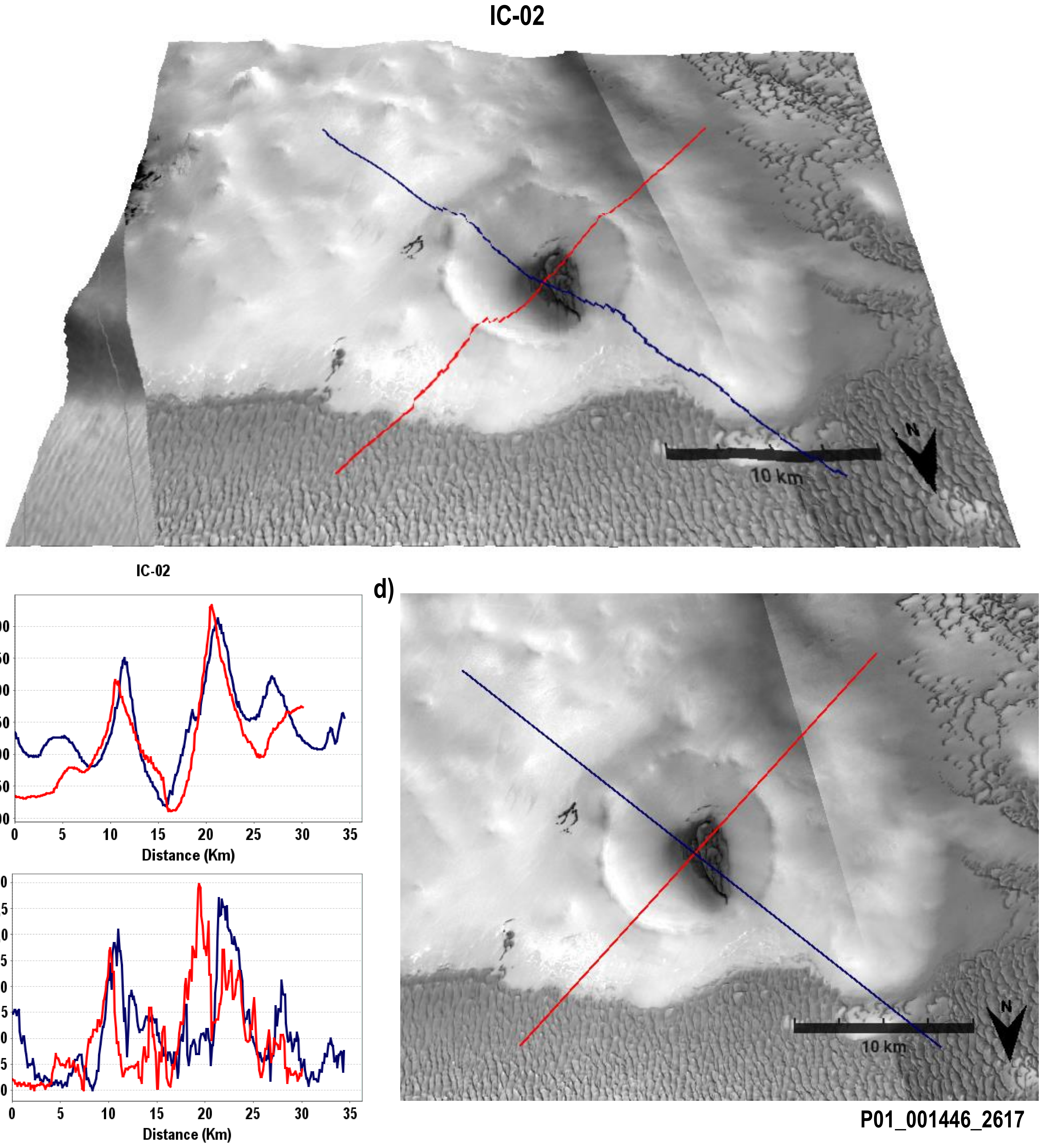

IC-02
a)
10 km
N
IC-02
b)
Altitude (Km)
-4,600
-4,650
-4,700
-4,750
-4,800
-4,850
-4,900
0 5 10 15 20 25 30 35
Distance (Km)
c)
Slope (degrees)
4,0
3,5
3,0
2,5
2,0
1,5
1,0
0,5
0,0
0 5 10 15 20 25 30 35
Distance (Km)
d)
10 km
N
P01_001446_2617

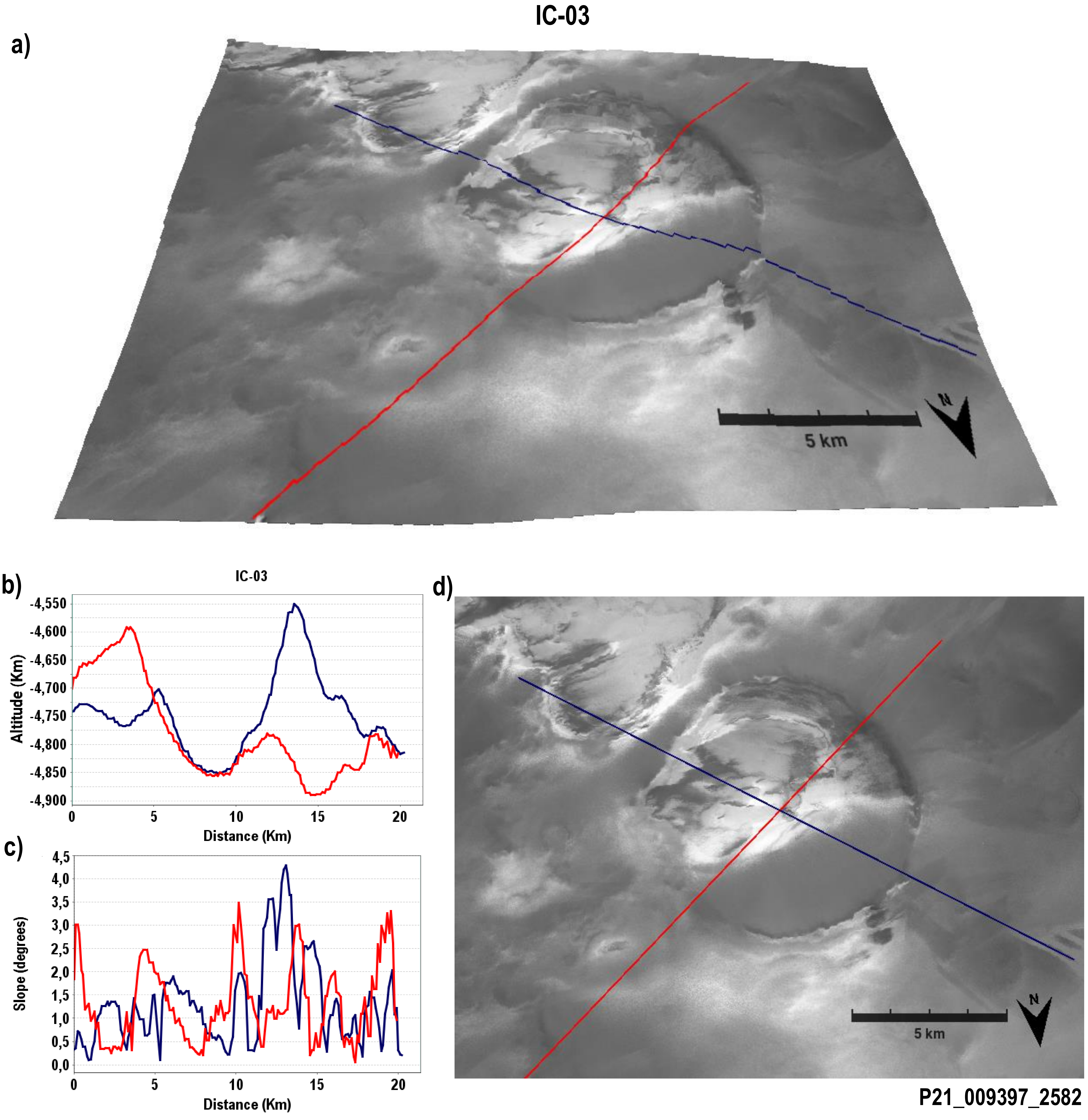
IC-03
a)
5 km
N
b)
IC-03
Altitude (Km)
-4,550
-4,600
-4,650
-4,700
-4,750
-4,800
-4,850
-4,900
0
5
10
15
20
Distance (Km)
c)
Slope (degrees)
4,5
4,0
3,5
3,0
2,5
2,0
1,5
1,0
0,5
0,0
Distance (Km)
d)
5 km
N
P21_009397_2582

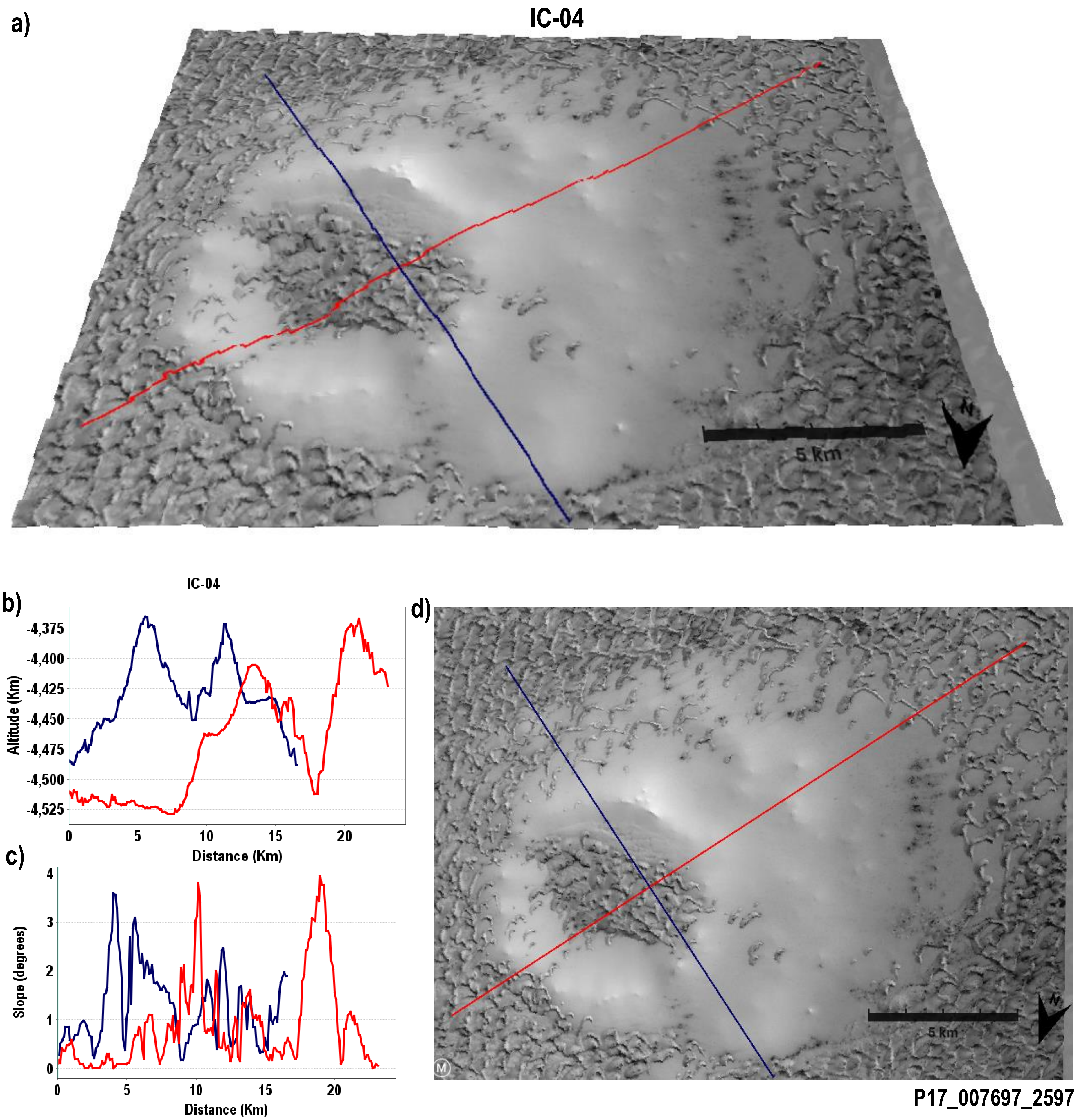
a)
IC-04
5 km
N
b)
IC-04
Altitude (Km)
-4,375
-4,400
-4,425
-4,450
-4,475
-4,500
-4,525
0
5
10
15
20
Distance (Km)
c)
Slope (degrees)
4
3
2
1
0
0
5
10
15
20
Distance (Km)
d)
5 km
N
P17_007697_2597

IC-05

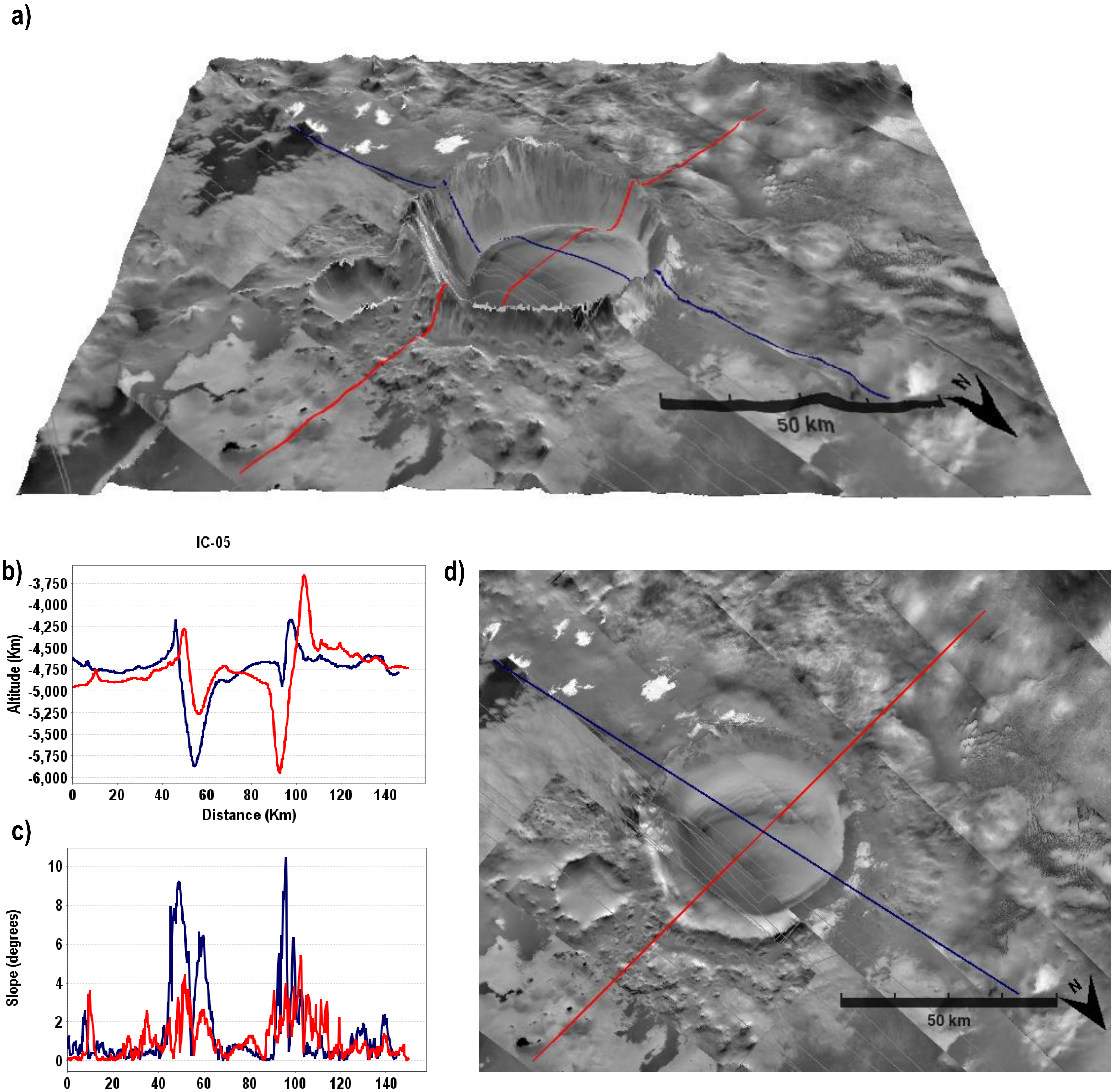

B02_010240_2575 / B01_010108_2573 / B02_010385_2572

IC-06

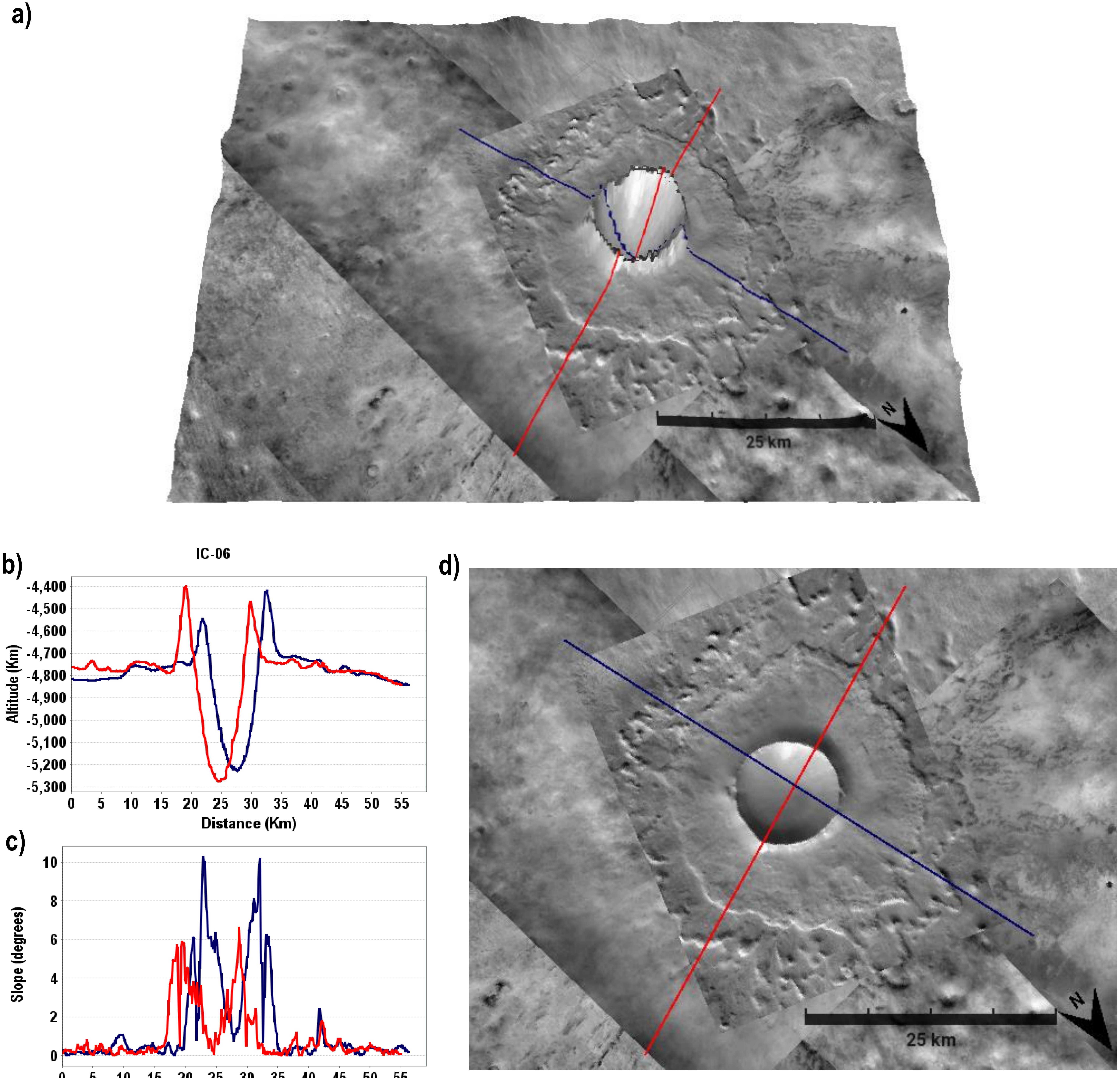

B21_018014_2858

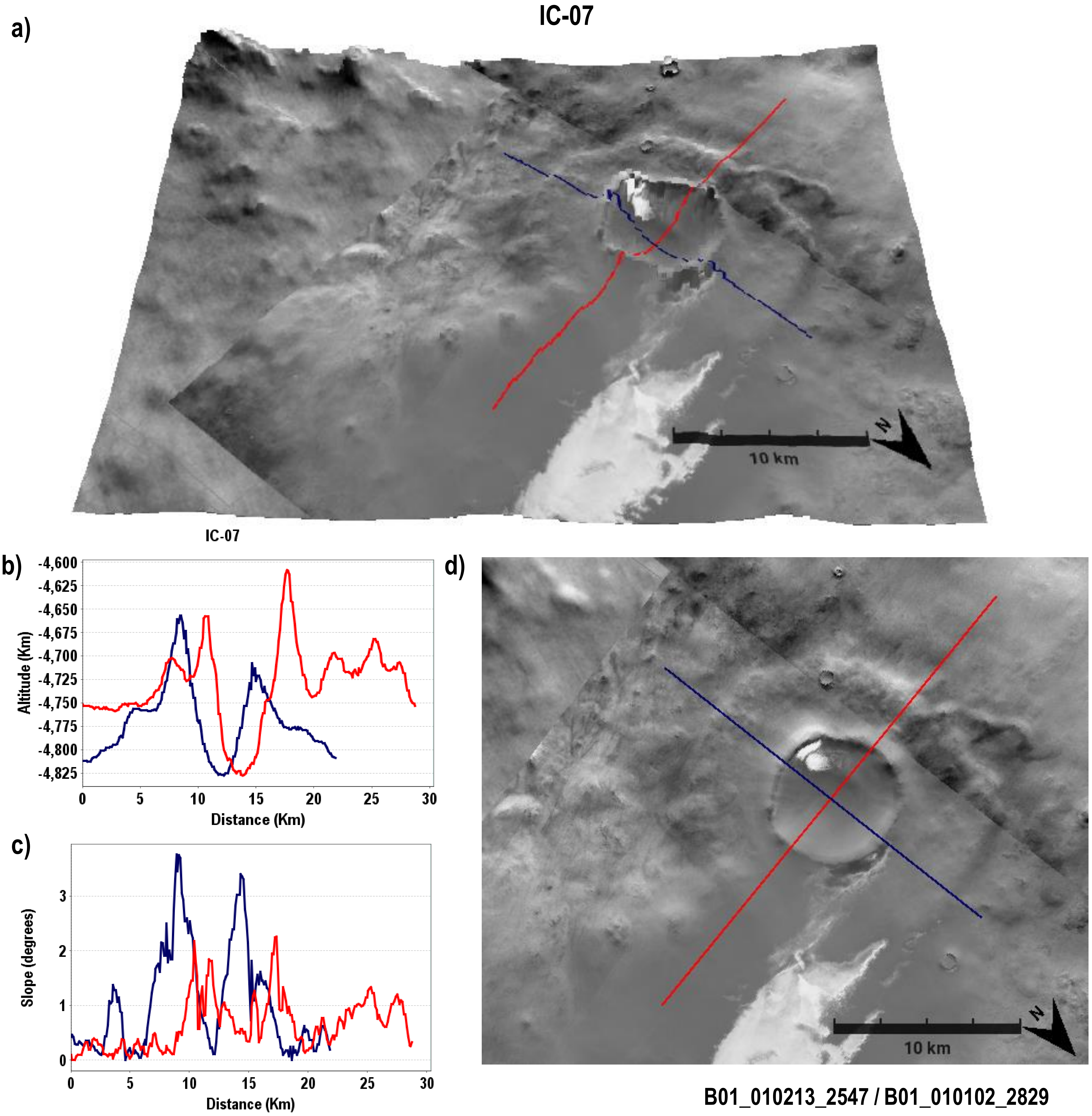
a)
IC-07
10 km
N
IC-07
b)
Altitude (Km)
-4,600
-4,625
-4,650
-4,675
-4,700
-4,725
-4,750
-4,775
-4,800
-4,825
0
5
10
15
20
25
30
Distance (Km)
c)
Slope (degrees)
0
1
2
3
Distance (Km)
d)
10 km
N
B01_010213_2547 / B01_010102_2829

IC-08

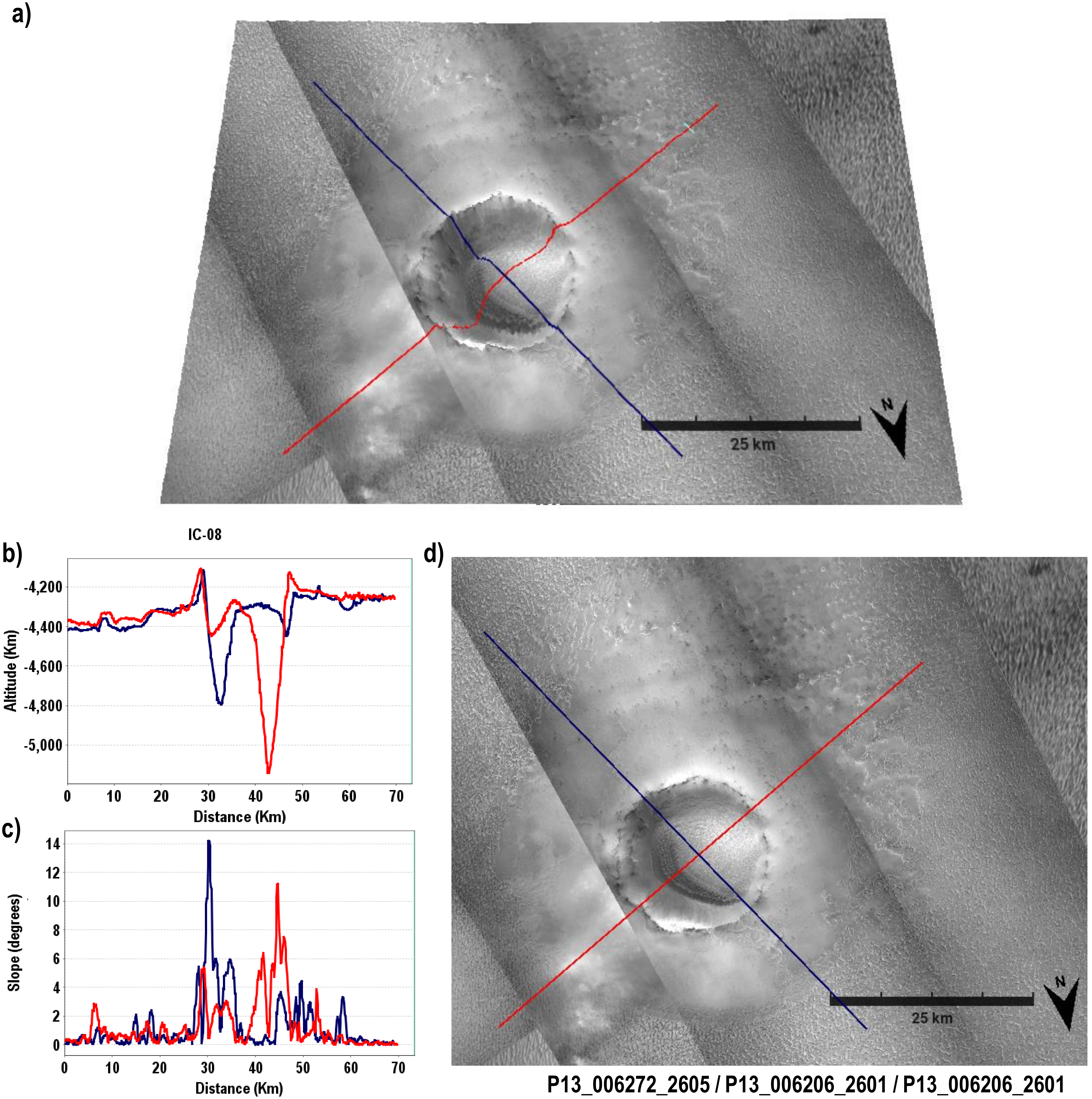
a)
25 km
N
b)
IC-08
Altitude (Km)
-4,200
-4,400
-4,600
-4,800
-5,000
0 10 20 30 40 50 60 70
Distance (Km)
c)
Slope (degrees)
14
12
10
8
6
4
2
0
0 10 20 30 40 50 60 70
Distance (Km)
d)
25 km
N
P13_006272_2605 / P13_006206_2601 / P13_006206_2601

IC-09

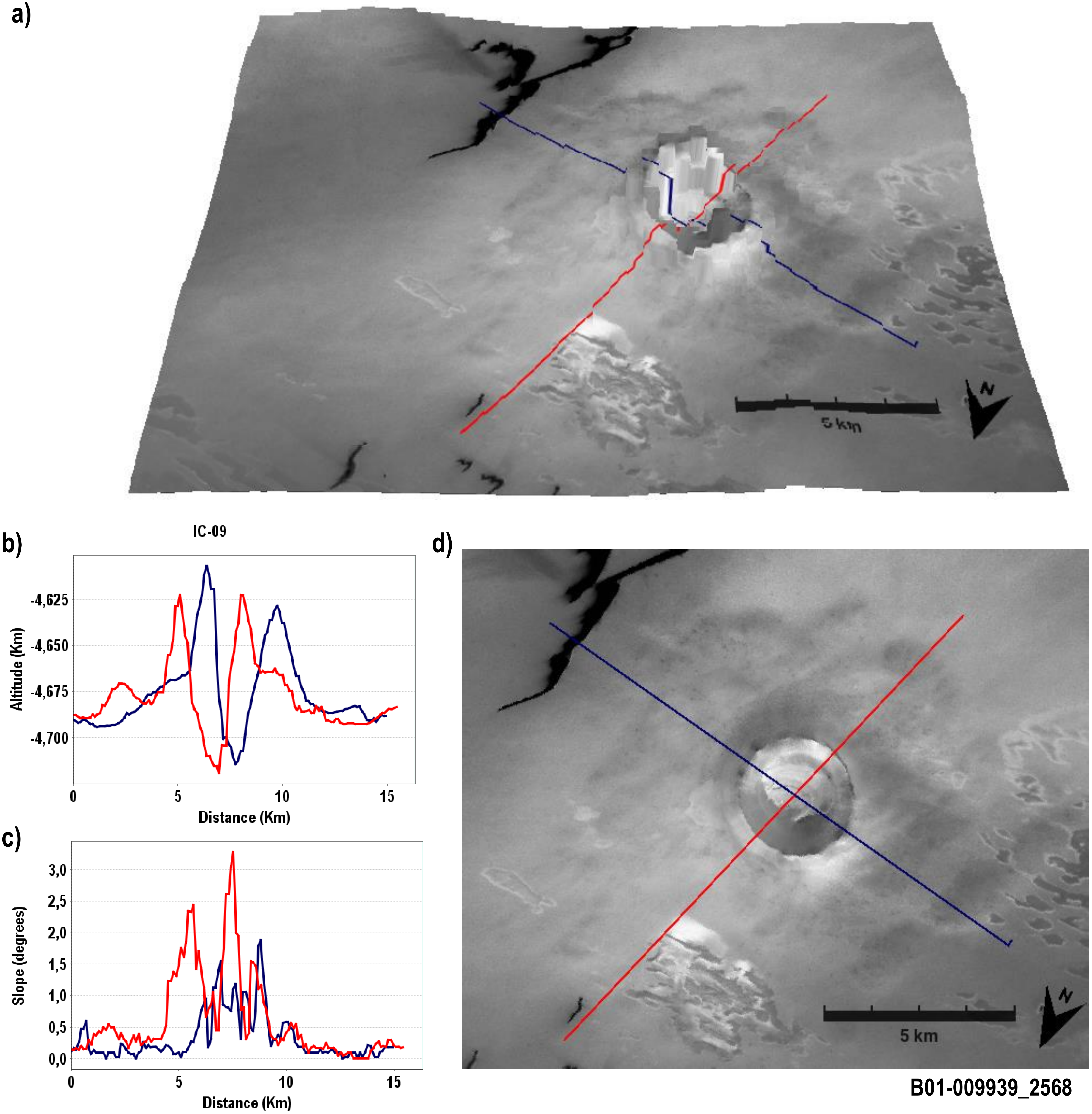

B01-009939_2568

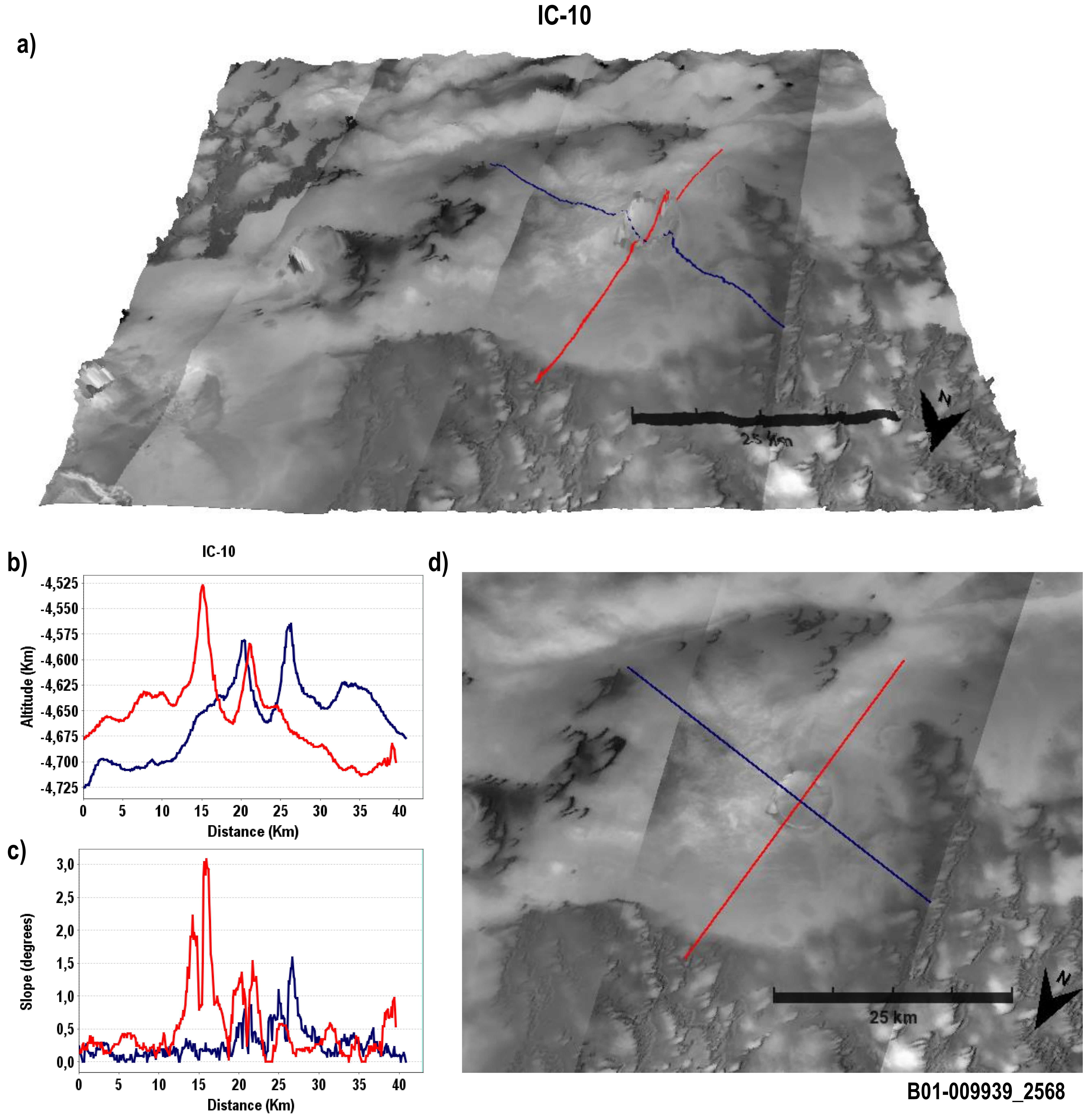

IC-10
a)
25 Km
N
b)
IC-10
Altitude (Km)
-4,525
-4,550
-4,575
-4,600
-4,625
-4,650
-4,675
-4,700
-4,725
0 5 10 15 20 25 30 35 40
Distance (Km)
c)
Slope (degrees)
3,0
2,5
2,0
1,5
1,0
0,5
0,0
0 5 10 15 20 25 30 35 40
Distance (Km)
d)
25 km
N
B01-009939_2568

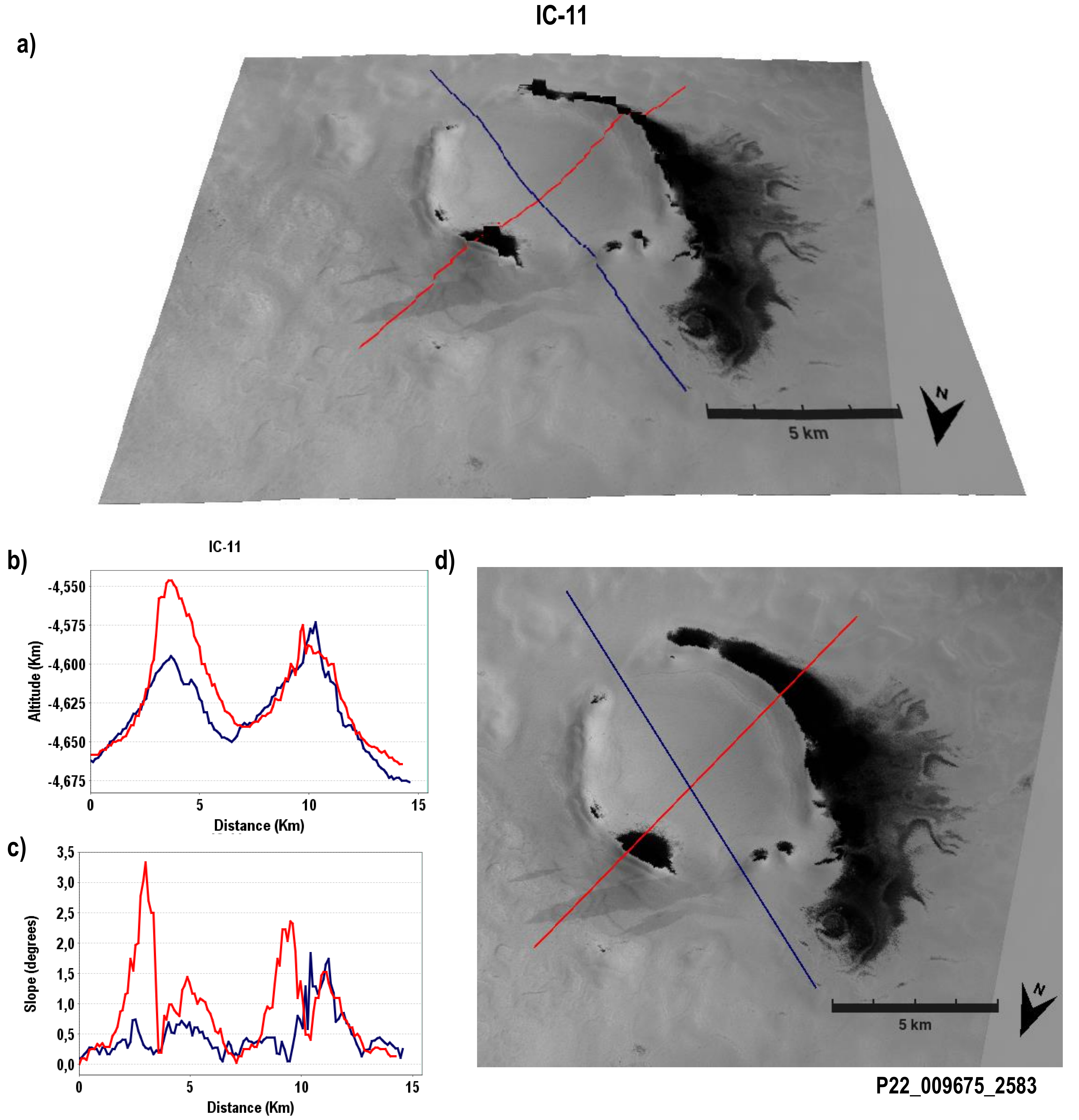

IC-11
a)
5 km
N
b)
IC-11
Altitude (Km)
-4,550
-4,575
-4,600
-4,625
-4,650
-4,675
0
5
10
15
Distance (Km)
c)
Slope (degrees)
3,5
3,0
2,5
2,0
1,5
1,0
0,5
0,0
0
5
10
15
Distance (Km)
d)
5 km
N
P22_009675_2583

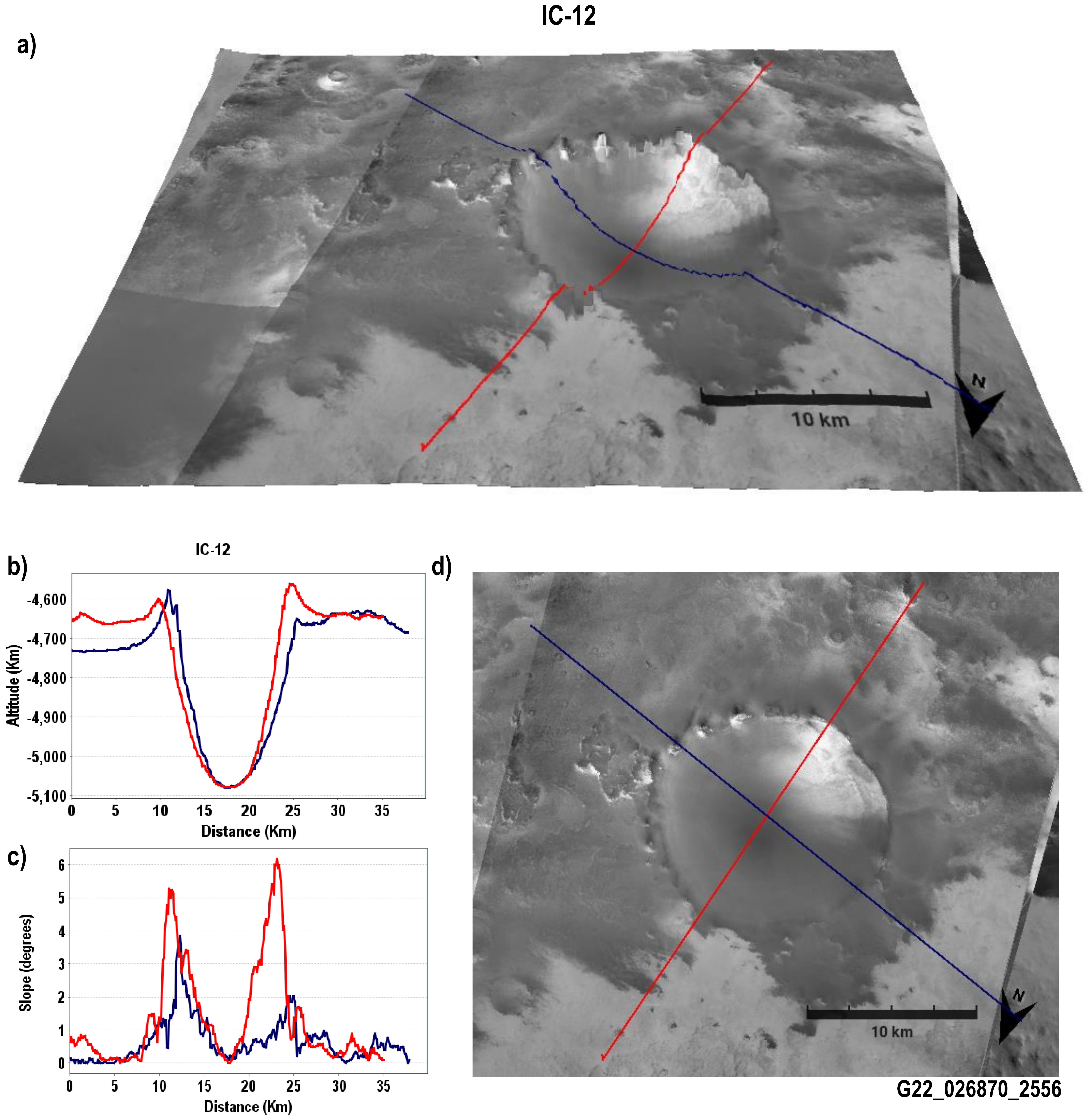
IC-12
a)
10 km
N
b)
IC-12
Altitude (Km)
-4,600
-4,700
-4,800
-4,900
-5,000
-5,100
0
5
10
15
20
25
30
35
Distance (Km)
c)
Slope (degrees)
6
5
4
3
2
1
0
Distance (Km)
d)
10 km
N
G22_026870_2556

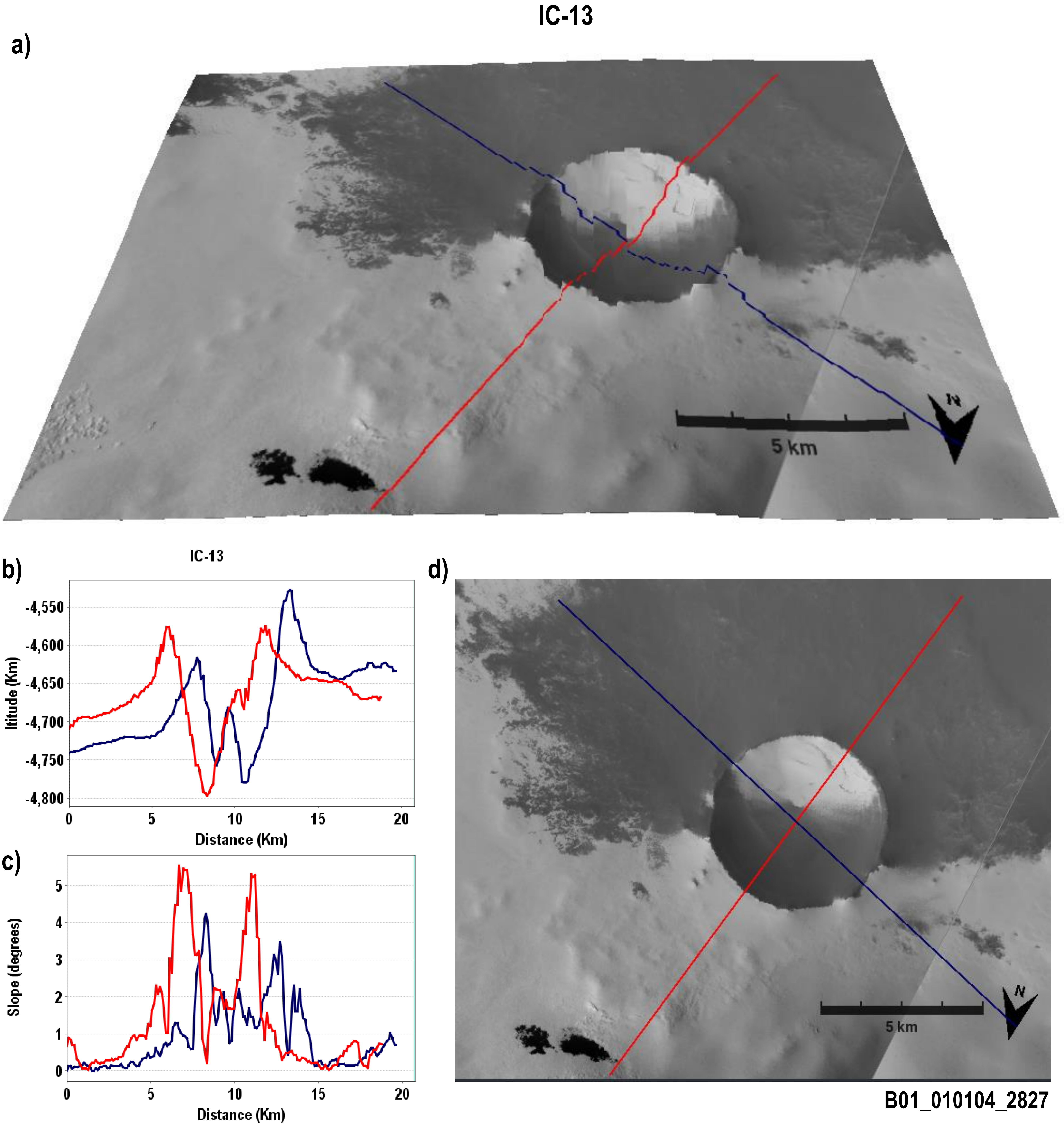
IC-13
a)
5 km
N
b)
IC-13
ltitude (Km)
-4,550
-4,600
-4,650
-4,700
-4,750
-4,800
0
5
10
15
20
Distance (Km)
c)
Slope (degrees)
5
4
3
2
1
0
0
5
10
15
20
Distance (Km)
d)
5 km
N
B01_010104_2827

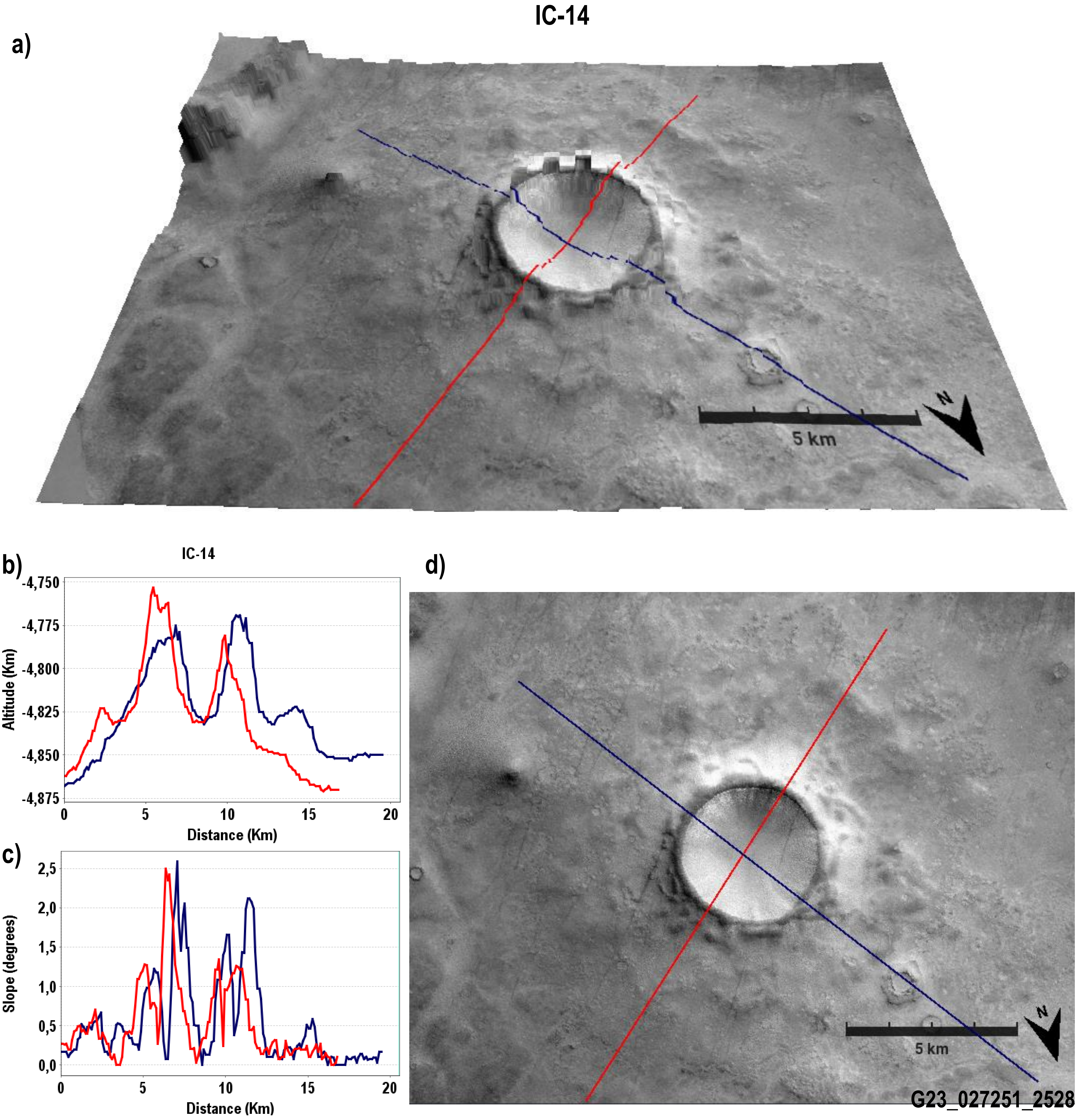

IC-14
a)
5 km
N
b)
IC-14
Altitude (Km)
-4,750
-4,775
-4,800
-4,825
-4,850
-4,875
0
5
10
15
20
Distance (Km)
c)
Slope (degrees)
2,5
2,0
1,5
1,0
0,5
0,0
Distance (Km)
d)
5 km
N
G23_027251_2528

IC-15

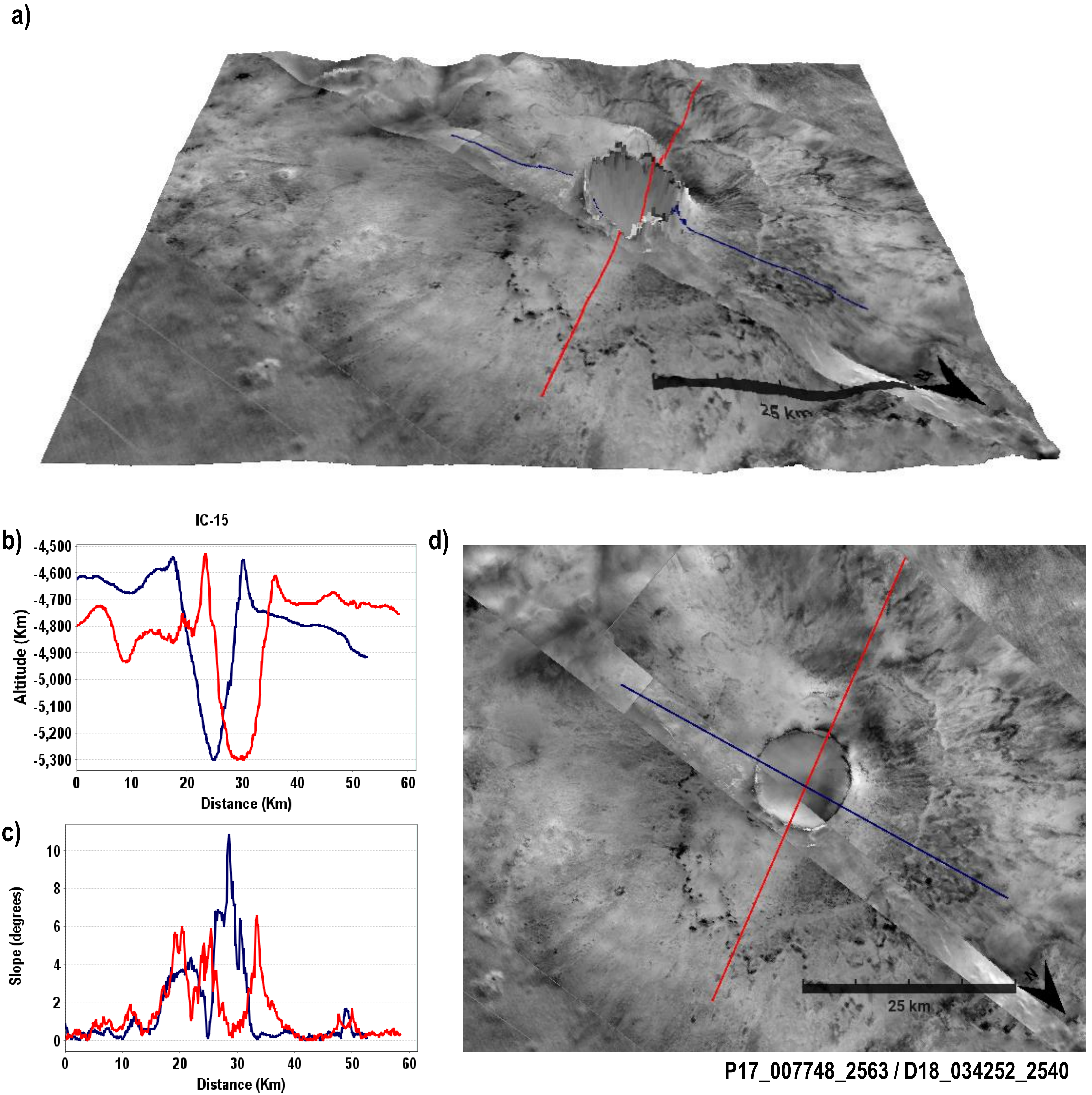

P17_007748_2563 / D18_034252_2540

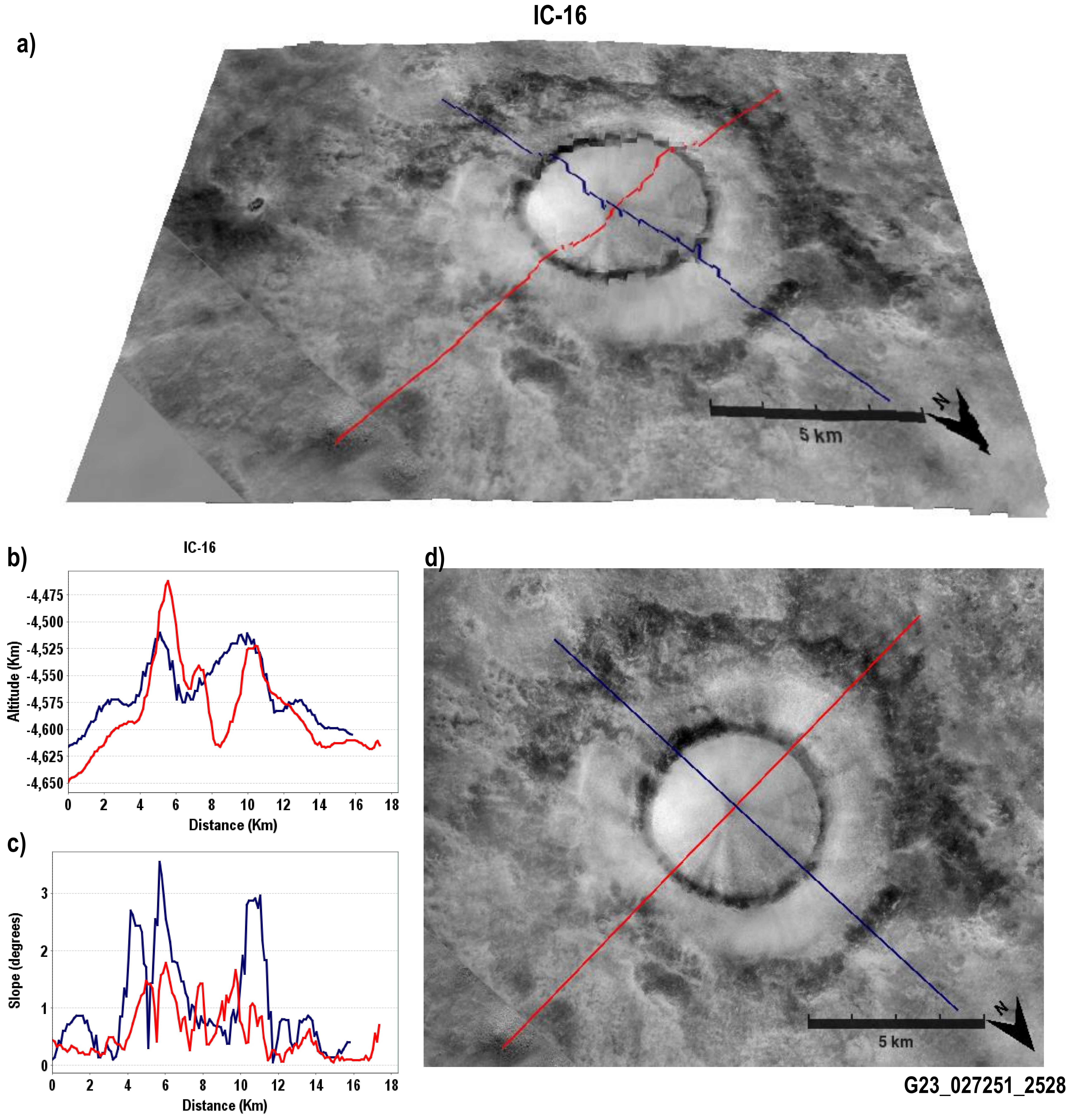

IC-16
a)
5 km
N
b)
IC-16
Altitude (Km)
-4,475
-4,500
-4,525
-4,550
-4,575
-4,600
-4,625
-4,650
0
2
4
6
8
10
12
14
16
18
Distance (Km)
c)
Slope (degrees)
3
2
1
0
Distance (Km)
d)
5 km
N
G23_027251_2528

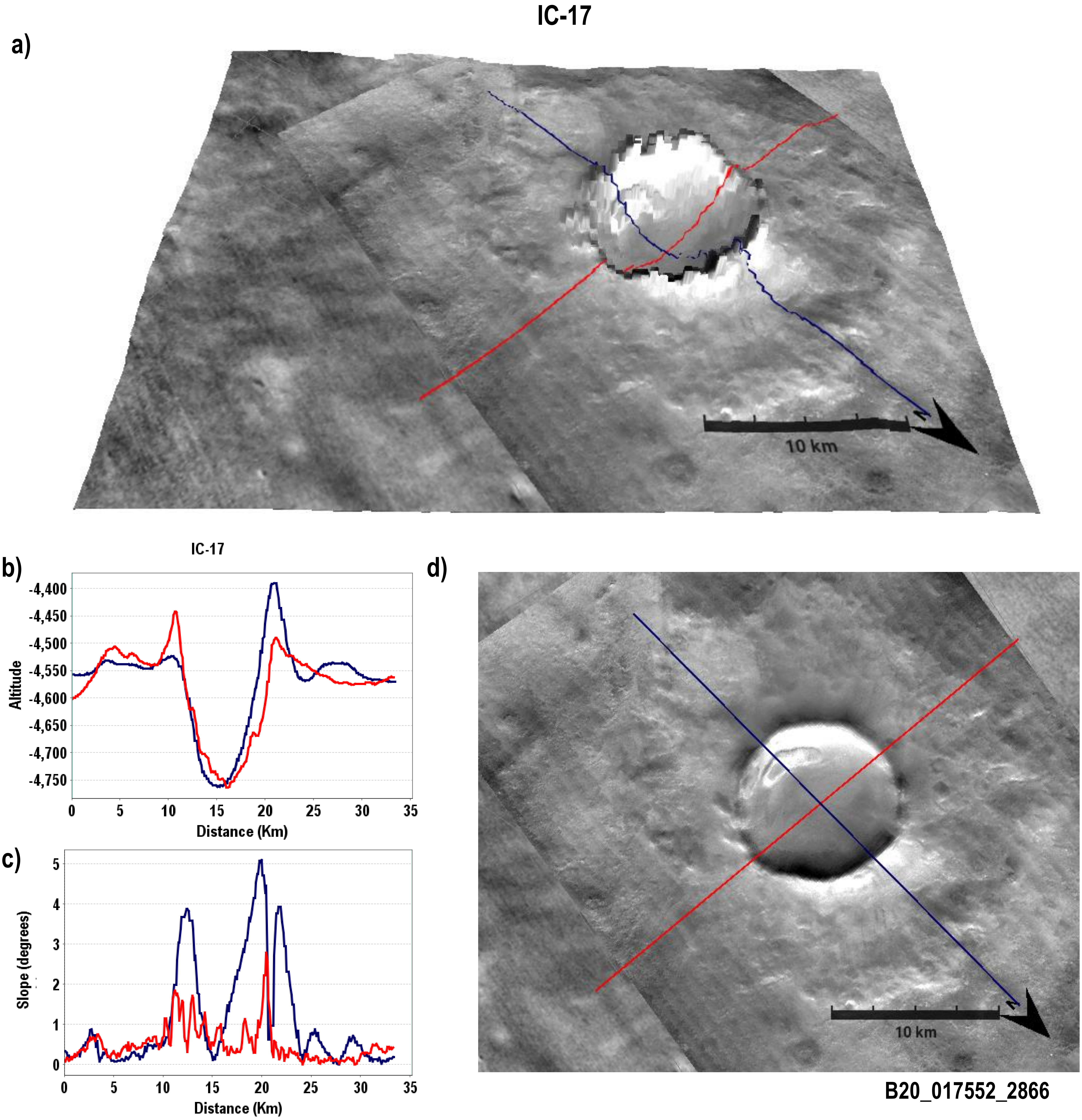
IC-17
a)
10 km
IC-17
b)
-4,400
-4,450
-4,500
-4,550
-4,600
-4,650
-4,700
-4,750
Altitude
0
5
10
15
20
25
30
35
Distance (Km)
c)
5
4
3
2
1
0
Slope (degrees)
Distance (Km)
d)
10 km
B20_017552_2866

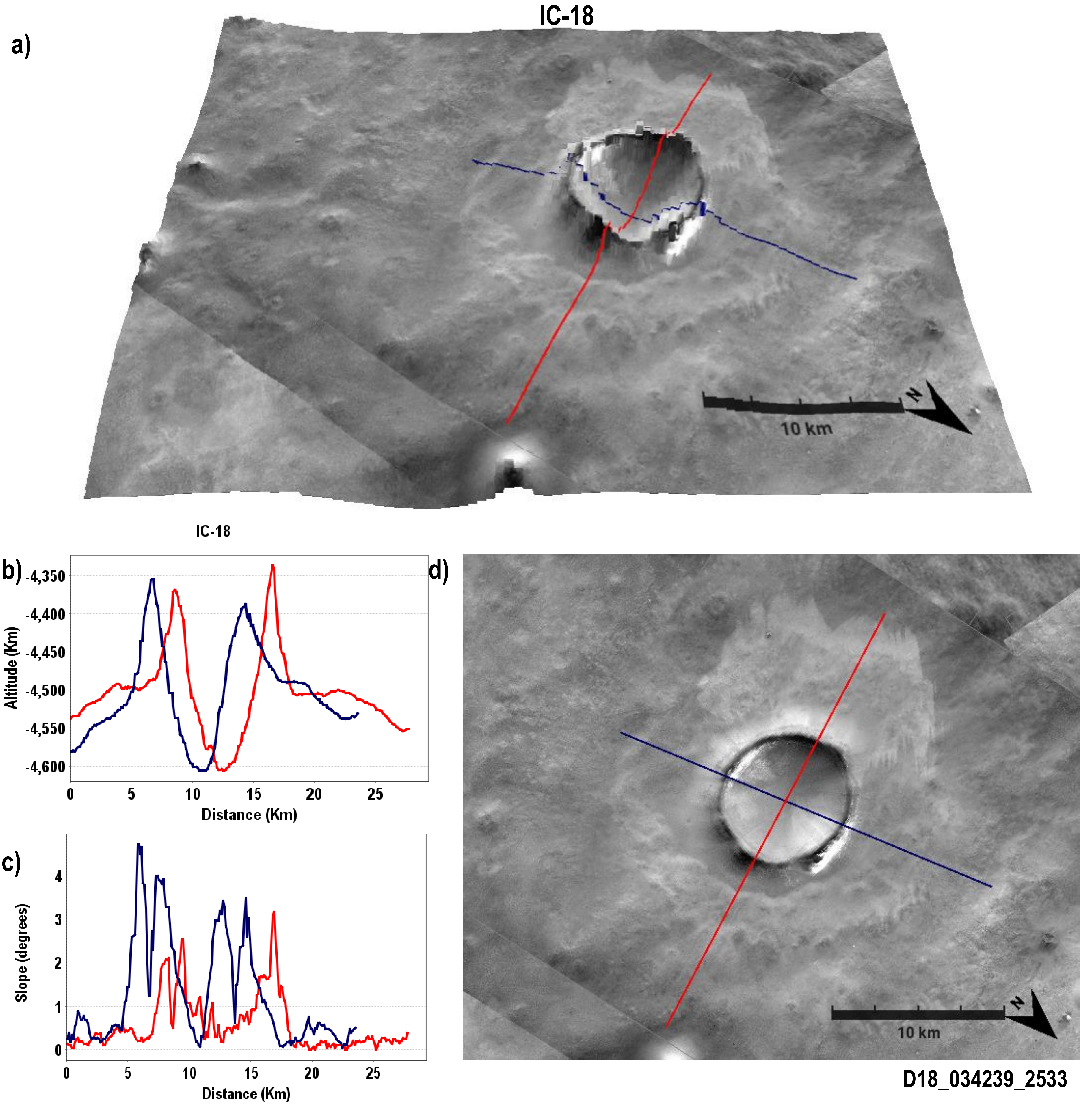

IC-18
a)
10 km
N
IC-18
b)
-4,350
-4,400
-4,450
-4,500
-4,550
-4,600
Altitude (Km)
0
5
10
15
20
25
Distance (Km)
c)
4
3
2
1
0
Slope (degrees)
0
5
10
15
20
25
Distance (Km)
d)
10 km
N
D18_034239_2533

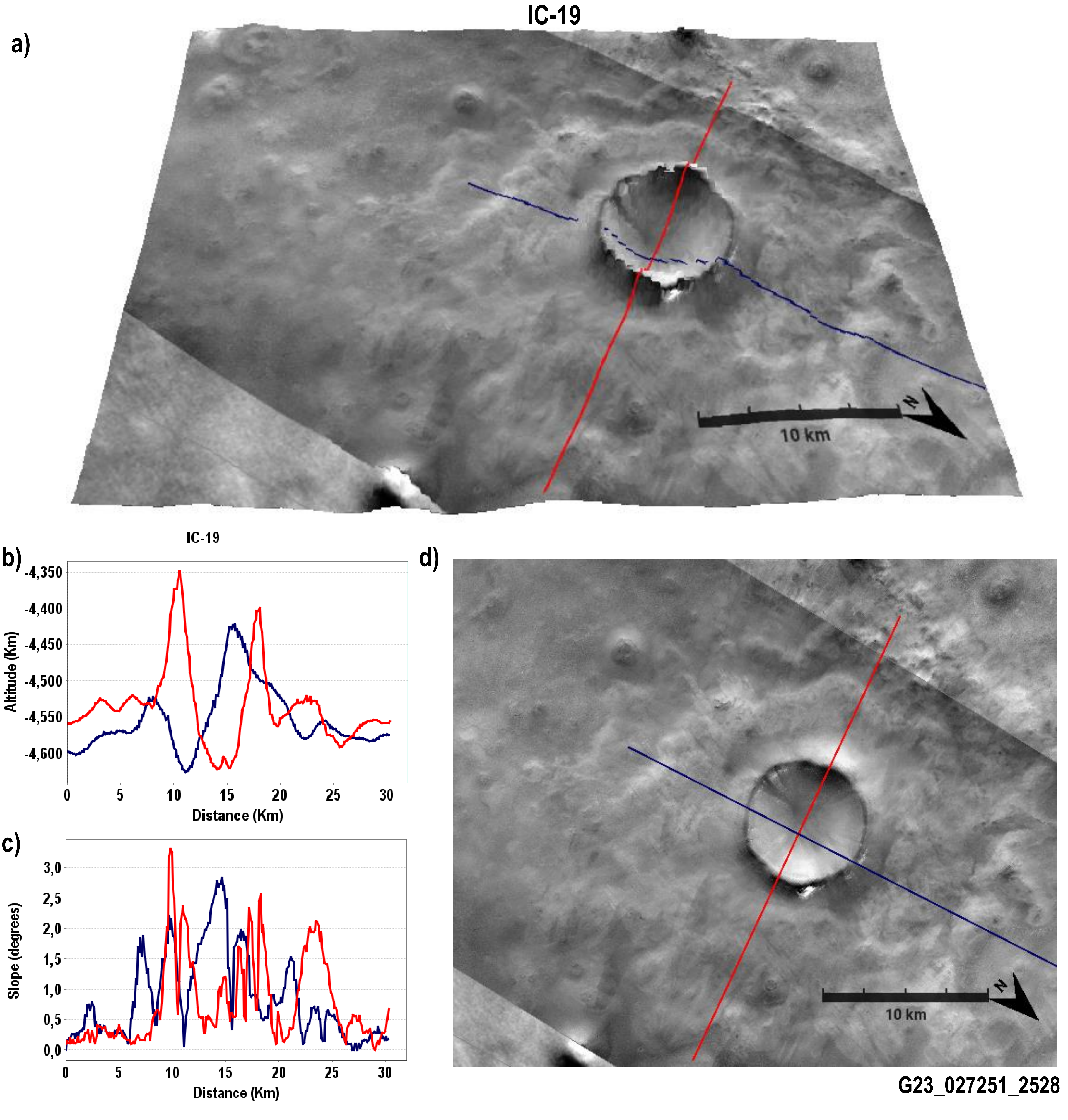
IC-19
a)
10 km
N
IC-19
b)
-4,350
-4,400
-4,450
-4,500
-4,550
-4,600
Altitude (Km)
0
5
10
15
20
25
30
Distance (Km)
c)
3,0
2,5
2,0
1,5
1,0
0,5
0,0
Slope (degrees)
Distance (Km)
d)
10 km
N
G23_027251_2528

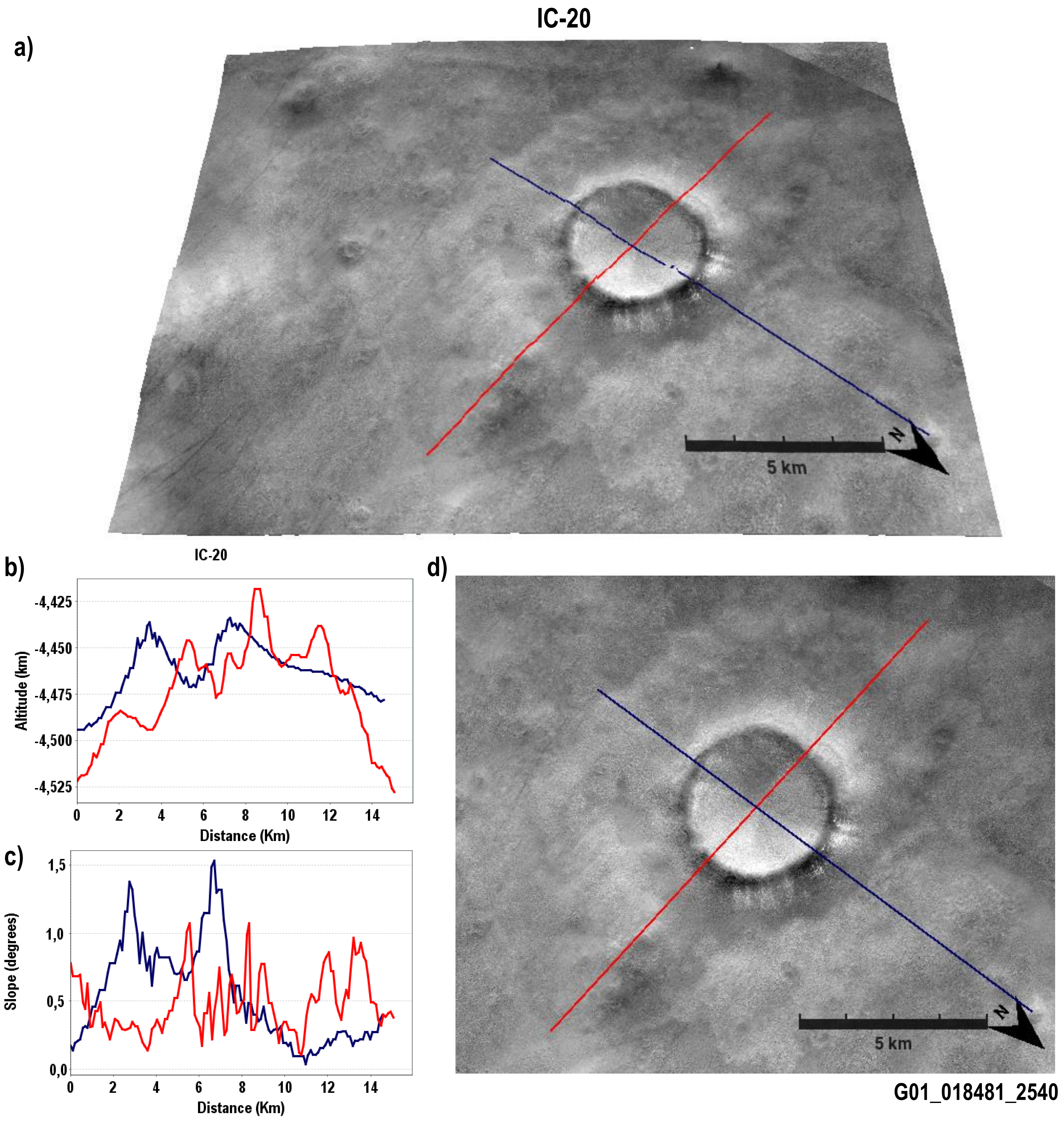
IC-20
a)
5 km
N
IC-20
b)
Altitude (km)
-4,425
-4,450
-4,475
-4,500
-4,525
Distance (Km)
c)
Slope (degrees)
1,5
1,0
0,5
0,0
Distance (Km)
d)
5 km
N
G01_018481_2540

IC-21

a)

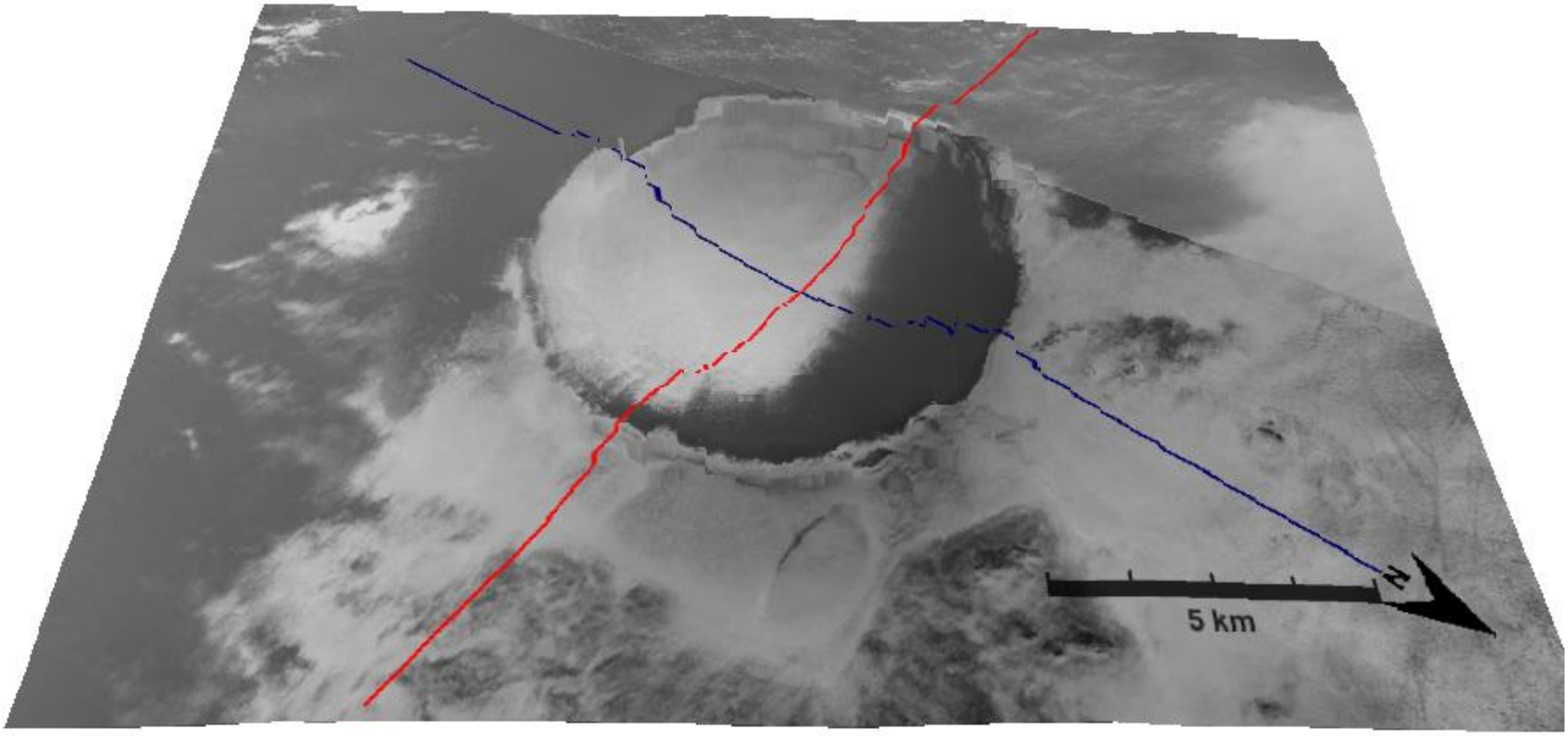

b)

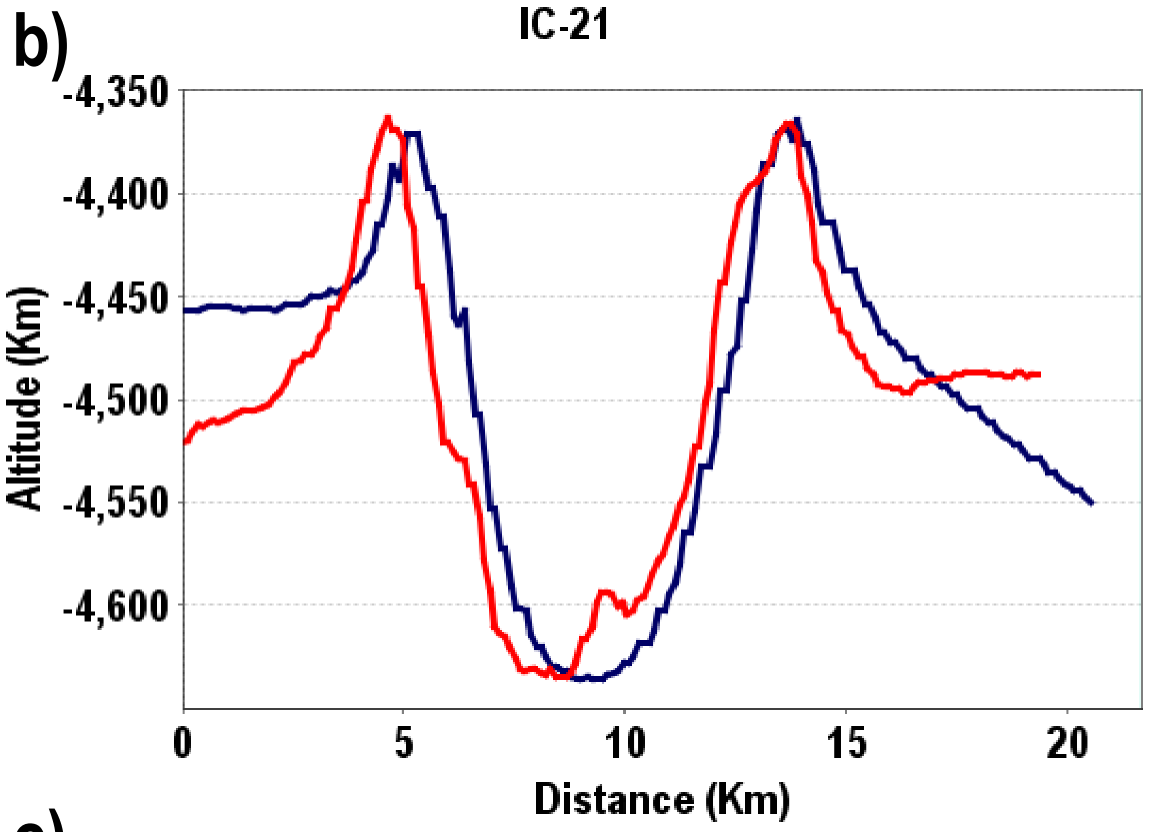

c)

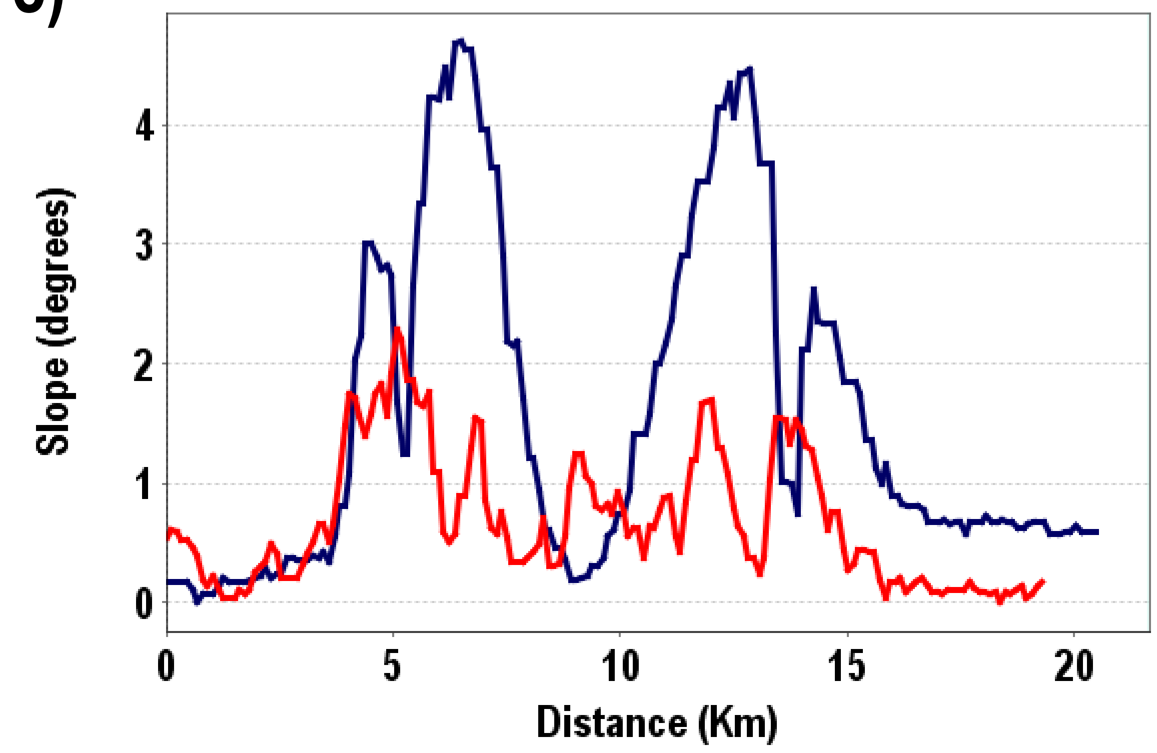

d)

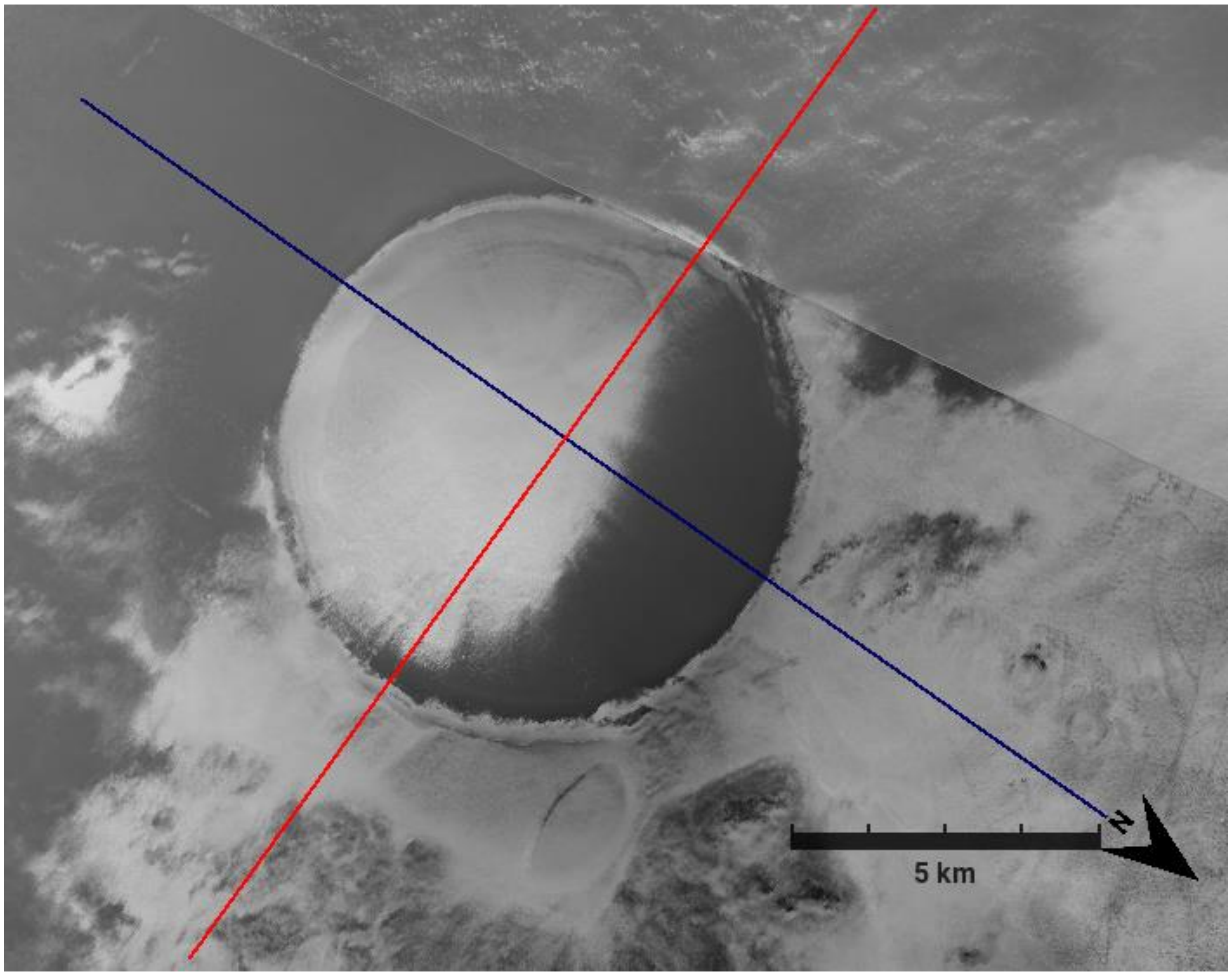

B01_010213_2547 / D19_034778_2525

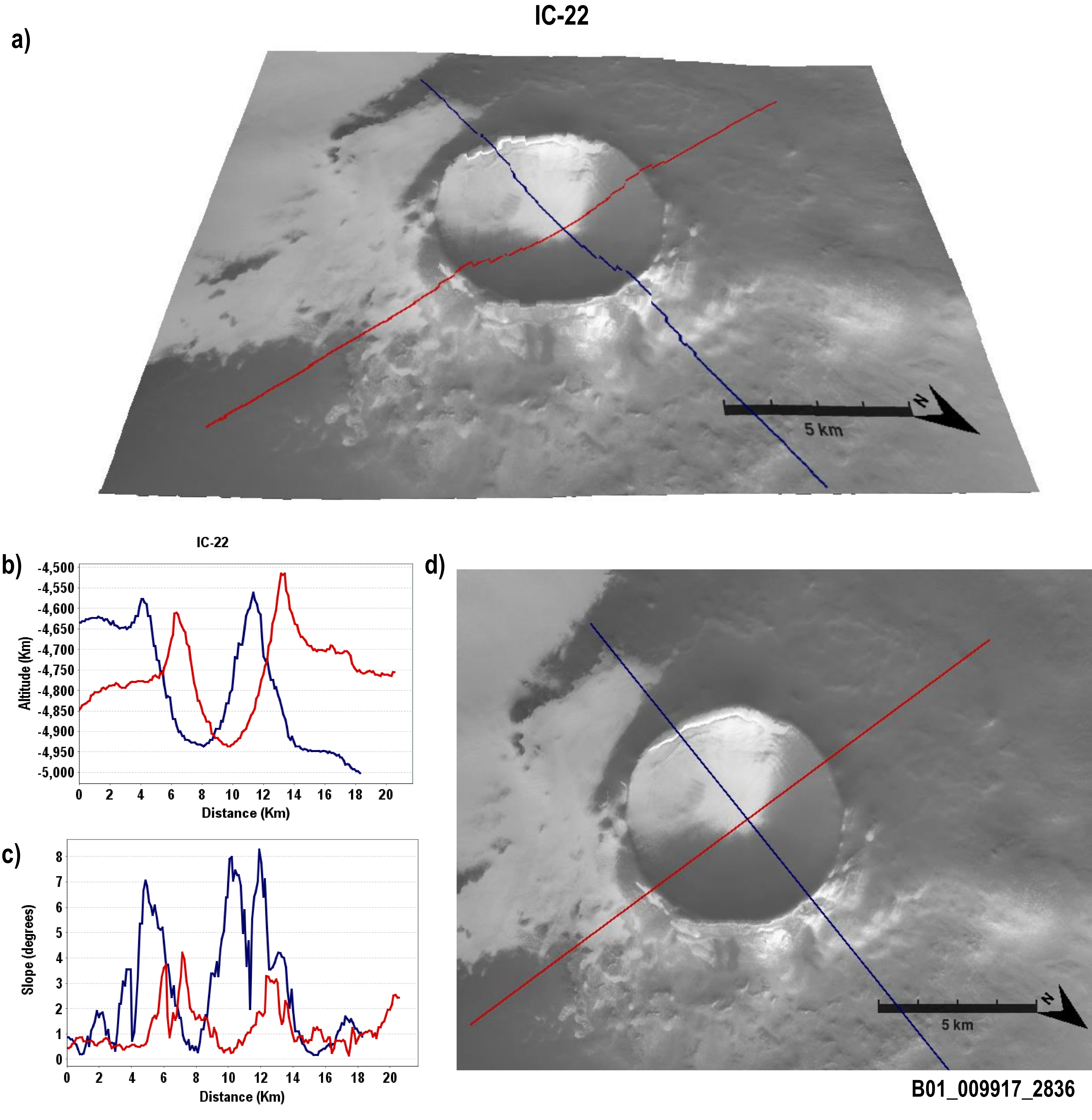

IC-22
a)
5 km
N
b)
IC-22
-4,500
-4,550
-4,600
-4,650
-4,700
-4,750
-4,800
-4,850
-4,900
-4,950
-5,000
Altitude (Km)
0 2 4 6 8 10 12 14 16 18 20
Distance (Km)
c)
8
7
6
5
4
3
2
1
0
Slope (degrees)
0 2 4 6 8 10 12 14 16 18 20
Distance (Km)
d)
5 km
N
B01_009917_2836

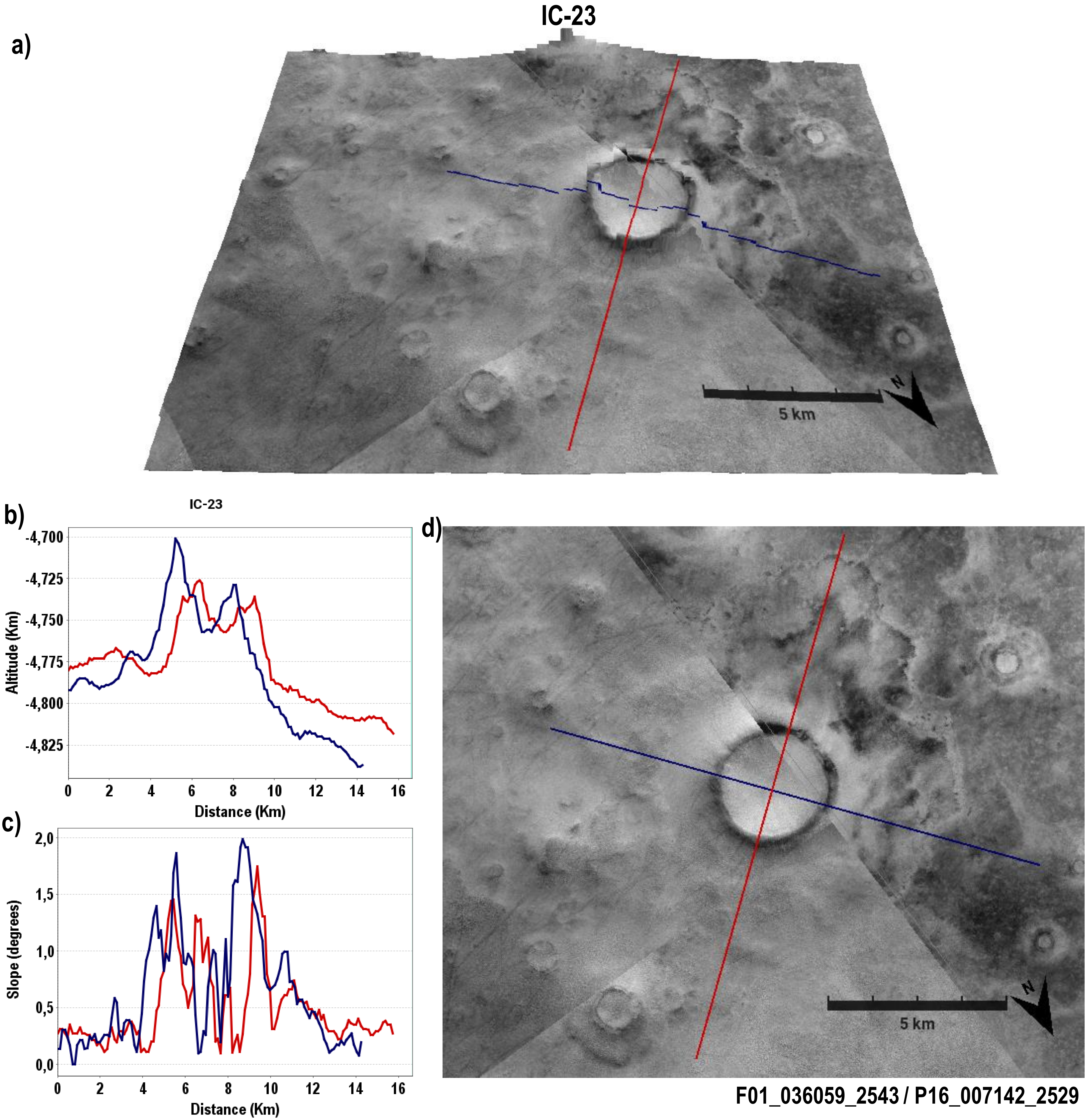
IC-23
a)
5 km
N
b)
IC-23
-4,700
-4,725
-4,750
-4,775
-4,800
-4,825
Altitude (Km)
0
2
4
6
8
10
12
14
16
Distance (Km)
c)
2,0
1,5
1,0
0,5
0,0
Slope (degrees)
Distance (Km)
d)
5 km
N
F01_036059_2543 / P16_007142_2529

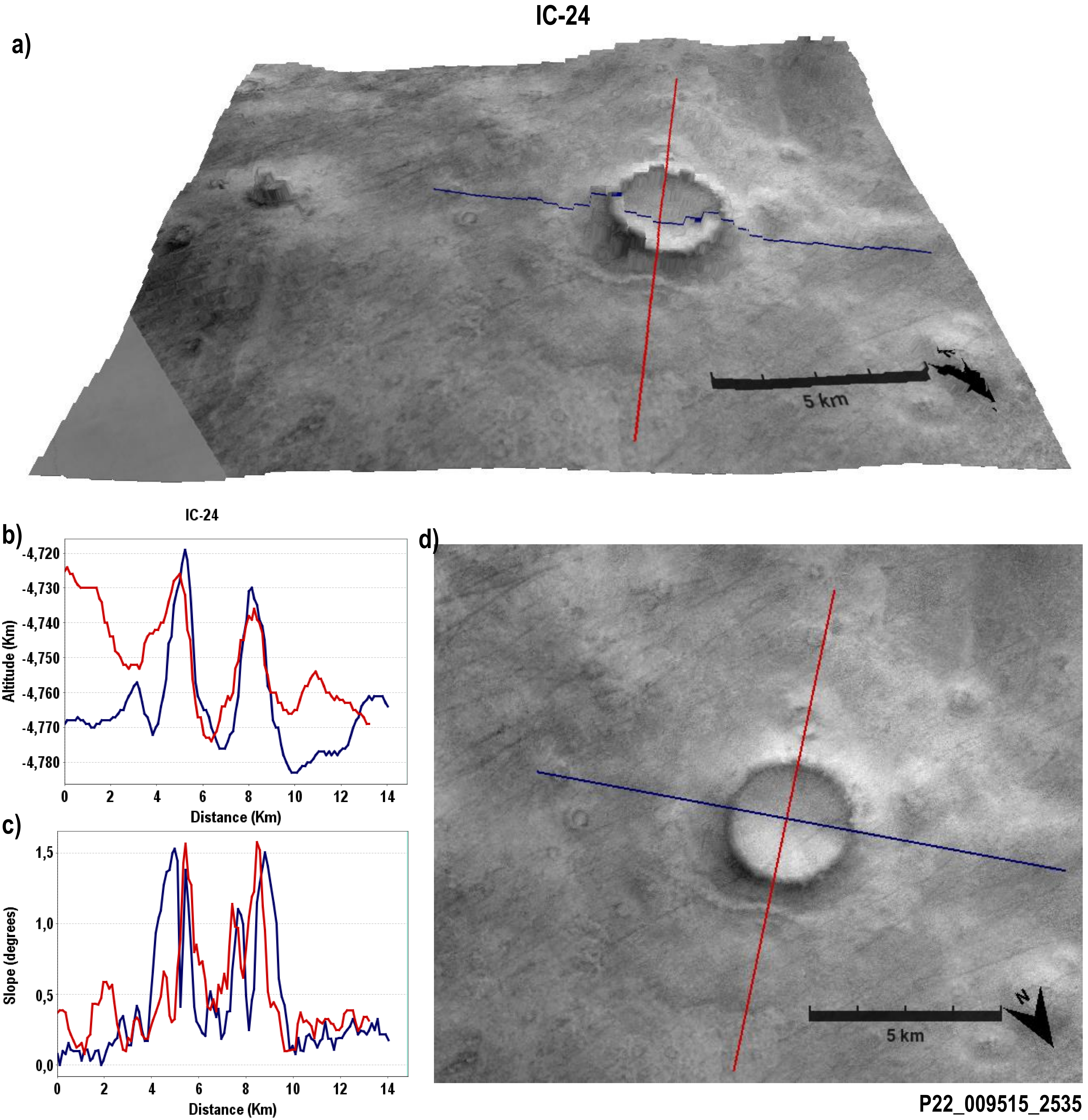
IC-24
a)
5 km
IC-24
b)
-4,720
-4,730
-4,740
-4,750
-4,760
-4,770
-4,780
Altitude (Km)
0
2
4
6
8
10
12
14
Distance (Km)
c)
1,5
1,0
0,5
0,0
Slope (degrees)
0
2
4
6
8
10
12
14
Distance (Km)
d)
5 km
N
P22_009515_2535

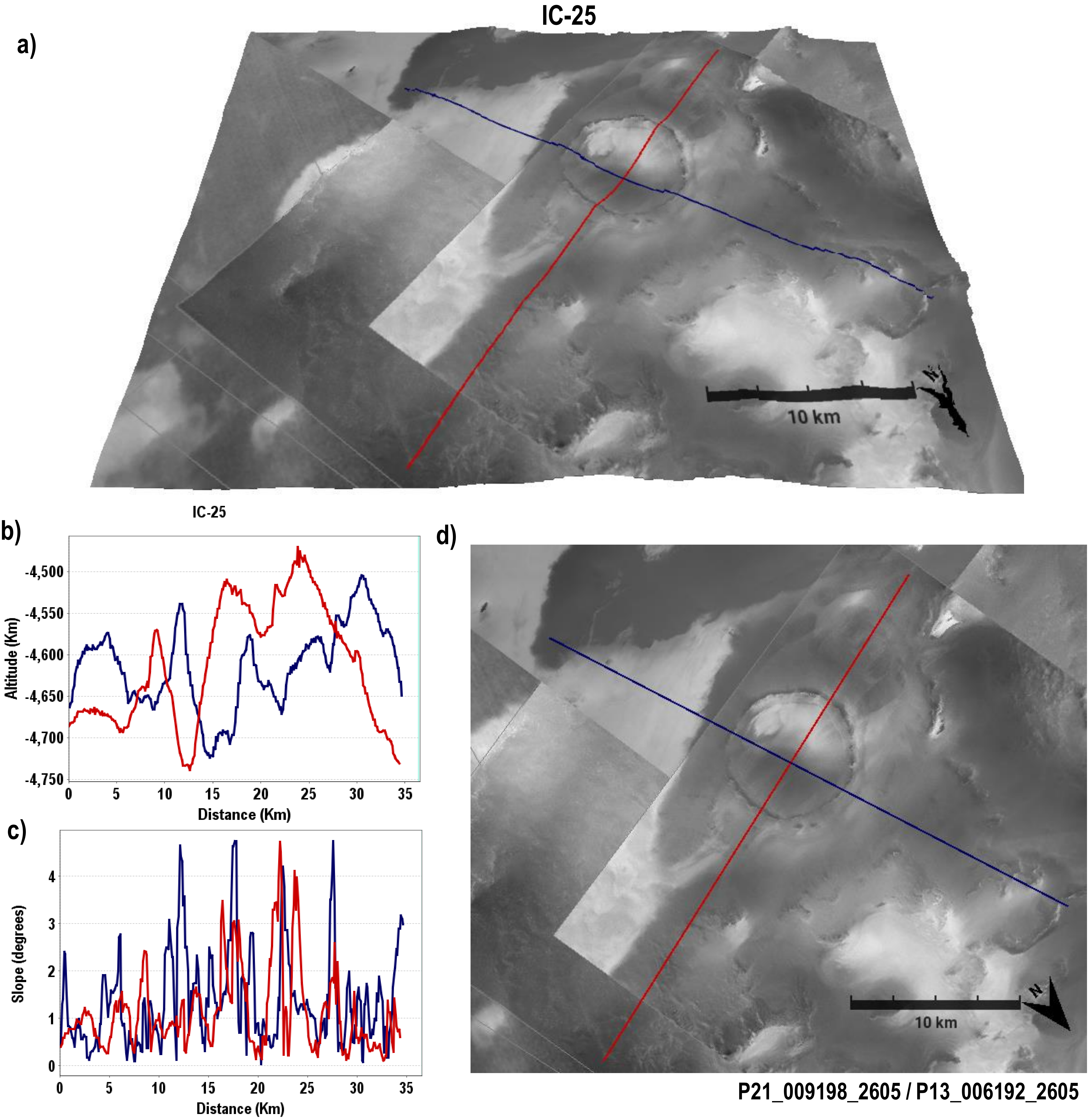
IC-25
a)
10 km
N
IC-25
b)
Altitude (Km)
-4,500
-4,550
-4,600
-4,650
-4,700
-4,750
0 5 10 15 20 25 30 35
Distance (Km)
c)
Slope (degrees)
0 1 2 3 4
0 5 10 15 20 25 30 35
Distance (Km)
d)
10 km
N
P21_009198_2605 / P13_006192_2605

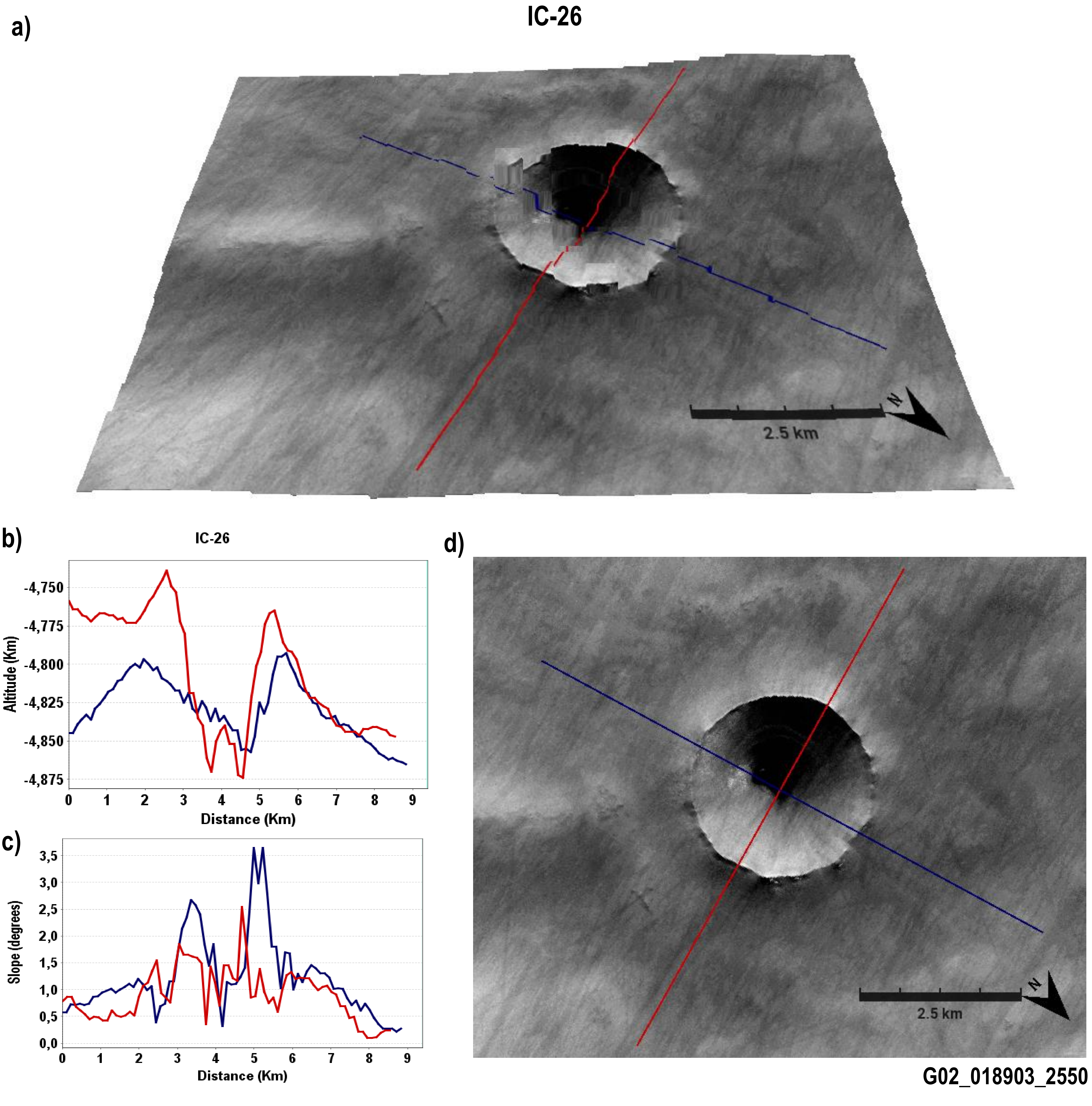

a)
IC-26
2.5 km
N
b)
IC-26
Altitude (Km)
-4,750
-4,775
-4,800
-4,825
-4,850
-4,875
Distance (Km)
0
1
2
3
4
5
6
7
8
9
c)
Slope (degrees)
3,5
3,0
2,5
2,0
1,5
1,0
0,5
0,0
Distance (Km)
d)
2.5 km
N
G02_018903_2550

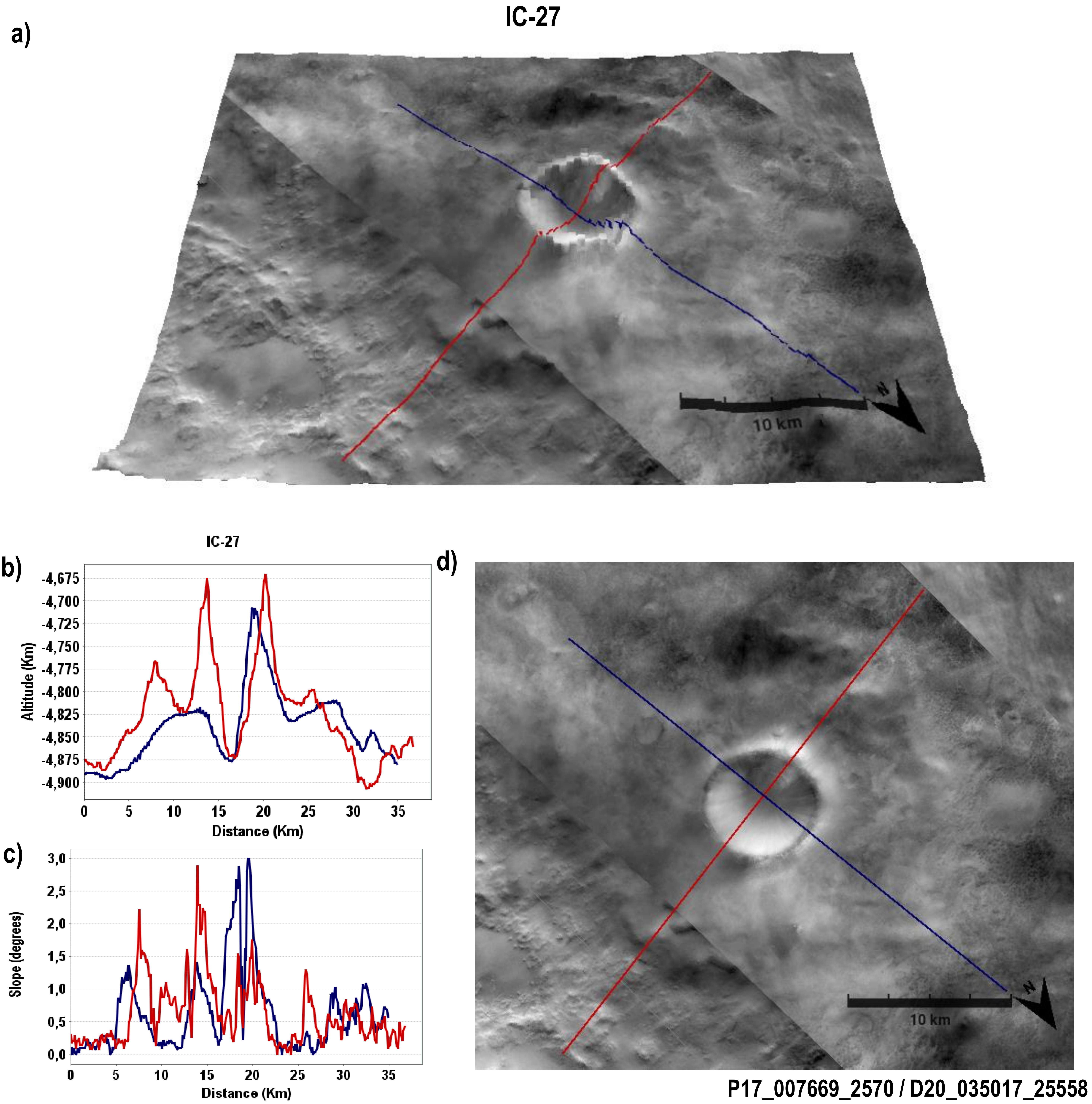

IC-27
a)
10 km
N
b)
IC-27
Altitude (Km)
-4,675
-4,700
-4,725
-4,750
-4,775
-4,800
-4,825
-4,850
-4,875
-4,900
0 5 10 15 20 25 30 35
Distance (Km)
c)
Slope (degrees)
3,0
2,5
2,0
1,5
1,0
0,5
0,0
0 5 10 15 20 25 30 35
Distance (Km)
d)
10 km
N
P17_007669_2570 / D20_035017_25558

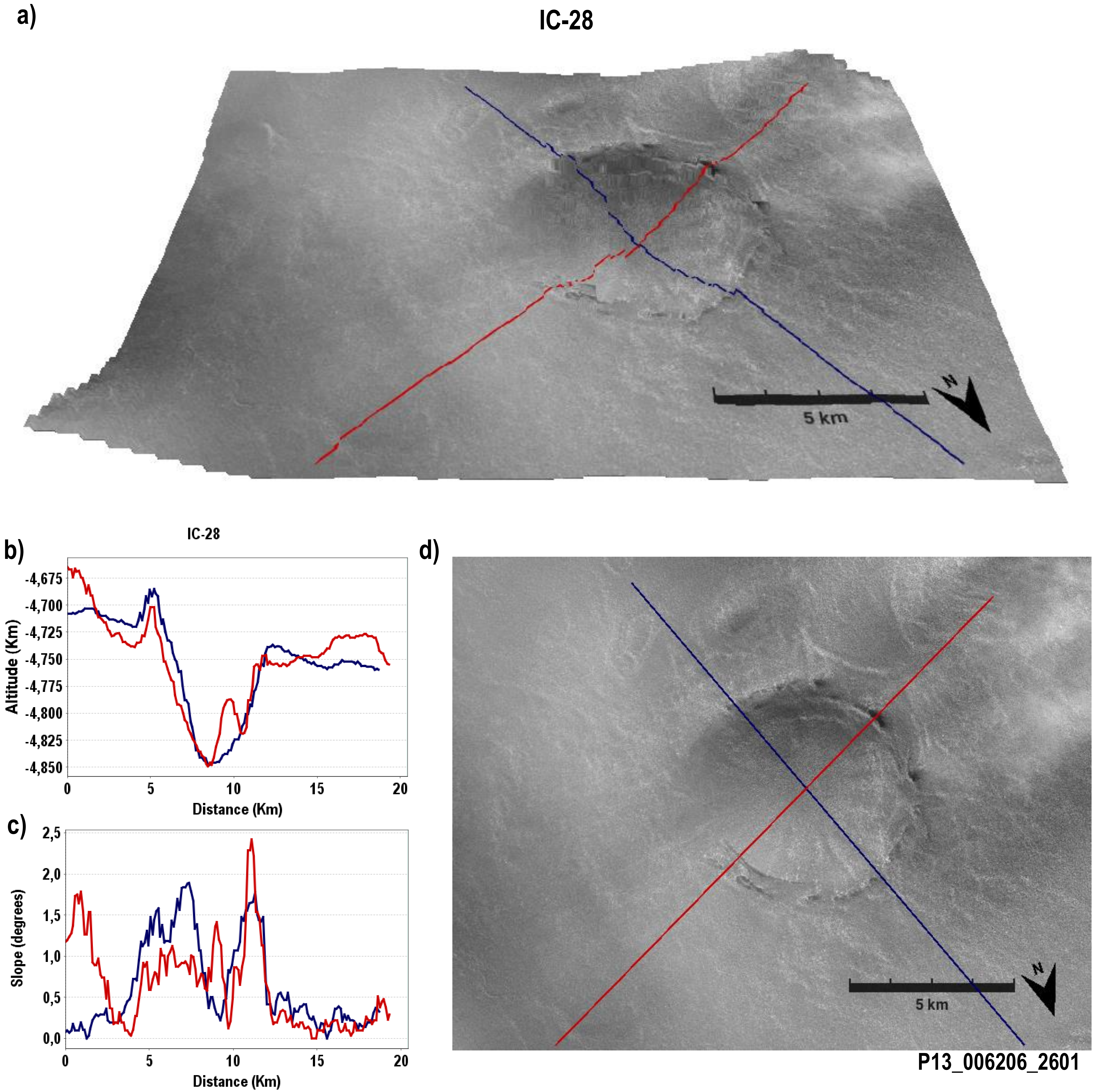

a)
IC-28
5 km
N
b)
IC-28
Altitude (Km)
-4,675
-4,700
-4,725
-4,750
-4,775
-4,800
-4,825
-4,850
0
5
10
15
20
Distance (Km)
c)
Slope (degrees)
2,5
2,0
1,5
1,0
0,5
0,0
0
5
10
15
20
Distance (Km)
d)
5 km
N
P13_006206_2601

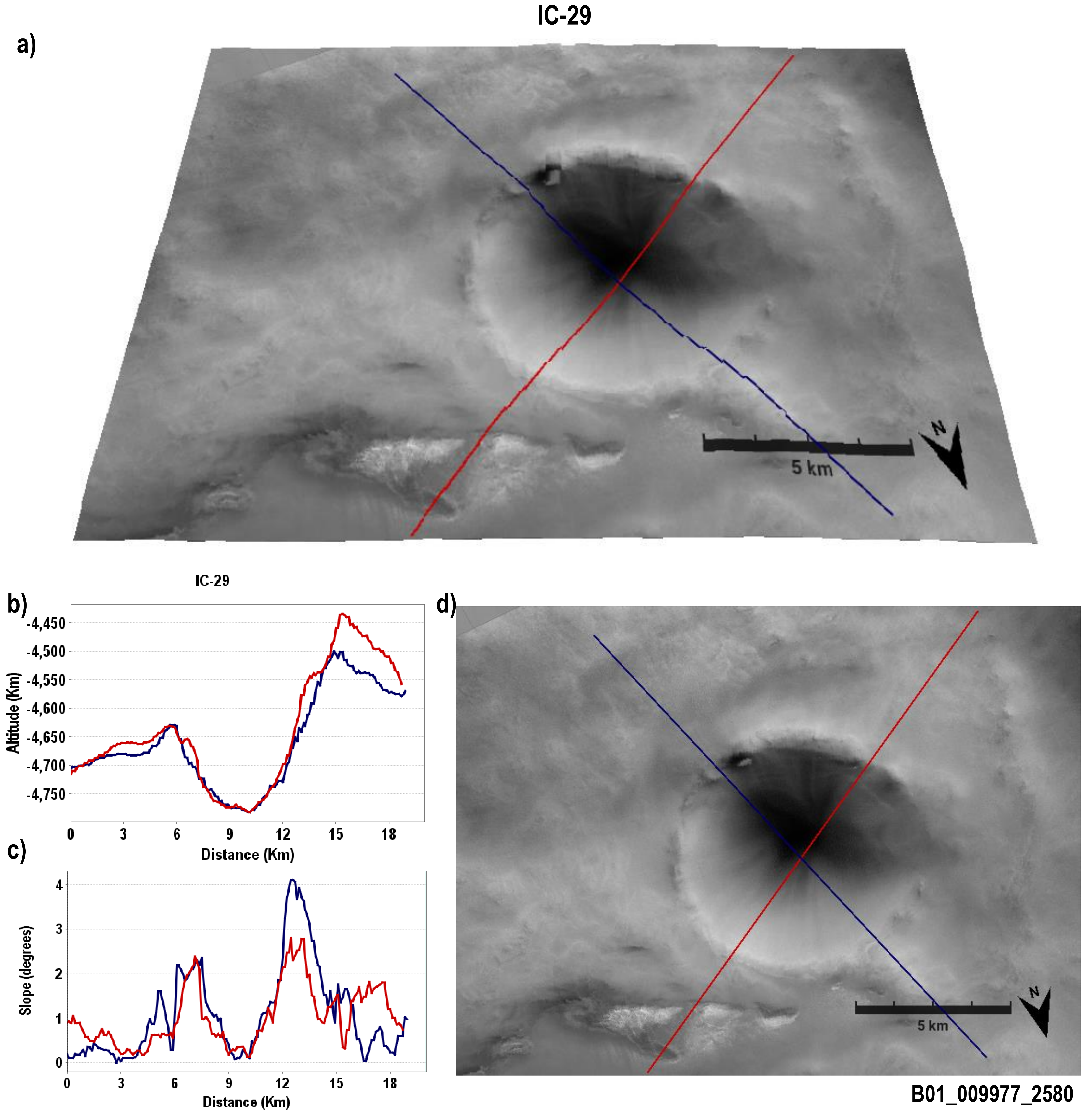

IC-29
a)
5 km
N
IC-29
b)
Altitude (Km)
-4,450
-4,500
-4,550
-4,600
-4,650
-4,700
-4,750
0
3
6
9
12
15
18
Distance (Km)
c)
Slope (degrees)
4
3
2
1
0
0
3
6
9
12
15
18
Distance (Km)
d)
5 km
N
B01_009977_2580

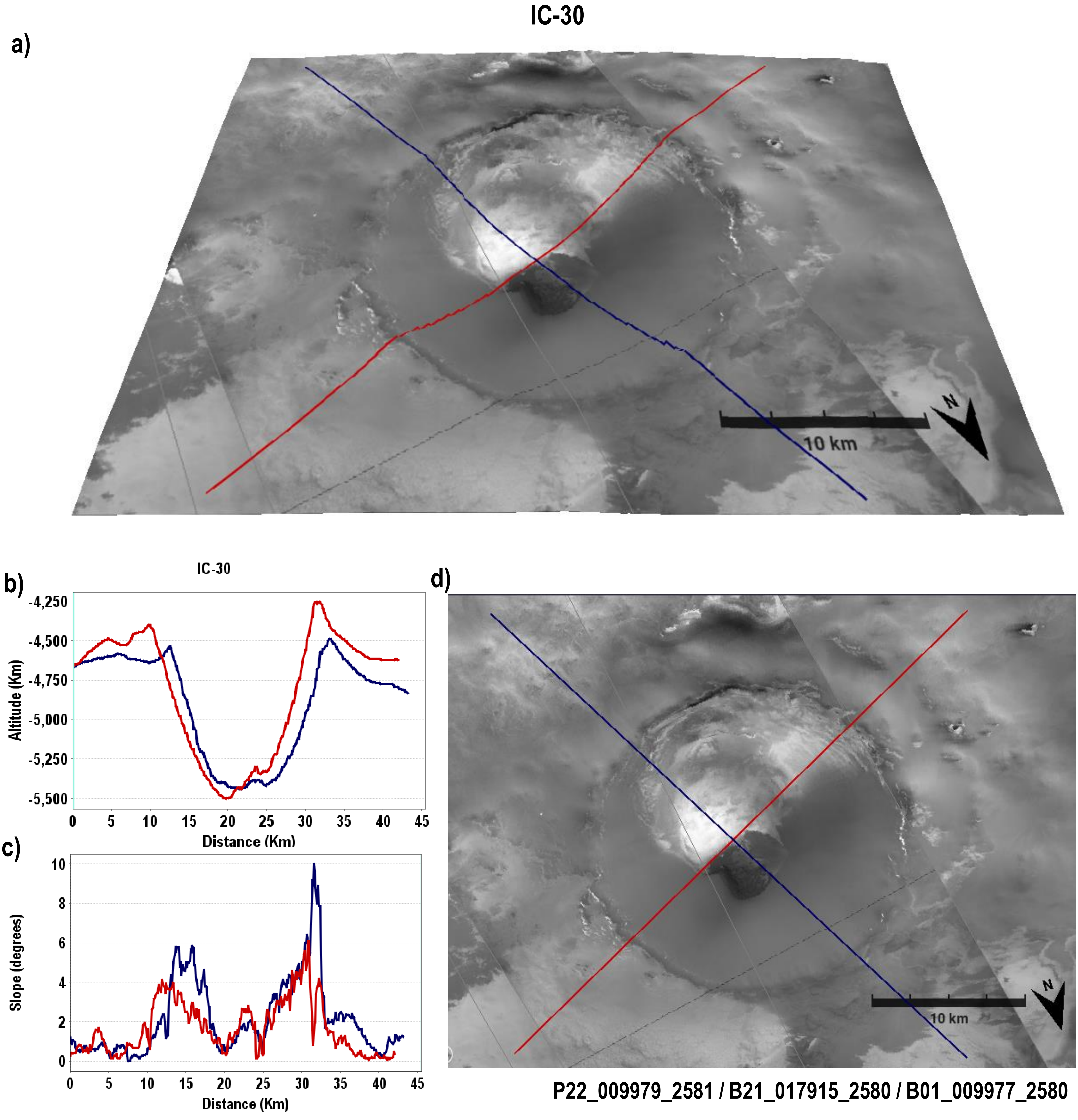
IC-30
a)
10 km
N
b)
IC-30
Altitude (Km)
-4,250
-4,500
-4,750
-5,000
-5,250
-5,500
0
5
10
15
20
25
30
35
40
45
Distance (Km)
c)
Slope (degrees)
10
8
6
4
2
0
Distance (Km)
d)
10 km
N
P22_009979_2581 / B21_017915_2580 / B01_009977_2580

UC-01

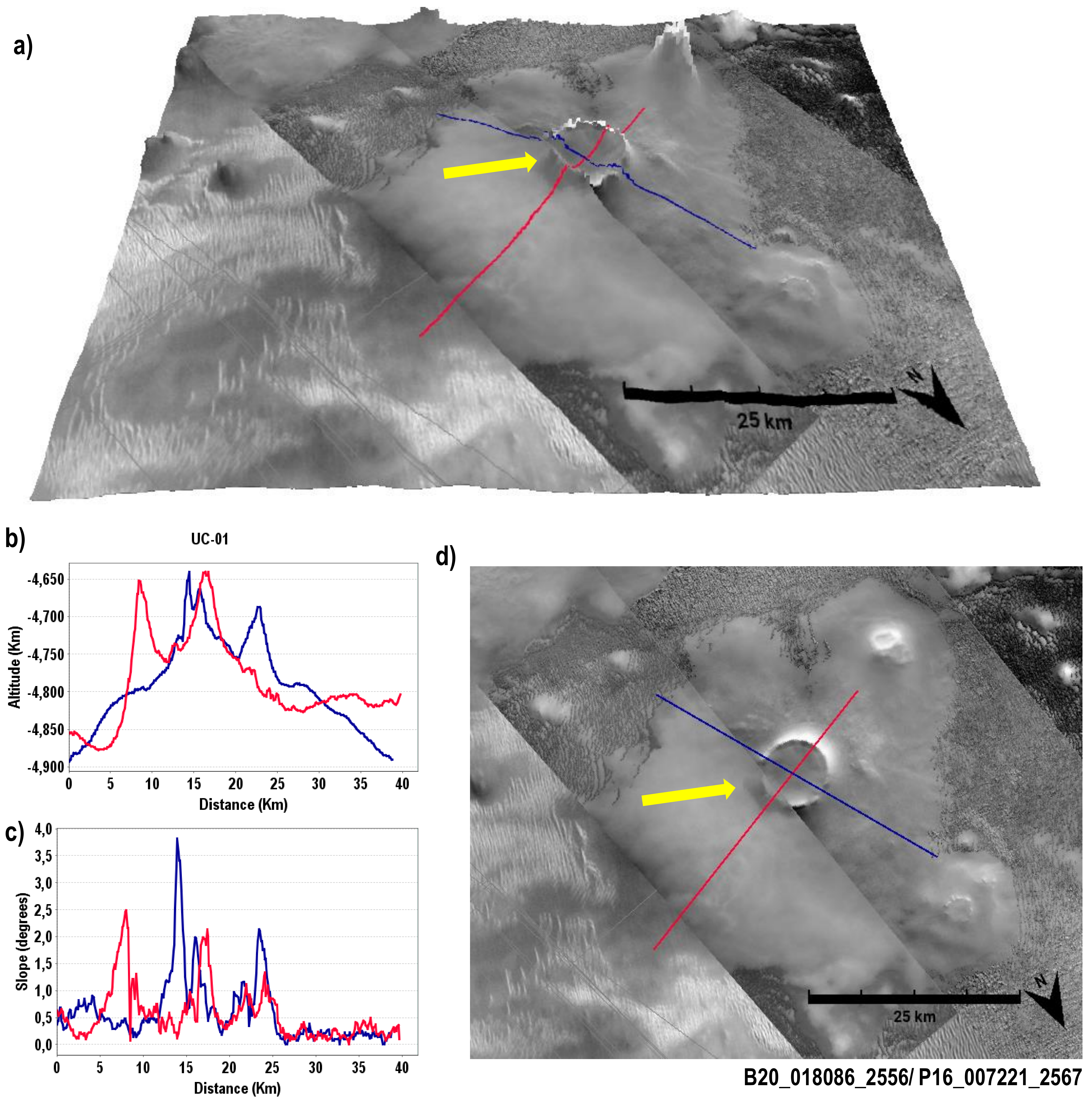

B20_018086_2556/ P16_007221_2567

UC-02

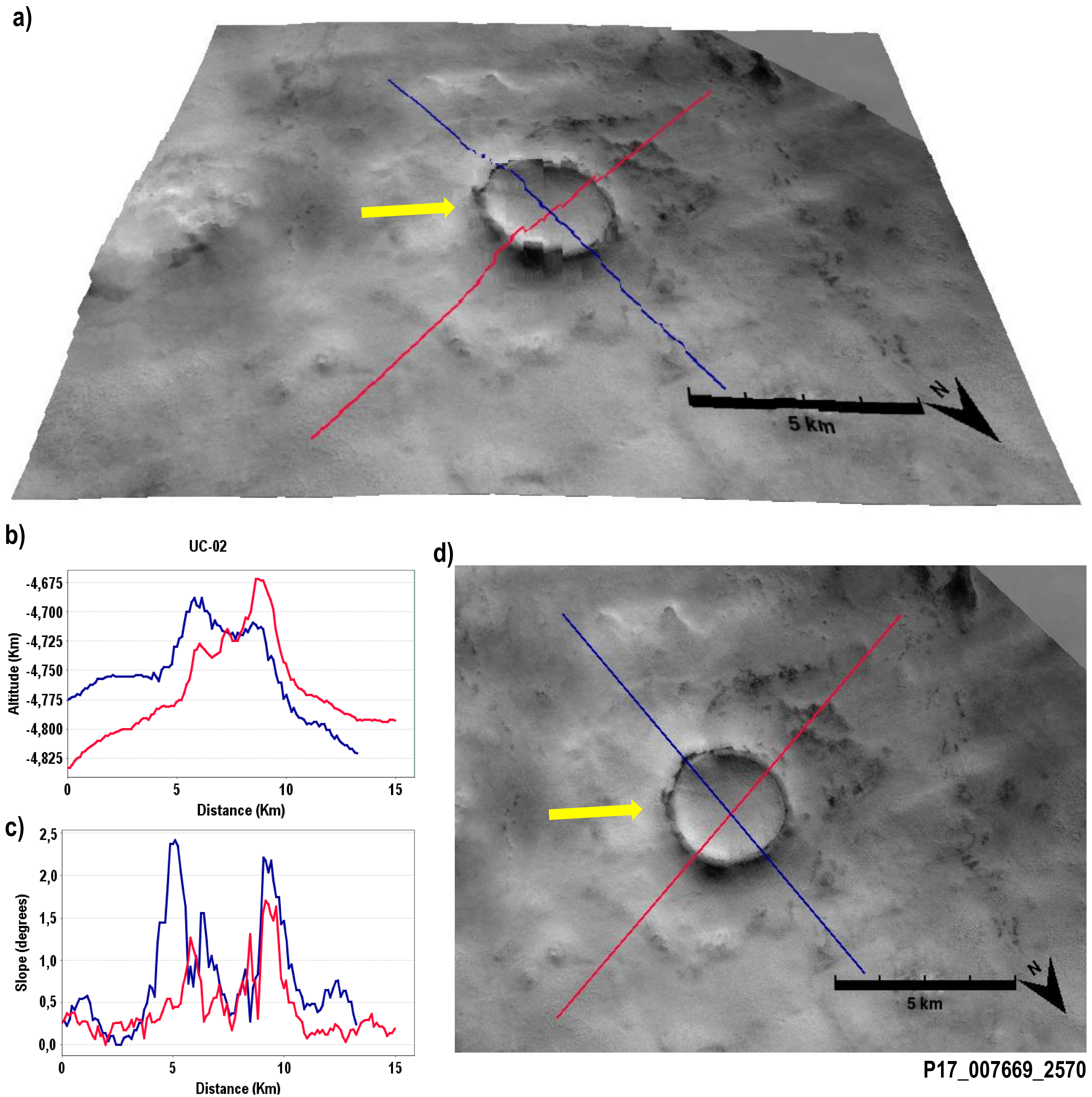

P17_007669_2570

UC-03

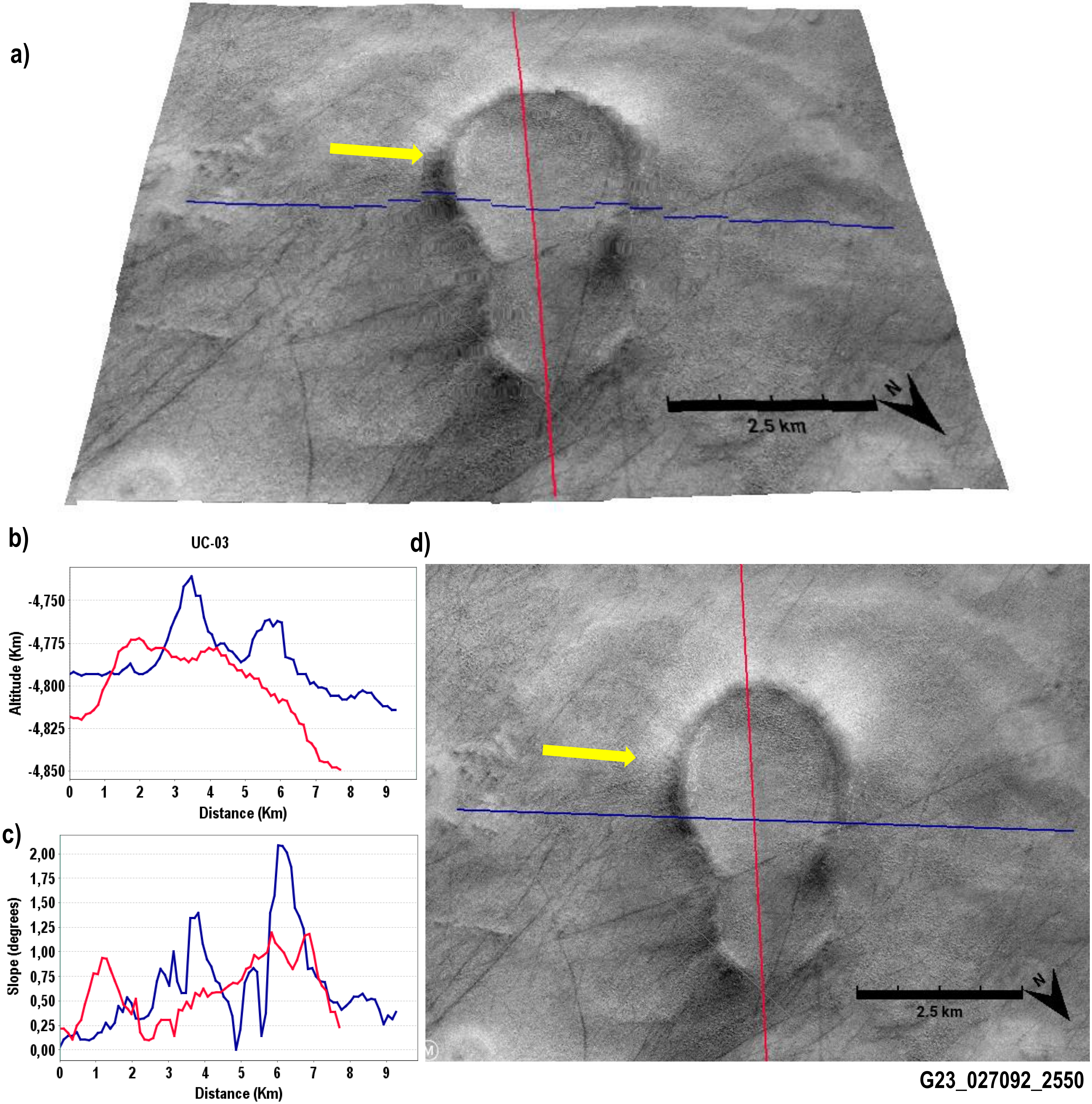

a)
2.5 km
N
b)
UC-03
Altitude (Km)
-4,750
-4,775
-4,800
-4,825
-4,850
Distance (Km)
0 1 2 3 4 5 6 7 8 9
c)
Slope (degrees)
2,00
1,75
1,50
1,25
1,00
0,75
0,50
0,25
0,00
Distance (Km)
0 1 2 3 4 5 6 7 8 9
d)
2.5 km
N
G23_027092_2550

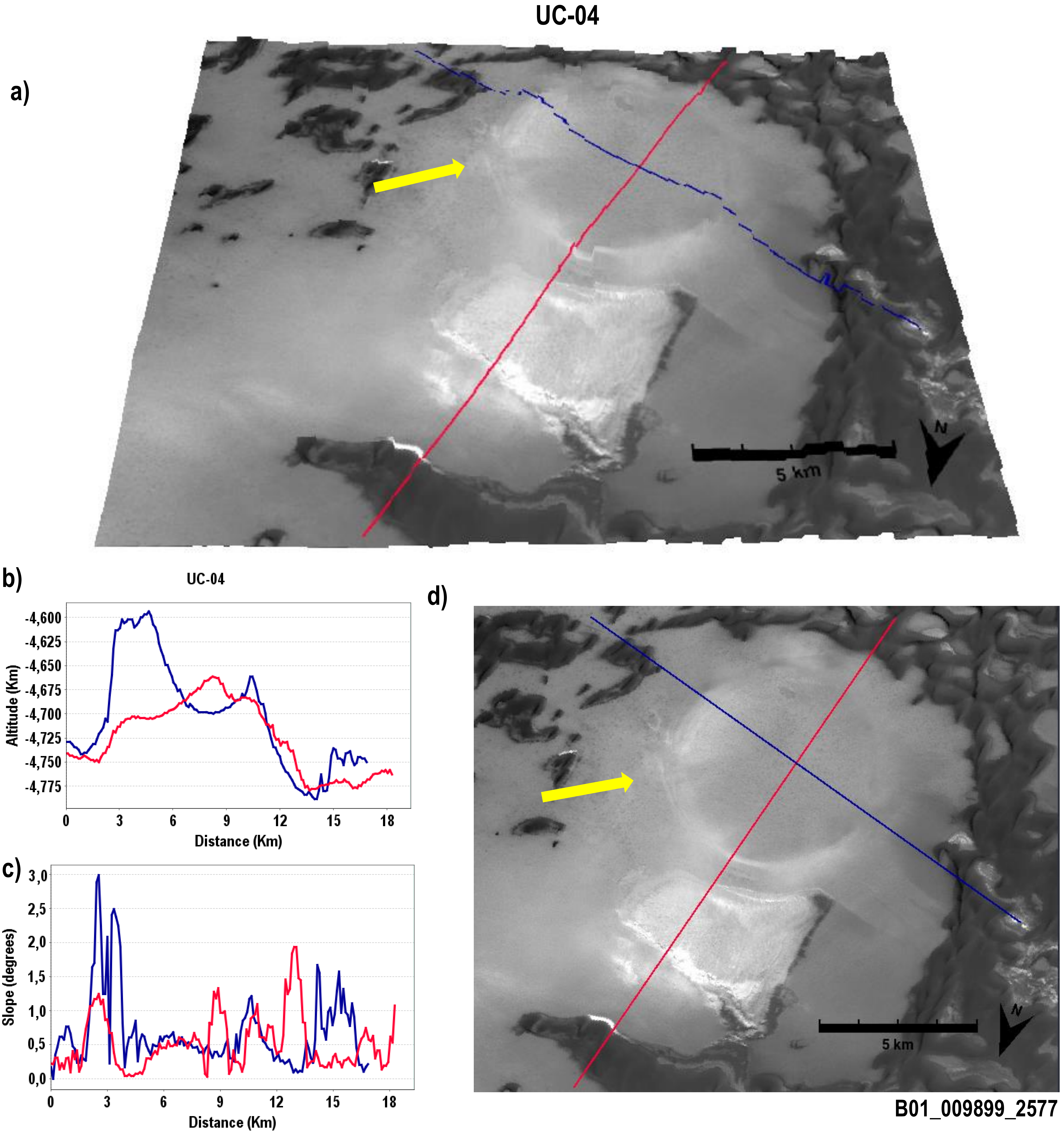

UC-04
a)
5 km
N
b)
UC-04
Altitude (Km)
-4,600
-4,625
-4,650
-4,675
-4,700
-4,725
-4,750
-4,775
0
3
6
9
12
15
18
Distance (Km)
c)
Slope (degrees)
3,0
2,5
2,0
1,5
1,0
0,5
0,0
Distance (Km)
d)
5 km
N
B01_009899_2577

UC-05

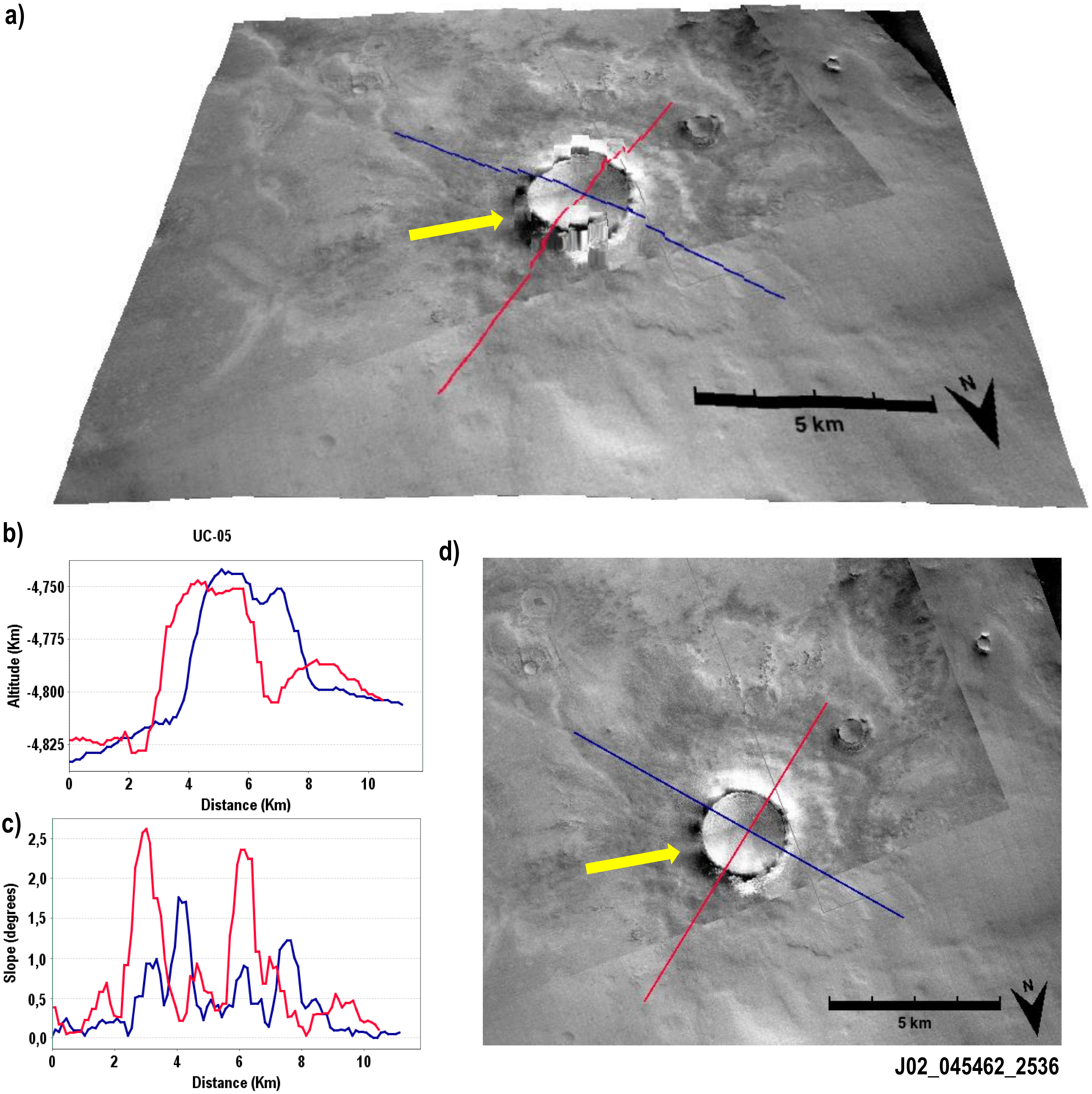

J02_045462_2536

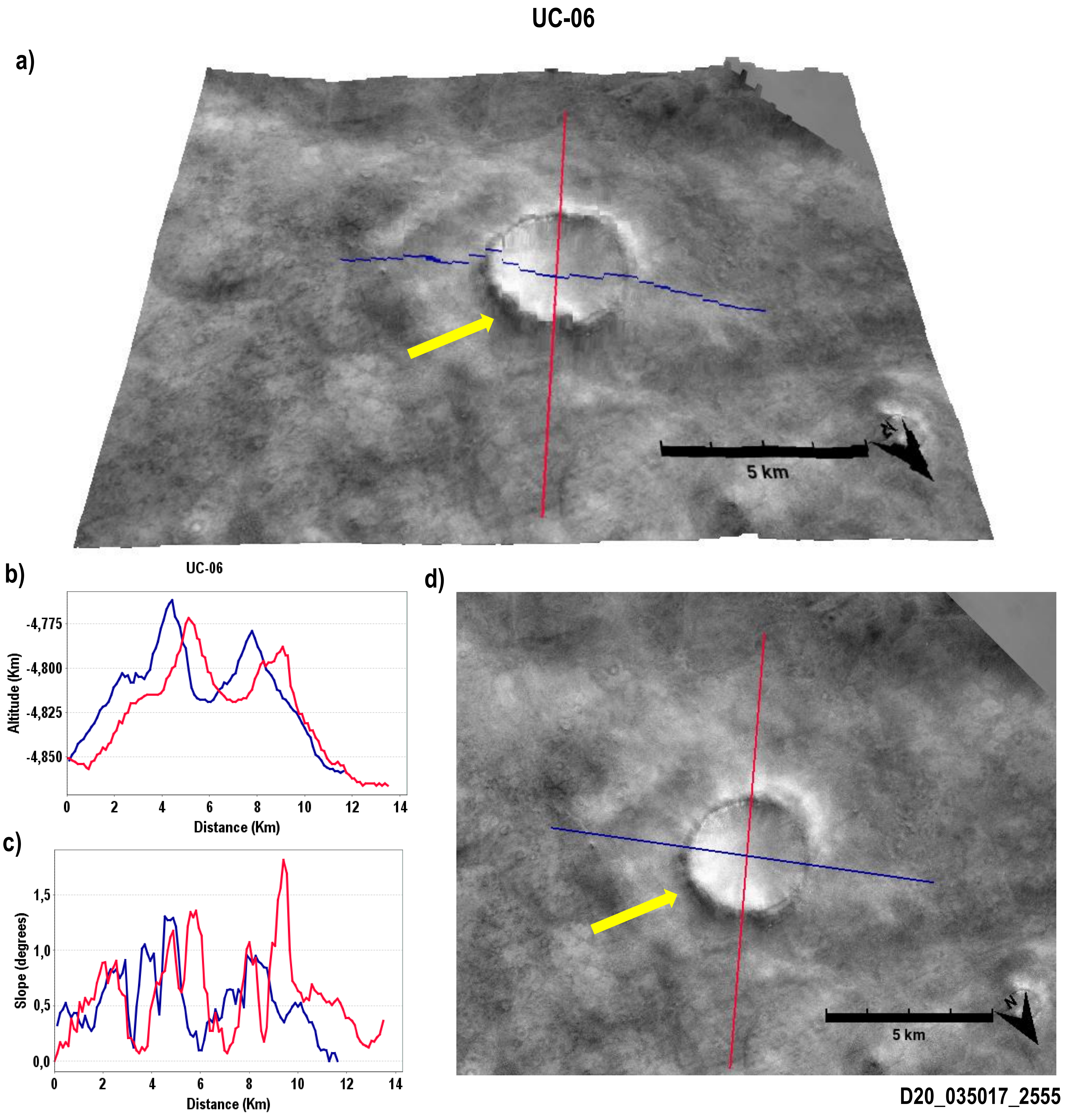
UC-06
a)
5 km
N
b)
UC-06
-4,775
-4,800
-4,825
-4,850
Altitude (Km)
0
2
4
6
8
10
12
14
Distance (Km)
c)
1,5
1,0
0,5
0,0
Slope (degrees)
0
2
4
6
8
10
12
14
Distance (Km)
d)
5 km
N
D20_035017_2555

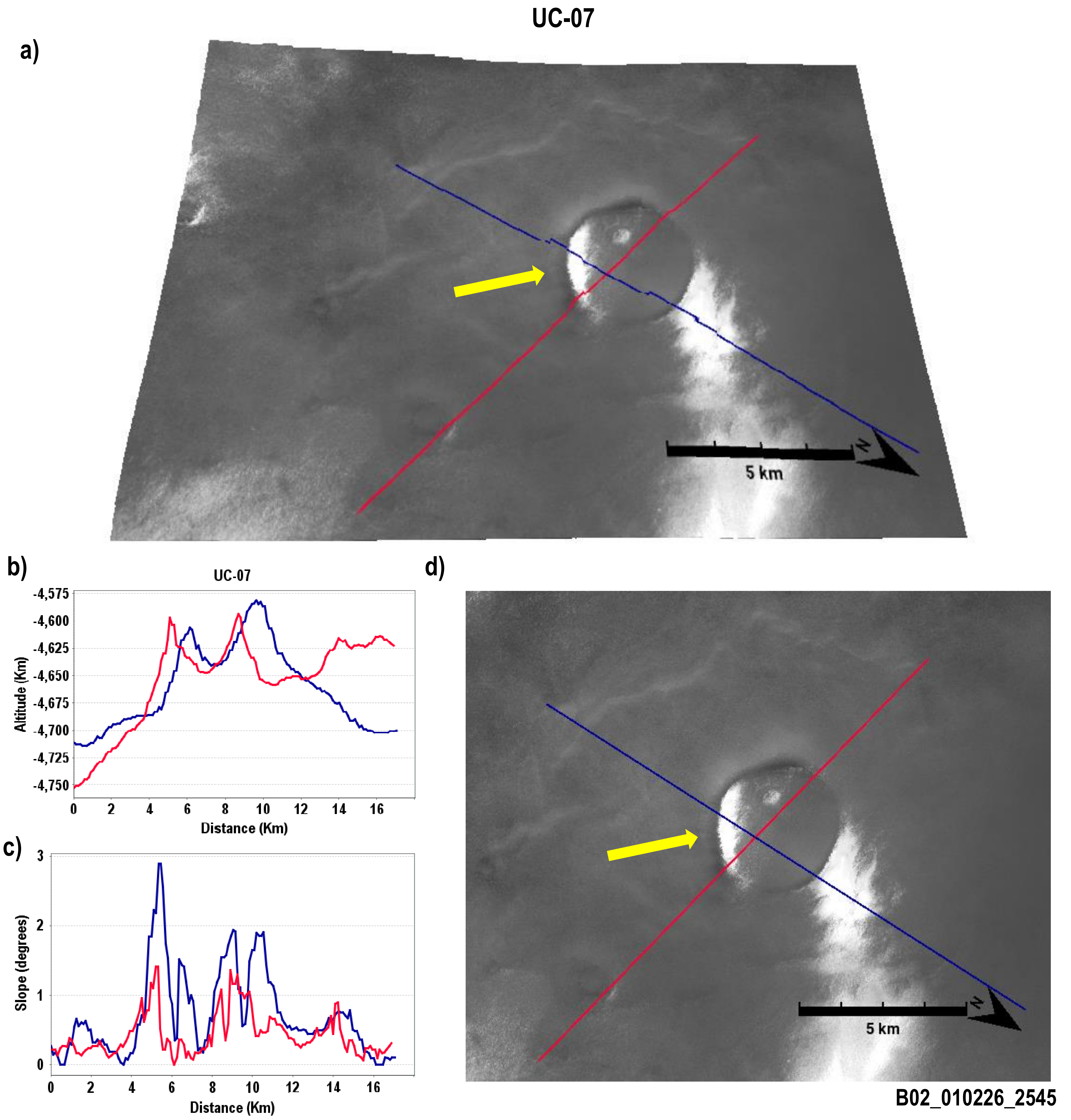
UC-07
a)
5 km
N
b)
UC-07
-4,575
-4,600
-4,625
-4,650
-4,675
-4,700
-4,725
-4,750
Altitude (Km)
0
2
4
6
8
10
12
14
16
Distance (Km)
c)
3
2
1
0
Slope (degrees)
0
2
4
6
8
10
12
14
16
Distance (Km)
d)
5 km
N
B02_010226_2545

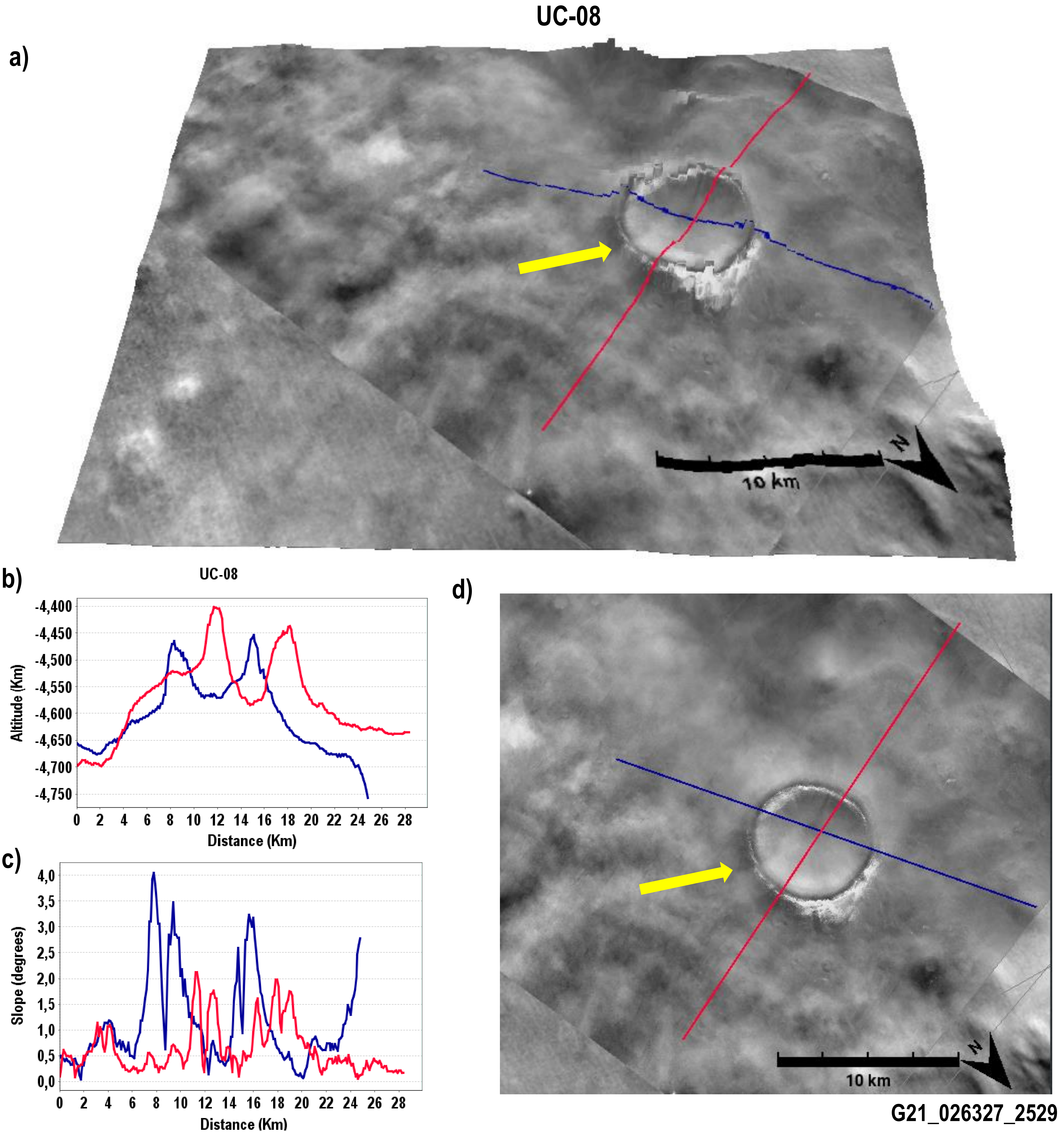
UC-08
a)
10 km
N
b)
UC-08
Altitude (Km)
-4,400
-4,450
-4,500
-4,550
-4,600
-4,650
-4,700
-4,750
0 2 4 6 8 10 12 14 16 18 20 22 24 26 28
Distance (Km)
c)
Slope (degrees)
4,0
3,5
3,0
2,5
2,0
1,5
1,0
0,5
0,0
0 2 4 6 8 10 12 14 16 18 20 22 24 26 28
Distance (Km)
d)
10 km
N
G21_026327_2529

UC-09

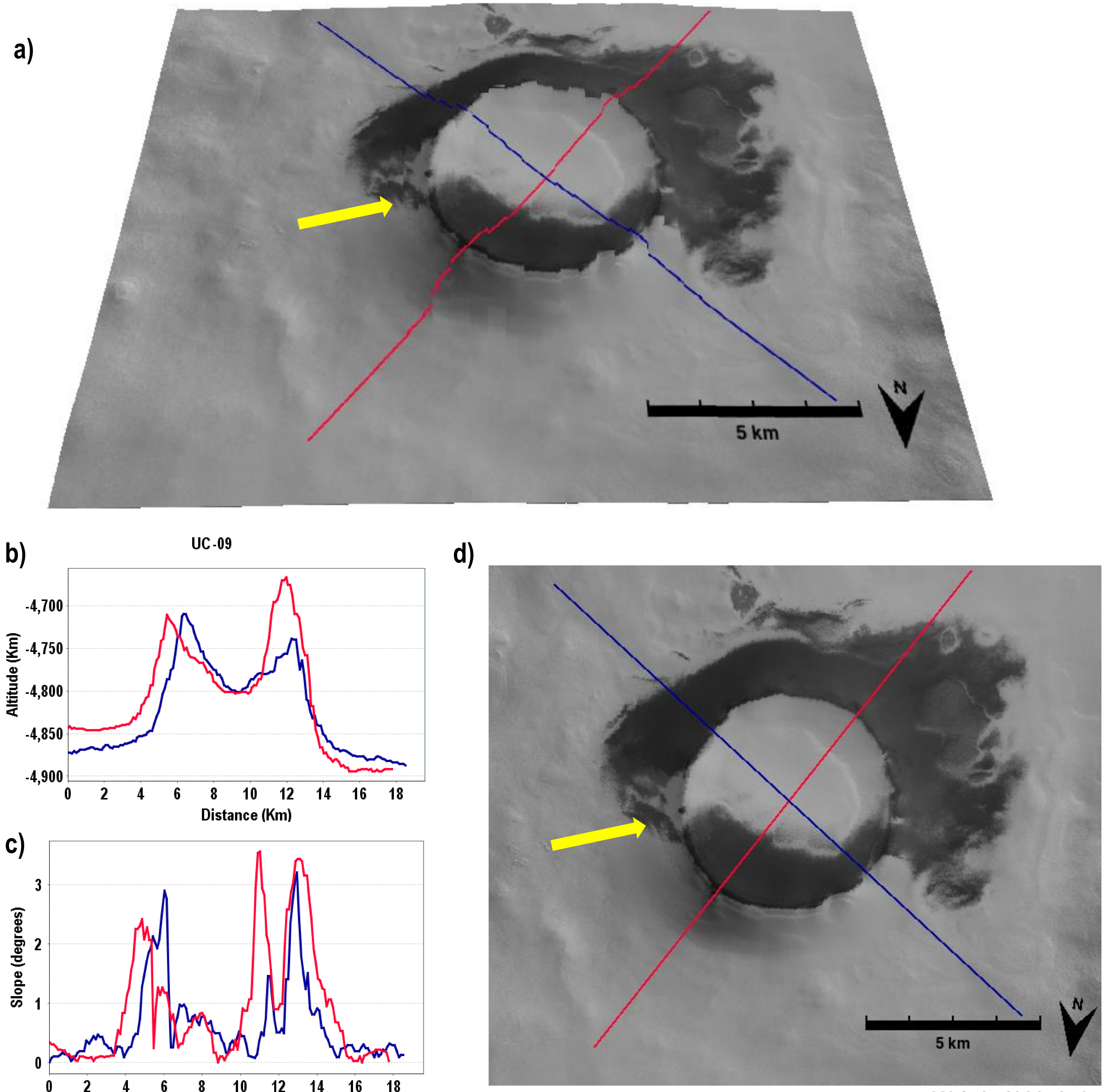

K04_054930_2565

UC-10

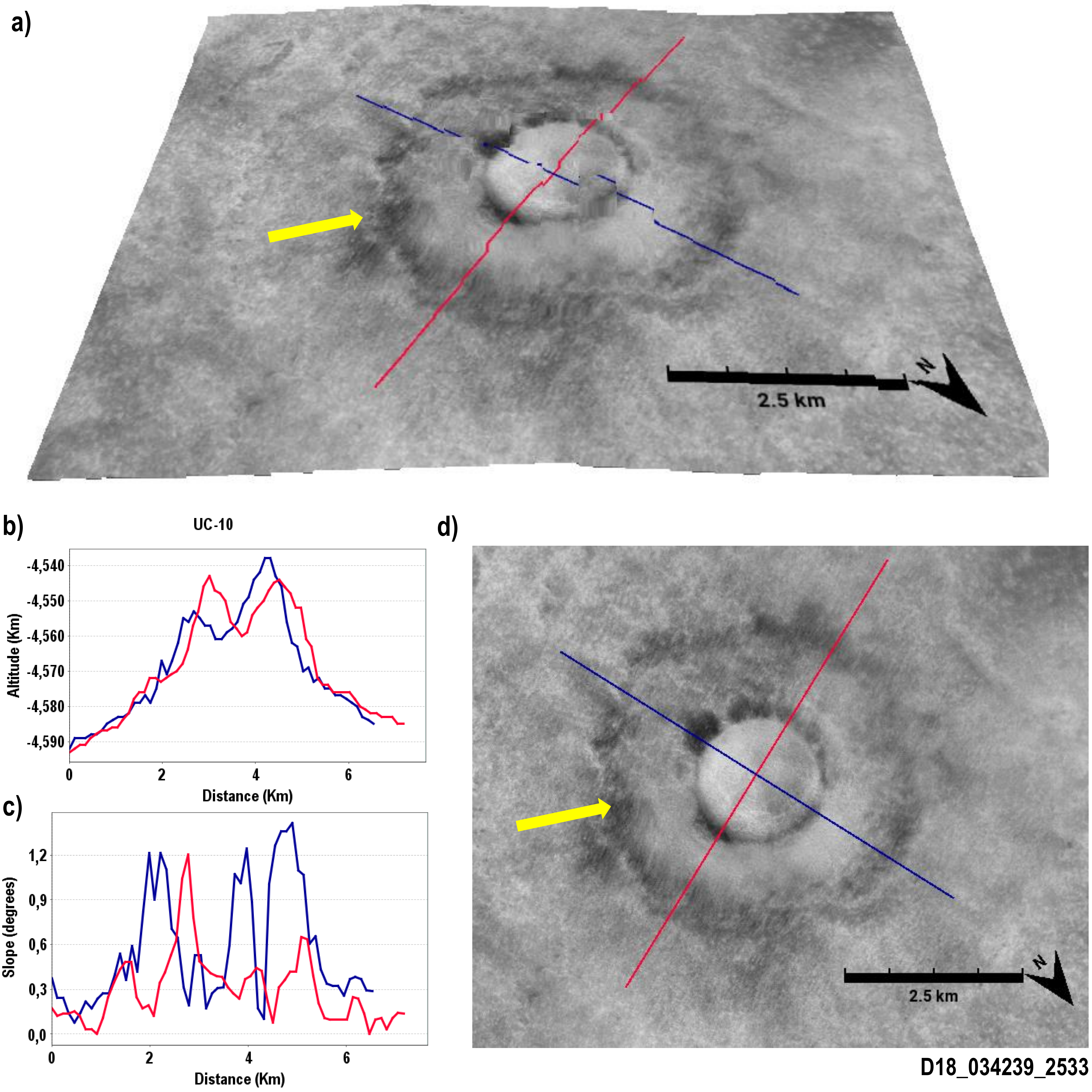

a)
2.5 km
N
b)
UC-10
Altitude (Km)
-4,540
-4,550
-4,560
-4,570
-4,580
-4,590
0
2
4
6
Distance (Km)
c)
Slope (degrees)
1,2
0,9
0,6
0,3
0,0
0
2
4
6
Distance (Km)
d)
2.5 km
N
D18_034239_2533

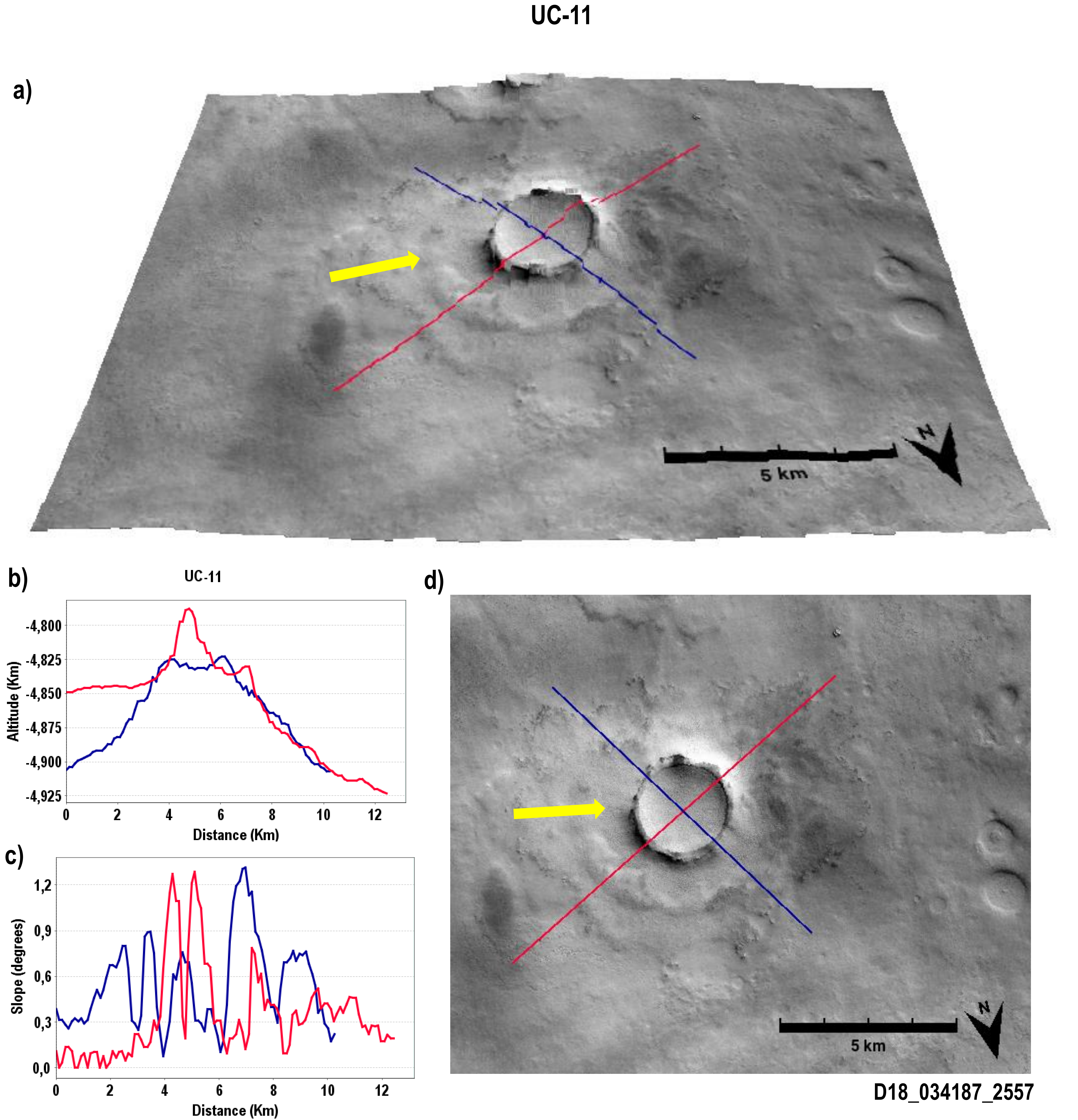
UC-11
a)
5 km
N
b)
UC-11
Altitude (Km)
-4,800
-4,825
-4,850
-4,875
-4,900
-4,925
0 2 4 6 8 10 12
Distance (Km)
c)
Slope (degrees)
1,2
0,9
0,6
0,3
0,0
0 2 4 6 8 10 12
Distance (Km)
d)
5 km
N
D18_034187_2557

UC-12

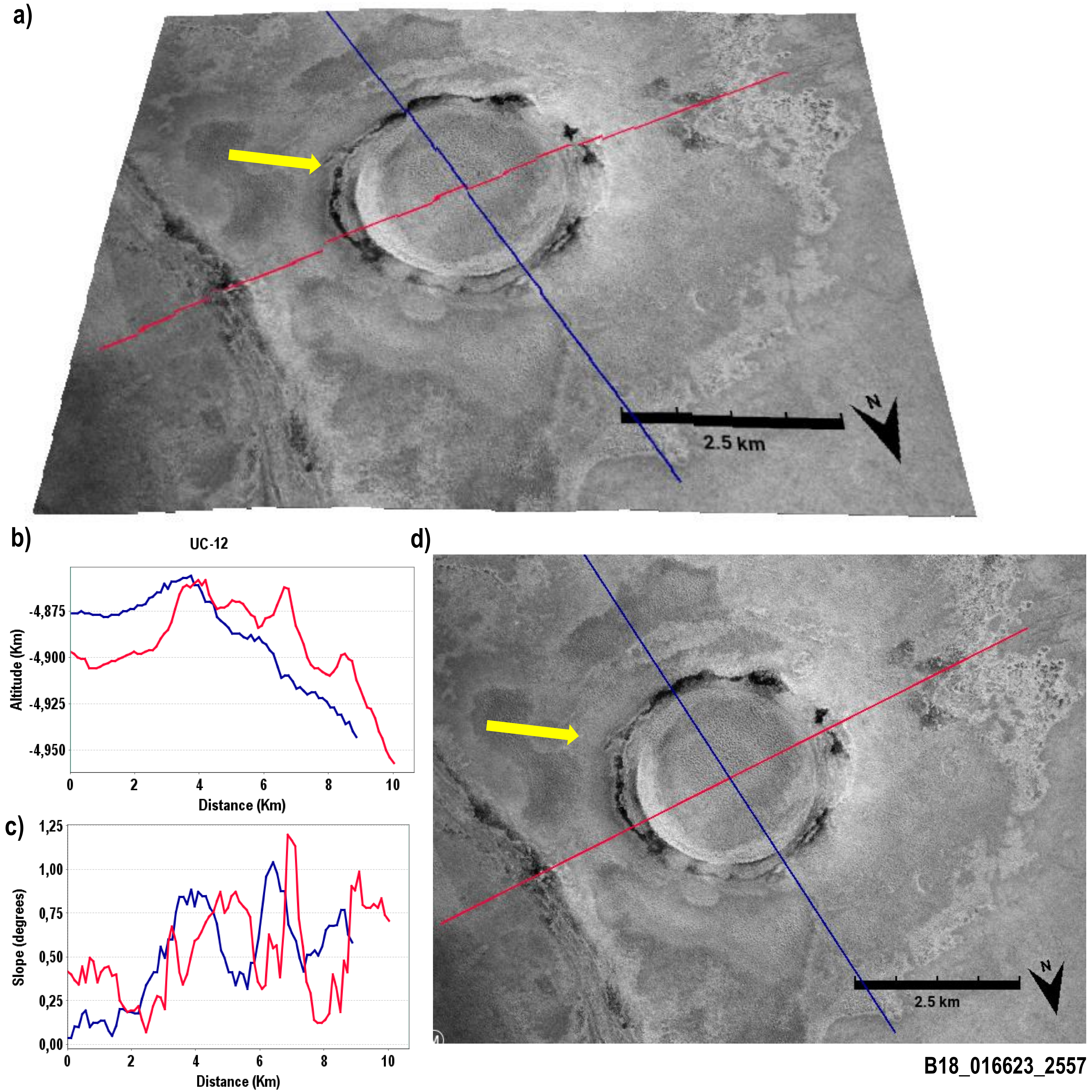
a)
2.5 km
N
b)
UC-12
Altitude (Km)
-4,875
-4,900
-4,925
-4,950
0
2
4
6
8
10
Distance (Km)
c)
Slope (degrees)
1,25
1,00
0,75
0,50
0,25
0,00
0
2
4
6
8
10
Distance (Km)
d)
2.5 km
N
B18_016623_2557

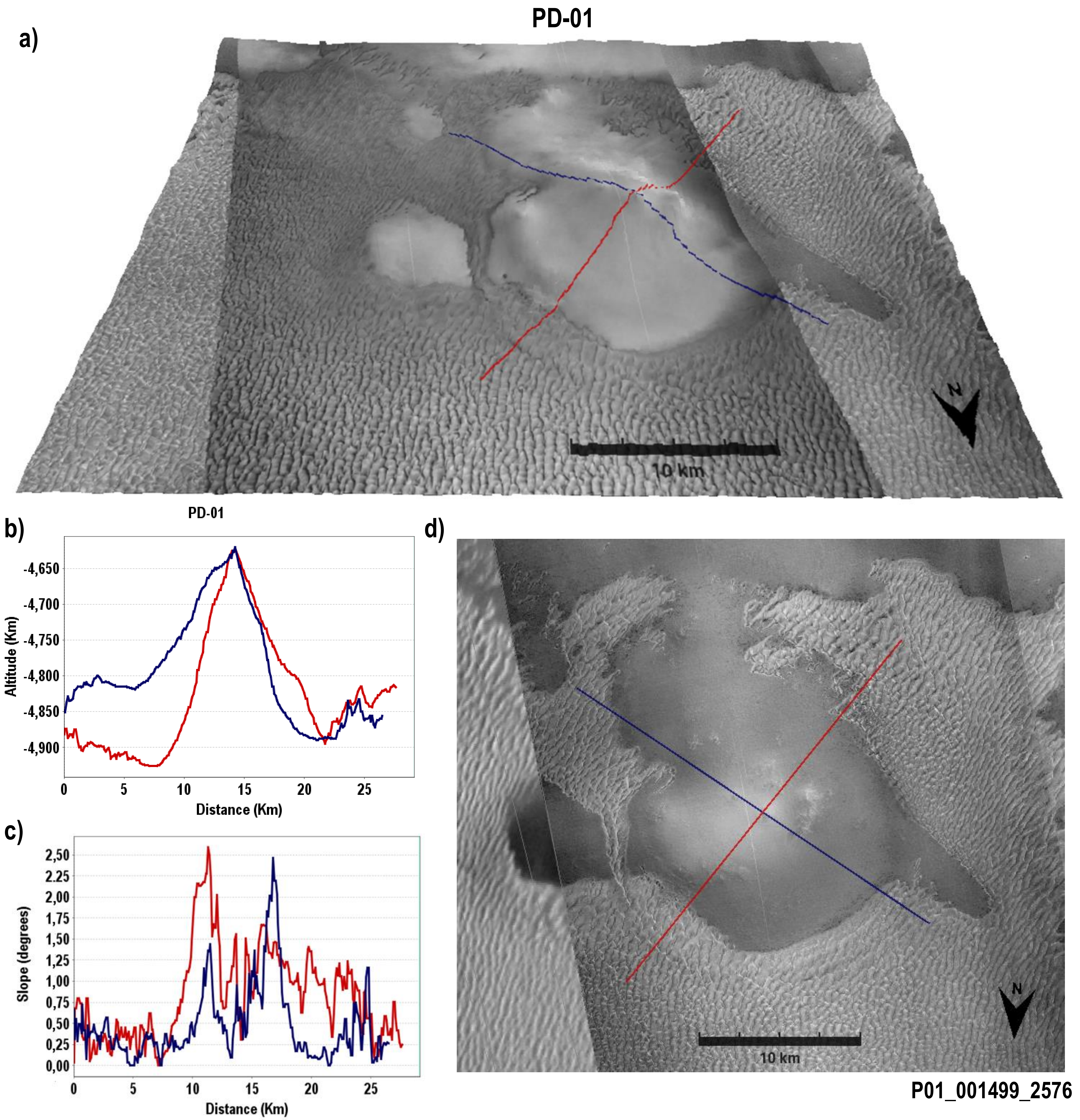
PD-01
a)
10 km
N
PD-01
b)
Altitude (Km)
-4,650
-4,700
-4,750
-4,800
-4,850
-4,900
0
5
10
15
20
25
Distance (Km)
c)
Slope (degrees)
2,50
2,25
2,00
1,75
1,50
1,25
1,00
0,75
0,50
0,25
0,00
0
5
10
15
20
25
Distance (Km)
d)
N
10 km
P01_001499_2576

PD-02

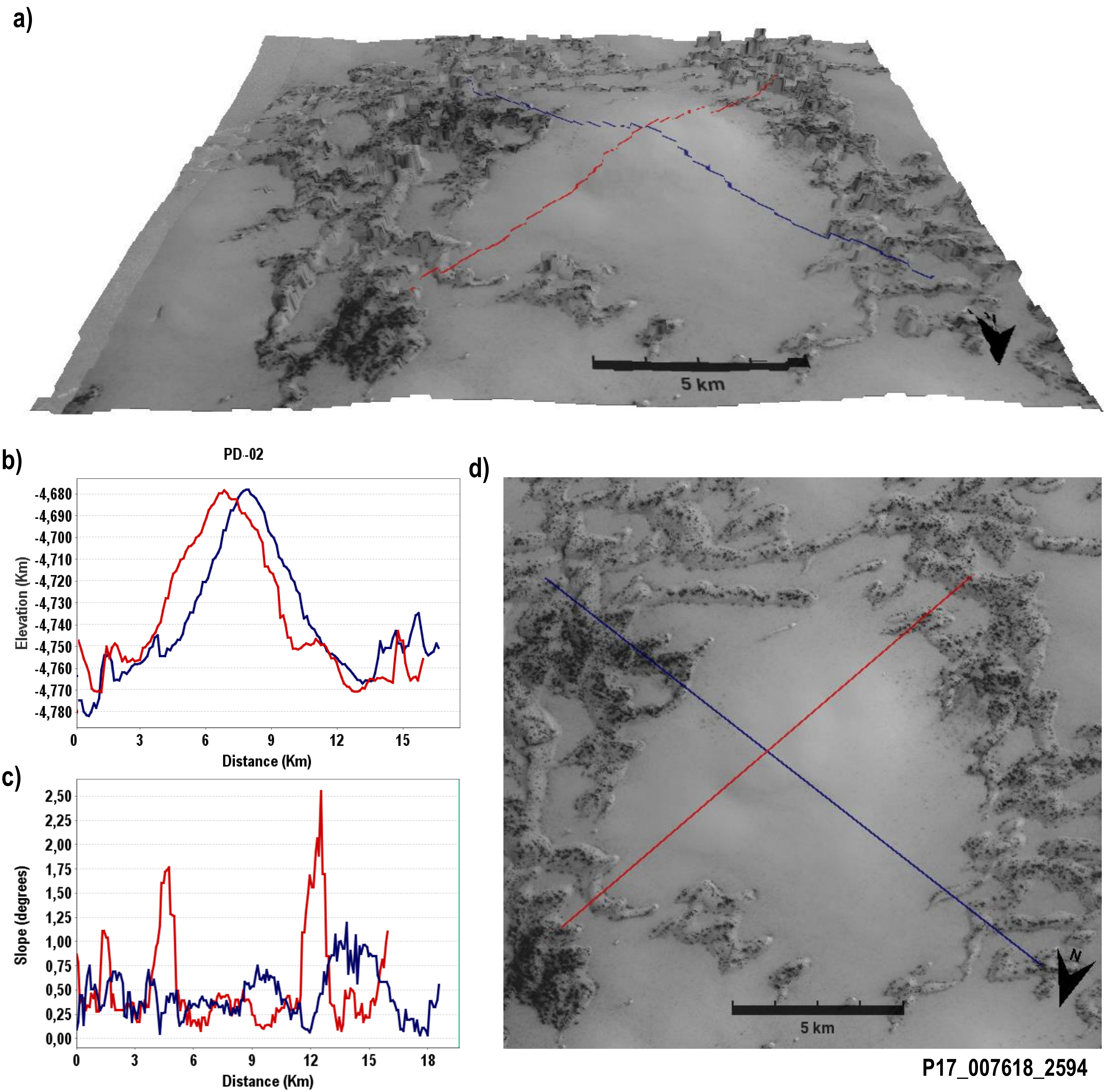

a)
5 km
b)
PD-02
Elevation (Km)
-4,680
-4,690
-4,700
-4,710
-4,720
-4,730
-4,740
-4,750
-4,760
-4,770
-4,780
0
3
6
9
12
15
Distance (Km)
c)
Slope (degrees)
2,50
2,25
2,00
1,75
1,50
1,25
1,00
0,75
0,50
0,25
0,00
0
3
6
9
12
15
18
Distance (Km)
d)
N
5 km
P17_007618_2594

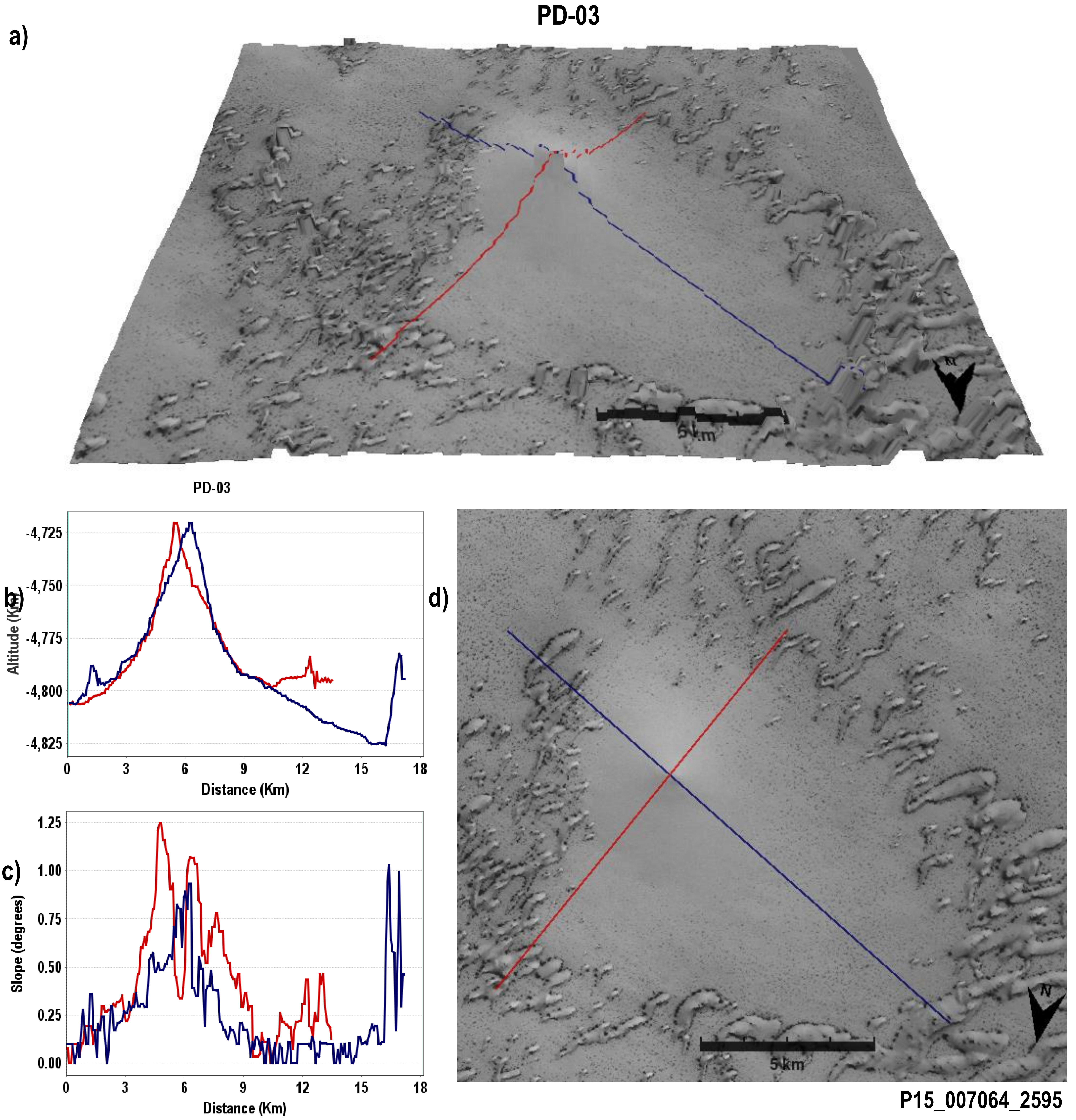

PD-03
a)
5 km
PD-03
b)
Altitude (Km)
-4,725
-4,750
-4,775
-4,800
-4,825
0
3
6
9
12
15
18
Distance (Km)
c)
Slope (degrees)
1.25
1.00
0.75
0.50
0.25
0.00
0
3
6
9
12
15
18
Distance (Km)
d)
N
5 km
P15_007064_2595

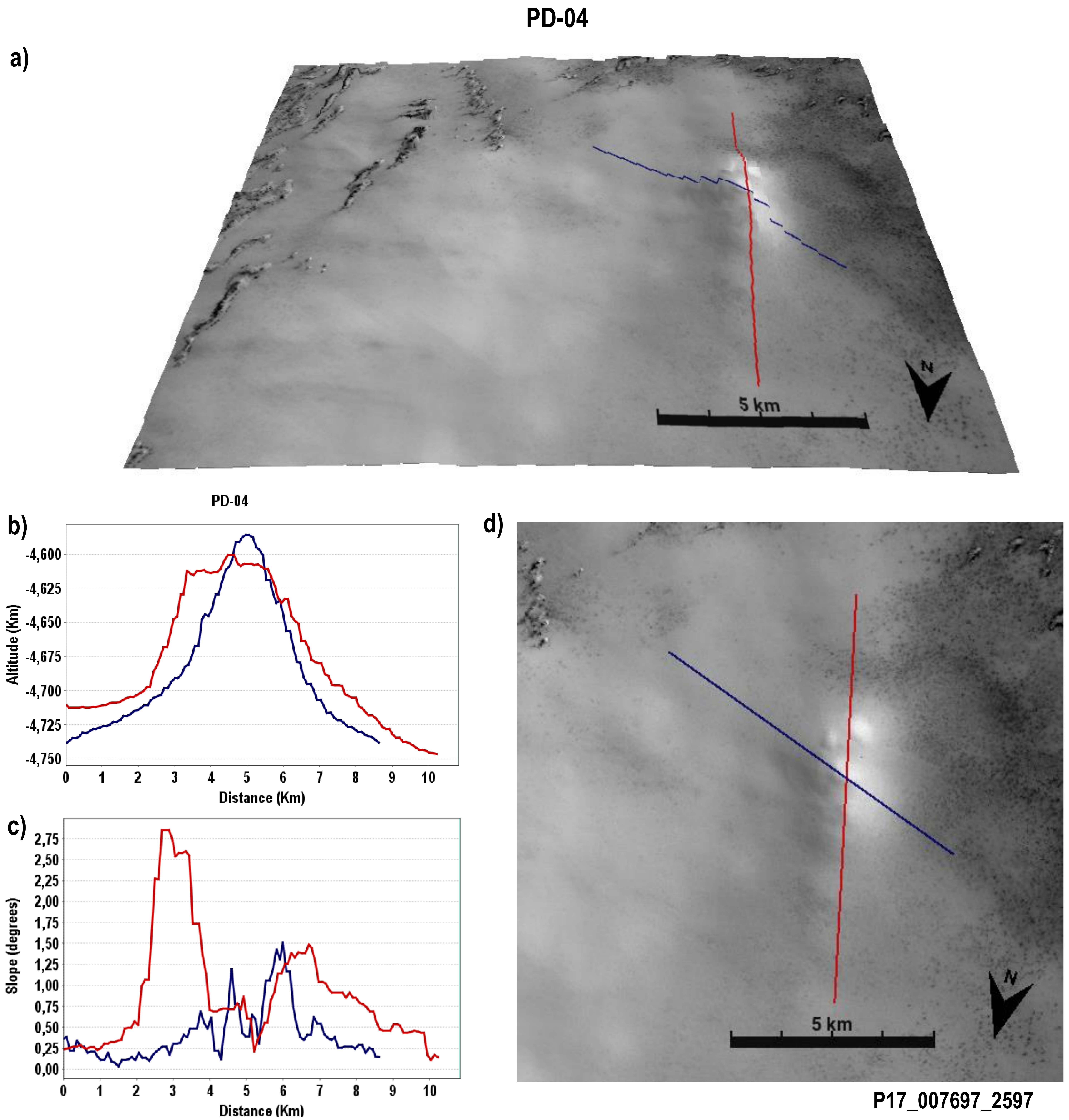
PD-04
a)
5 km
N
PD-04
b)
Altitude (Km)
-4,600
-4,625
-4,650
-4,675
-4,700
-4,725
-4,750
0 1 2 3 4 5 6 7 8 9 10
Distance (Km)
c)
Slope (degrees)
2,75
2,50
2,25
2,00
1,75
1,50
1,25
1,00
0,75
0,50
0,25
0,00
0 1 2 3 4 5 6 7 8 9 10
Distance (Km)
d)
5 km
N
P17_007697_2597

PD-05

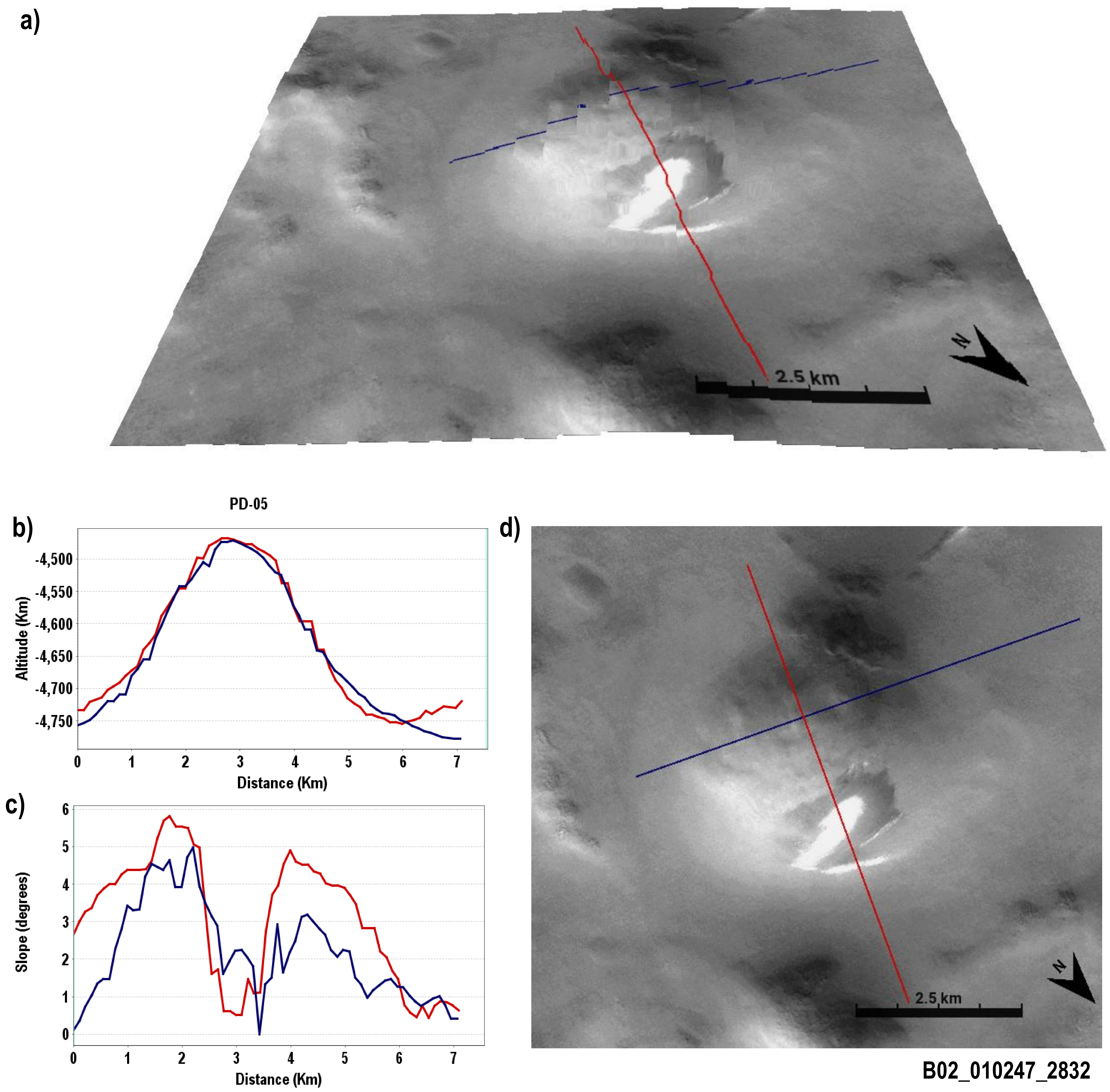
a)
2.5 km
N
b)
PD-05
Altitude (Km)
-4,500
-4,550
-4,600
-4,650
-4,700
-4,750
Distance (Km)
0 1 2 3 4 5 6 7
c)
Slope (degrees)
0 1 2 3 4 5 6
Distance (Km)
0 1 2 3 4 5 6 7
d)
2.5 km
N
B02_010247_2832

PD-06

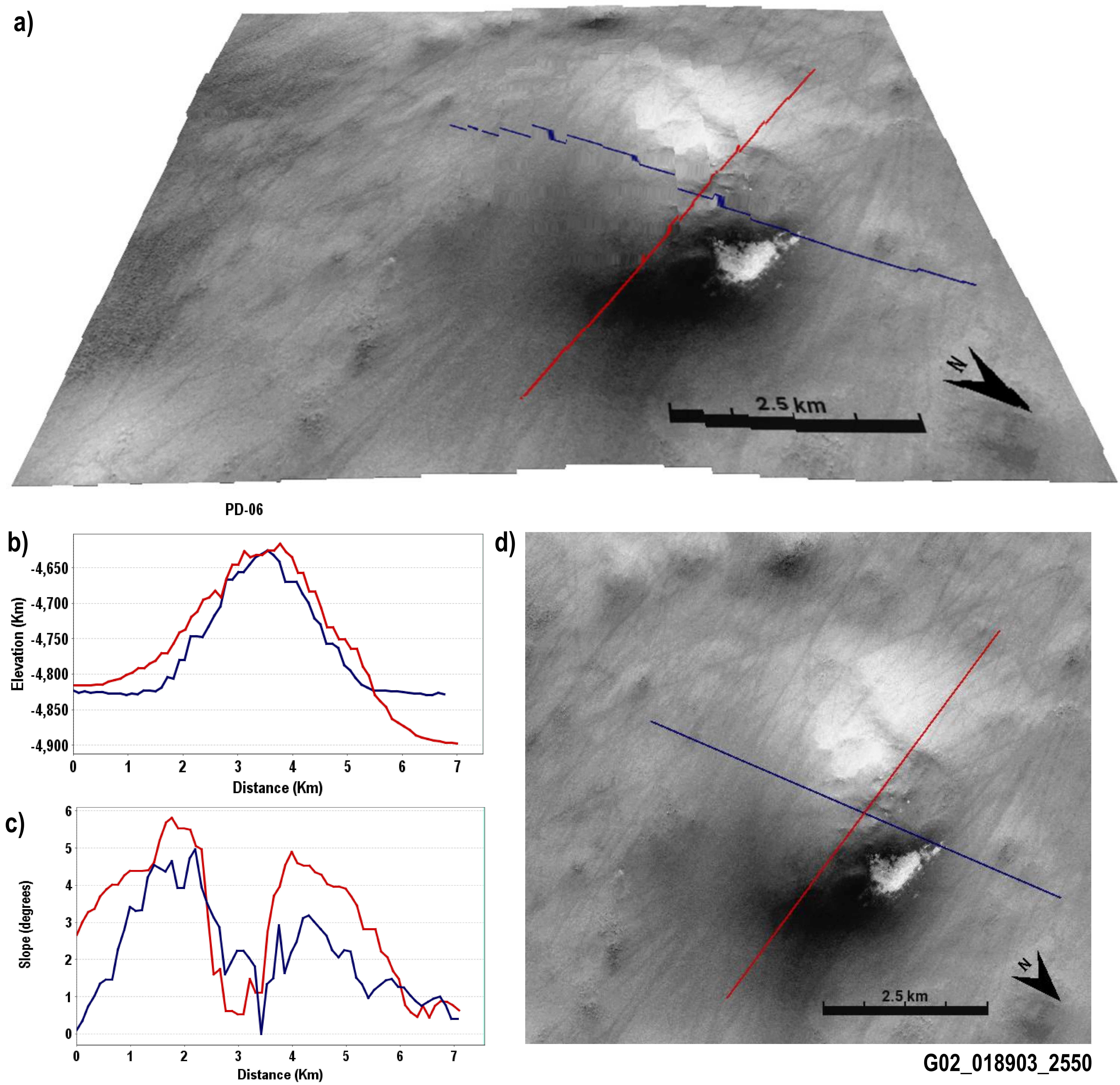

G02_018903_2550

PD-07

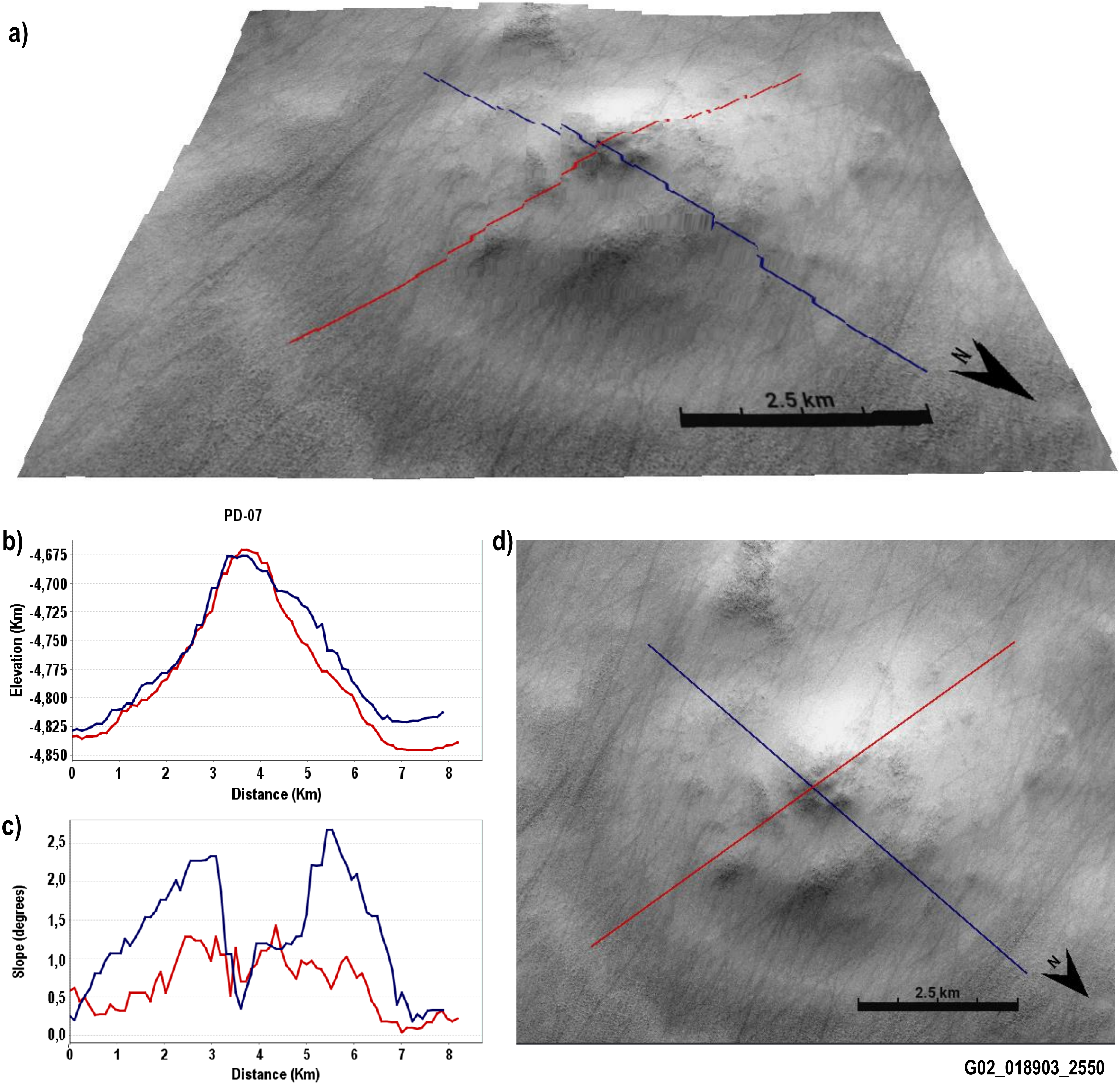

G02_018903_2550

PD-08

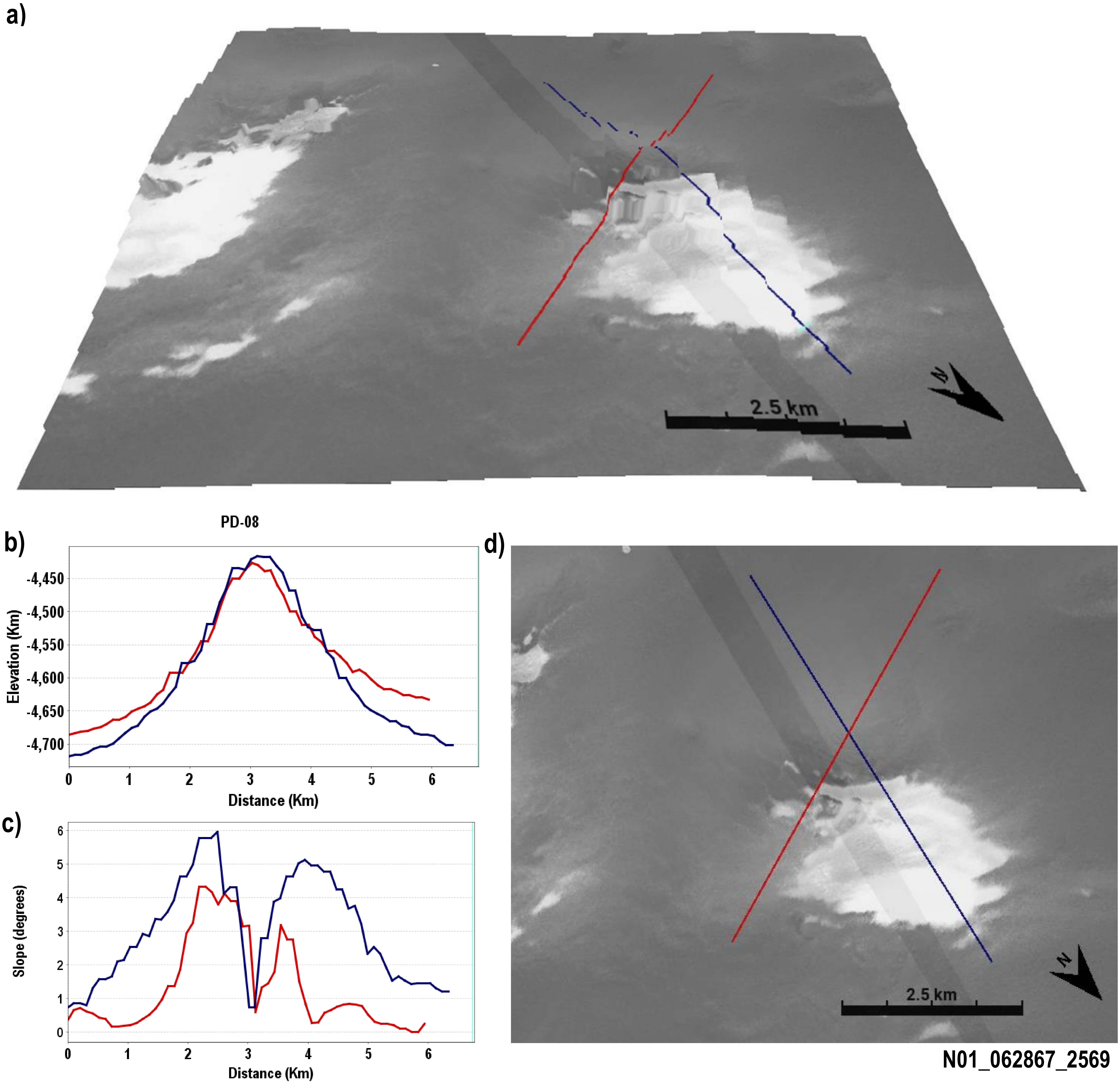

N01_062867_2569

PD-09

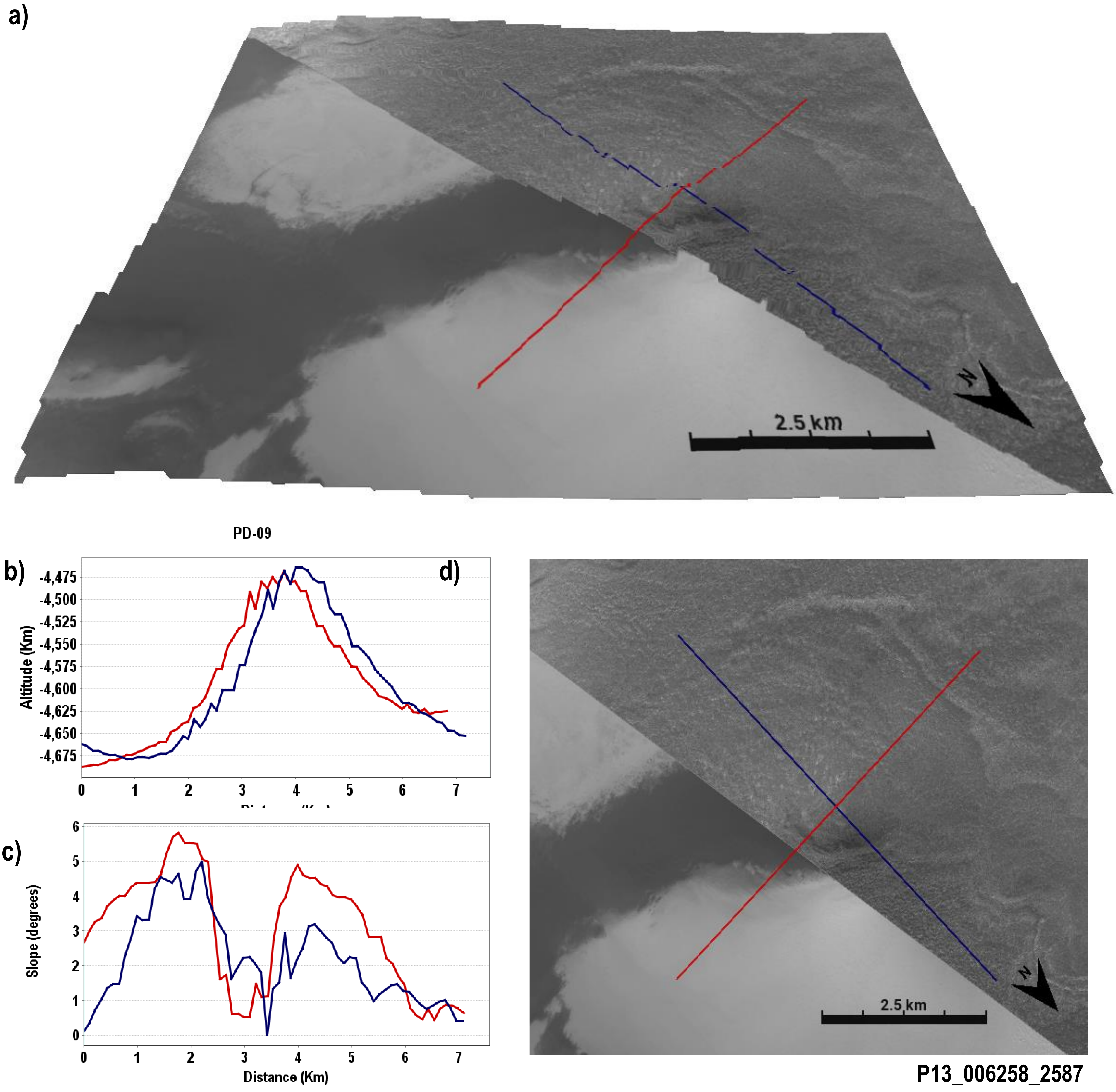

P13_006258_2587

PD-10

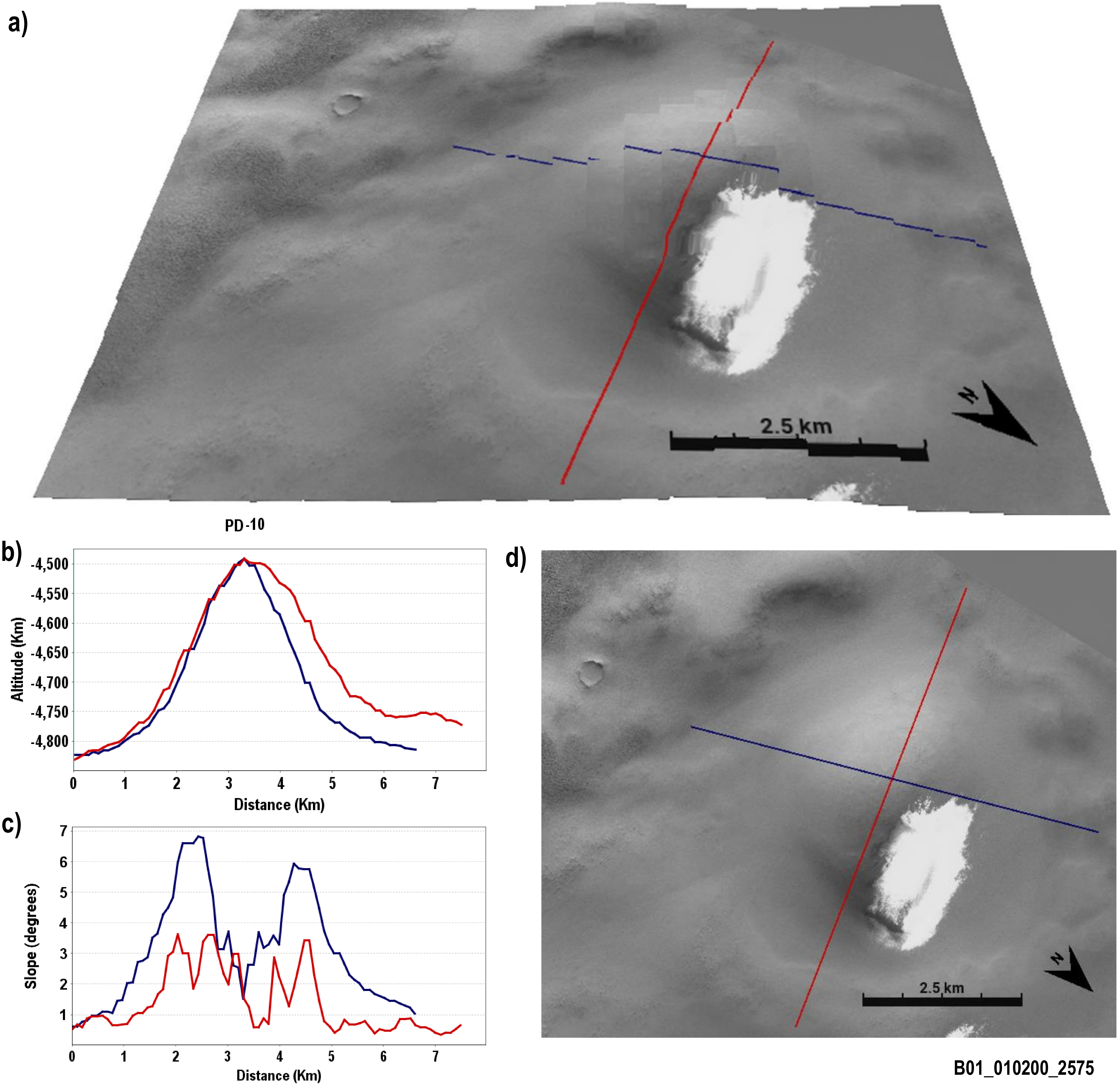
a)
2.5 km
N
PD-10
b)
Altitude (Km)
-4,500
-4,550
-4,600
-4,650
-4,700
-4,750
-4,800
Distance (Km)
0 1 2 3 4 5 6 7
c)
Slope (degrees)
1 2 3 4 5 6 7
Distance (Km)
0 1 2 3 4 5 6 7
d)
2.5 km
N
B01_010200_2575

PD-11

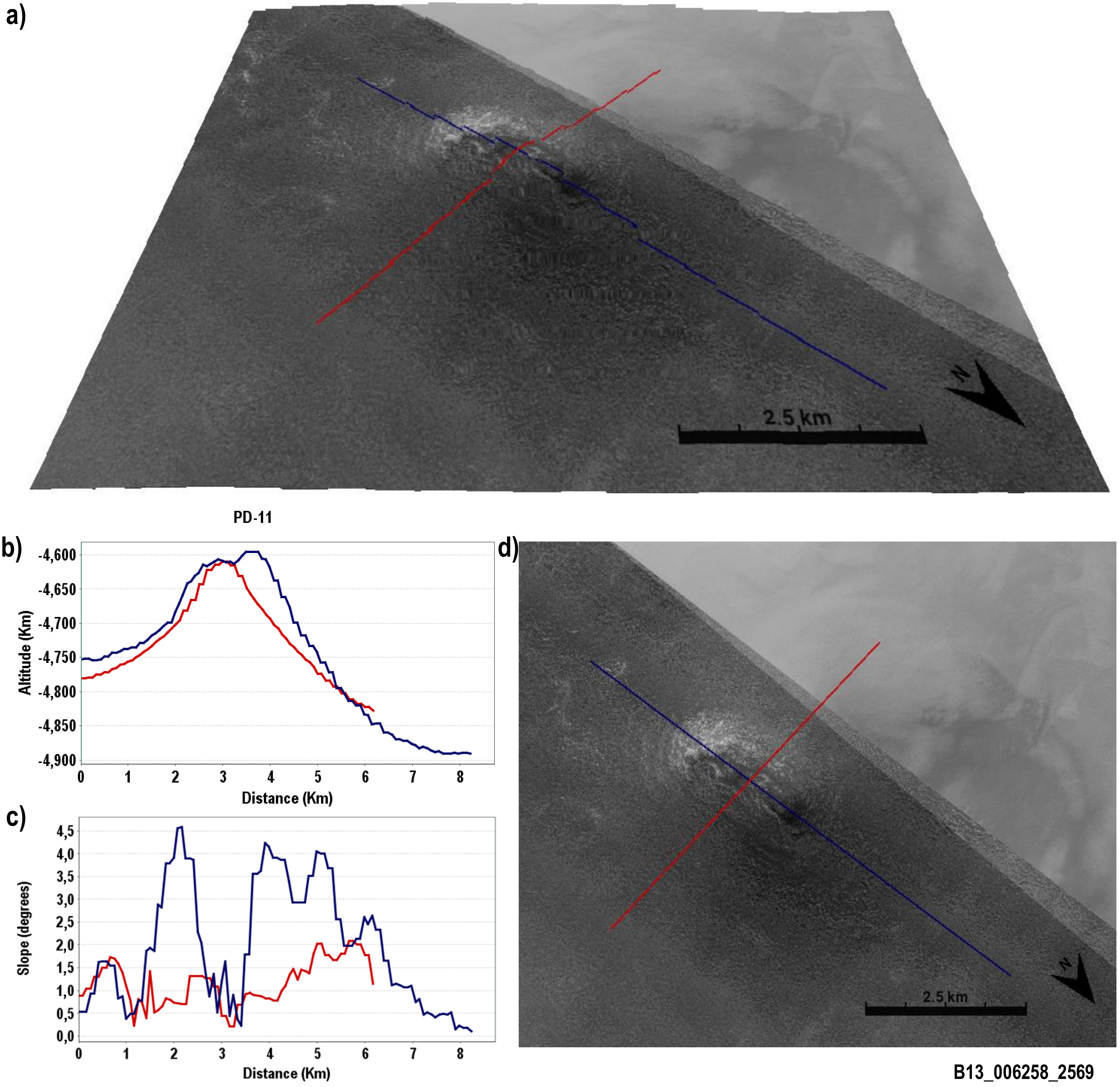
a)
2.5 km
N
PD-11
b)
Altitude (Km)
-4,600
-4,650
-4,700
-4,750
-4,800
-4,850
-4,900
Distance (Km)
0
1
2
3
4
5
6
7
8
c)
Slope (degrees)
4,5
4,0
3,5
3,0
2,5
2,0
1,5
1,0
0,5
0,0
Distance (Km)
d)
2.5 km
N
B13_006258_2569

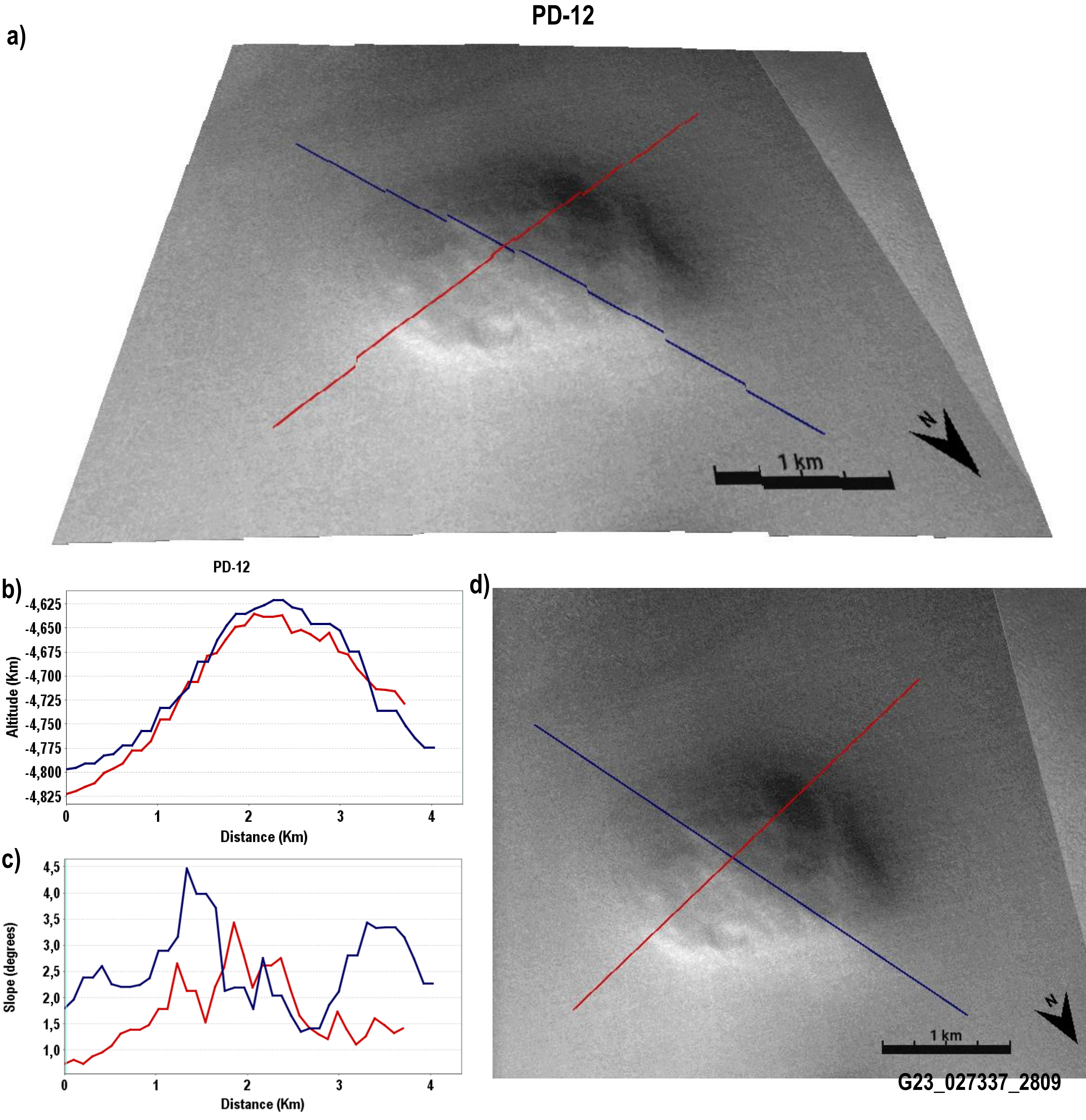

PD-12
a)
1 km
N
PD-12
b)
Altitude (Km)
-4,625
-4,650
-4,675
-4,700
-4,725
-4,750
-4,775
-4,800
-4,825
0
1
2
3
4
Distance (Km)
c)
Slope (degrees)
4,5
4,0
3,5
3,0
2,5
2,0
1,5
1,0
0
1
2
3
4
Distance (Km)
d)
1 km
N
G23_027337_2809

PD-13

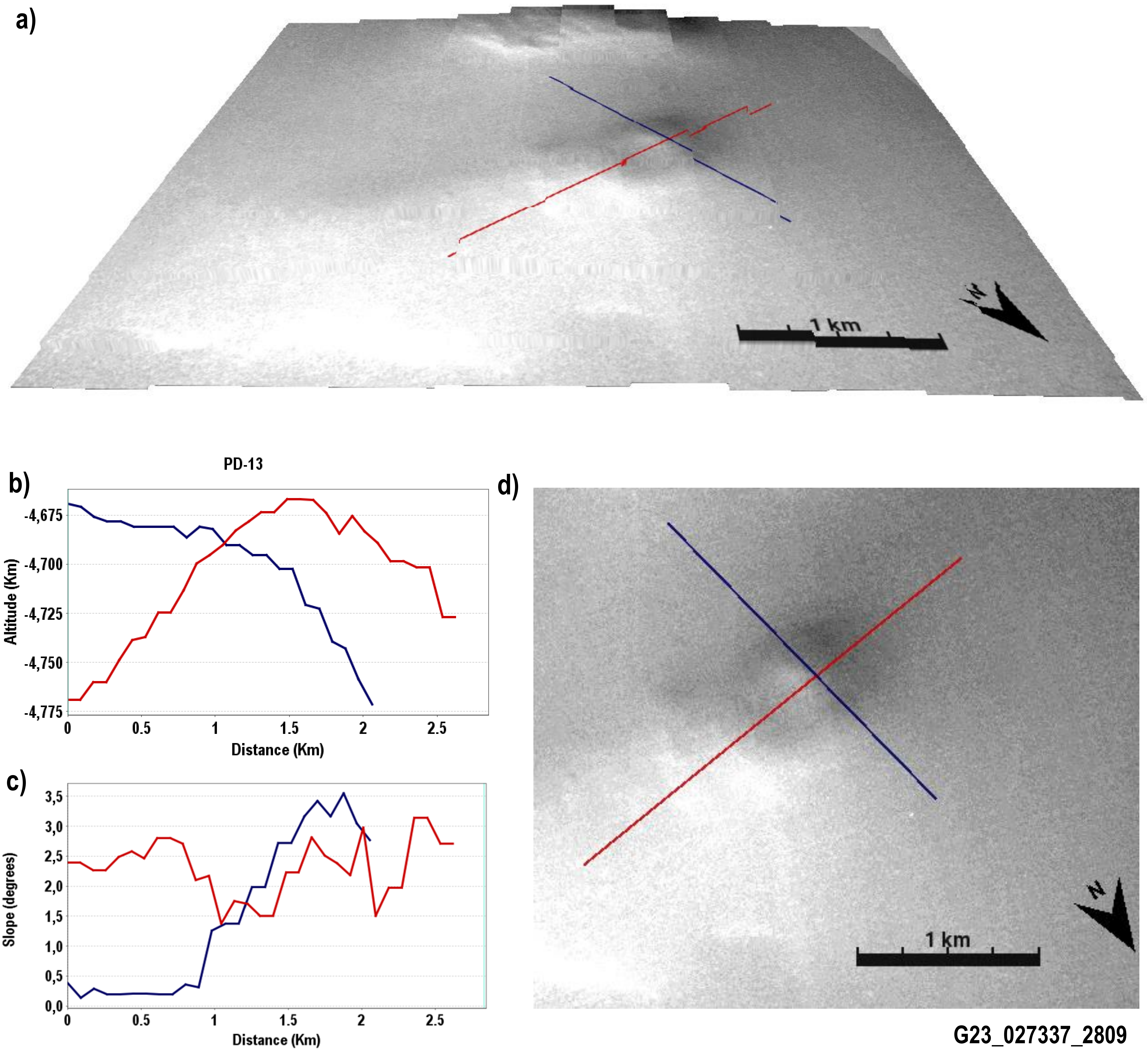
a)
1 km
N
b)
PD-13
Altitude (Km)
-4,675
-4,700
-4,725
-4,750
-4,775
0
0.5
1
1.5
2
2.5
Distance (Km)
c)
Slope (degrees)
3,5
3,0
2,5
2,0
1,5
1,0
0,5
0,0
0
0.5
1
1.5
2
2.5
Distance (Km)
d)
1 km
N
G23_027337_2809

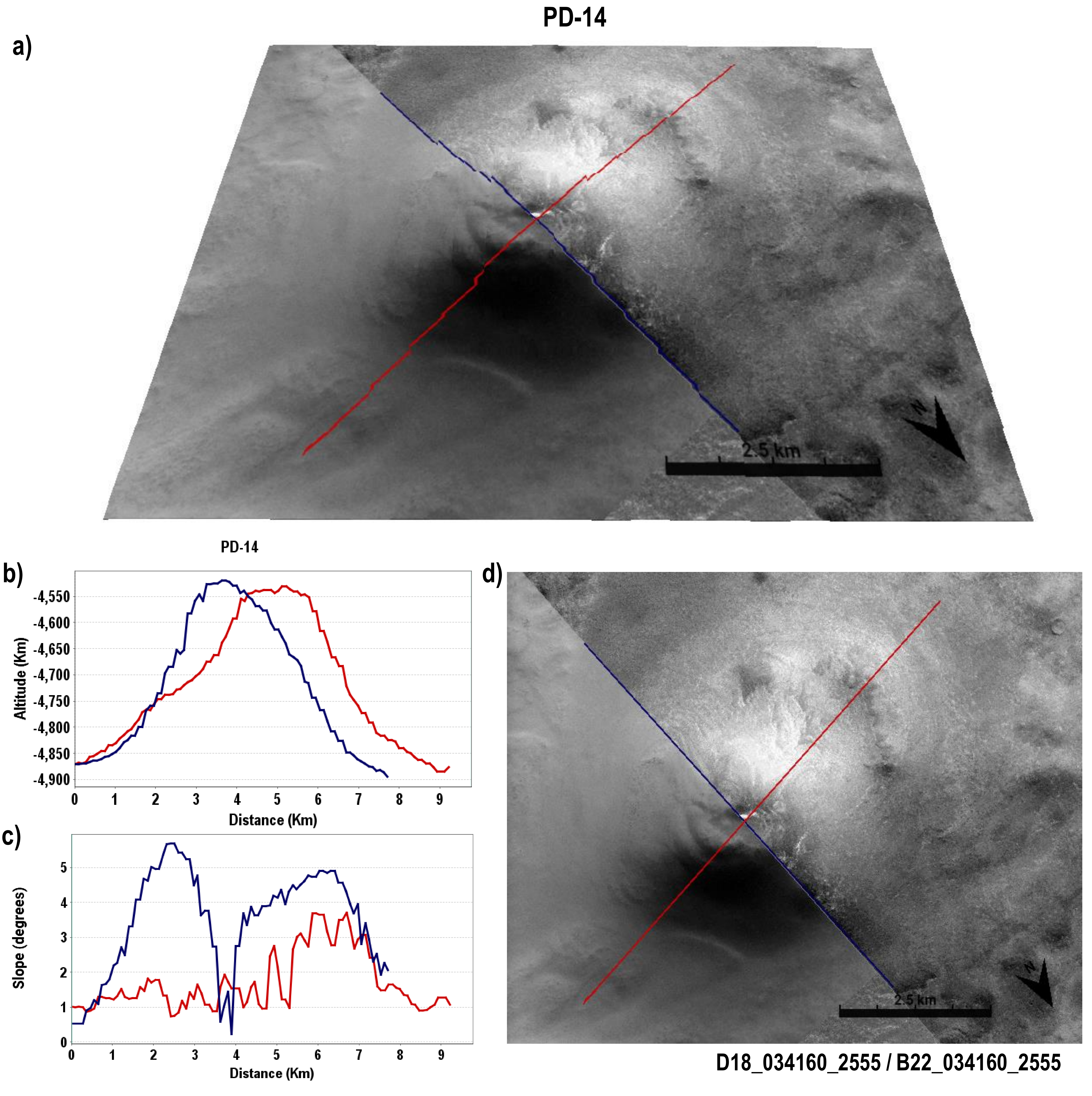
PD-14
a)
2.5 km
N
PD-14
b)
Altitude (Km)
-4,550
-4,600
-4,650
-4,700
-4,750
-4,800
-4,850
-4,900
0 1 2 3 4 5 6 7 8 9
Distance (Km)
c)
Slope (degrees)
0 1 2 3 4 5
0 1 2 3 4 5 6 7 8 9
Distance (Km)
d)
2.5 km
N
D18_034160_2555 / B22_034160_2555

PD-15

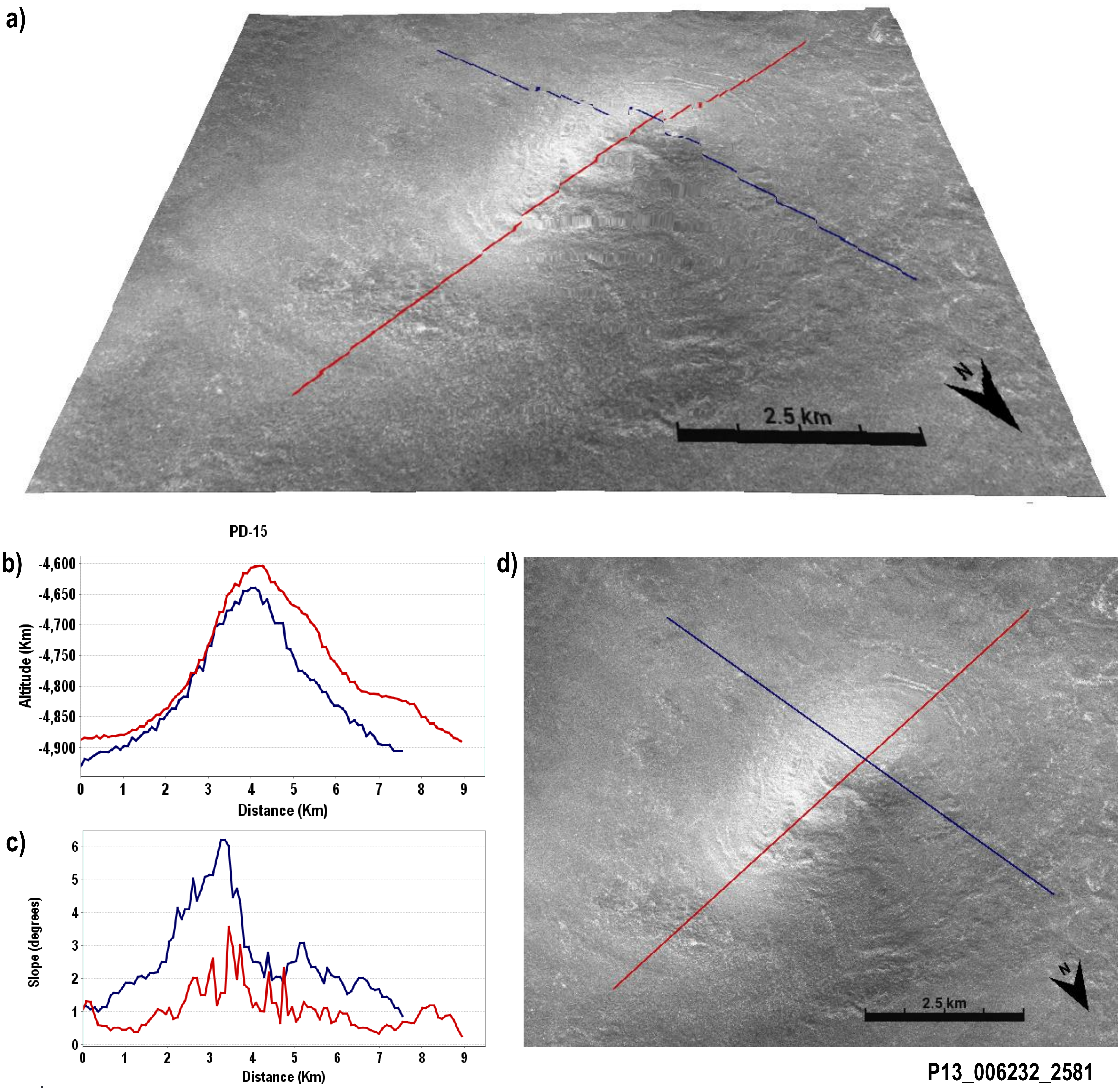
a)
2.5 km
N
b)
PD-15
-4,600
-4,650
-4,700
-4,750
-4,800
-4,850
-4,900
Altitude (Km)
0 1 2 3 4 5 6 7 8 9
Distance (Km)
c)
6
5
4
3
2
1
0
Slope (degrees)
0 1 2 3 4 5 6 7 8 9
Distance (Km)
d)
2.5 km
N
P13_006232_2581

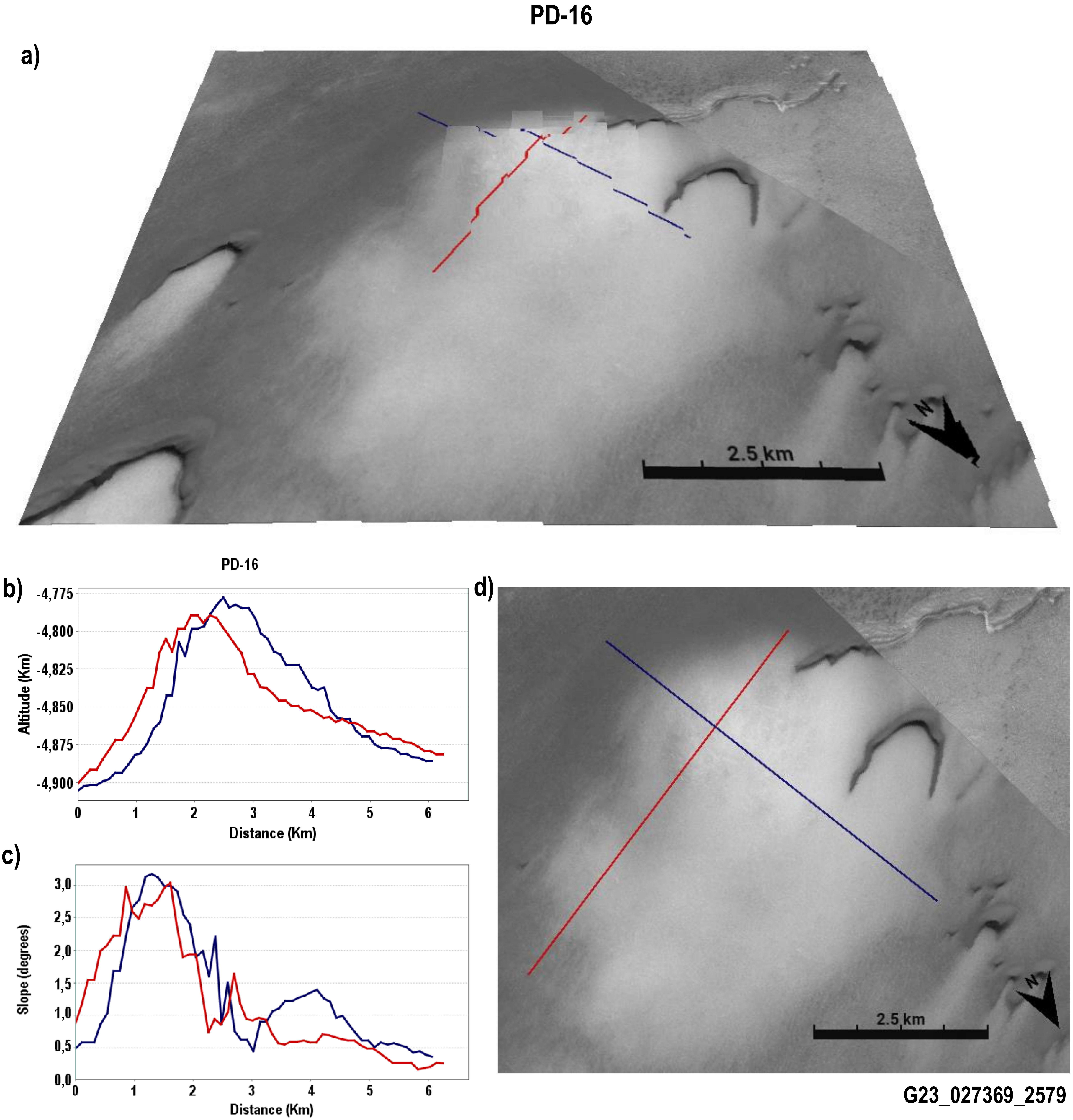

PD-16
a)
2.5 km
N
PD-16
b)
Altitude (Km)
-4,775
-4,800
-4,825
-4,850
-4,875
-4,900
0 1 2 3 4 5 6
Distance (Km)
c)
Slope (degrees)
3,0
2,5
2,0
1,5
1,0
0,5
0,0
0 1 2 3 4 5 6
Distance (Km)
d)
2.5 km
N
G23_027369_2579

PD-17

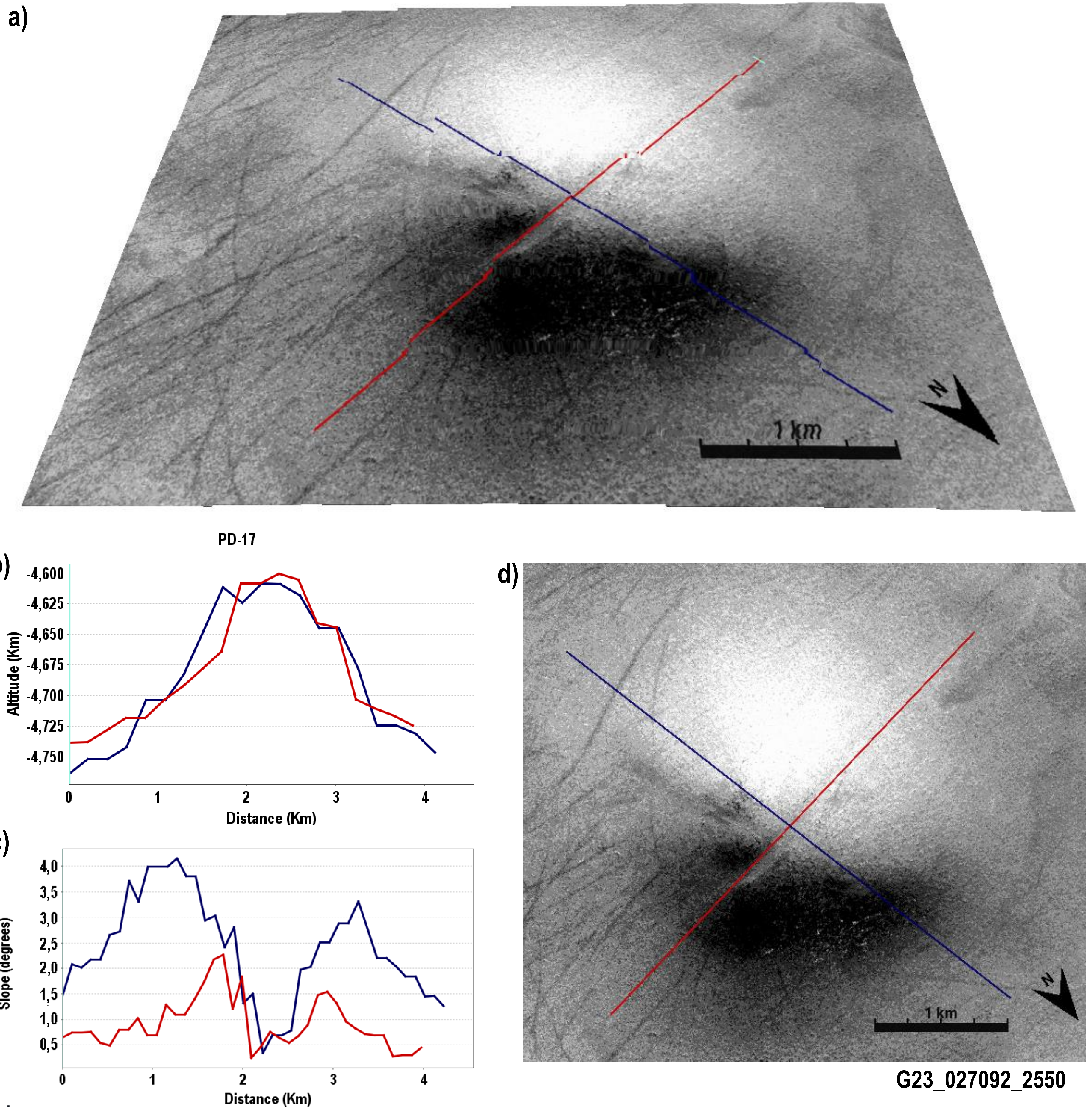
a)
1 km
N
PD-17
b)
Altitude (Km)
-4,600
-4,625
-4,650
-4,675
-4,700
-4,725
-4,750
0
1
2
3
4
Distance (Km)
c)
Slope (degrees)
4,0
3,5
3,0
2,5
2,0
1,5
1,0
0,5
0
1
2
3
4
Distance (Km)
d)
1 km
N
G23_027092_2550

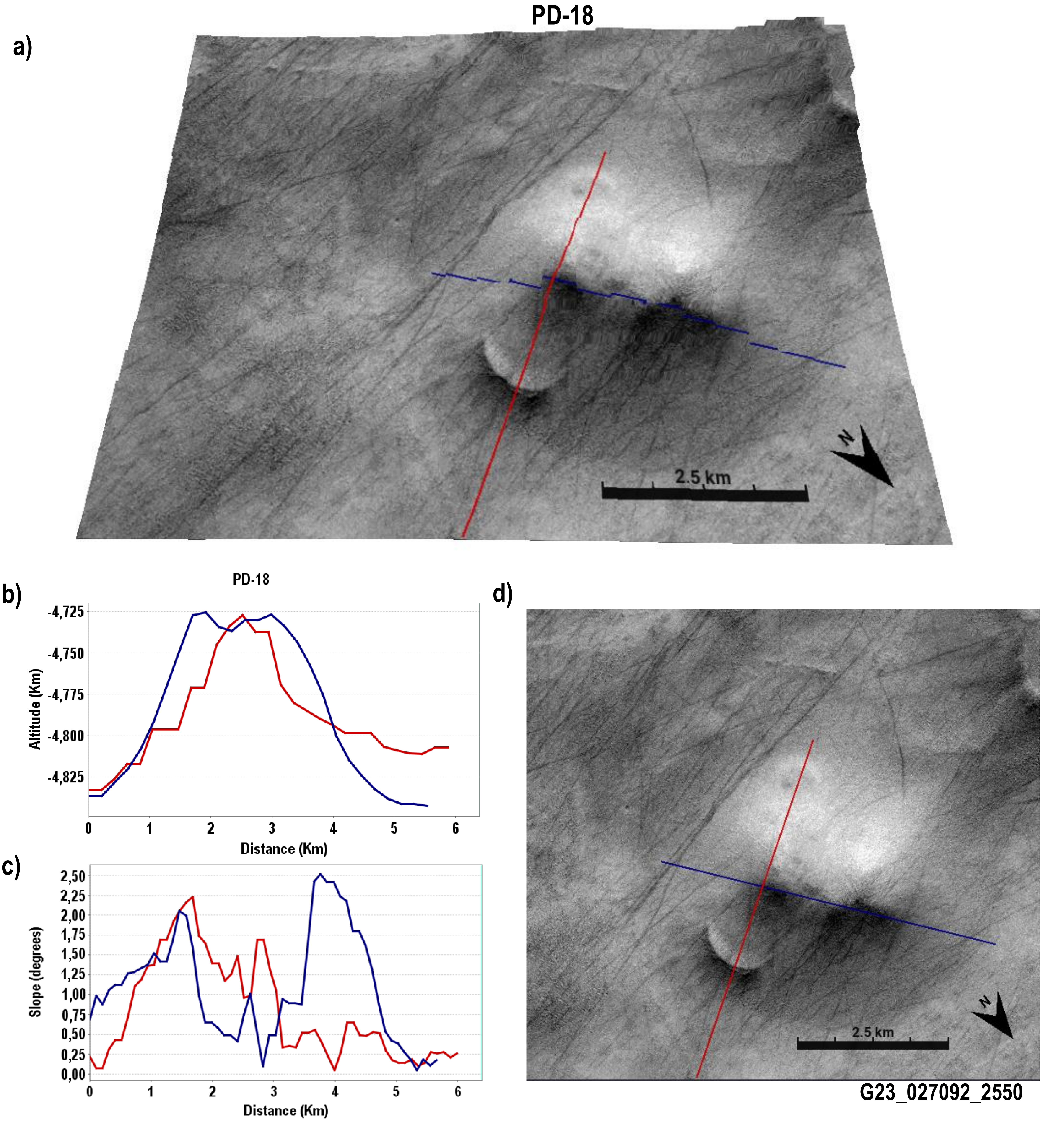
PD-18
a)
2.5 km
N
b)
PD-18
Altitude (Km)
-4,725
-4,750
-4,775
-4,800
-4,825
Distance (Km)
0
1
2
3
4
5
6
c)
Slope (degrees)
2,50
2,25
2,00
1,75
1,50
1,25
1,00
0,75
0,50
0,25
0,00
Distance (Km)
d)
2.5 km
N
G23_027092_2550

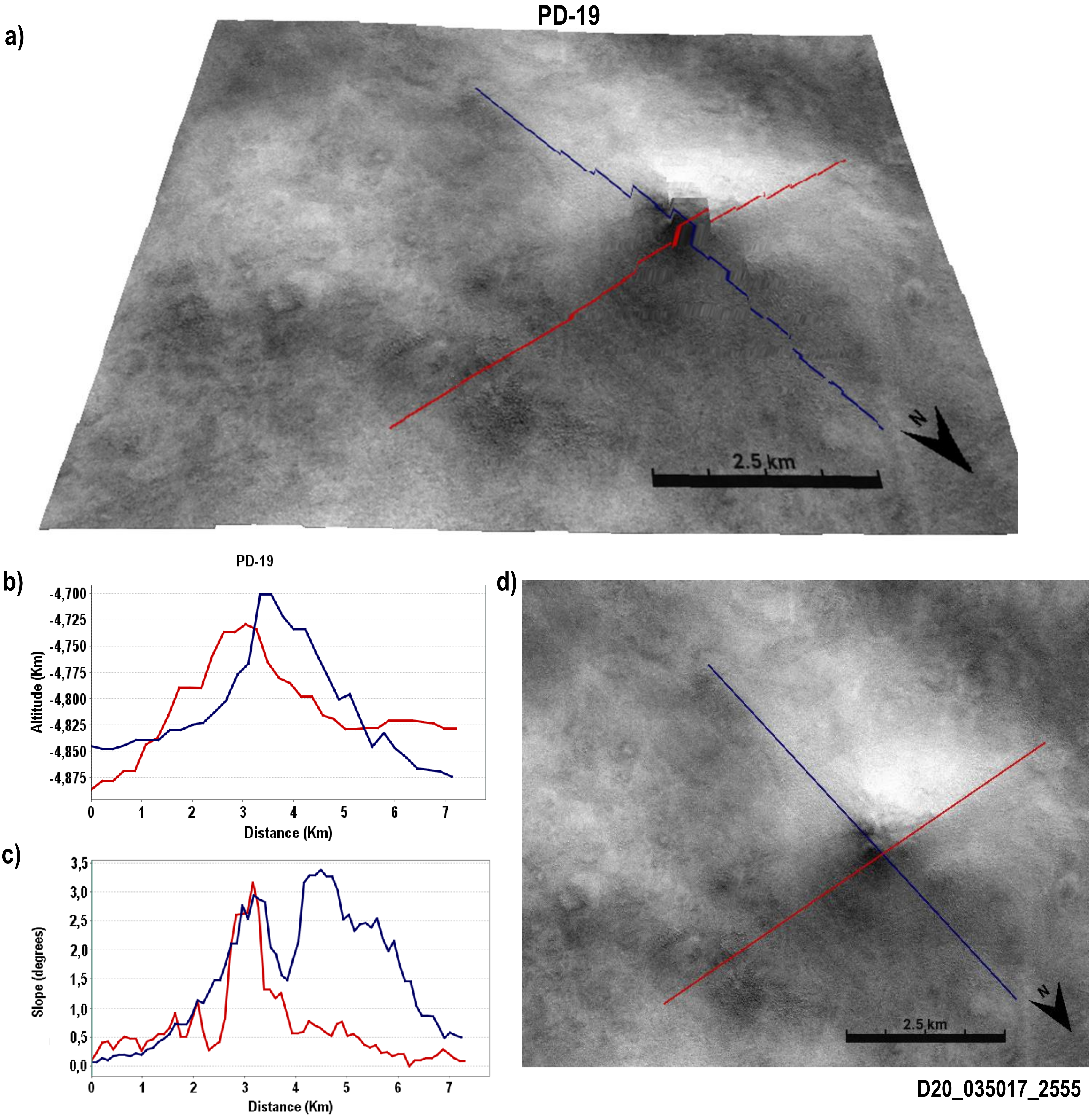
PD-19
a)
2.5 km
N
PD-19
b)
Altitude (Km)
-4,700
-4,725
-4,750
-4,775
-4,800
-4,825
-4,850
-4,875
Distance (Km)
0
1
2
3
4
5
6
7
c)
Slope (degrees)
3,5
3,0
2,5
2,0
1,5
1,0
0,5
0,0
Distance (Km)
d)
2.5 km
N
D20_035017_2555

PD-20

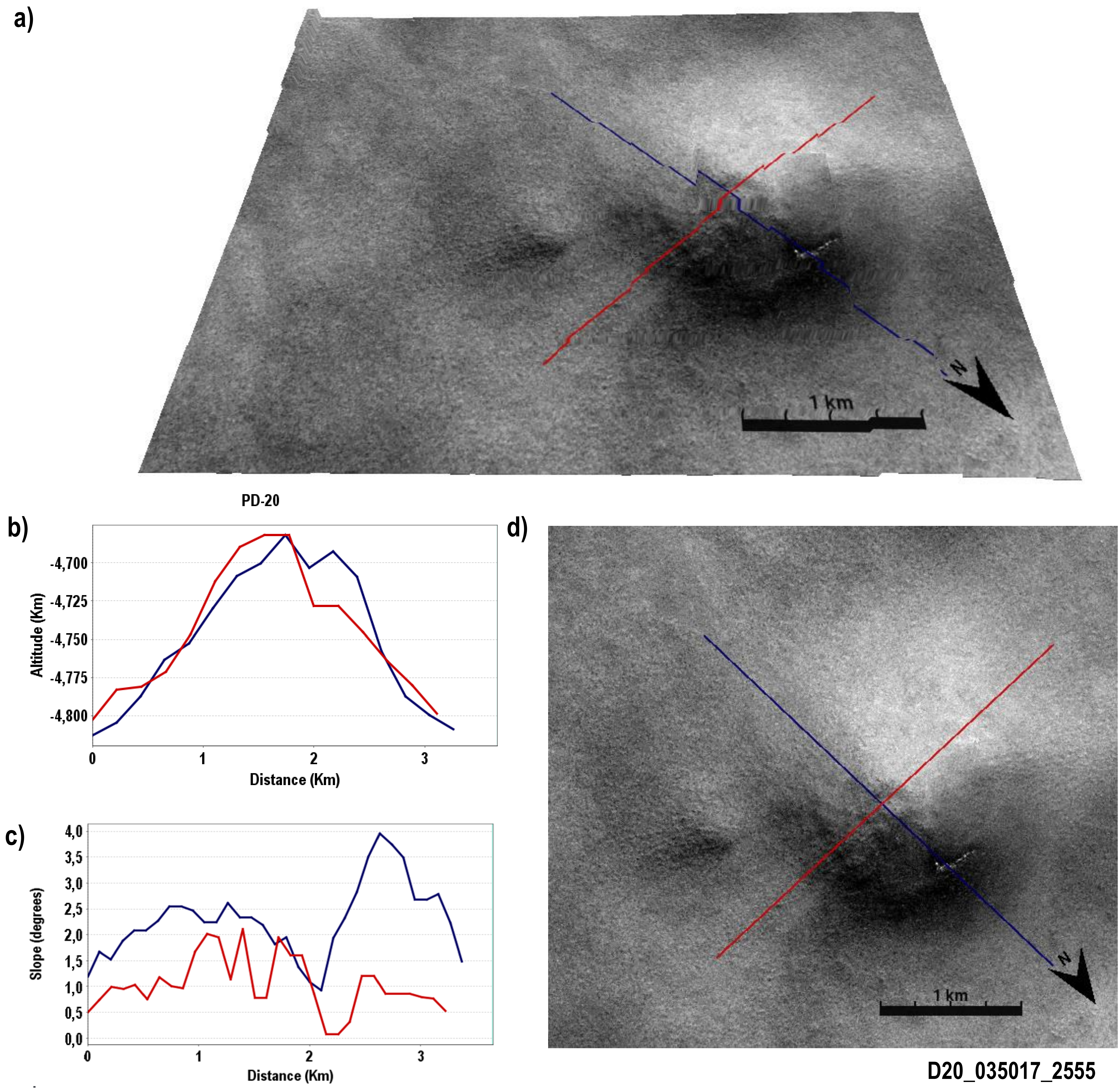

D20_035017_2555

PD-21

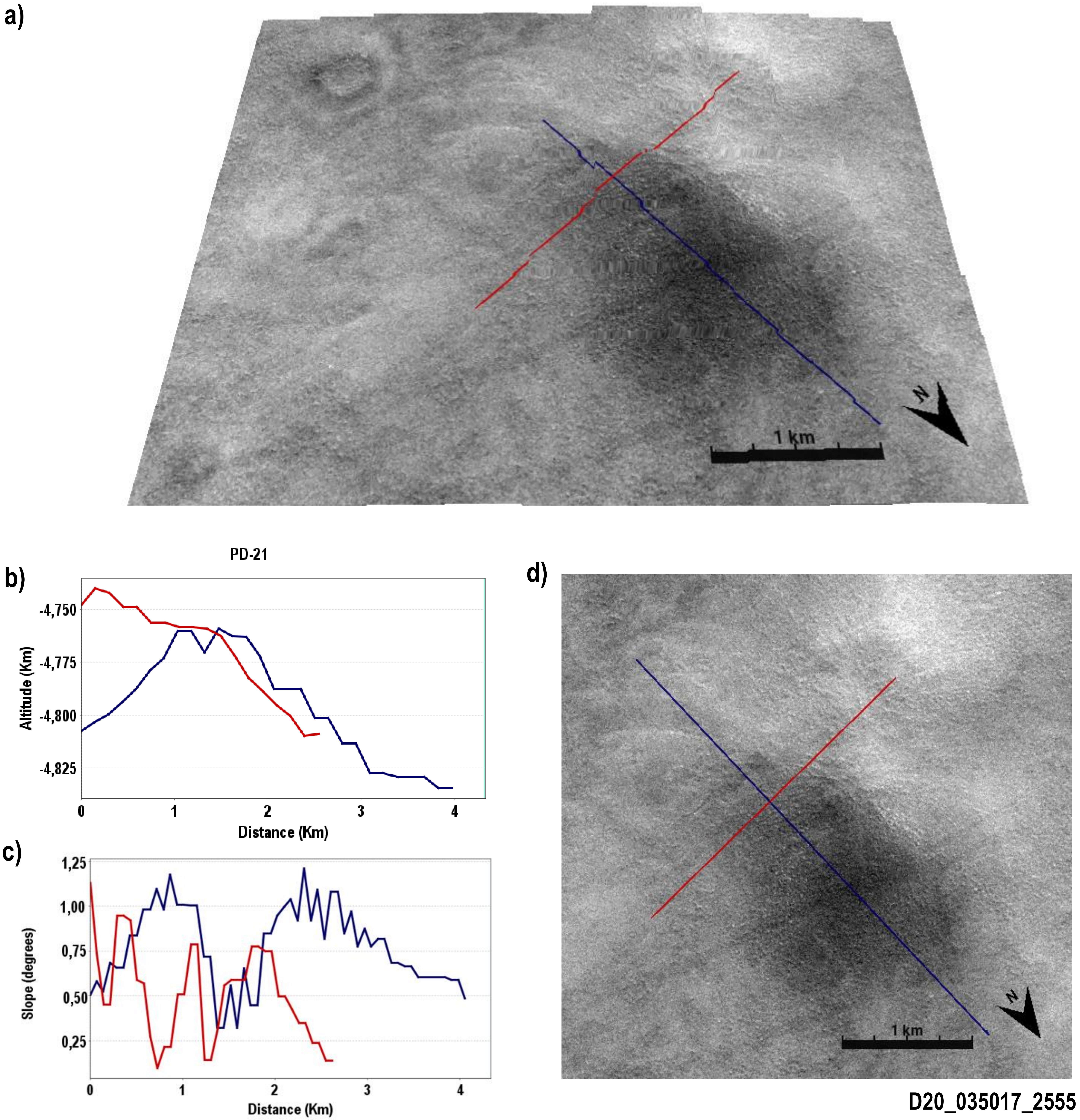
a)
1 km
N
b)
PD-21
Altitude (Km)
-4,750
-4,775
-4,800
-4,825
0
1
2
3
4
Distance (Km)
c)
Slope (degrees)
1,25
1,00
0,75
0,50
0,25
0
1
2
3
4
Distance (Km)
d)
1 km
N
D20_035017_2555

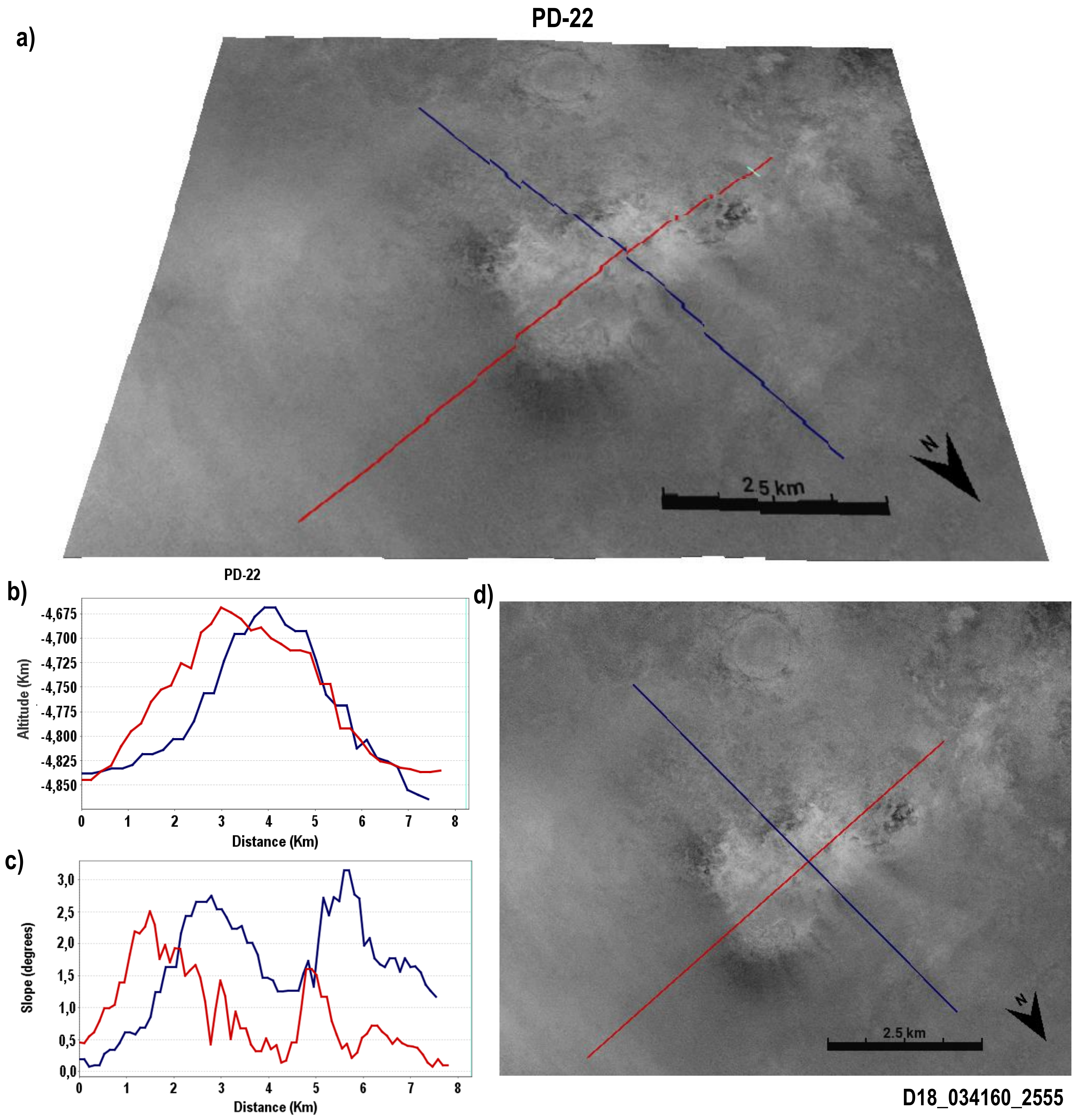
PD-22
a)
2.5 km
N
b)
PD-22
Altitude (Km)
-4,675
-4,700
-4,725
-4,750
-4,775
-4,800
-4,825
-4,850
0 1 2 3 4 5 6 7 8
Distance (Km)
c)
Slope (degrees)
3,0
2,5
2,0
1,5
1,0
0,5
0,0
0 1 2 3 4 5 6 7 8
Distance (Km)
d)
2.5 km
N
D18_034160_2555

PD-23

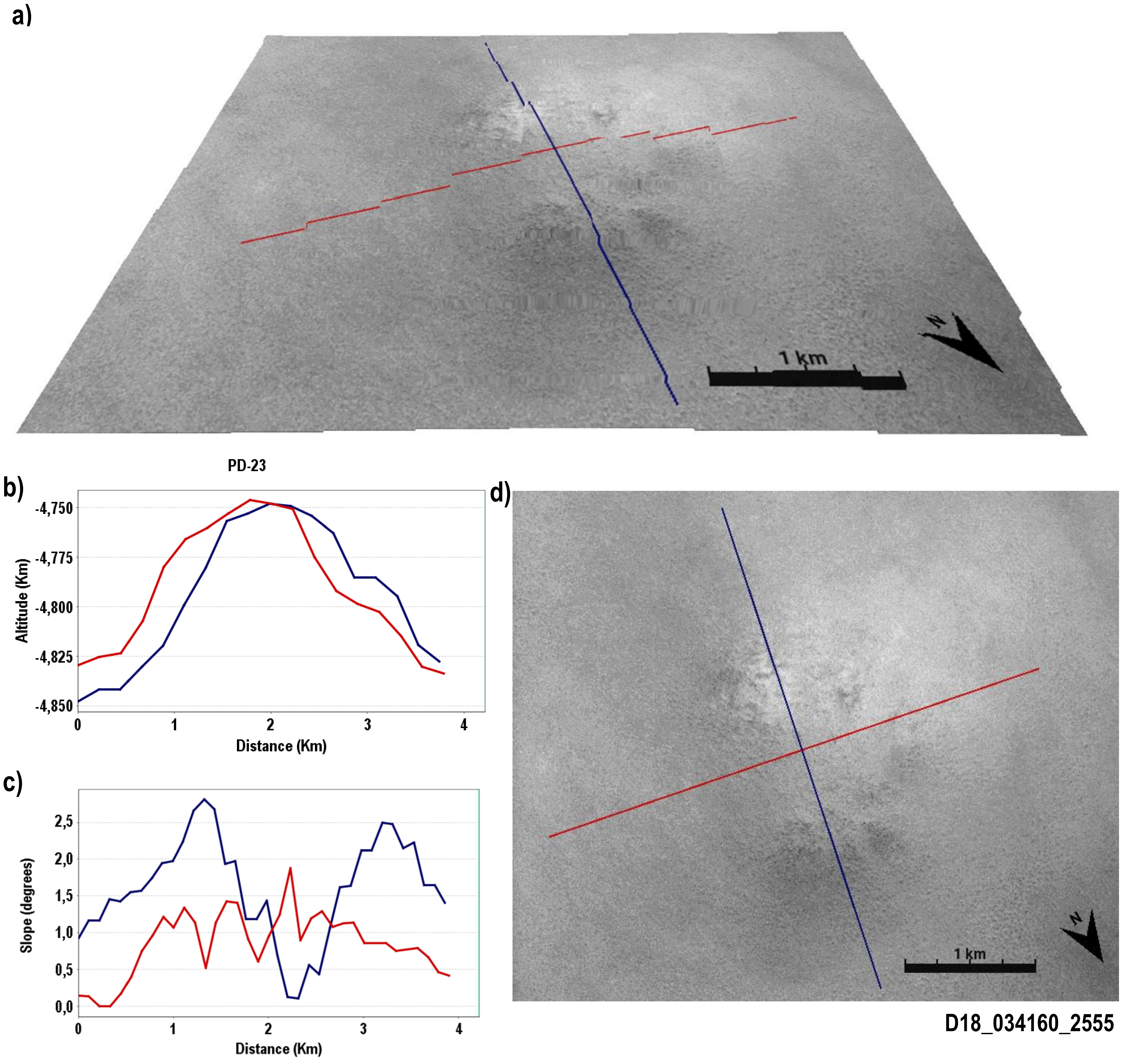

D18_034160_2555

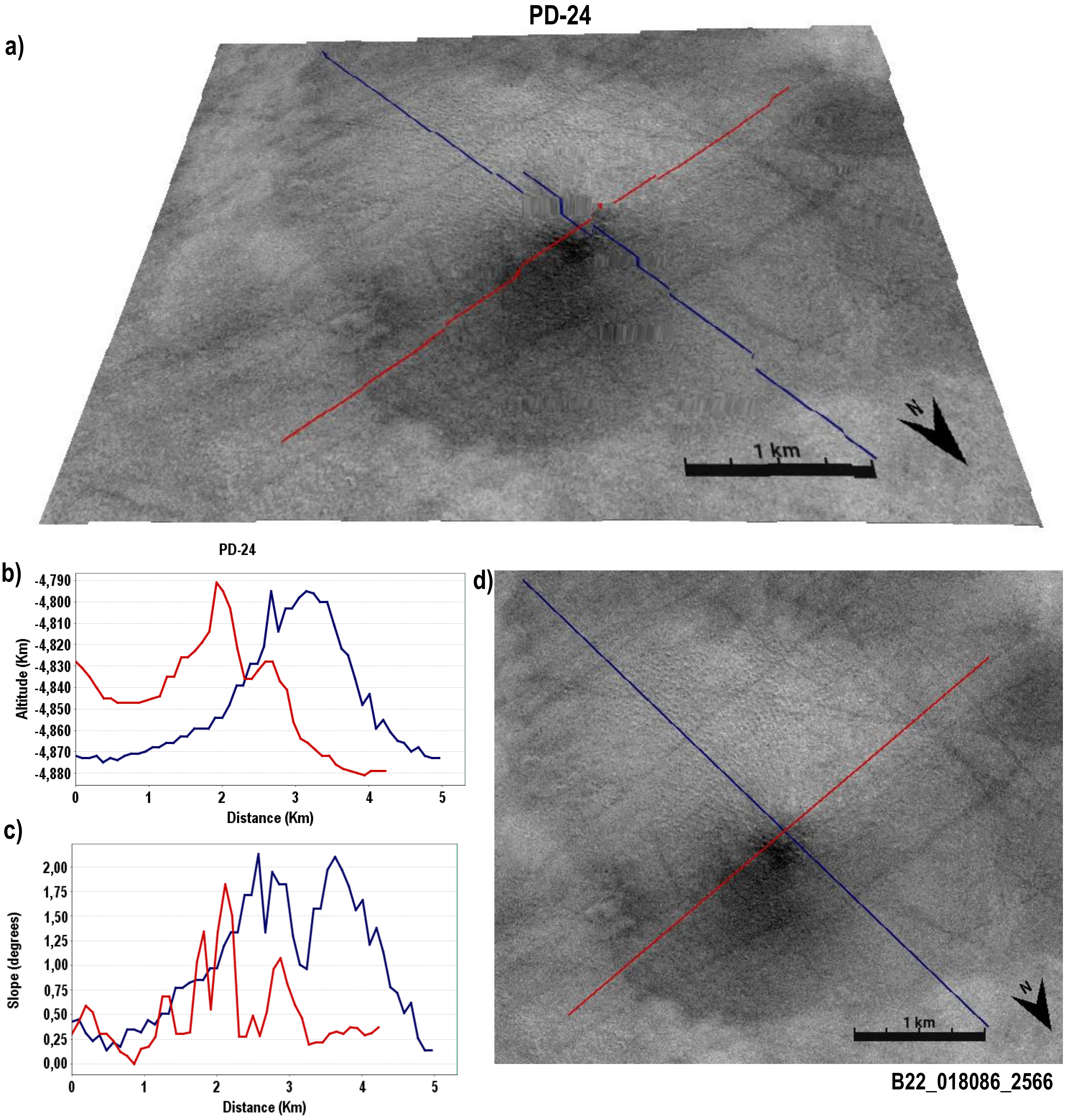
PD-24
a)
1 km
N
PD-24
b)
Altitude (Km)
-4,790
-4,800
-4,810
-4,820
-4,830
-4,840
-4,850
-4,860
-4,870
-4,880
0
1
2
3
4
5
Distance (Km)
c)
Slope (degrees)
2,00
1,75
1,50
1,25
1,00
0,75
0,50
0,25
0,00
0
1
2
3
4
5
Distance (Km)
d)
1 km
N
B22_018086_2566

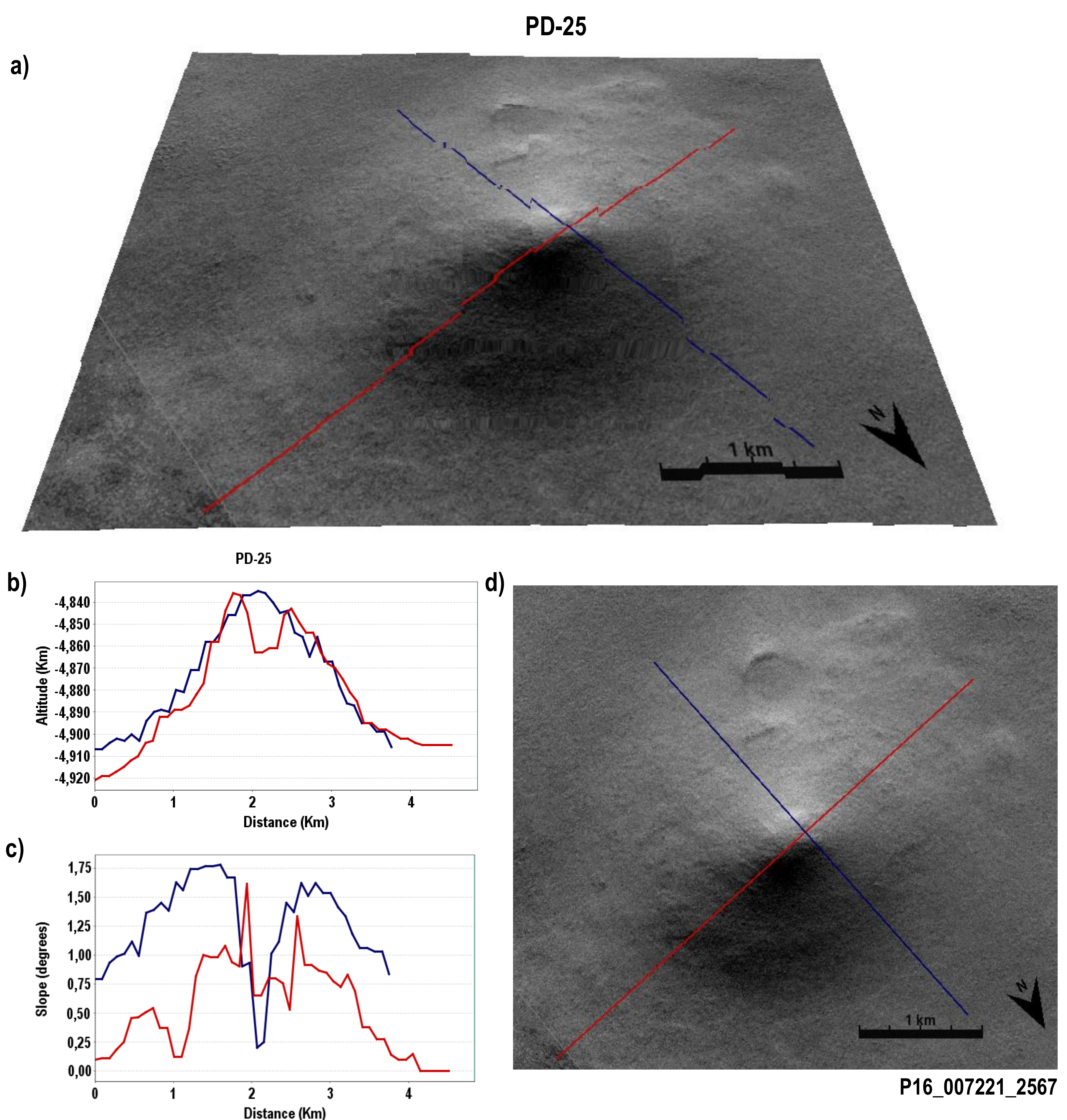
PD-25
a)
1 km
N
b)
PD-25
Altitude (Km)
-4,840
-4,850
-4,860
-4,870
-4,880
-4,890
-4,900
-4,910
-4,920
0
1
2
3
4
Distance (Km)
c)
Slope (degrees)
1,75
1,50
1,25
1,00
0,75
0,50
0,25
0,00
0
1
2
3
4
Distance (Km)
d)
1 km
N
P16_007221_2567

PD-26

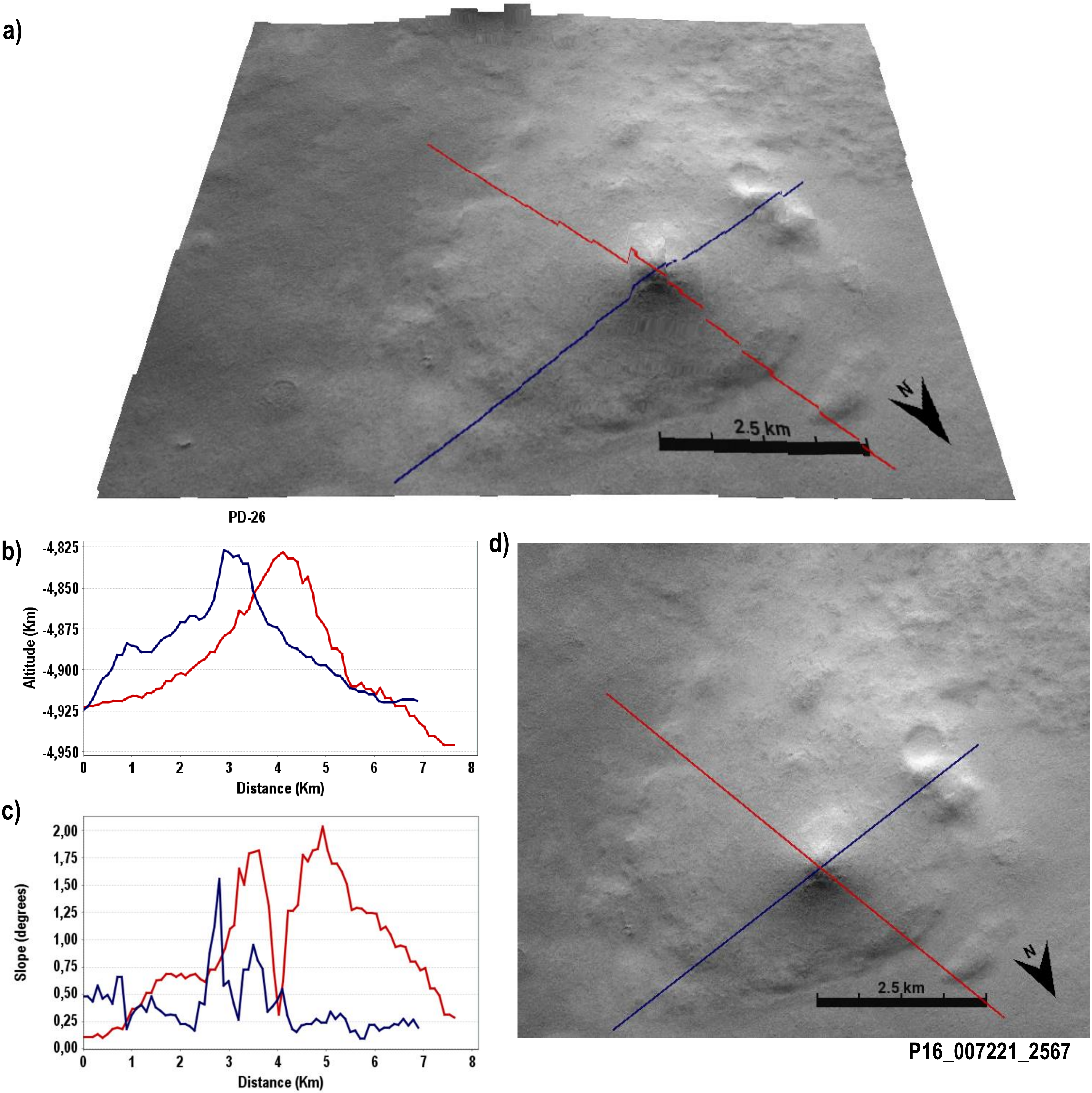
a)
2.5 km
N
PD-26
b)
-4,825
-4,850
-4,875
-4,900
-4,925
-4,950
Altitude (Km)
0
1
2
3
4
5
6
7
8
Distance (Km)
c)
2,00
1,75
1,50
1,25
1,00
0,75
0,50
0,25
0,00
Slope (degrees)
Distance (Km)
d)
2.5 km
N
P16_007221_2567

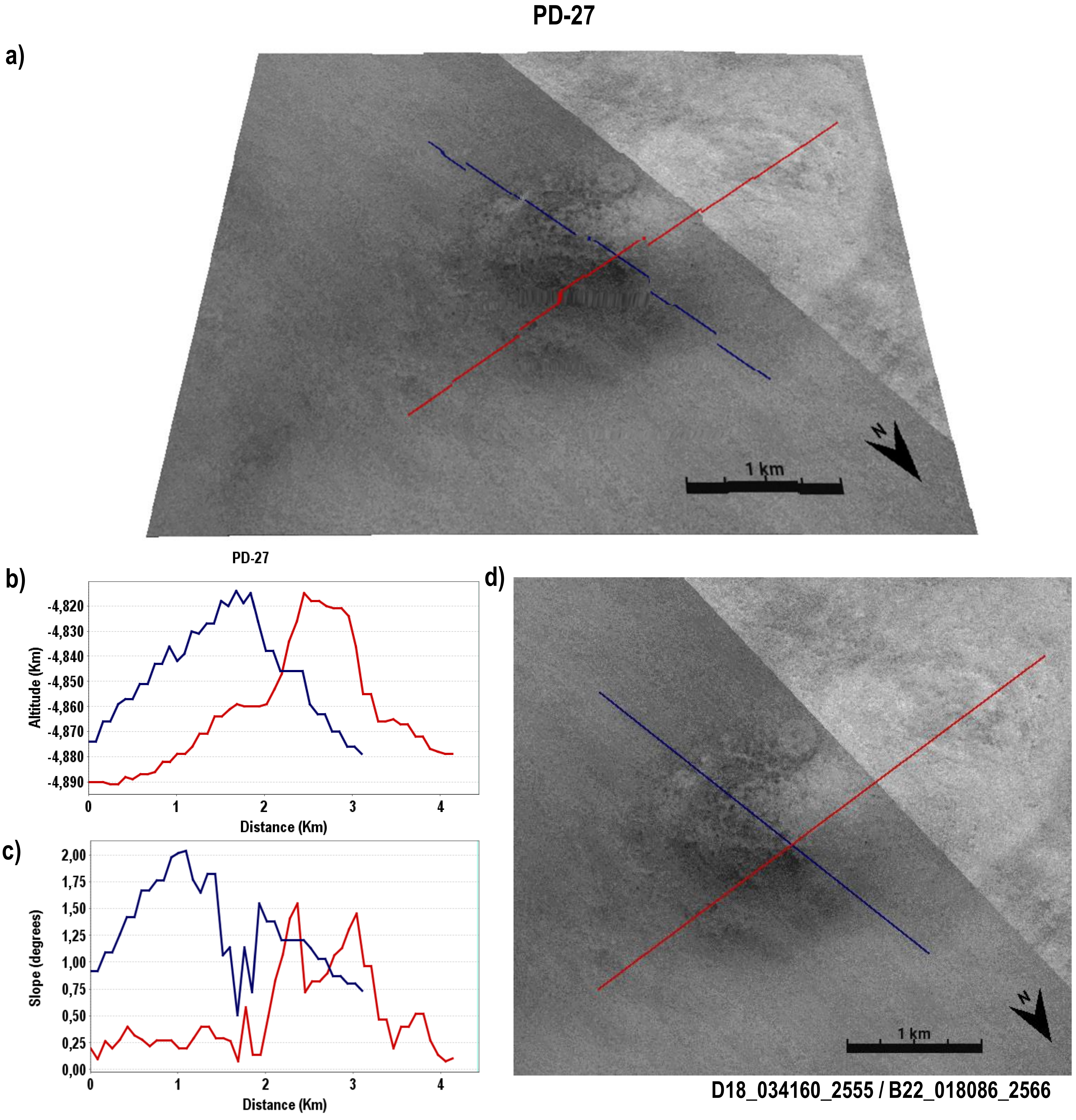
PD-27
a)
1 km
N
PD-27
b)
-4,820
-4,830
-4,840
-4,850
-4,860
-4,870
-4,880
-4,890
Altitude (Km)
0
1
2
3
4
Distance (Km)
c)
2,00
1,75
1,50
1,25
1,00
0,75
0,50
0,25
0,00
Slope (degrees)
0
1
2
3
4
Distance (Km)
d)
1 km
N
D18_034160_2555 / B22_018086_2566

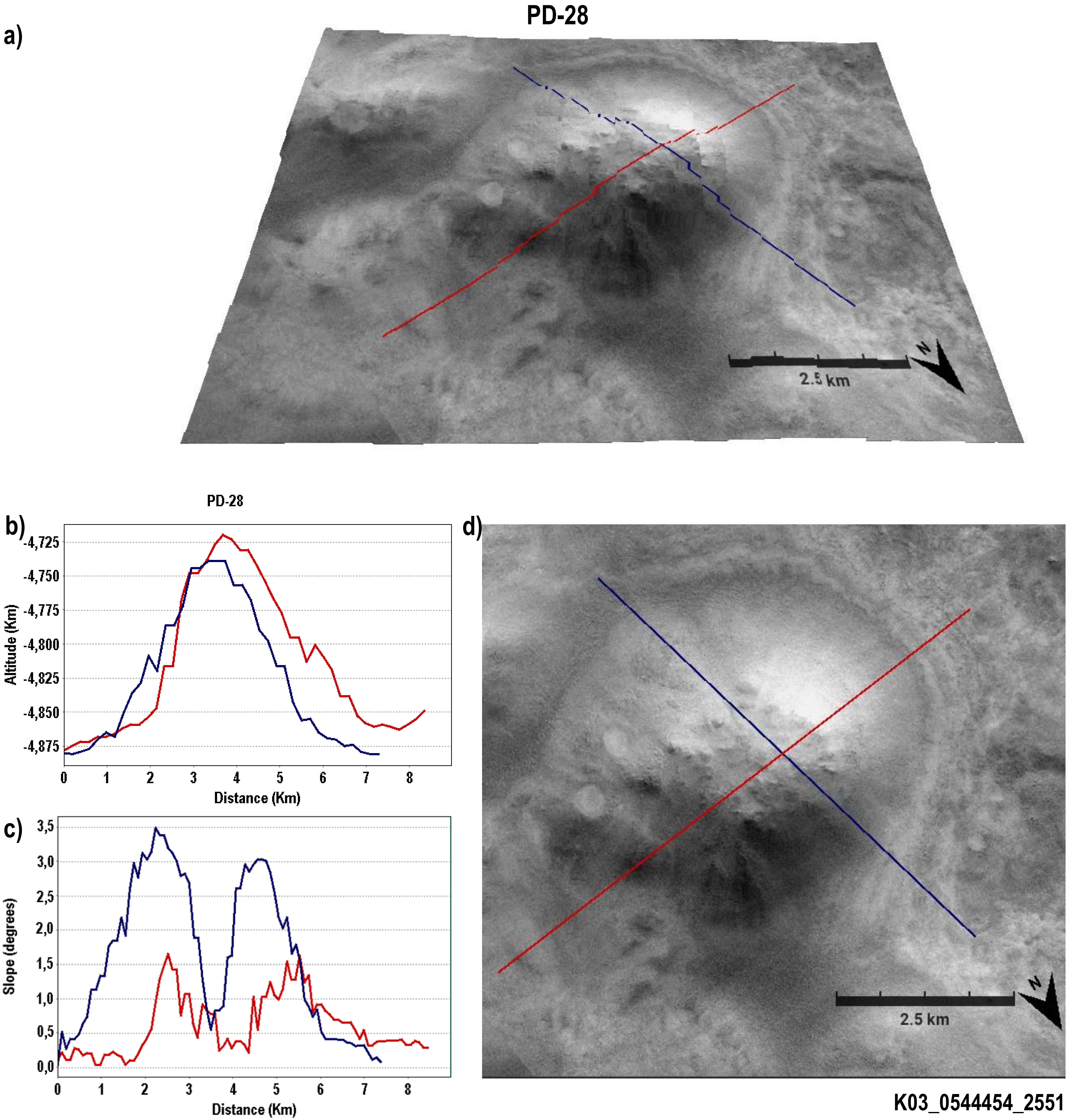
PD-28
a)
2.5 km
N
PD-28
b)
Altitude (Km)
-4,725
-4,750
-4,775
-4,800
-4,825
-4,850
-4,875
Distance (Km)
0
1
2
3
4
5
6
7
8
c)
Slope (degrees)
3,5
3,0
2,5
2,0
1,5
1,0
0,5
0,0
Distance (Km)
d)
2.5 km
N
K03_0544454_2551

PD-29

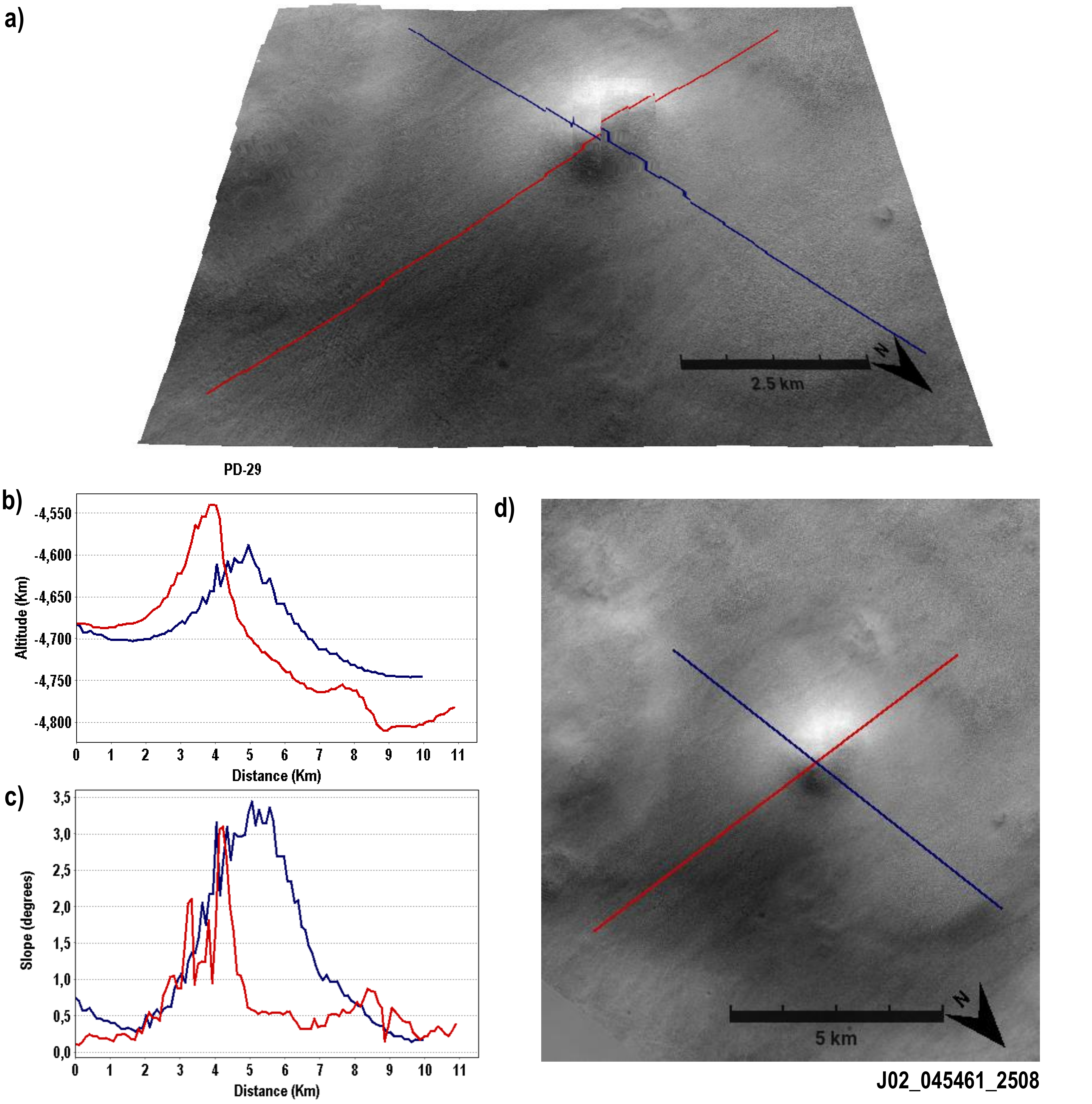
a)
2.5 km
N
PD-29
b)
Altitude (Km)
-4,550
-4,600
-4,650
-4,700
-4,750
-4,800
0 1 2 3 4 5 6 7 8 9 10 11
Distance (Km)
c)
Slope (degrees)
3,5
3,0
2,5
2,0
1,5
1,0
0,5
0,0
0 1 2 3 4 5 6 7 8 9 10 11
Distance (Km)
d)
5 km
N
J02_045461_2508

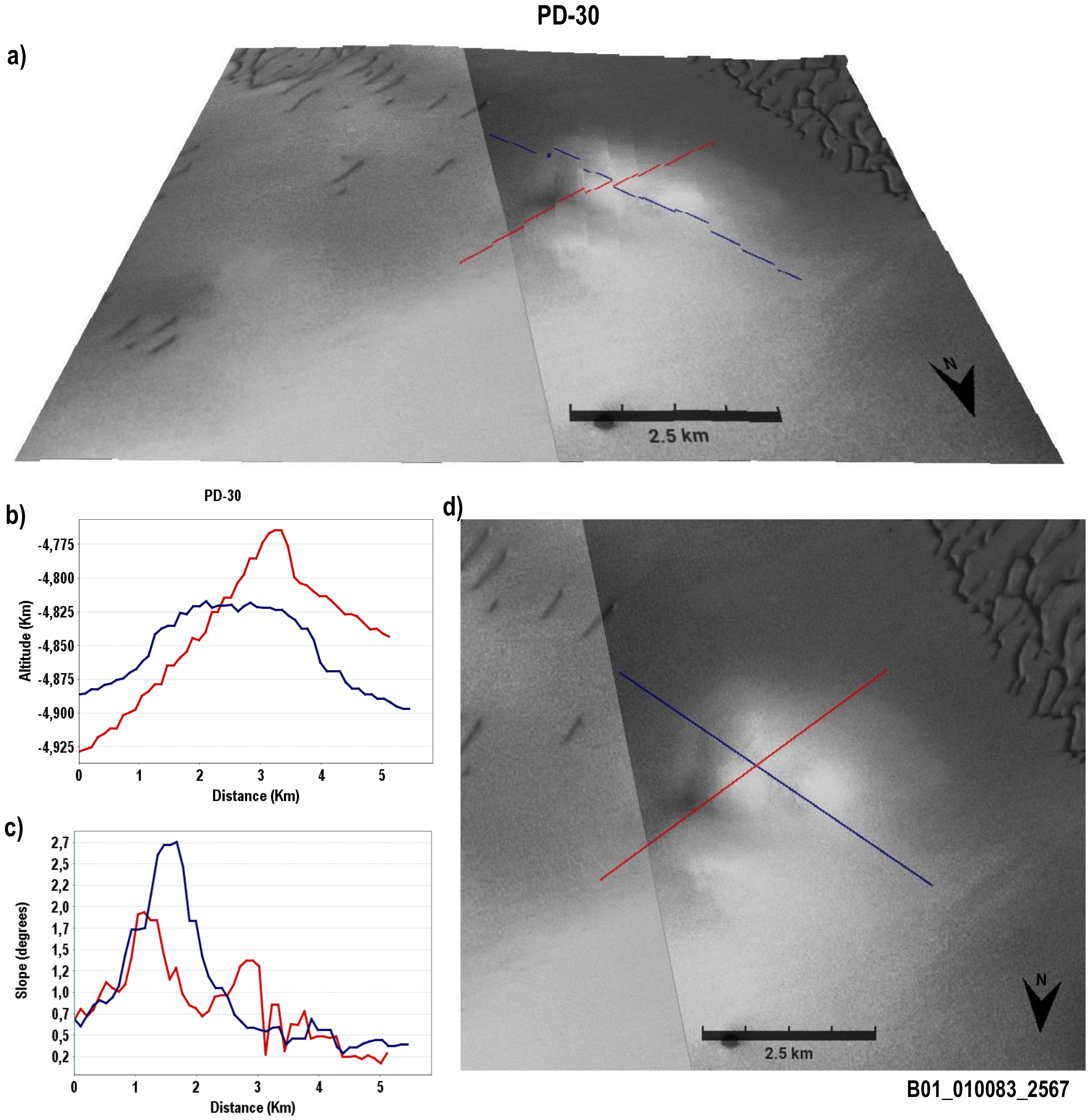
PD-30
a)
N
2.5 km
b)
PD-30
Altitude (Km)
-4,775
-4,800
-4,825
-4,850
-4,875
-4,900
-4,925
0
1
2
3
4
5
Distance (Km)
c)
Slope (degrees)
2,7
2,5
2,2
2,0
1,7
1,5
1,2
1,0
0,7
0,5
0,2
0
1
2
3
4
5
Distance (Km)
d)
N
2.5 km
B01_010083_2567

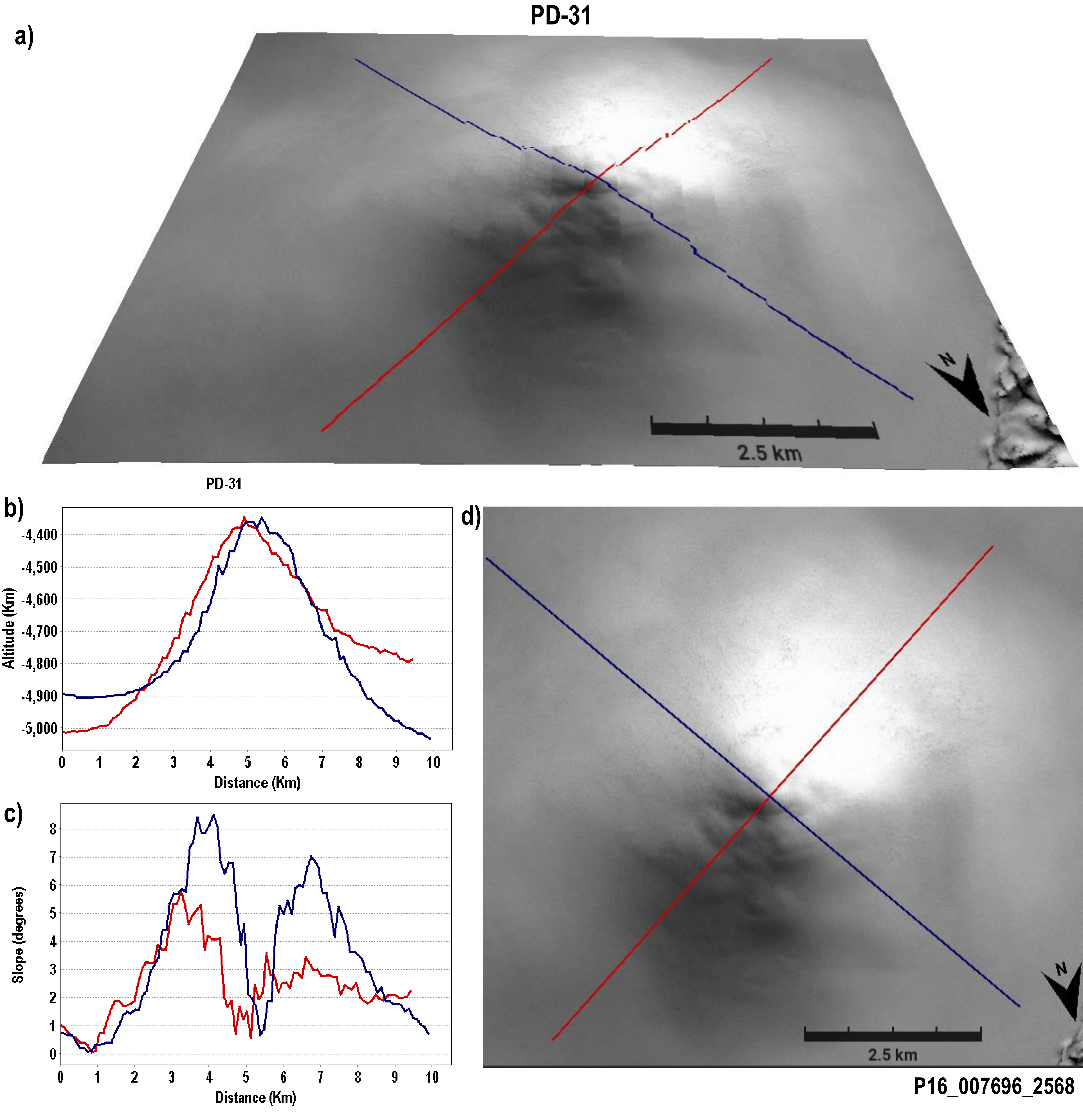
PD-31
a)
2.5 km
N
PD-31
b)
Altitude (Km)
-4,400
-4,500
-4,600
-4,700
-4,800
-4,900
-5,000
0 1 2 3 4 5 6 7 8 9 10
Distance (Km)
c)
Slope (degrees)
0 1 2 3 4 5 6 7 8
0 1 2 3 4 5 6 7 8 9 10
Distance (Km)
d)
2.5 km
N
P16_007696_2568

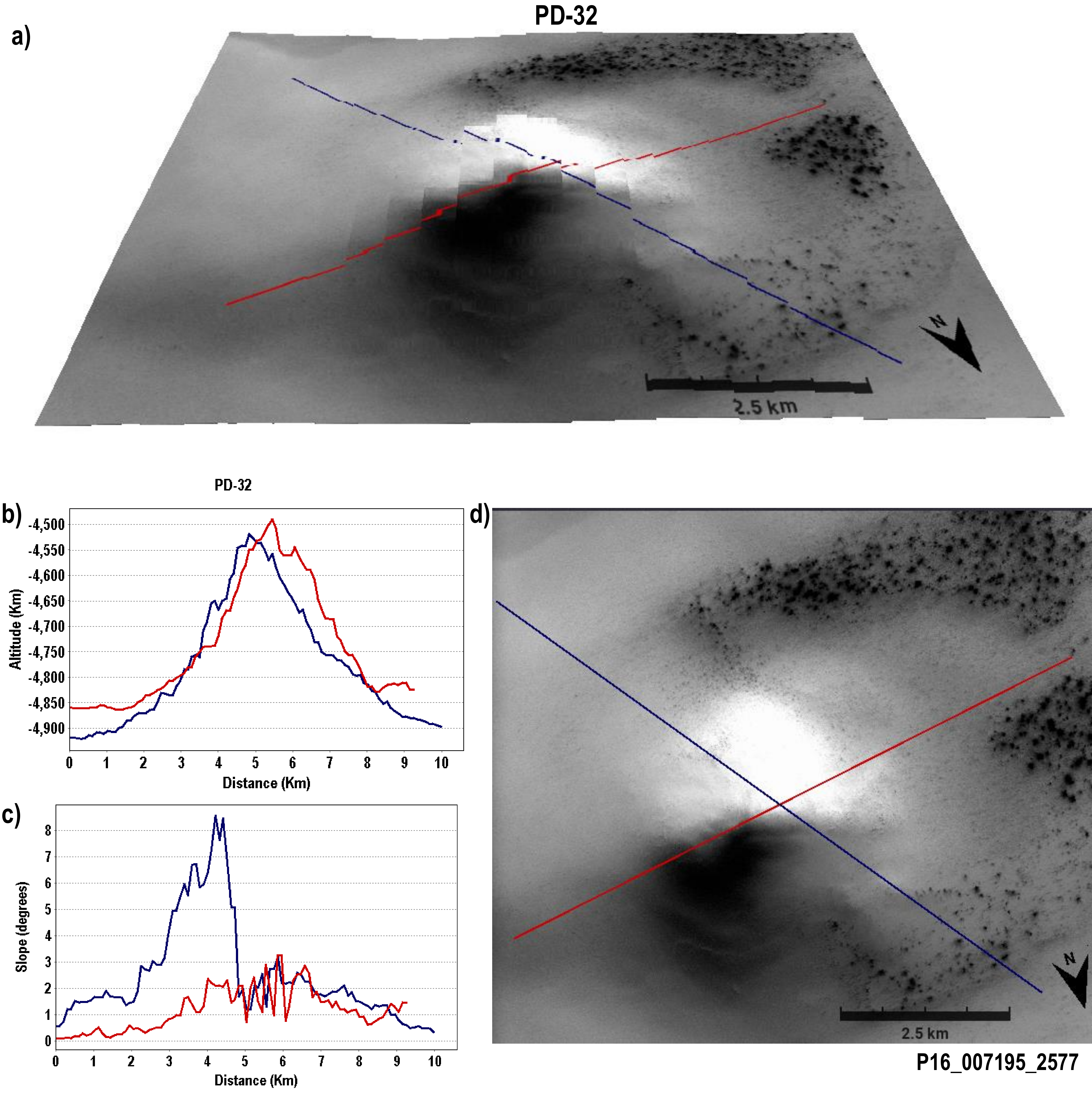

PD-32
a)
2.5 km
N
PD-32
b)
-4,500
-4,550
-4,600
-4,650
-4,700
-4,750
-4,800
-4,850
-4,900
Altitude (Km)
0 1 2 3 4 5 6 7 8 9 10
Distance (Km)
c)
0 1 2 3 4 5 6 7 8
Slope (degrees)
0 1 2 3 4 5 6 7 8 9 10
Distance (Km)
d)
N
2.5 km
P16_007195_2577

PD-33

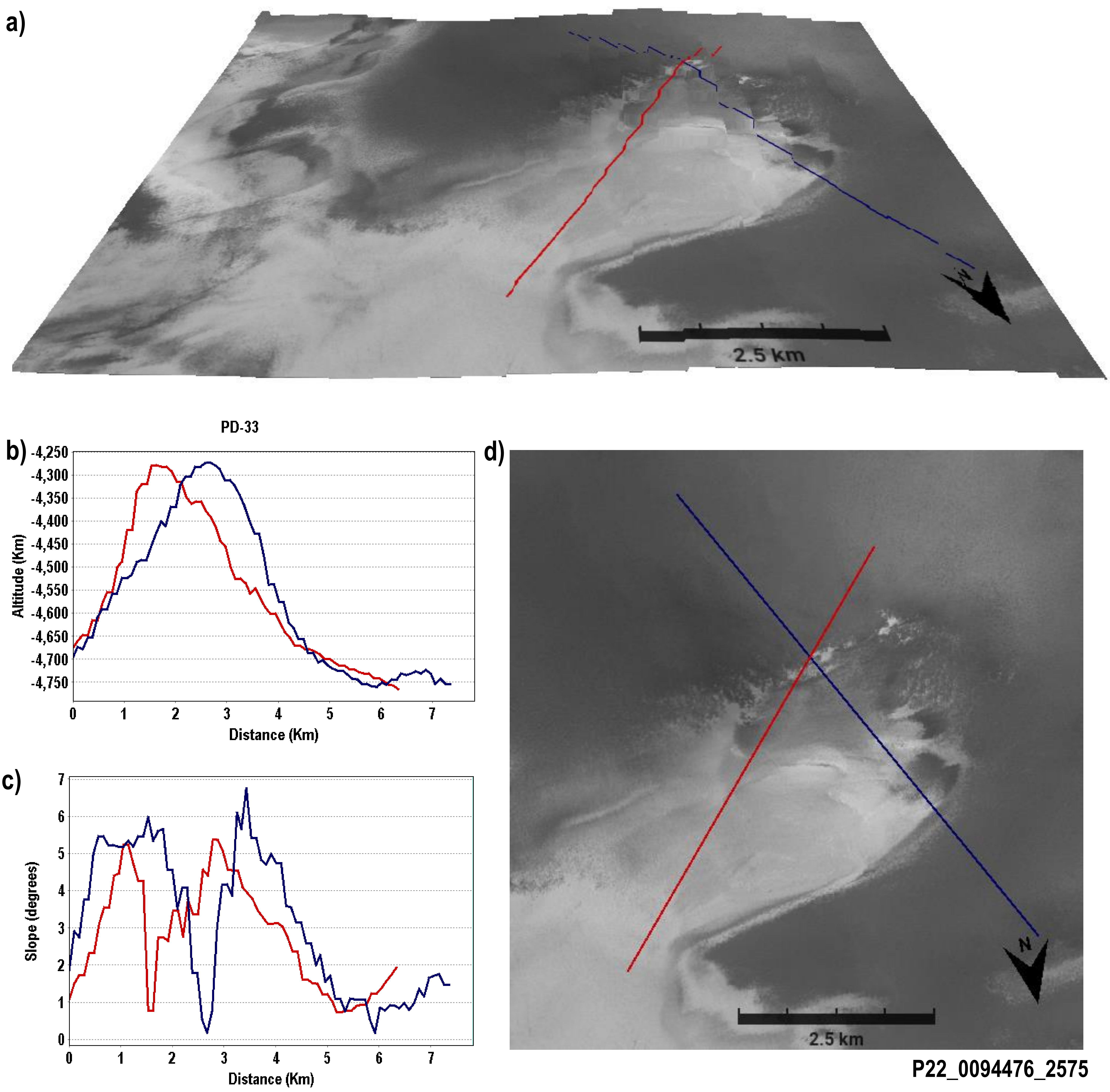
a)
2.5 km
N
b)
PD-33
Altitude (Km)
-4,250
-4,300
-4,350
-4,400
-4,450
-4,500
-4,550
-4,600
-4,650
-4,700
-4,750
Distance (Km)
0
1
2
3
4
5
6
7
c)
Slope (degrees)
0
1
2
3
4
5
6
7
Distance (Km)
d)
N
2.5 km
P22_0094476_2575

PD-34

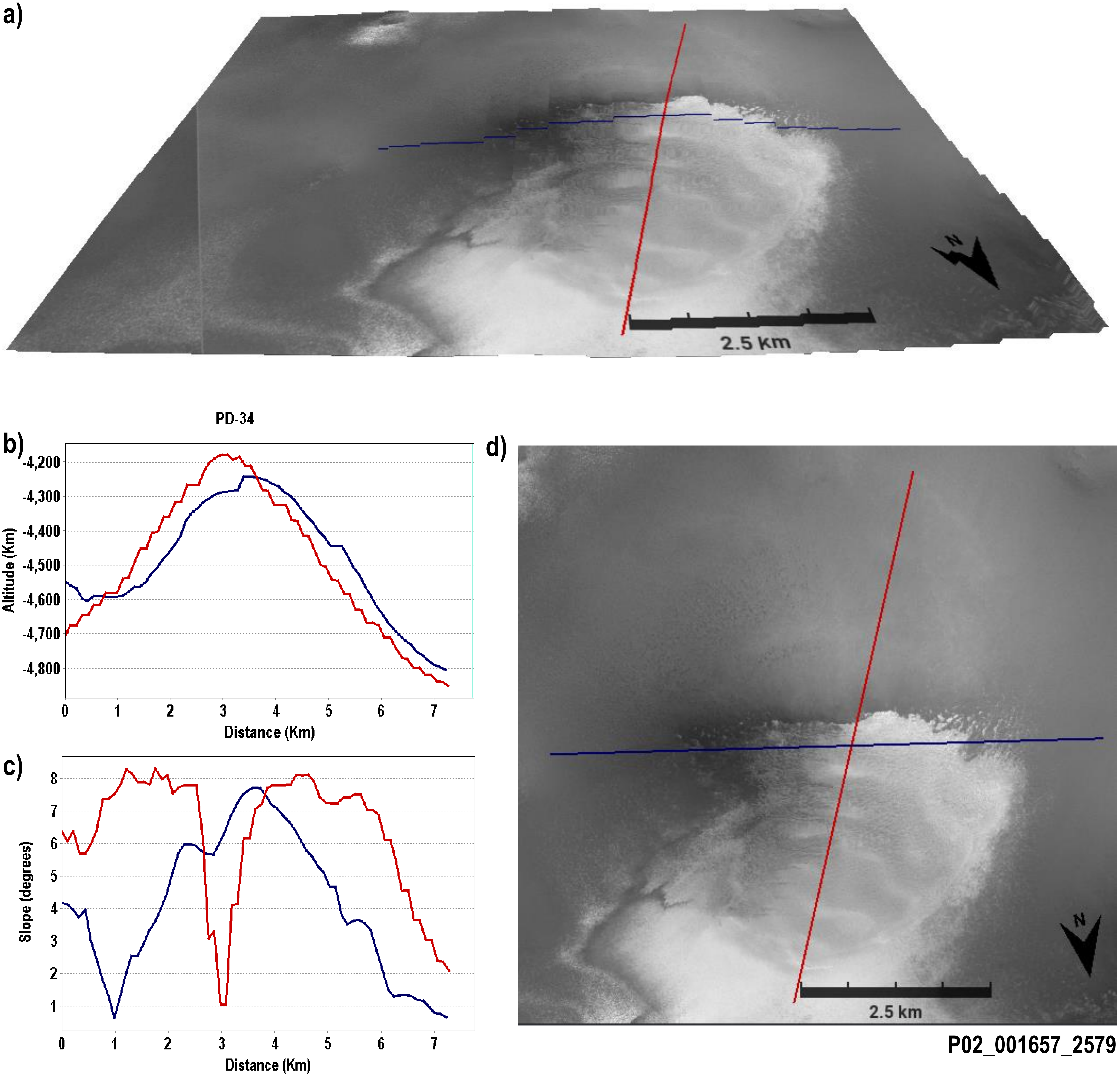
a)
N
2.5 km
PD-34
b)
Altitude (Km)
-4,200
-4,300
-4,400
-4,500
-4,600
-4,700
-4,800
0
1
2
3
4
5
6
7
Distance (Km)
c)
Slope (degrees)
1
2
3
4
5
6
7
8
0
1
2
3
4
5
6
7
Distance (Km)
d)
N
2.5 km
P02_001657_2579

PD-35

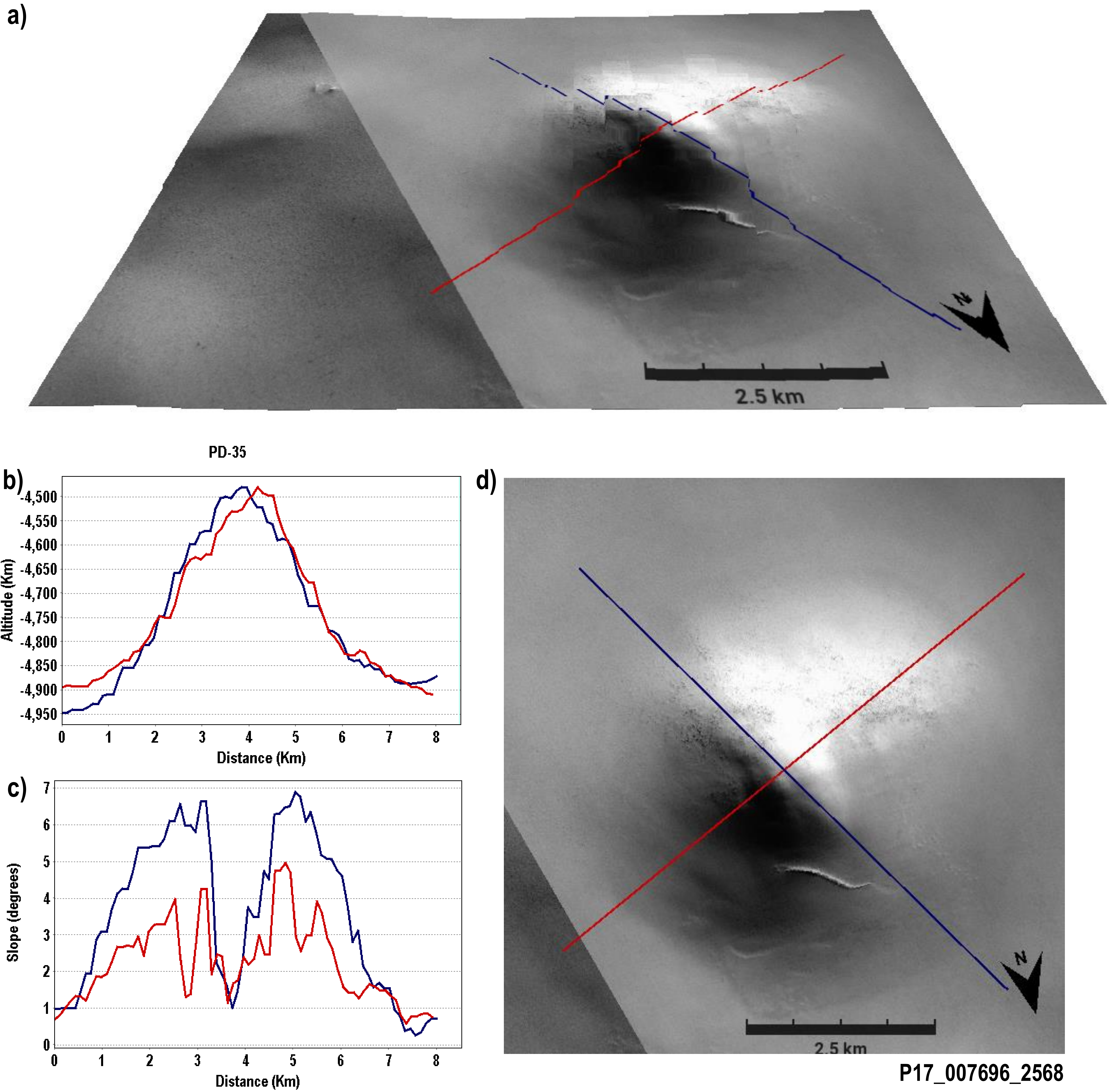

P17_007696_2568

PD-36

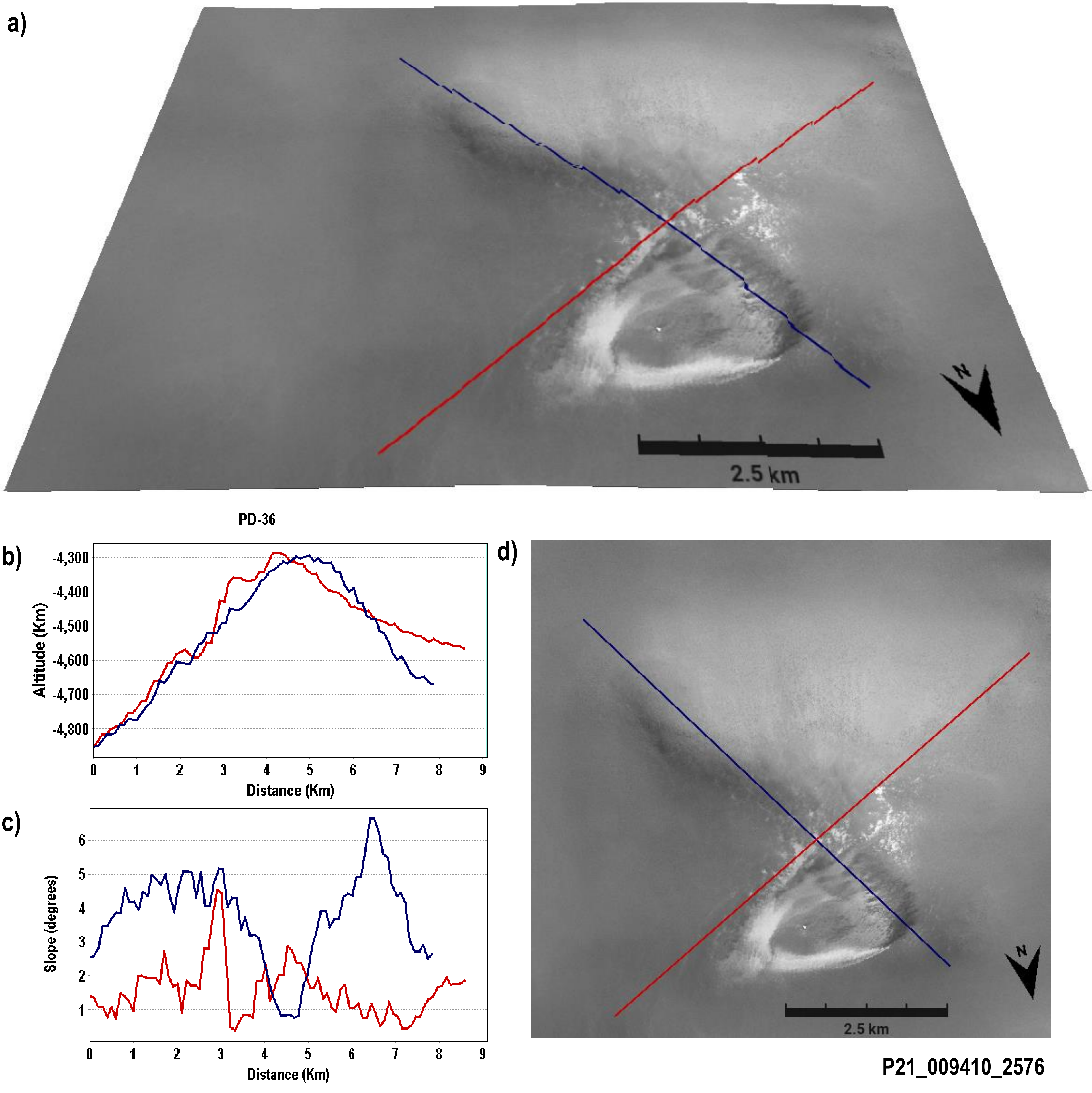

a)
N
2.5 km
PD-36
b)
Altitude (Km)
-4,300
-4,400
-4,500
-4,600
-4,700
-4,800
0 1 2 3 4 5 6 7 8 9
Distance (Km)
c)
Slope (degrees)
6
5
4
3
2
1
0 1 2 3 4 5 6 7 8 9
Distance (Km)
d)
N
2.5 km
P21_009410_2576

PD-37

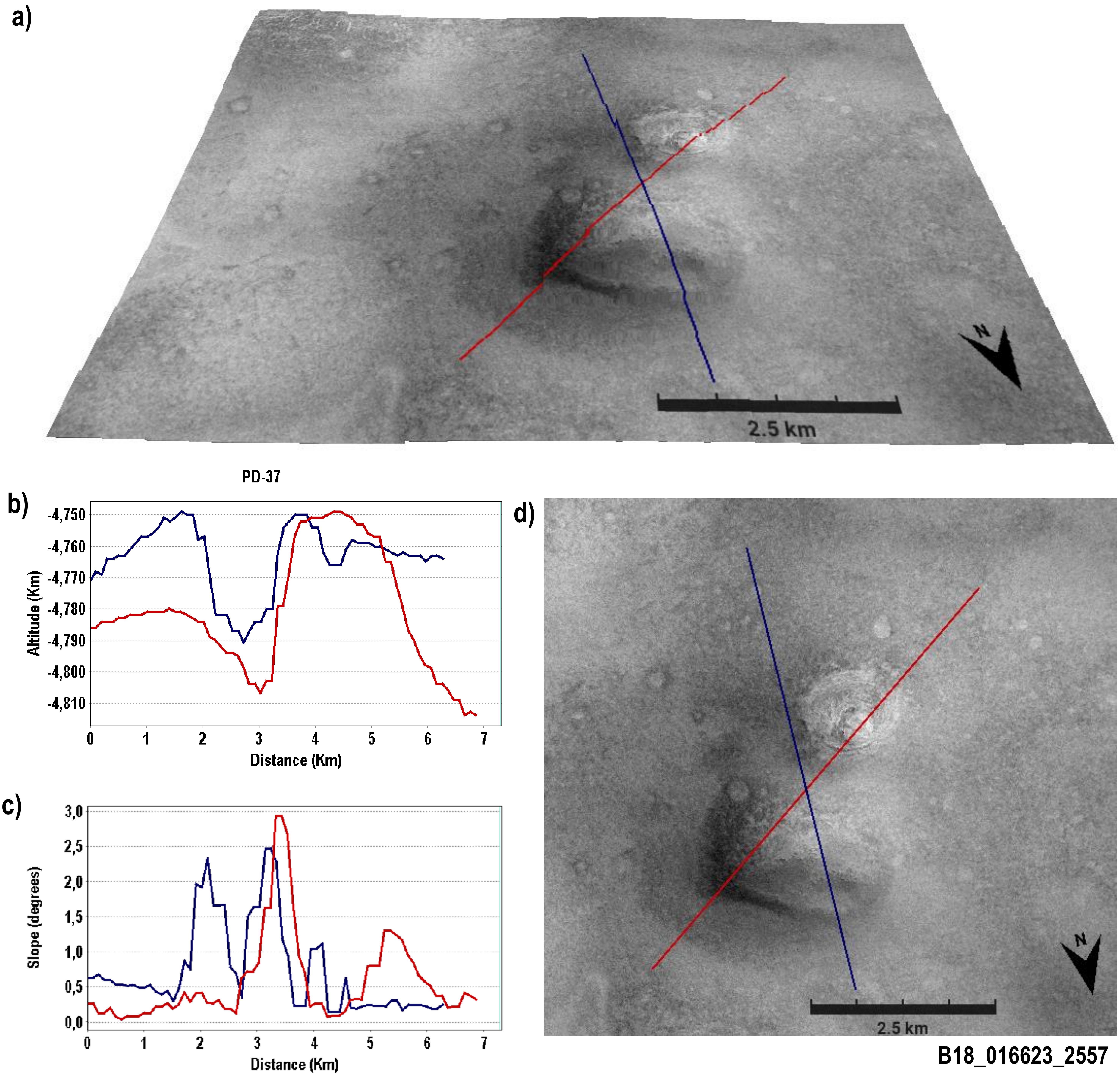

B18_016623_2557

PD-38

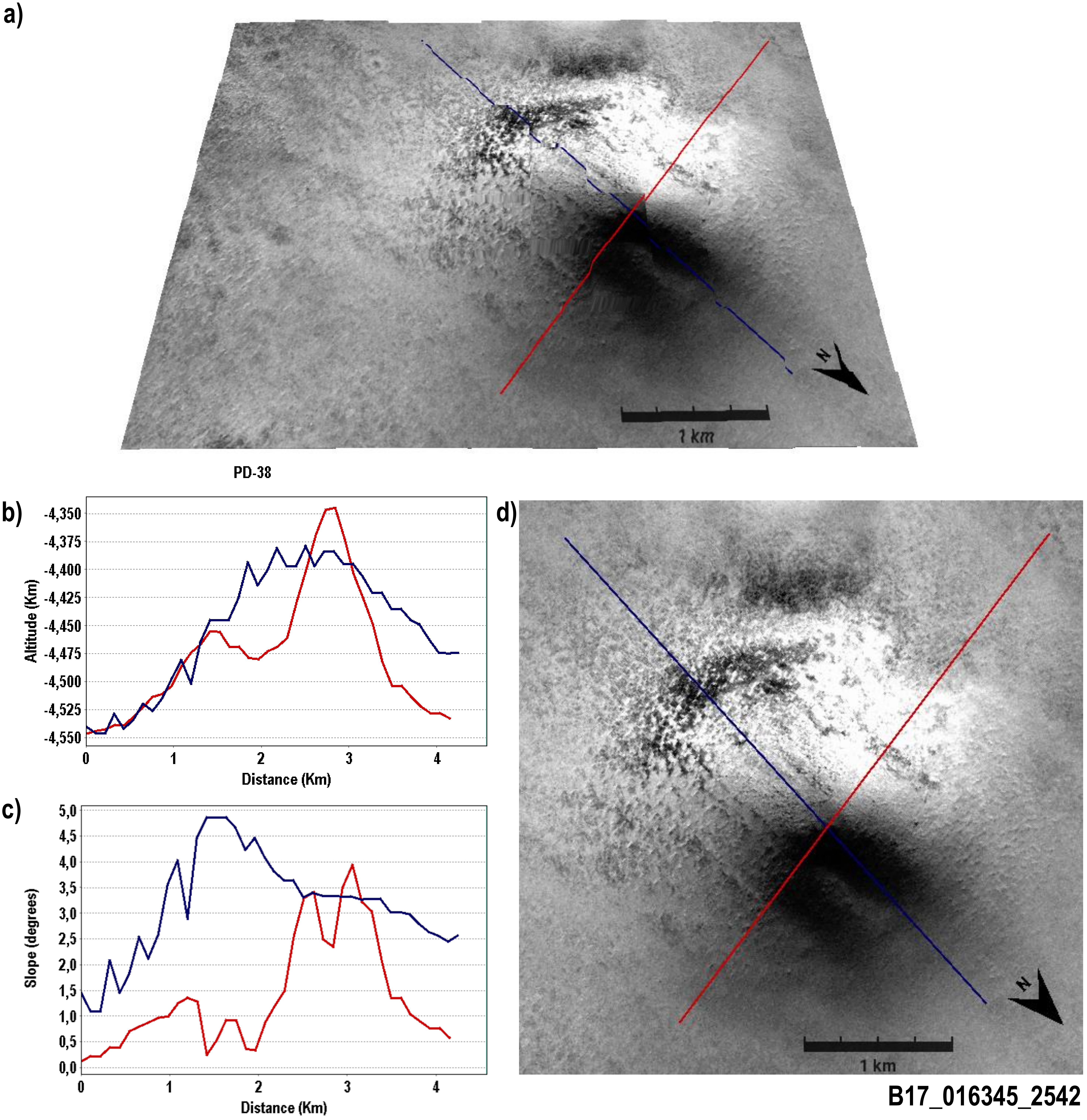

a)
N
1 km
PD-38
b)
Altitude (Km)
-4,350
-4,375
-4,400
-4,425
-4,450
-4,475
-4,500
-4,525
-4,550
0
1
2
3
4
Distance (Km)
c)
Slope (degrees)
5,0
4,5
4,0
3,5
3,0
2,5
2,0
1,5
1,0
0,5
0,0
0
1
2
3
4
Distance (Km)
d)
N
1 km
B17_016345_2542

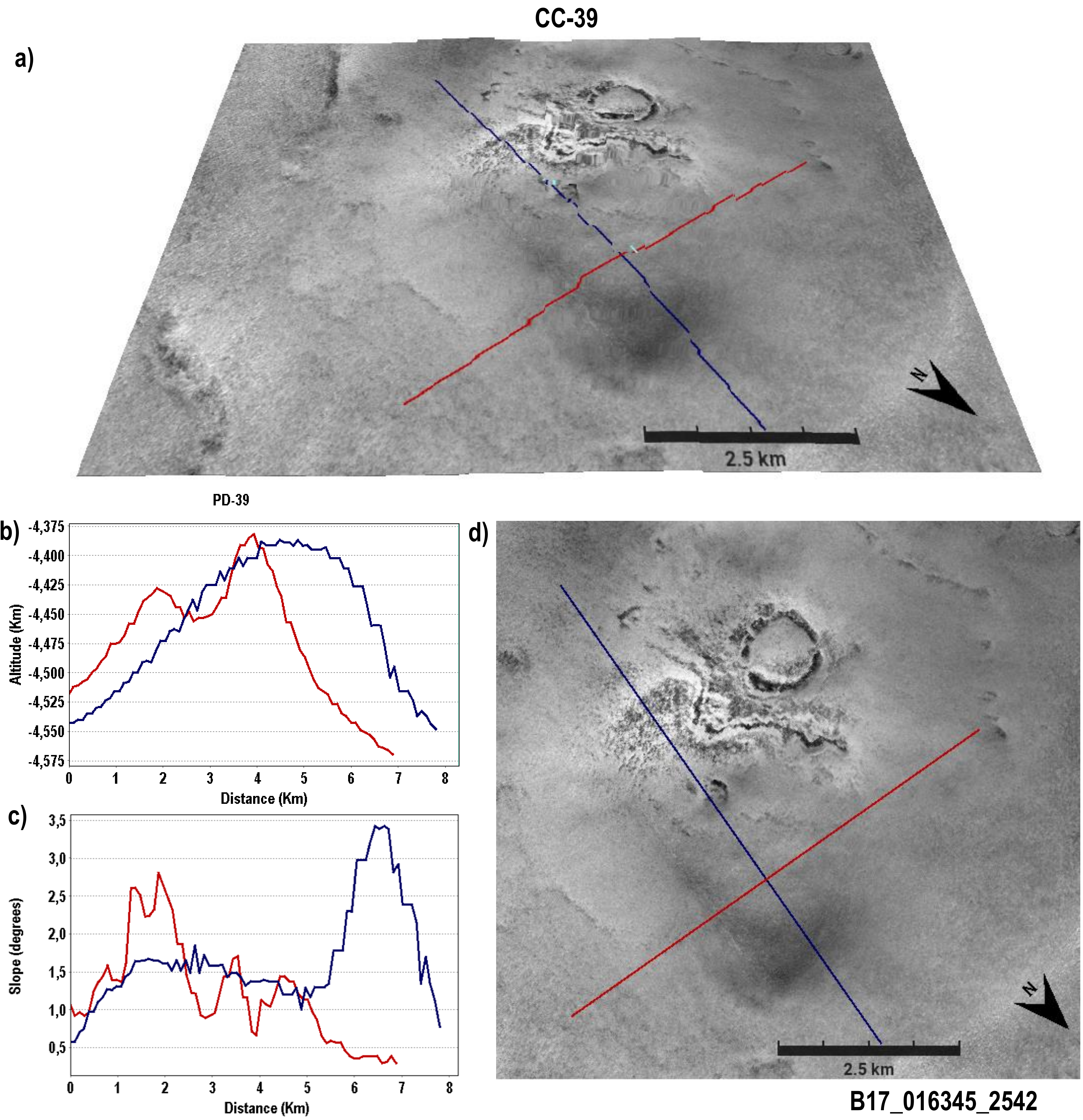

CC-39
a)
2.5 km
N
PD-39
b)
Altitude (Km)
-4,375
-4,400
-4,425
-4,450
-4,475
-4,500
-4,525
-4,550
-4,575
0
1
2
3
4
5
6
7
8
Distance (Km)
c)
Slope (degrees)
3,5
3,0
2,5
2,0
1,5
1,0
0,5
0
1
2
3
4
5
6
7
8
Distance (Km)
d)
2.5 km
N
B17_016345_2542

PD-40

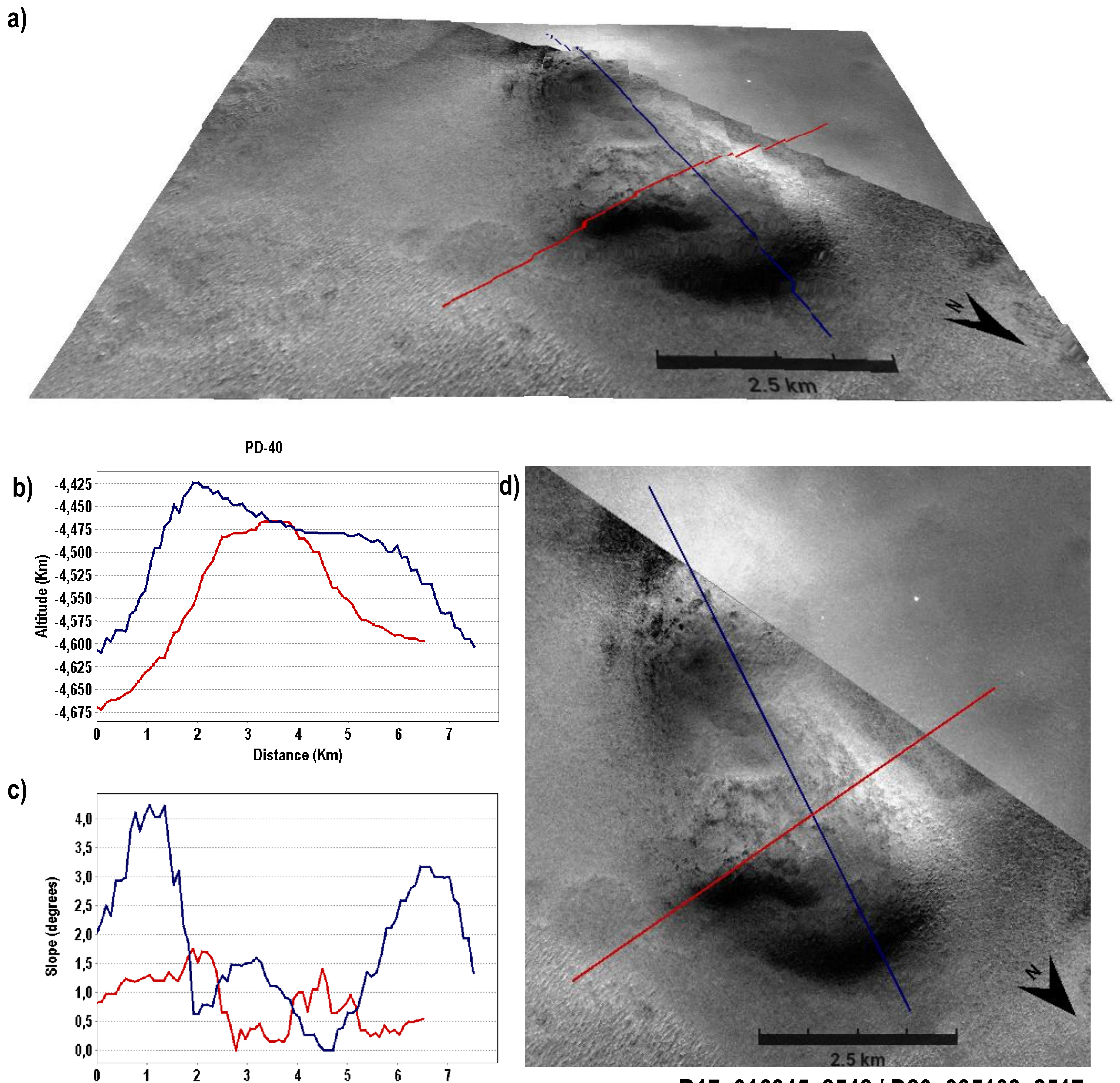

B17_016345_2542 / D20_035109_2517

SD-01

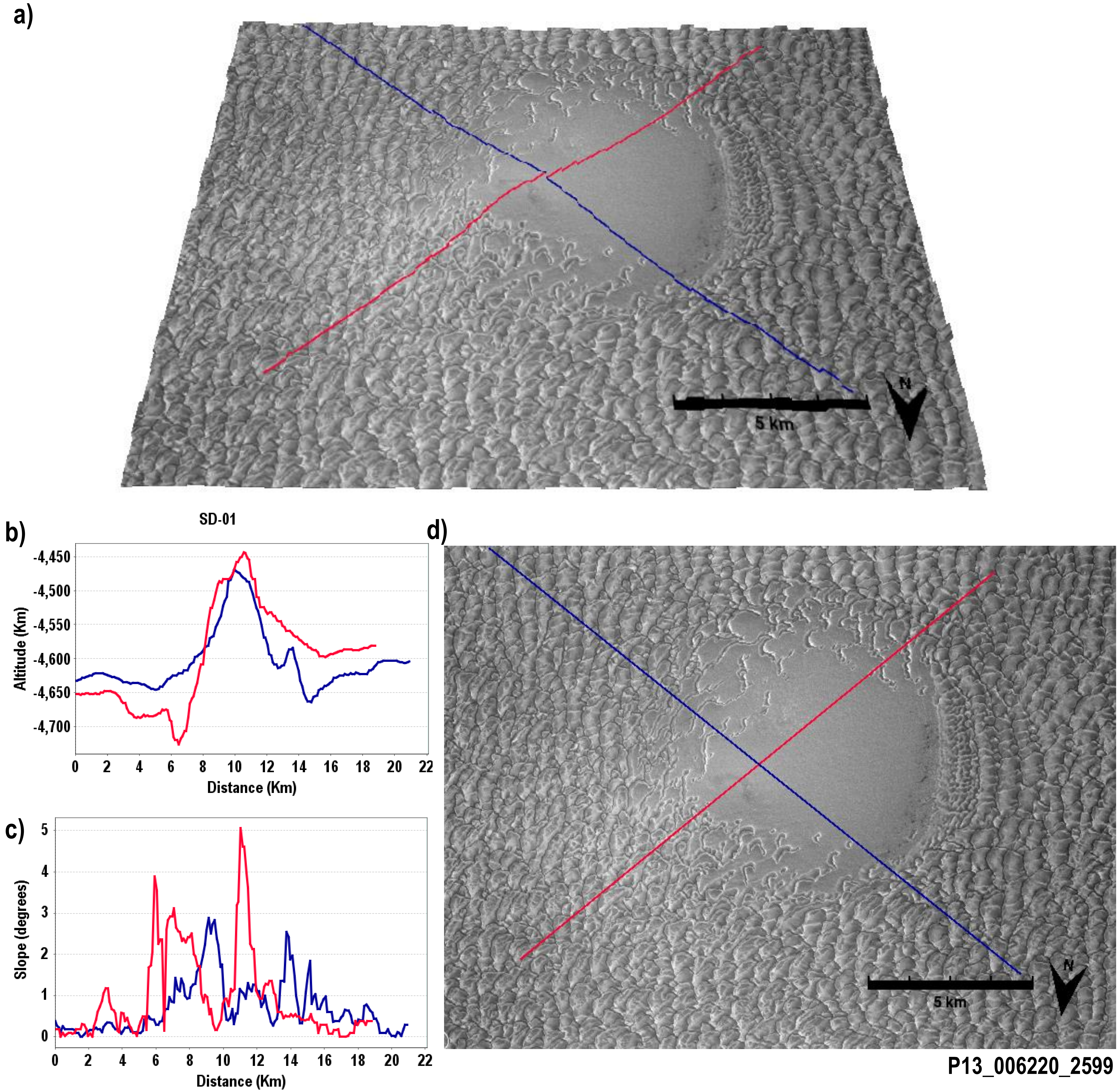

P13_006220_2599

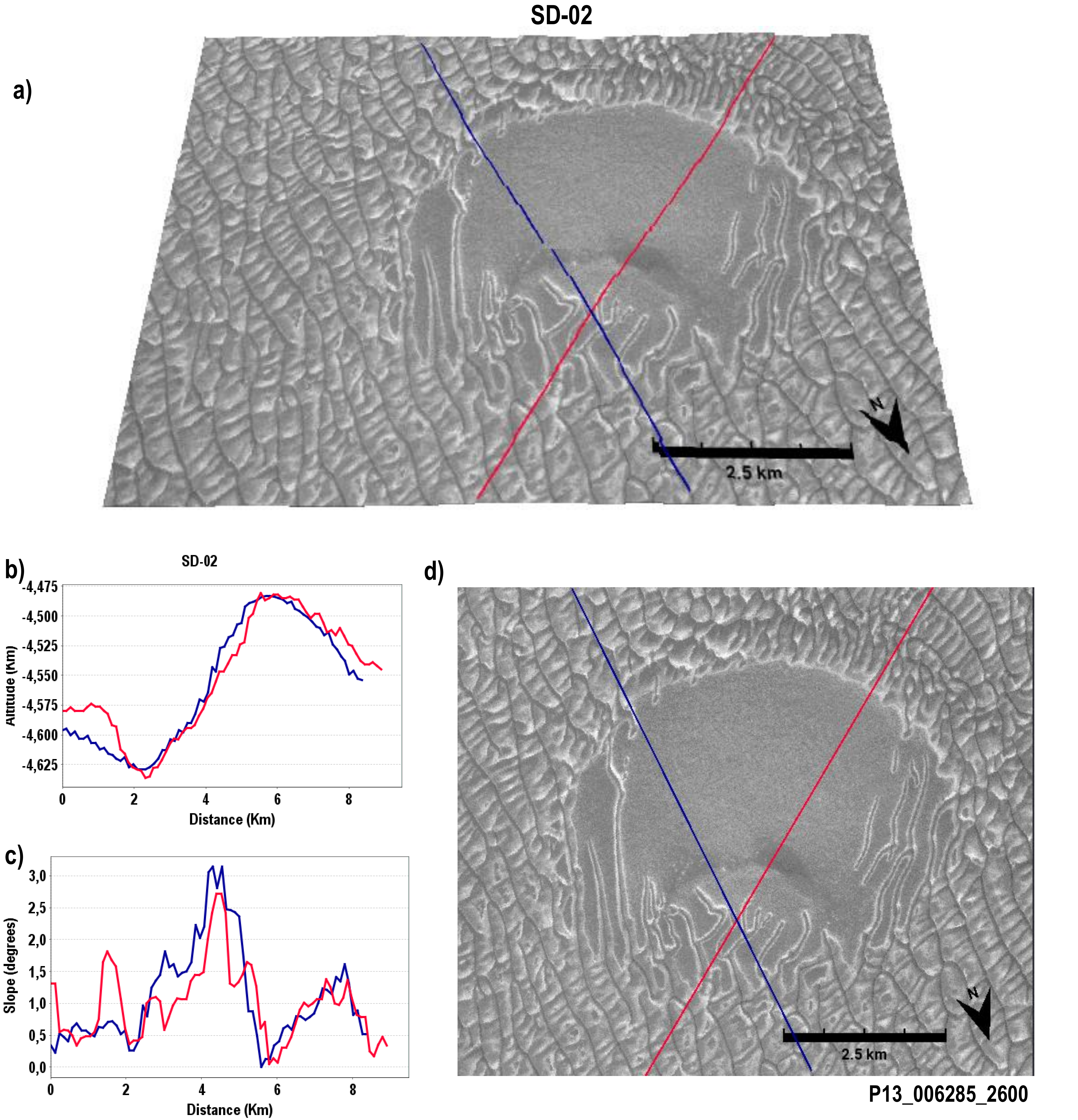
SD-02
a)
2.5 km
N
b)
SD-02
-4,475
-4,500
-4,525
-4,550
-4,575
-4,600
-4,625
Altitude (Km)
0
2
4
6
8
Distance (Km)
c)
3,0
2,5
2,0
1,5
1,0
0,5
0,0
Slope (degrees)
Distance (Km)
d)
2.5 km
N
P13_006285_2600

SD-03

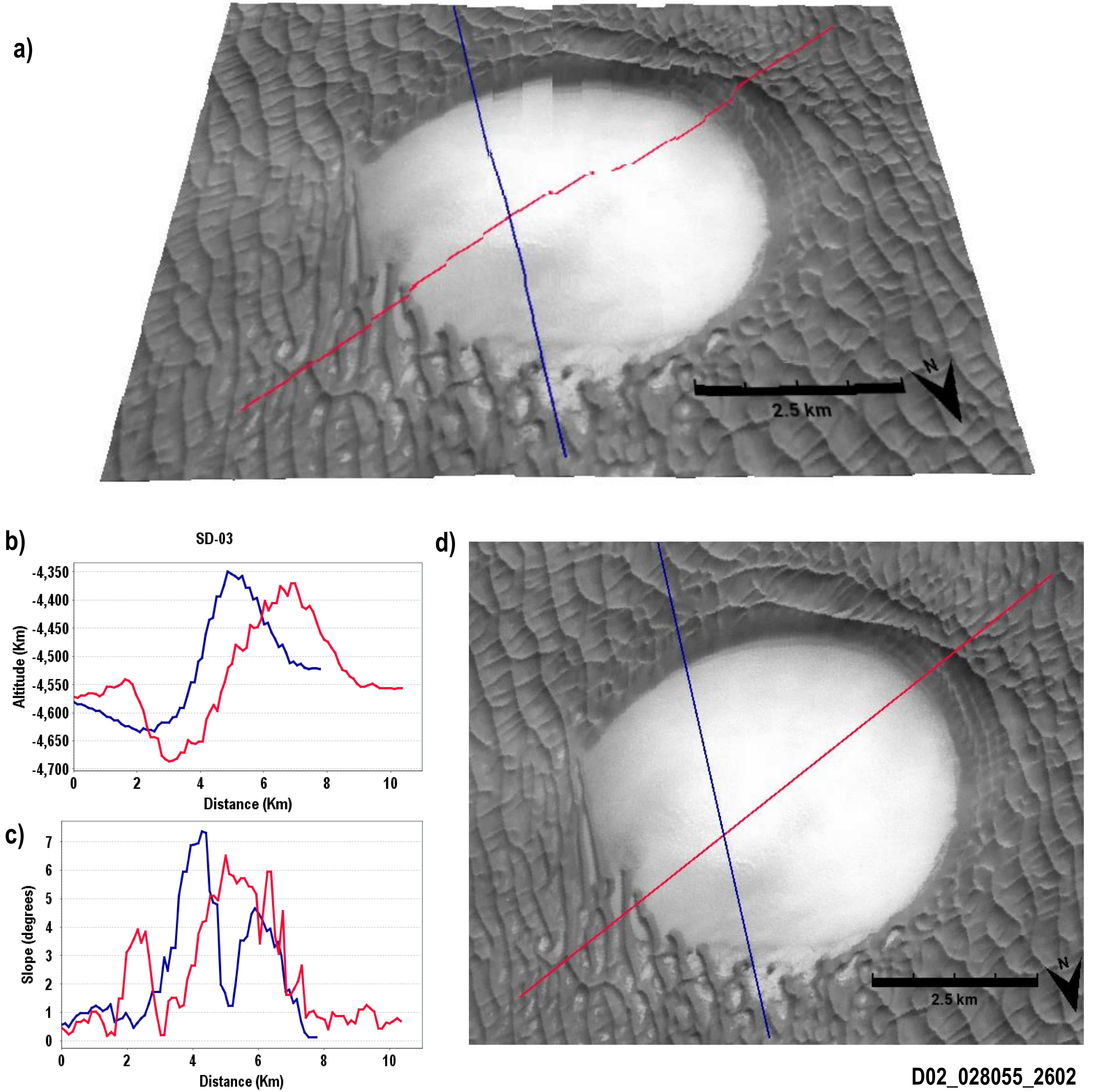

D02_028055_2602

SD-04

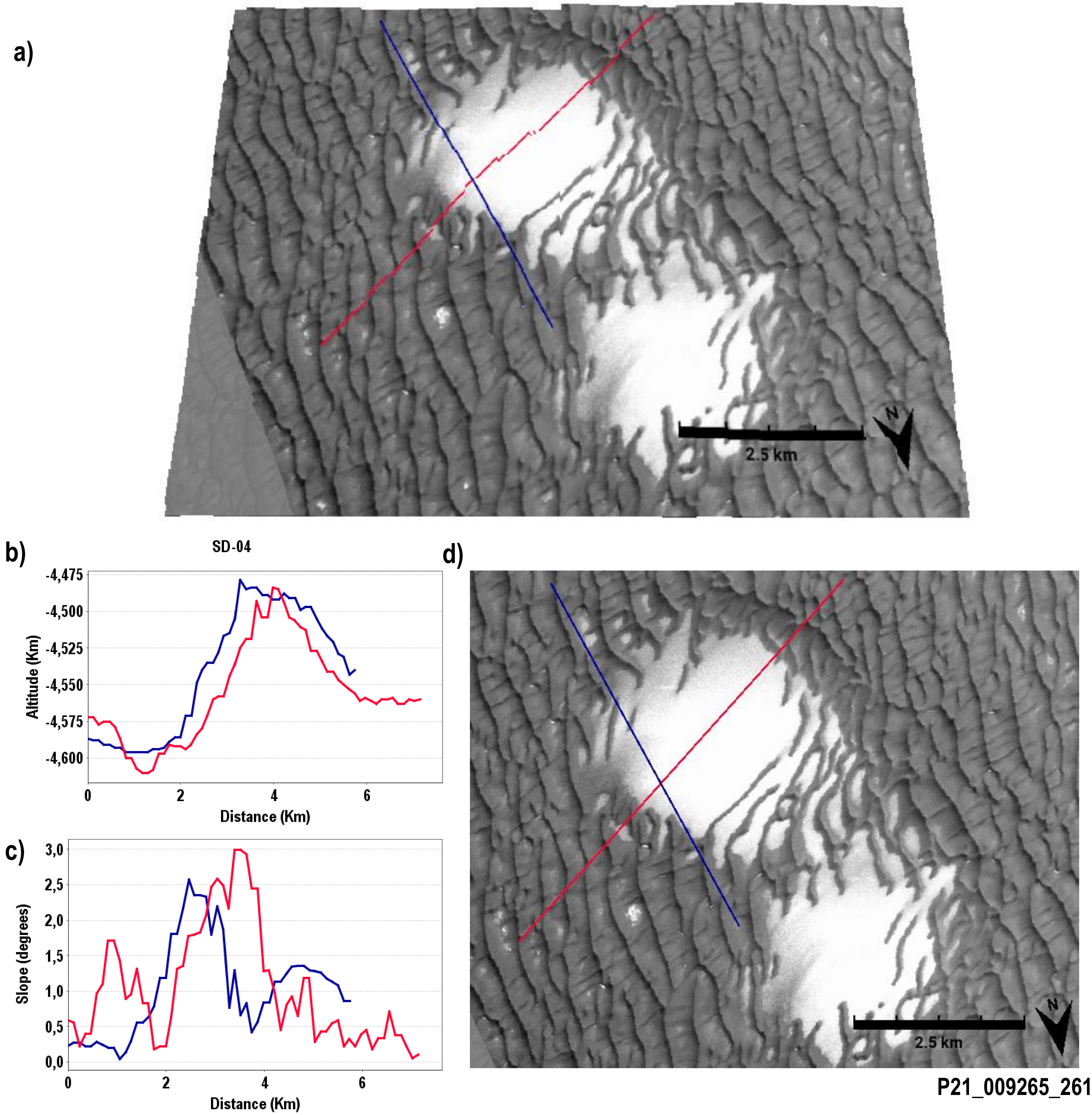

P21_009265_2610

SD-05

a)

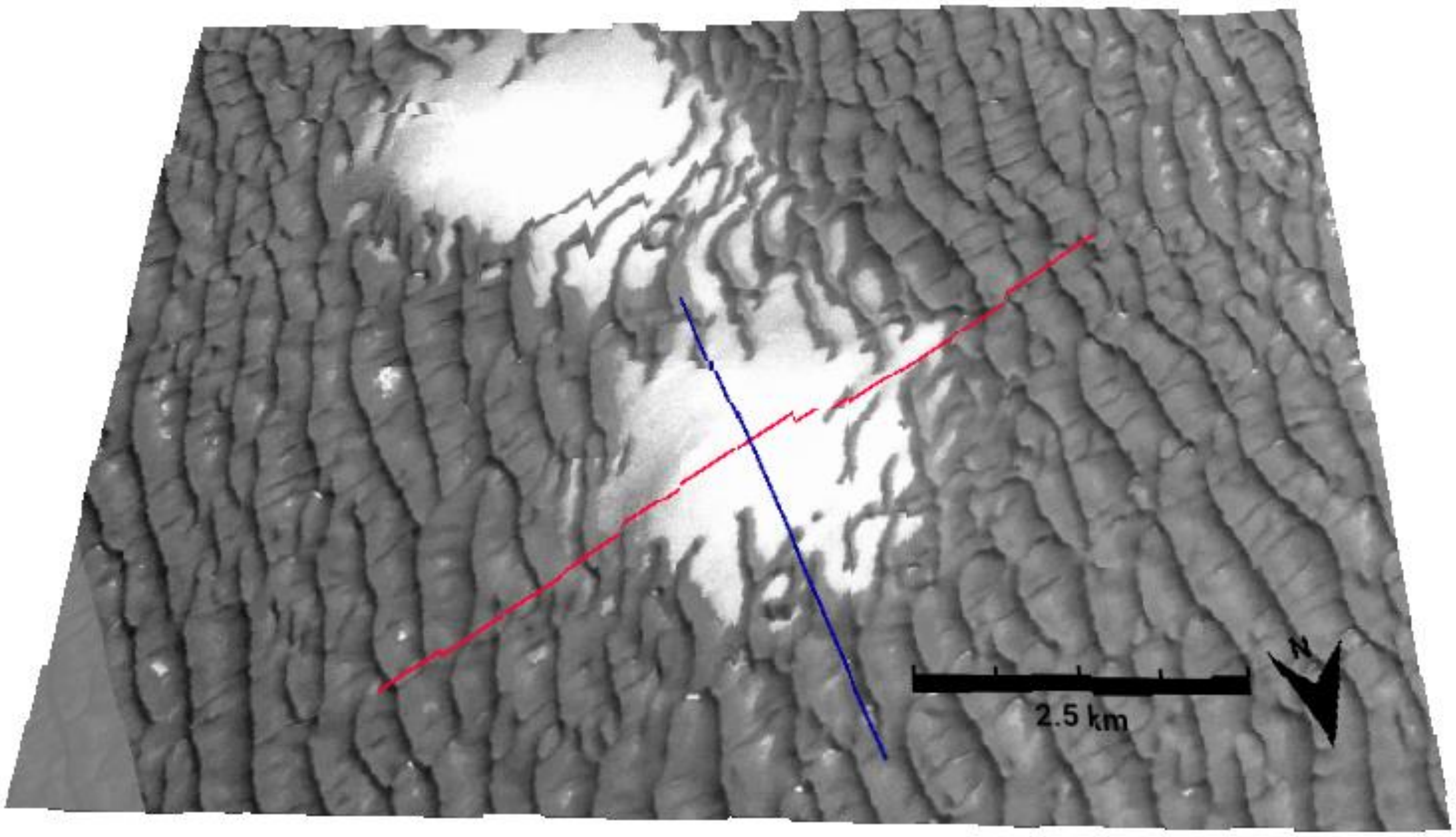

b)

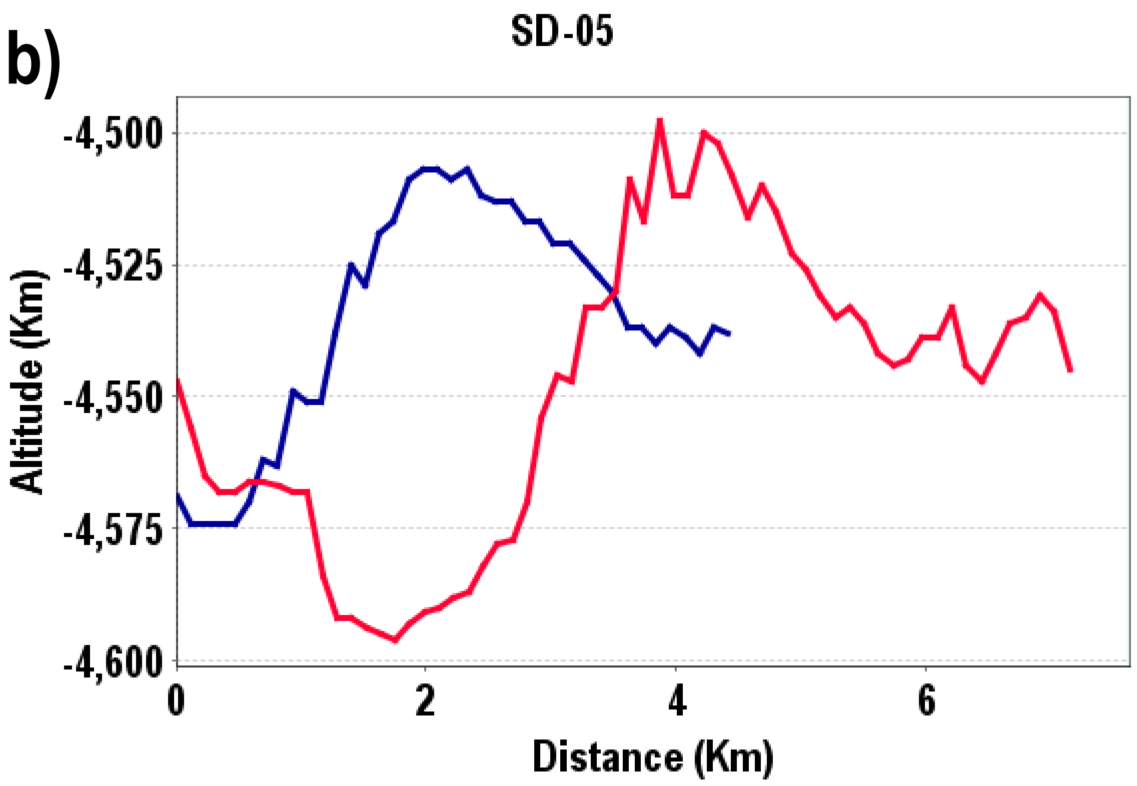

c)

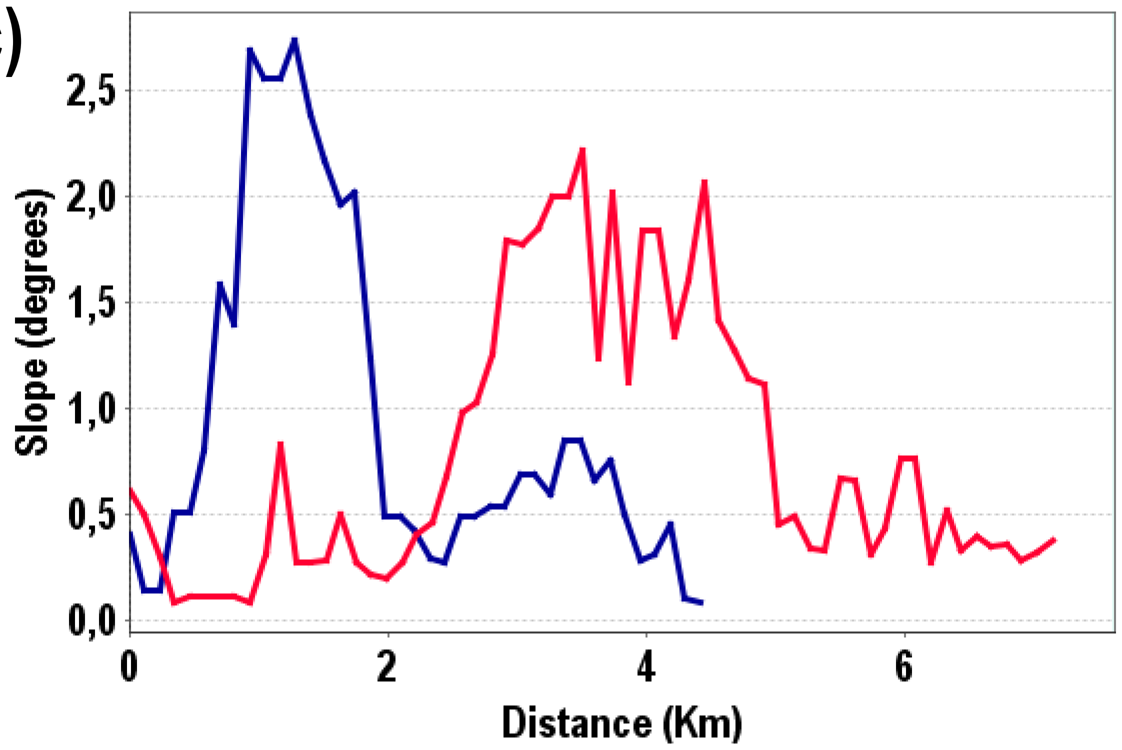

d)

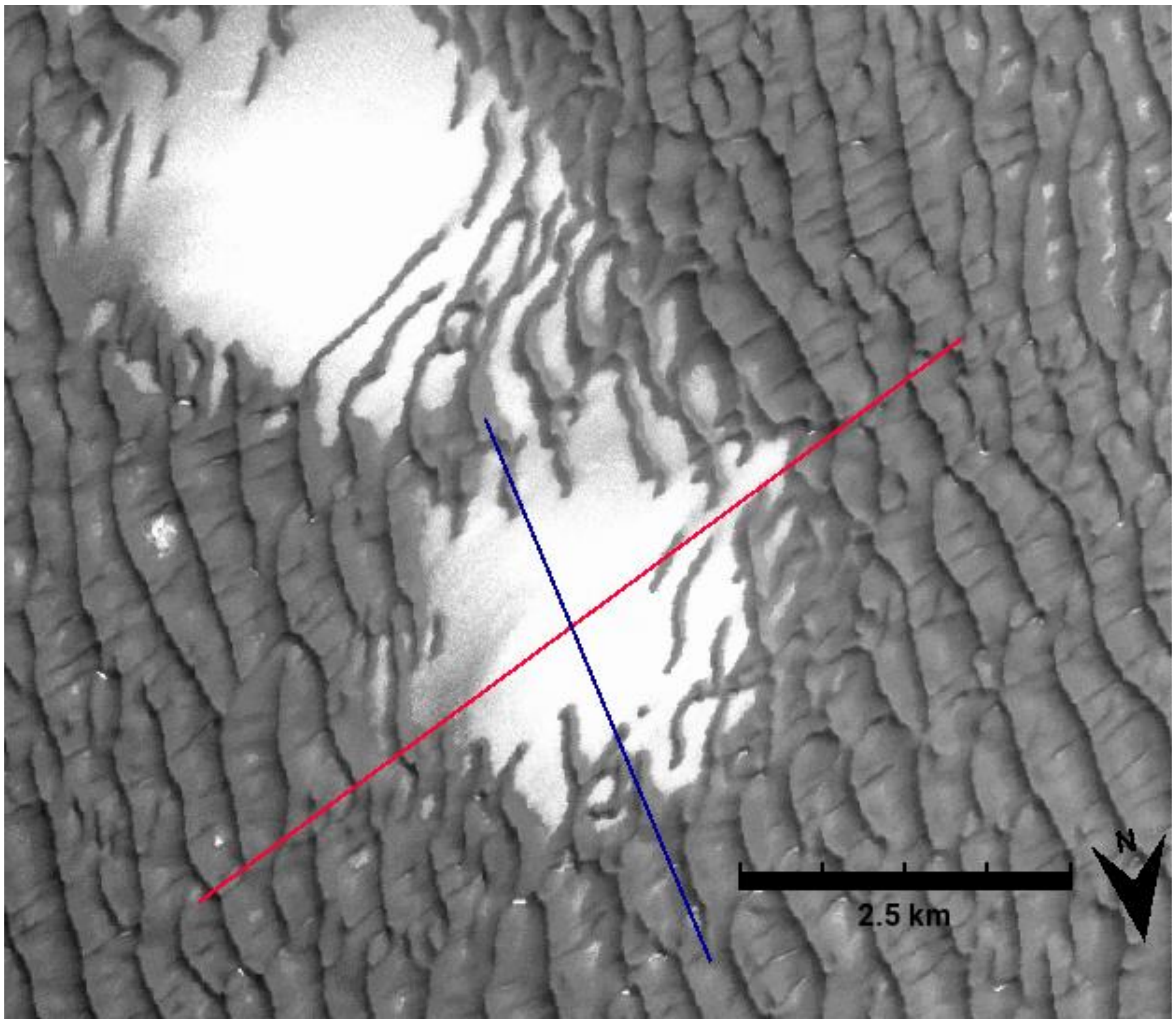

P21_009265_2610

SD-06

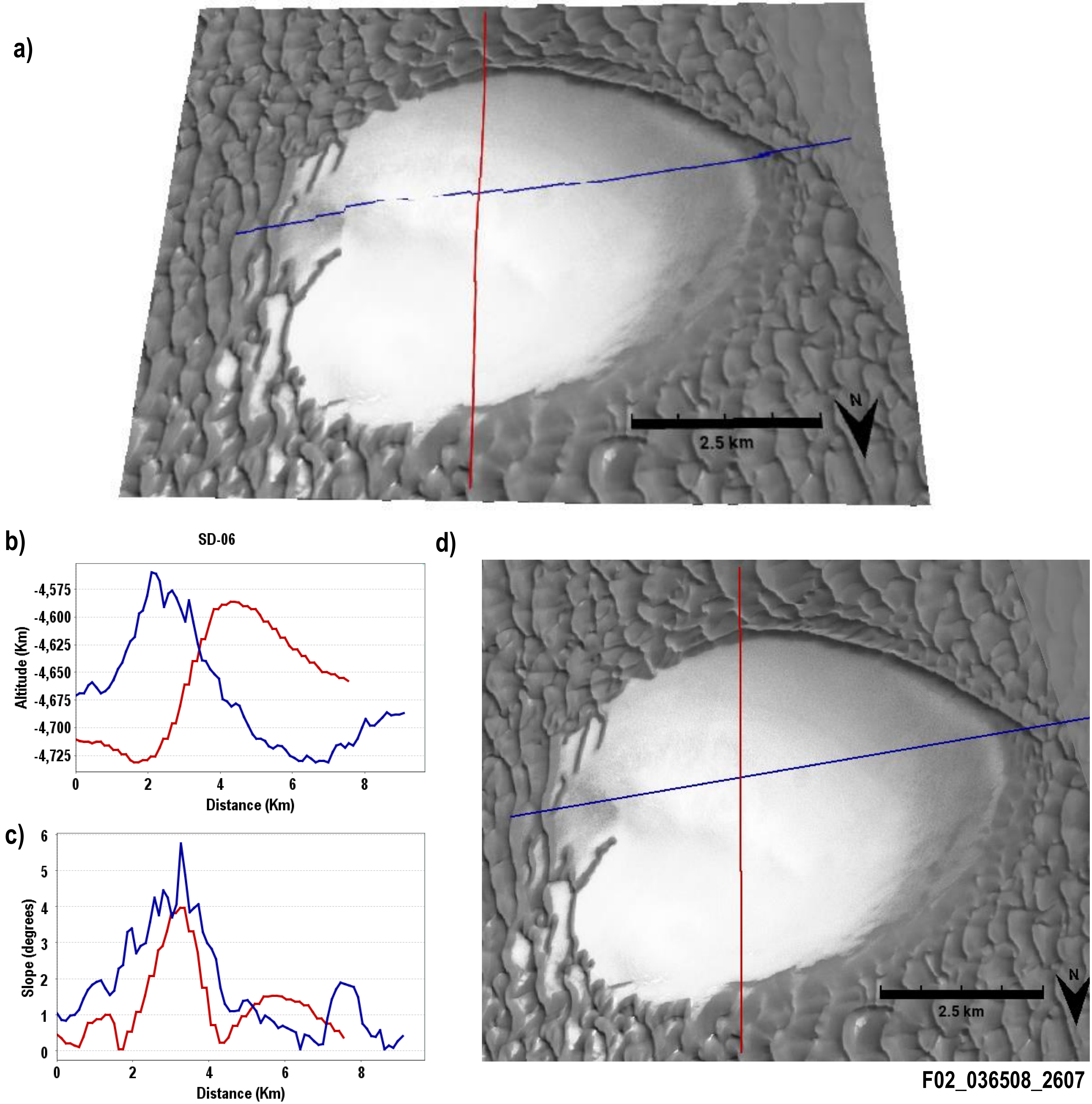
a)
2.5 km
N
b)
SD-06
Altitude (Km)
-4,575
-4,600
-4,625
-4,650
-4,675
-4,700
-4,725
0
2
4
6
8
Distance (Km)
c)
Slope (degrees)
6
5
4
3
2
1
0
0
2
4
6
8
Distance (Km)
d)
2.5 km
N
F02_036508_2607

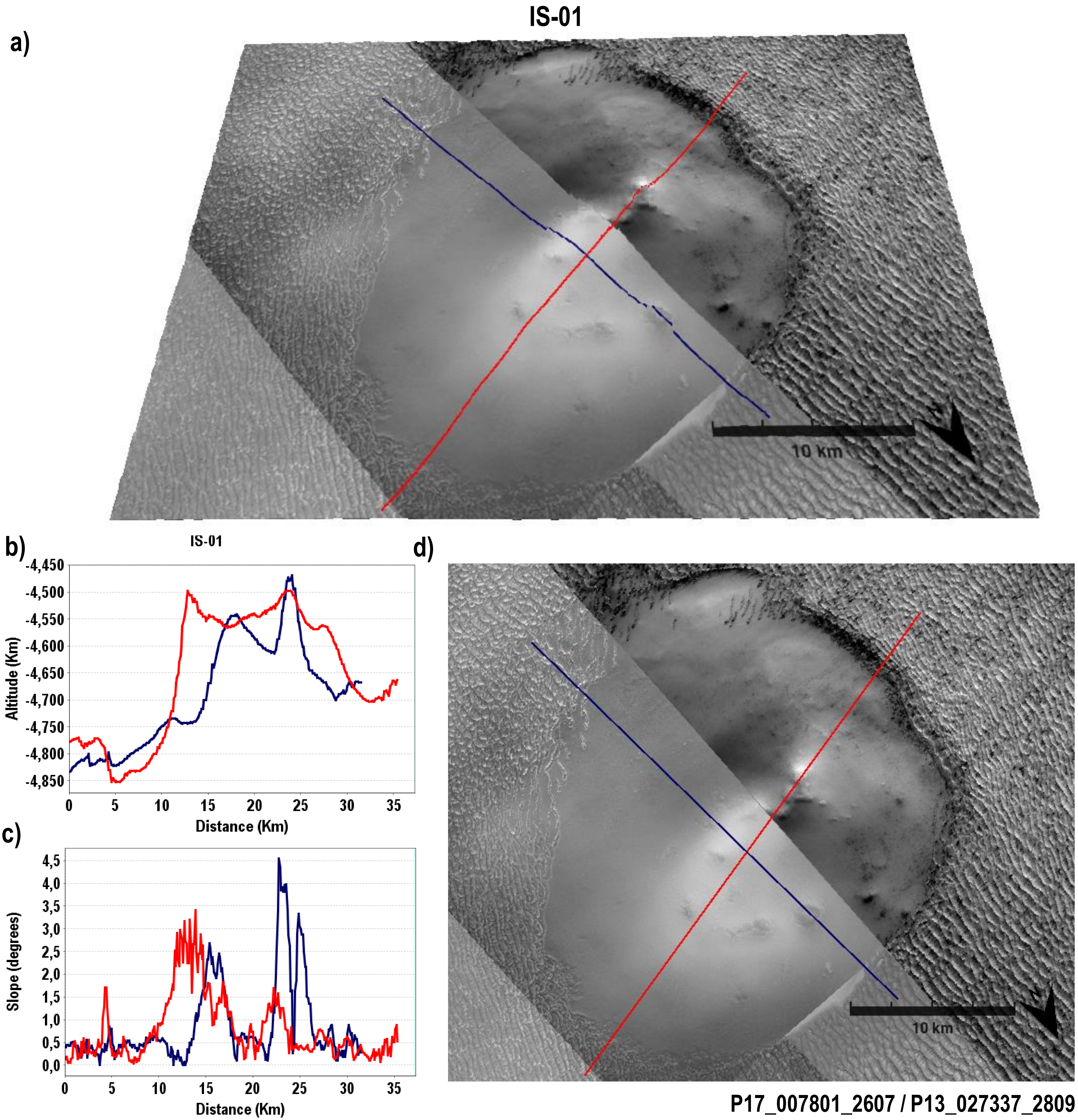
IS-01
a)
10 km
b)
IS-01
Altitude (Km)
-4,450
-4,500
-4,550
-4,600
-4,650
-4,700
-4,750
-4,800
-4,850
0 5 10 15 20 25 30 35
Distance (Km)
c)
Slope (degrees)
4,5
4,0
3,5
3,0
2,5
2,0
1,5
1,0
0,5
0,0
0 5 10 15 20 25 30 35
Distance (Km)
d)
10 km
P17_007801_2607 / P13_027337_2809

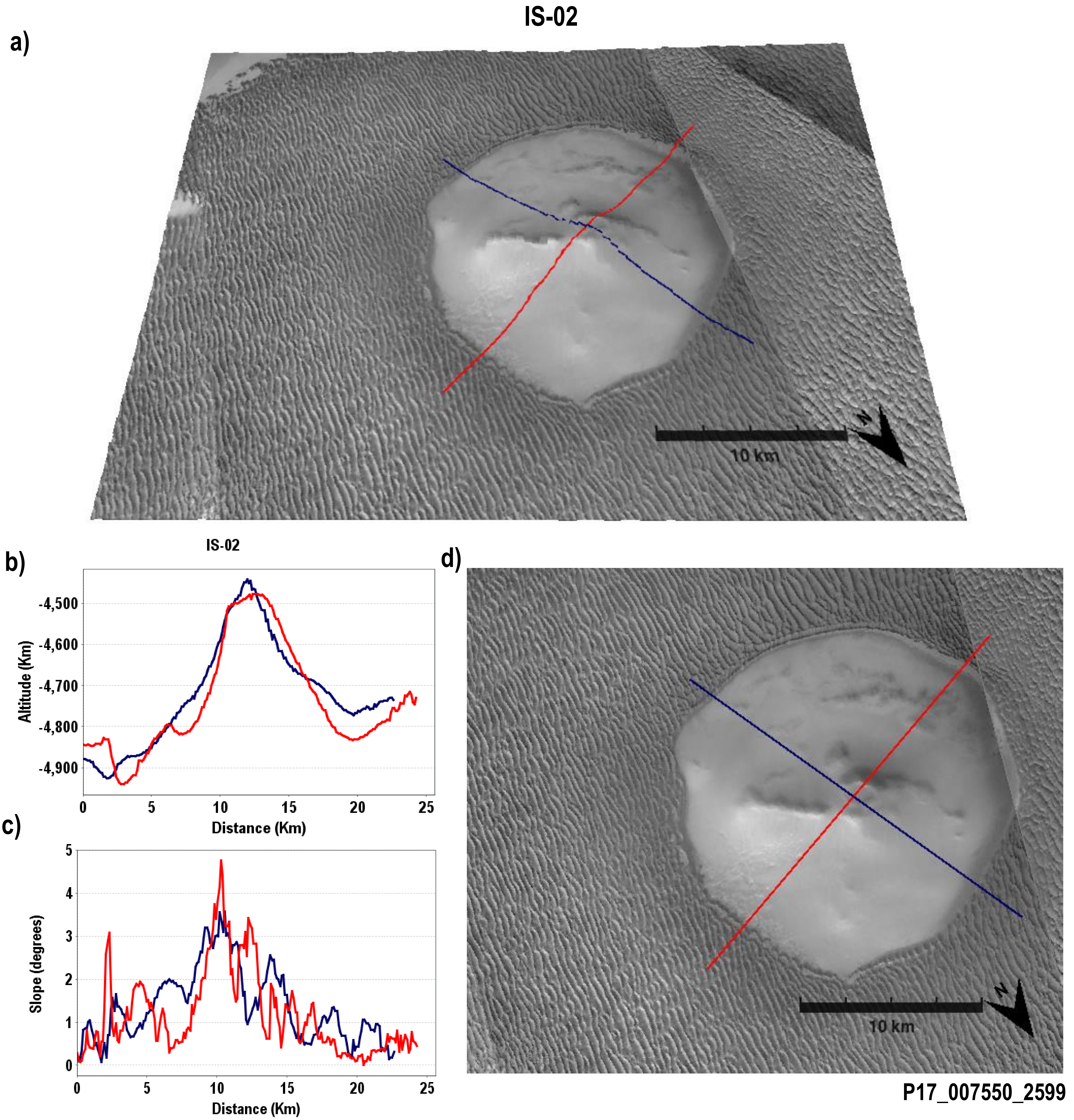
IS-02
a)
10 km
N
b)
IS-02
Altitude (Km)
-4,500
-4,600
-4,700
-4,800
-4,900
0
5
10
15
20
25
Distance (Km)
c)
Slope (degrees)
5
4
3
2
1
0
0
5
10
15
20
25
Distance (Km)
d)
10 km
N
P17_007550_2599

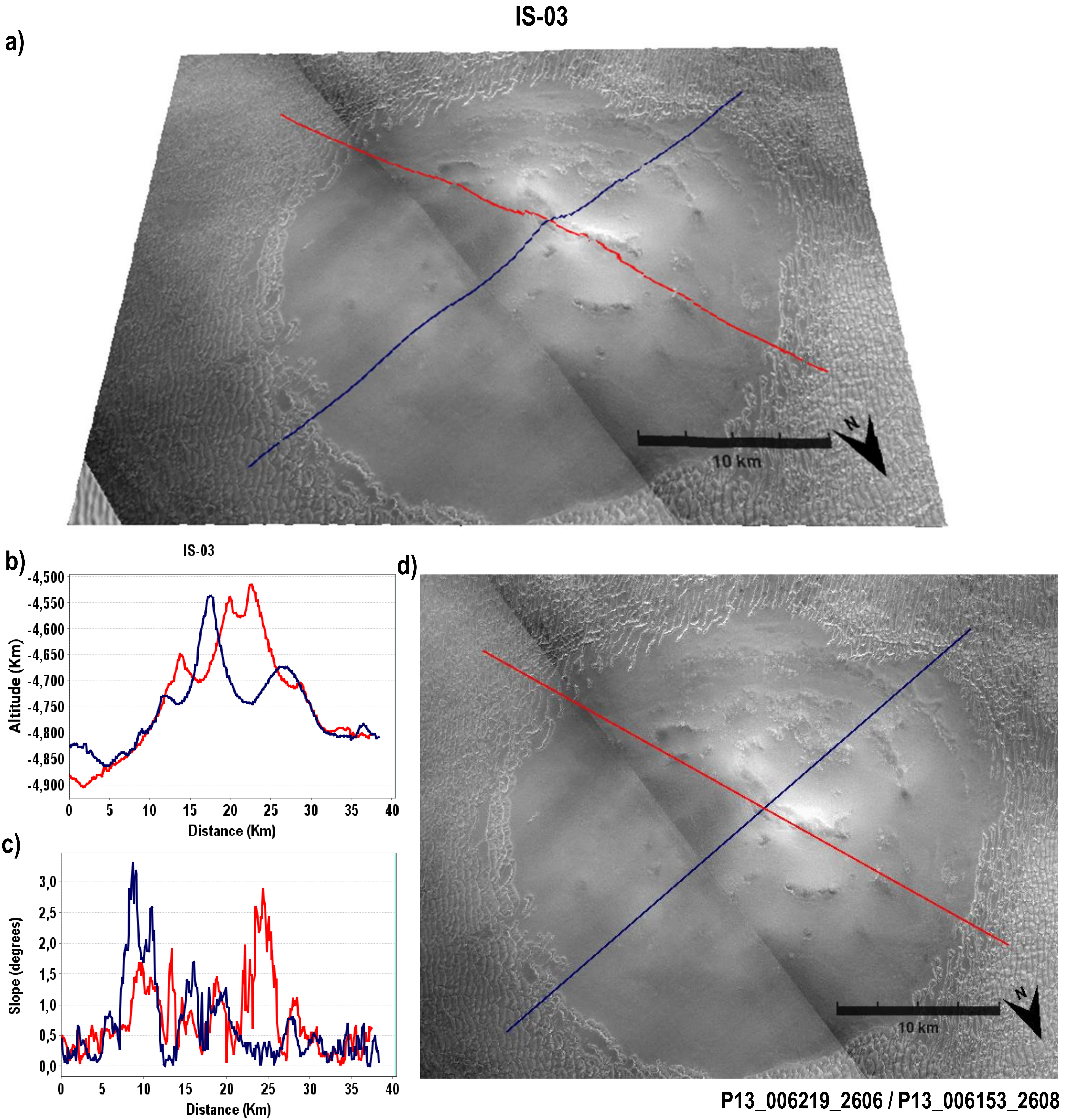
IS-03
a)
10 km
N
b)
IS-03
Altitude (Km)
-4,500
-4,550
-4,600
-4,650
-4,700
-4,750
-4,800
-4,850
-4,900
Distance (Km)
0
5
10
15
20
25
30
35
40
c)
Slope (degrees)
3,0
2,5
2,0
1,5
1,0
0,5
0,0
Distance (Km)
0
5
10
15
20
25
30
35
40
d)
10 km
N
P13_006219_2606 / P13_006153_2608

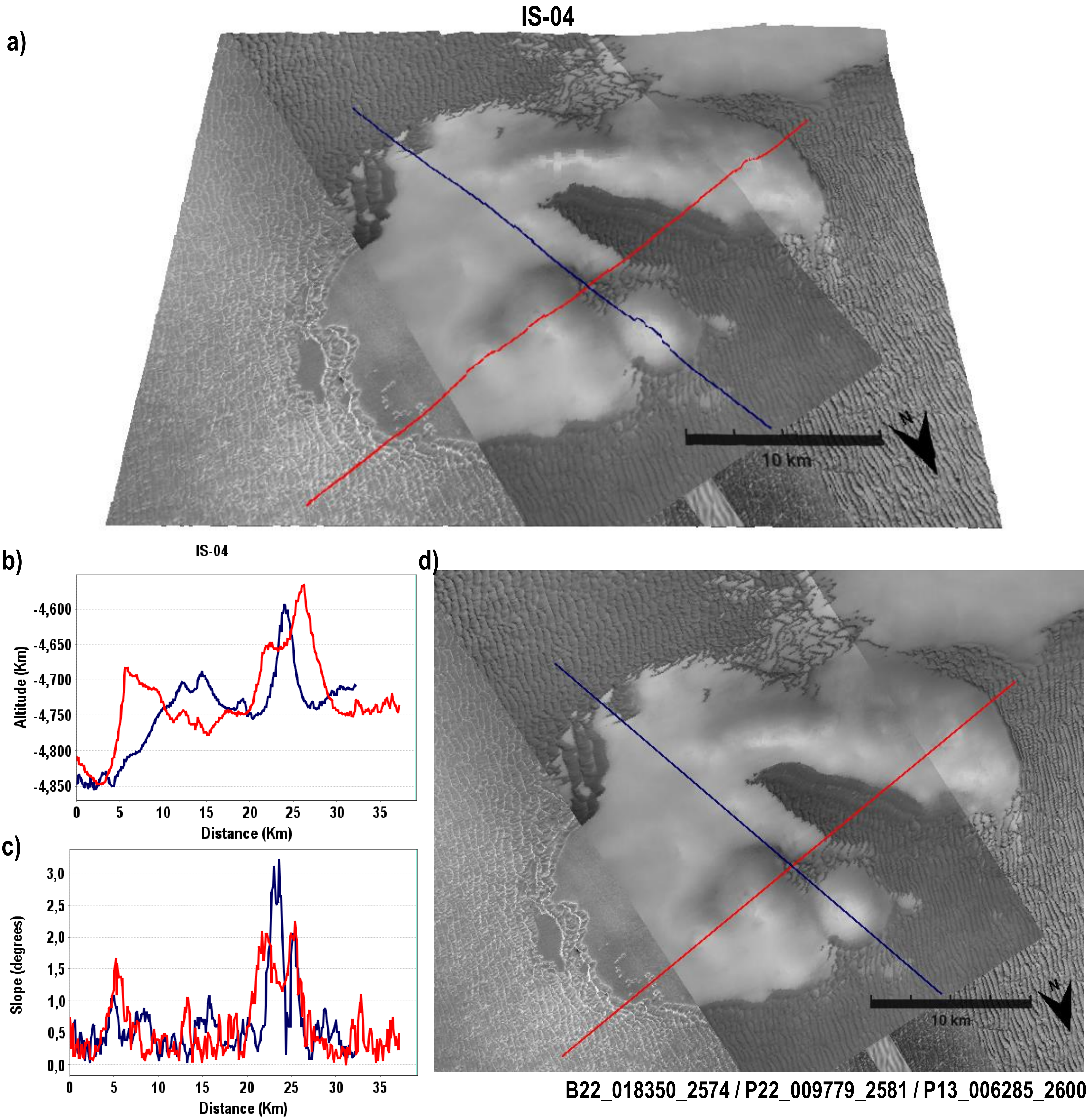

IS-04
a)
10 km
N
b)
IS-04
Altitude (Km)
-4,600
-4,650
-4,700
-4,750
-4,800
-4,850
0
5
10
15
20
25
30
35
Distance (Km)
c)
Slope (degrees)
3,0
2,5
2,0
1,5
1,0
0,5
0,0
Distance (Km)
d)
10 km
B22_018350_2574 / P22_009779_2581 / P13_006285_2600

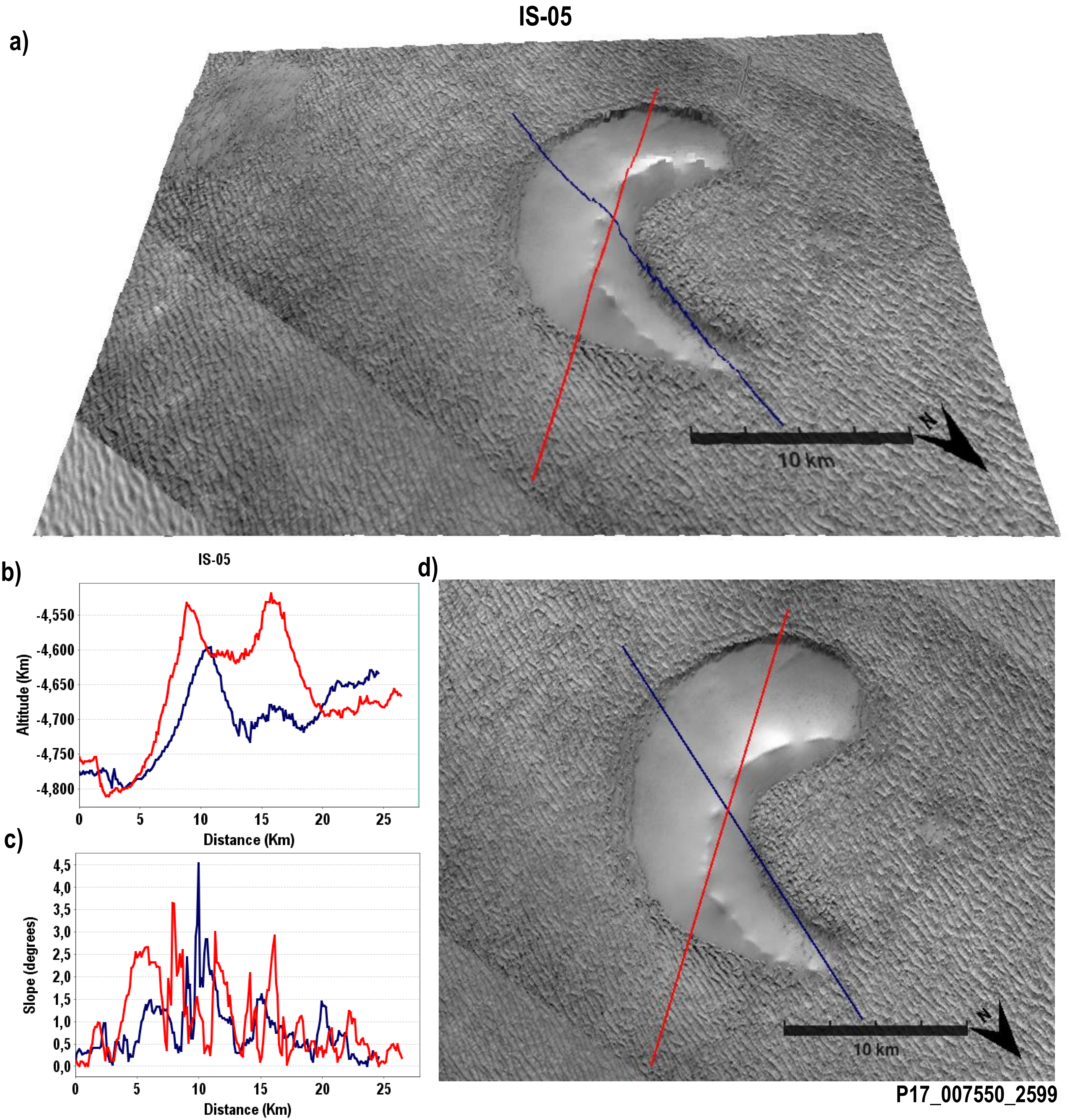
IS-05
a)
10 km
N
b)
IS-05
Altitude (Km)
-4,550
-4,600
-4,650
-4,700
-4,750
-4,800
0
5
10
15
20
25
Distance (Km)
c)
Slope (degrees)
4,5
4,0
3,5
3,0
2,5
2,0
1,5
1,0
0,5
0,0
Distance (Km)
d)
10 km
N
P17_007550_2599

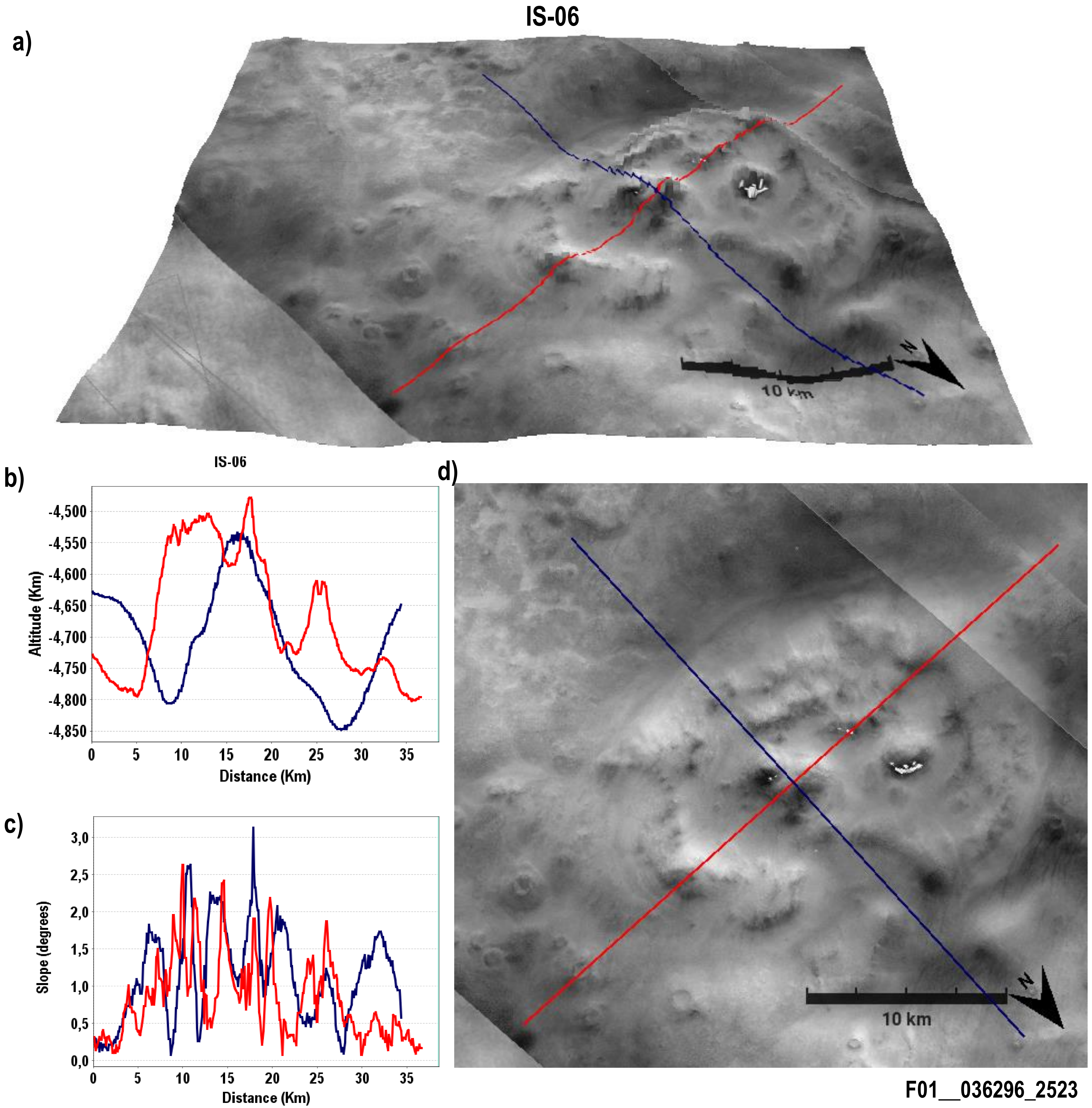
IS-06
a)
10 km
N
IS-06
b)
Altitude (Km)
-4,500
-4,550
-4,600
-4,650
-4,700
-4,750
-4,800
-4,850
0
5
10
15
20
25
30
35
Distance (Km)
c)
Slope (degrees)
3,0
2,5
2,0
1,5
1,0
0,5
0,0
Distance (Km)
d)
10 km
N
F01__036296_2523

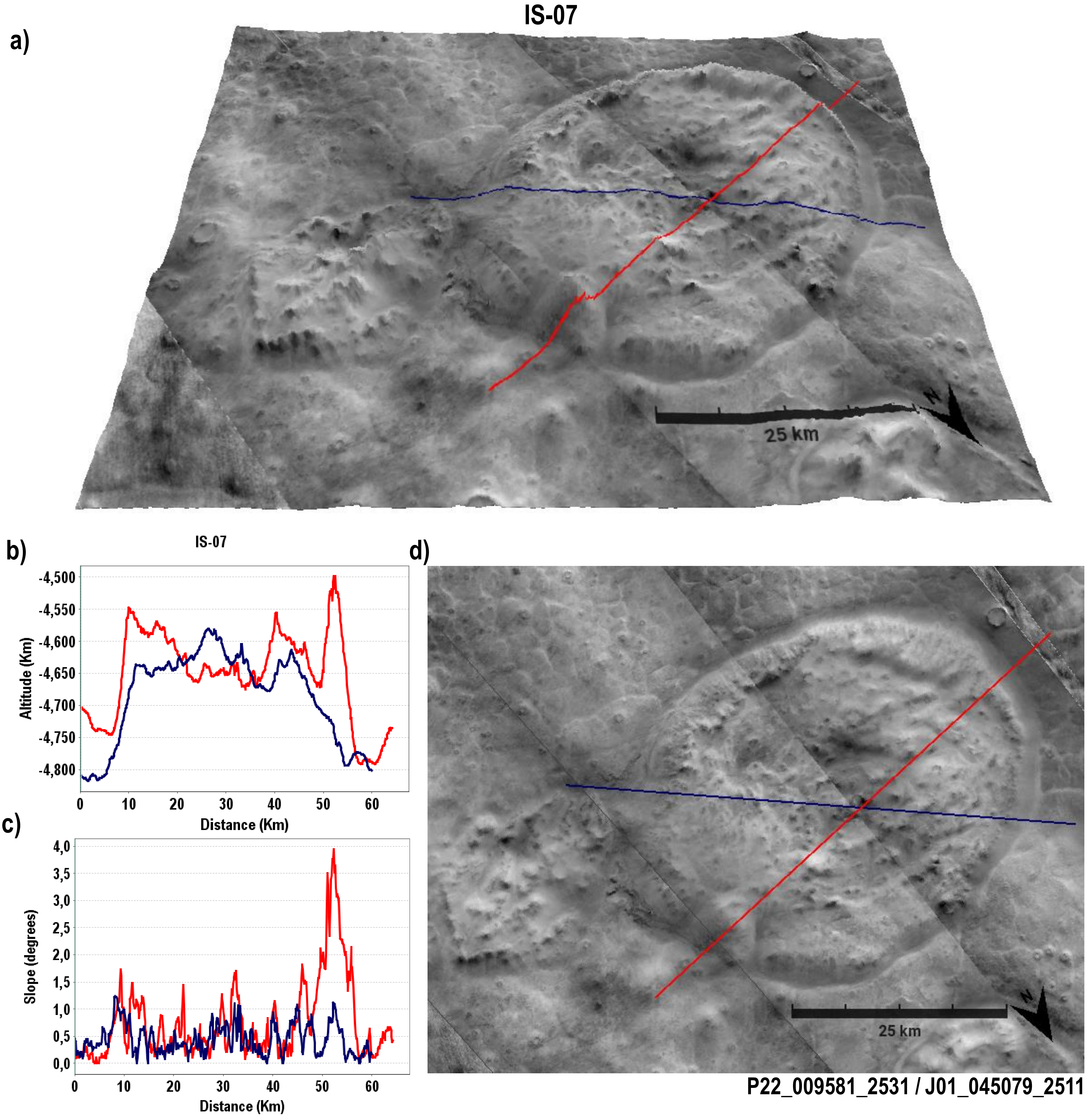
IS-07
a)
25 km
N
b)
IS-07
Altitude (Km)
-4,500
-4,550
-4,600
-4,650
-4,700
-4,750
-4,800
0
10
20
30
40
50
60
Distance (Km)
c)
Slope (degrees)
4,0
3,5
3,0
2,5
2,0
1,5
1,0
0,5
0,0
0
10
20
30
40
50
60
Distance (Km)
d)
25 km
N
P22_009581_2531 / J01_045079_2511

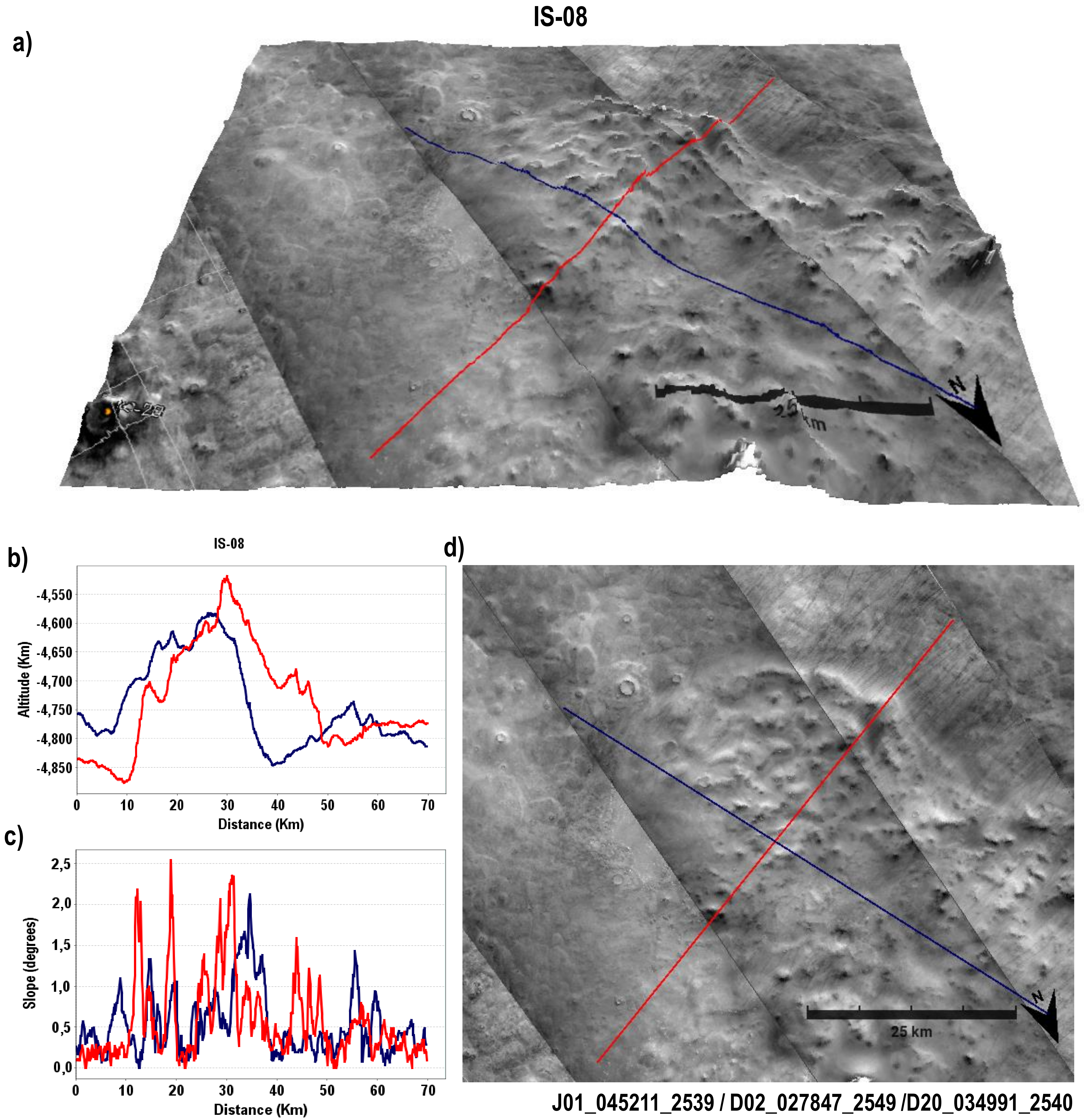

J01_045211_2539 / D02_027847_2549 /D20_034991_2540

IS-09

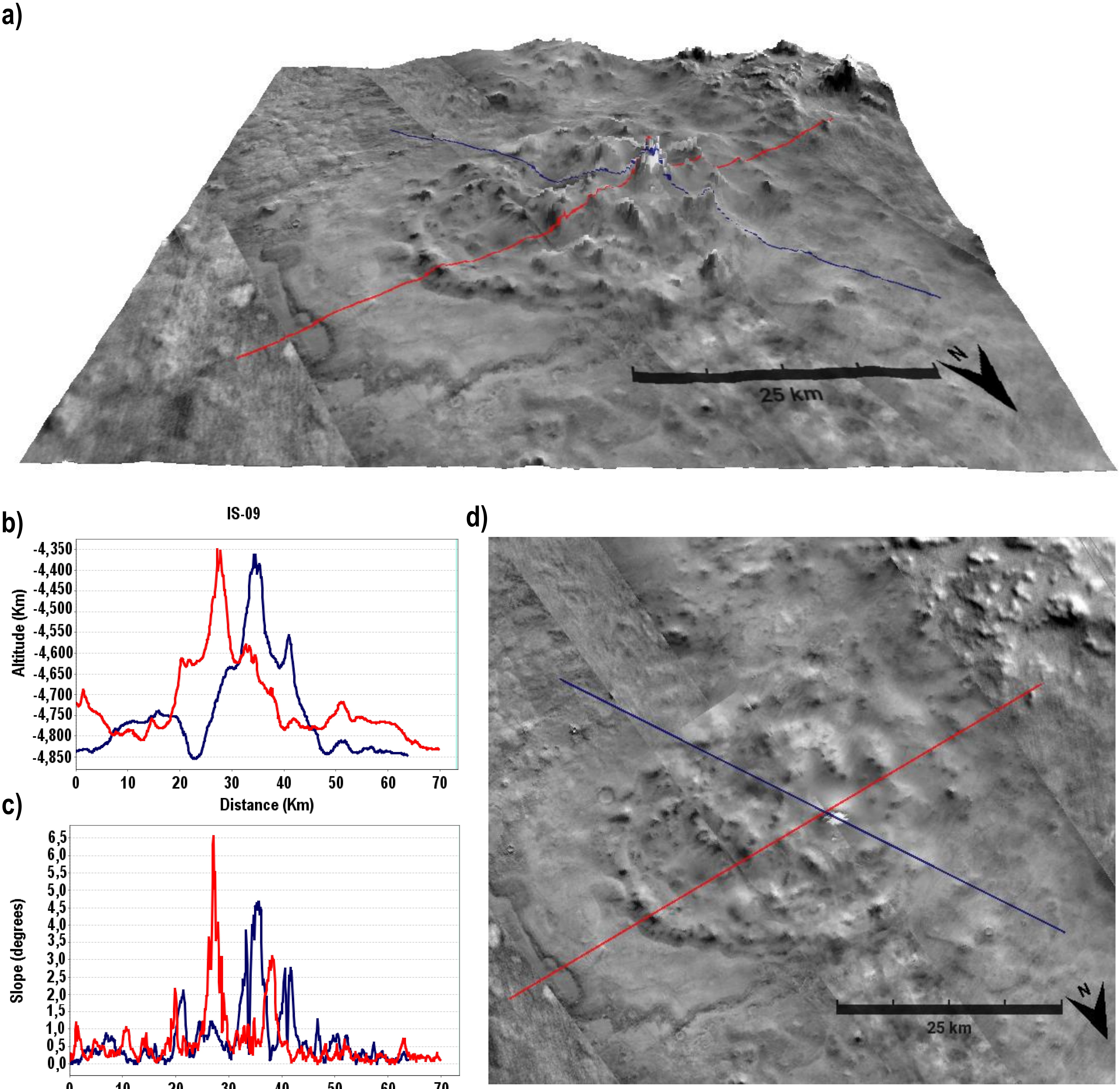

J01_045211_2539 /J03_046134_2546/ J01_04343_2539

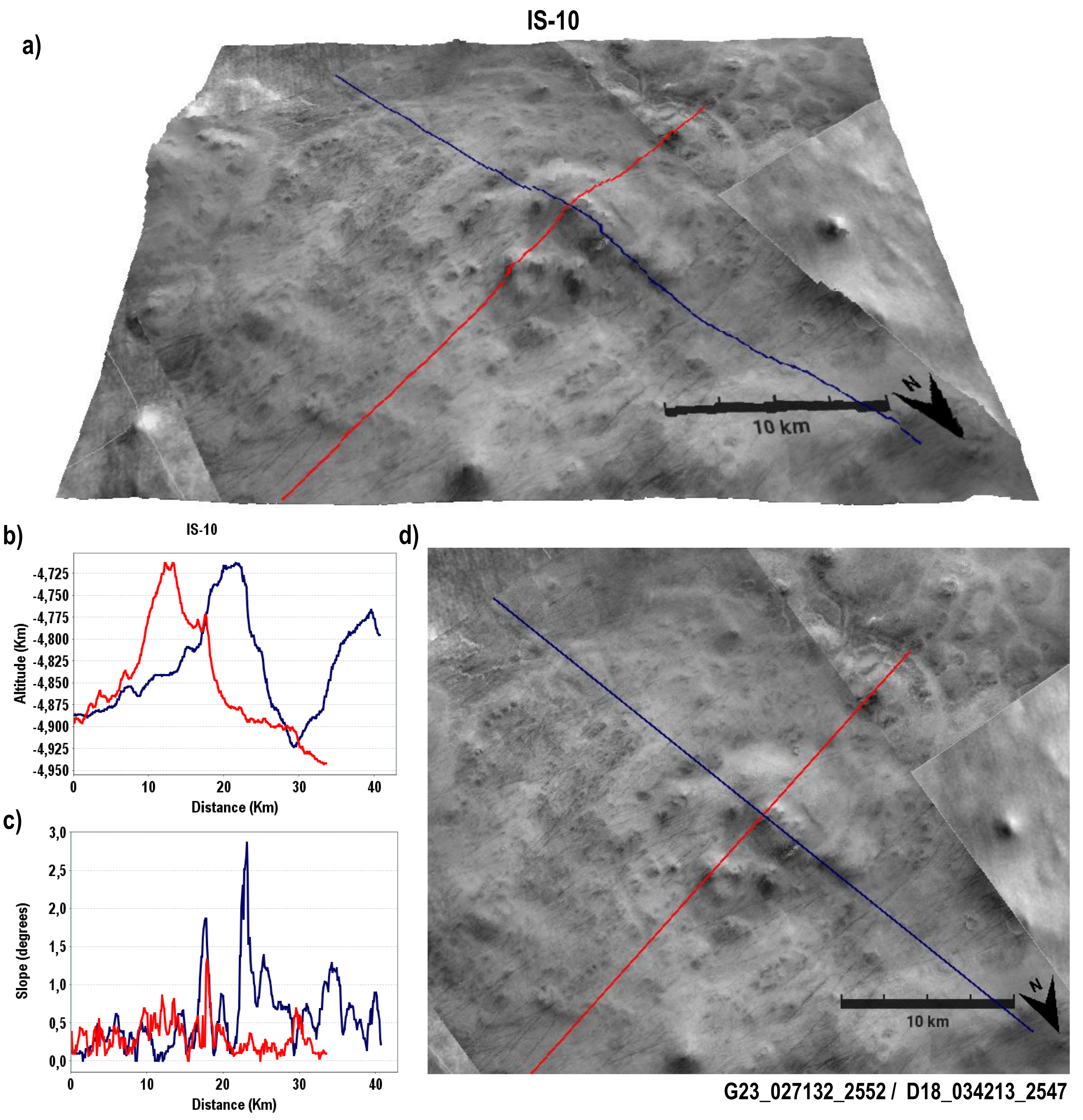
IS-10
a)
10 km
N
b)
IS-10
Altitude (Km)
-4,725
-4,750
-4,775
-4,800
-4,825
-4,850
-4,875
-4,900
-4,925
-4,950
0
10
20
30
40
Distance (Km)
c)
Slope (degrees)
3,0
2,5
2,0
1,5
1,0
0,5
0,0
Distance (Km)
d)
10 km
G23_027132_2552 / D18_034213_2547

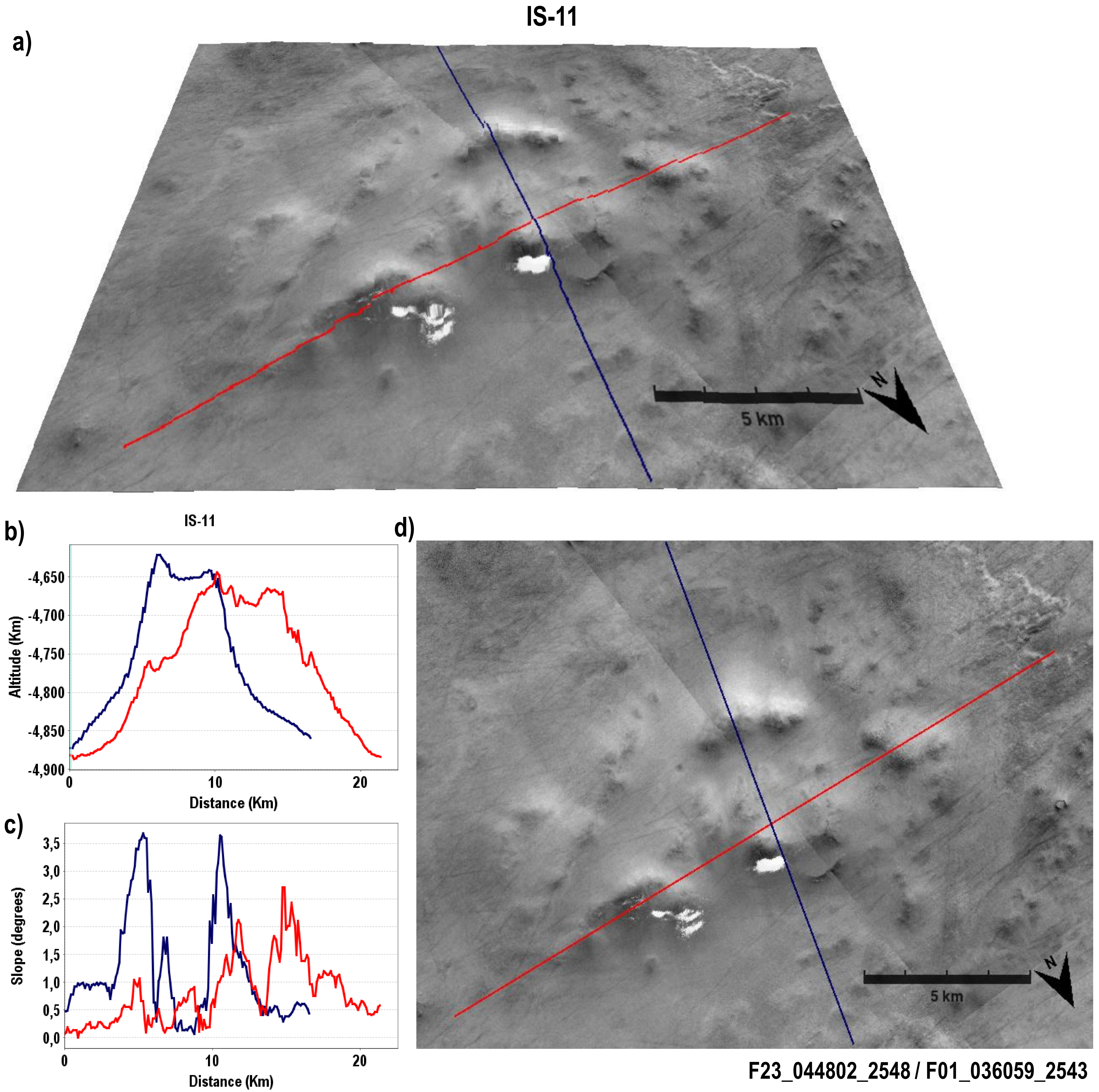

IS-11
a)
5 km
N
b)
IS-11
Altitude (Km)
-4,650
-4,700
-4,750
-4,800
-4,850
-4,900
0
10
20
Distance (Km)
c)
Slope (degrees)
3,5
3,0
2,5
2,0
1,5
1,0
0,5
0,0
0
10
20
Distance (Km)
d)
5 km
N
F23_044802_2548 / F01_036059_2543

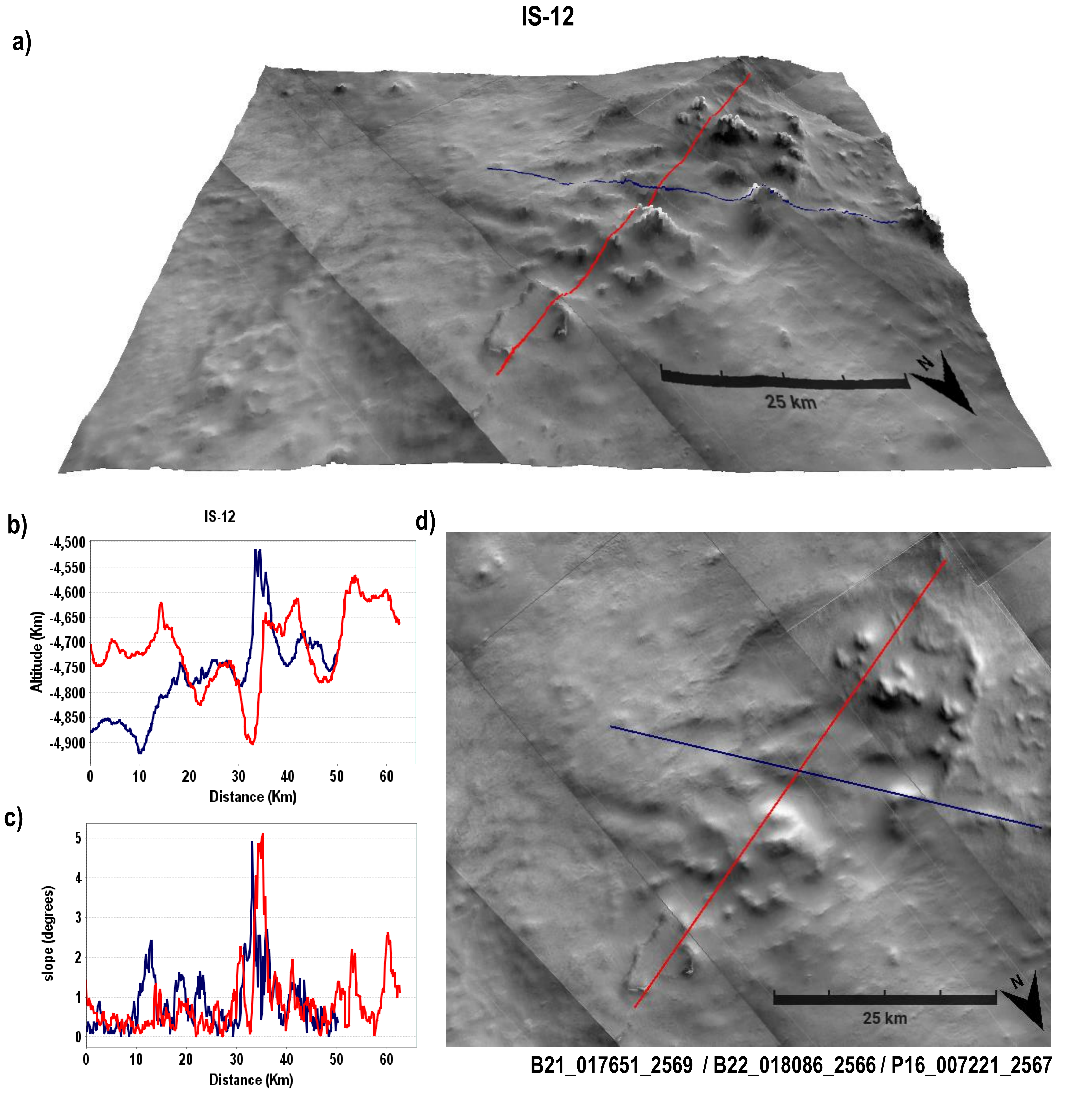
IS-12
a)
25 km
N
b)
IS-12
Altitude (Km)
-4,500
-4,550
-4,600
-4,650
-4,700
-4,750
-4,800
-4,850
-4,900
0
10
20
30
40
50
60
Distance (Km)
c)
slope (degrees)
5
4
3
2
1
0
0
10
20
30
40
50
60
Distance (Km)
d)
25 km
N
B21_017651_2569 / B22_018086_2566 / P16_007221_2567

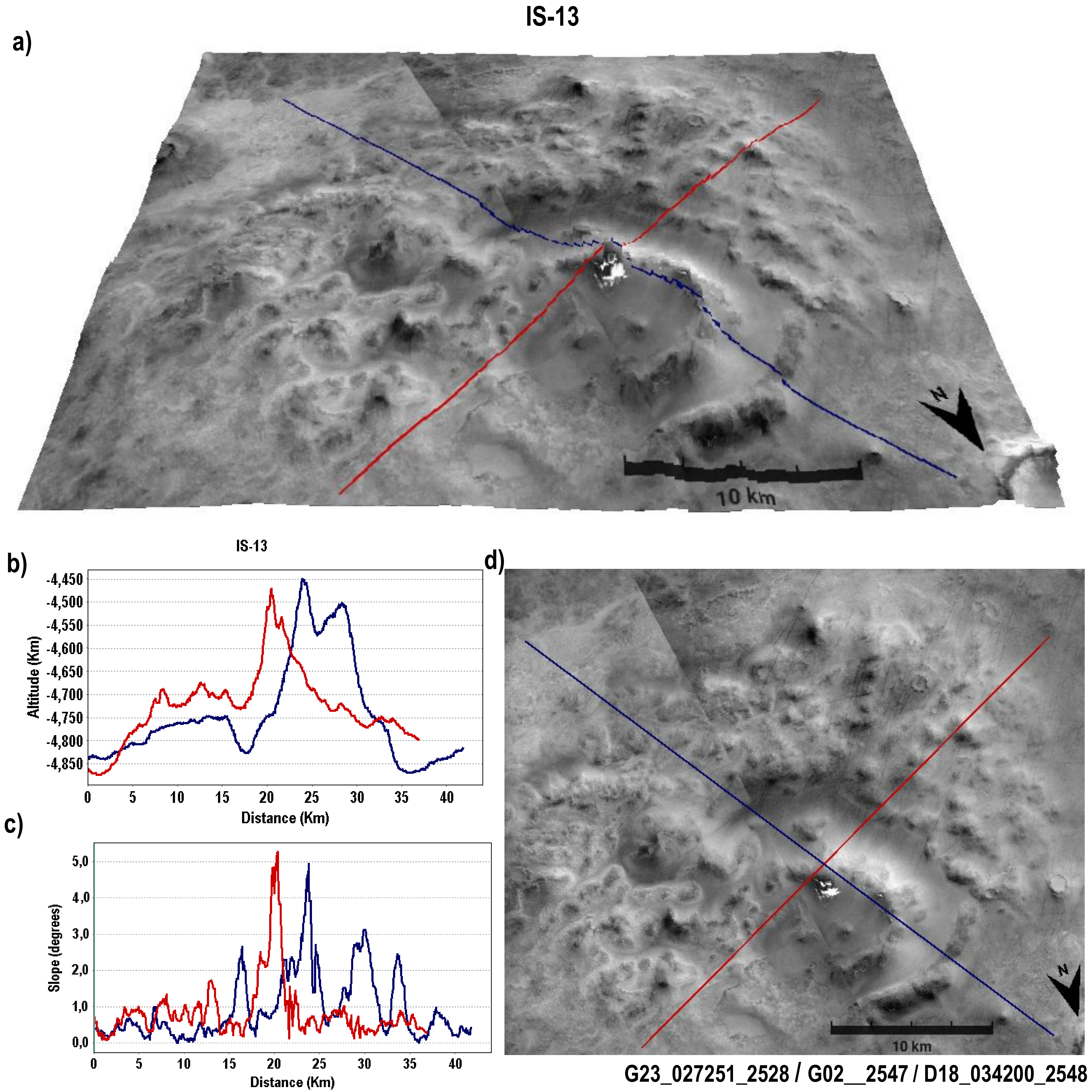

G23_027251_2528 / G02__2547 / D18_034200_2548

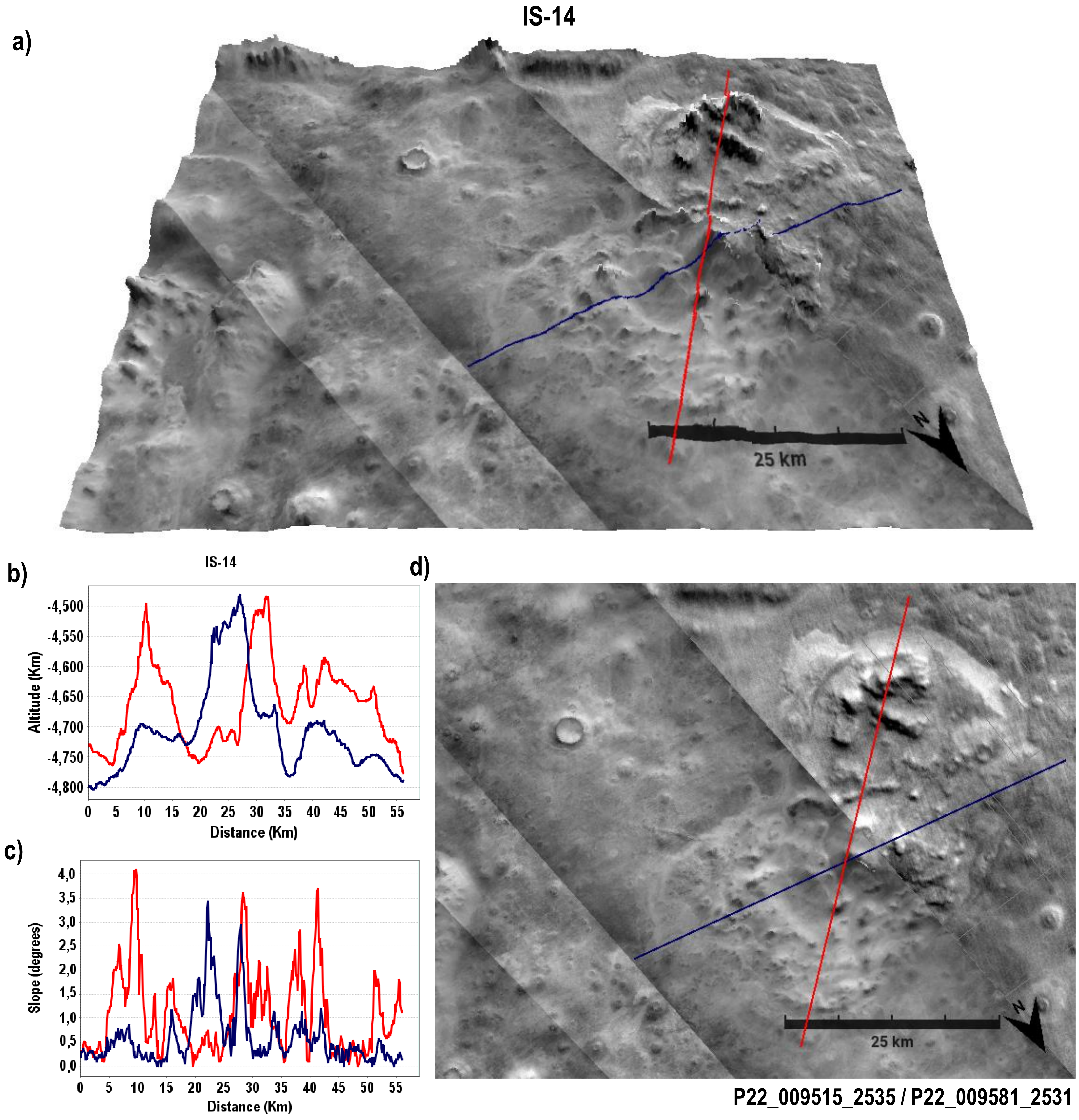

IS-14
a)
25 km
N
b)
IS-14
Altitude (Km)
-4,500
-4,550
-4,600
-4,650
-4,700
-4,750
-4,800
0 5 10 15 20 25 30 35 40 45 50 55
Distance (Km)
c)
Slope (degrees)
4,0
3,5
3,0
2,5
2,0
1,5
1,0
0,5
0,0
0 5 10 15 20 25 30 35 40 45 50 55
Distance (Km)
d)
25 km
N
P22_009515_2535 / P22_009581_2531

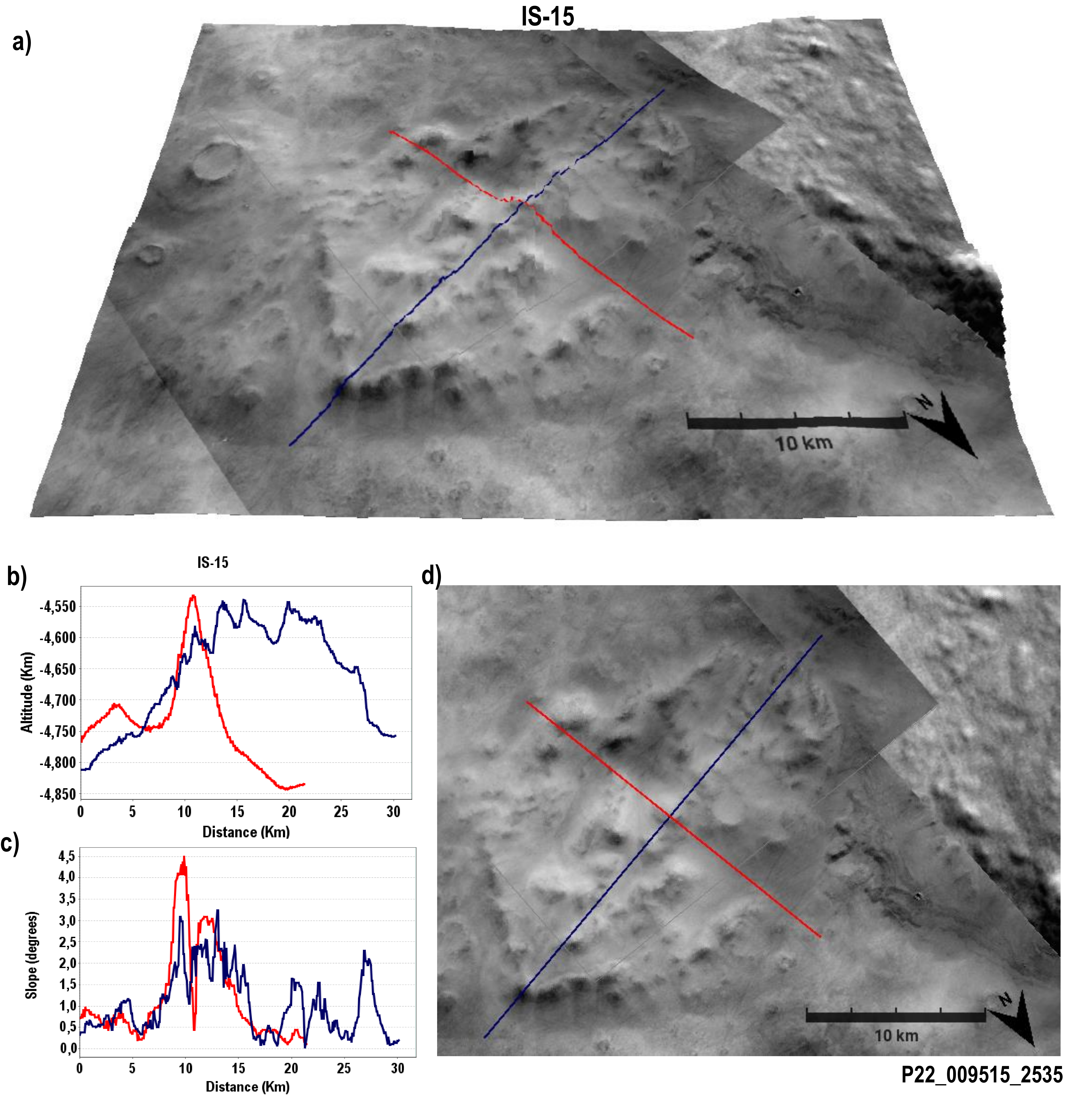

IS-15
a)
10 km
N
b)
IS-15
Altitude (Km)
-4,550
-4,600
-4,650
-4,700
-4,750
-4,800
-4,850
Distance (Km)
0
5
10
15
20
25
30
c)
Slope (degrees)
4,5
4,0
3,5
3,0
2,5
2,0
1,5
1,0
0,5
0,0
Distance (Km)
0
5
10
15
20
25
30
d)
10 km
N
P22_009515_2535

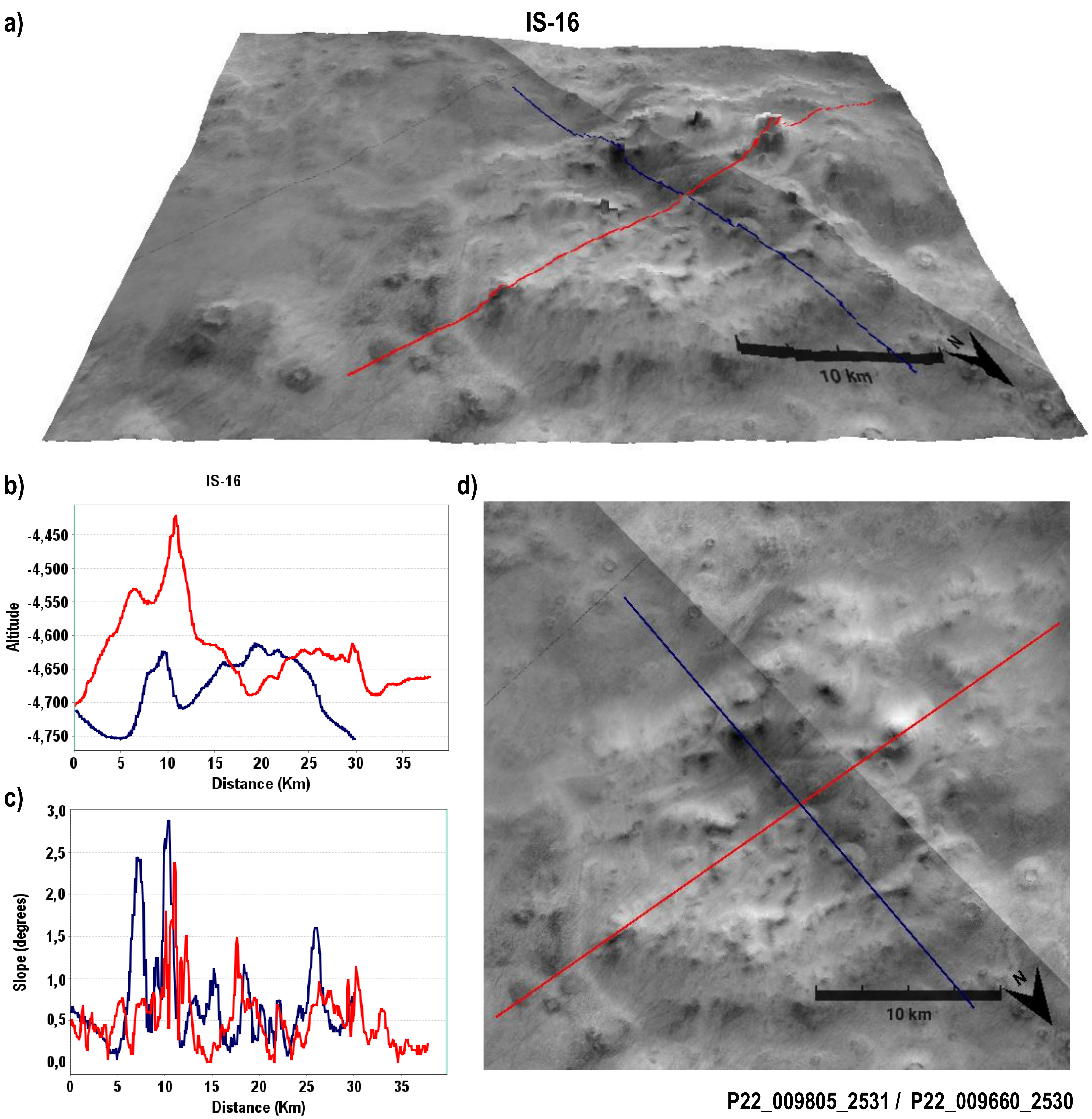
a)
IS-16
10 km
N
b)
IS-16
Altitude
-4,450
-4,500
-4,550
-4,600
-4,650
-4,700
-4,750
0 5 10 15 20 25 30 35
Distance (Km)
c)
Slope (degrees)
3,0
2,5
2,0
1,5
1,0
0,5
0,0
0 5 10 15 20 25 30 35
Distance (Km)
d)
10 km
N
P22_009805_2531 / P22_009660_2530

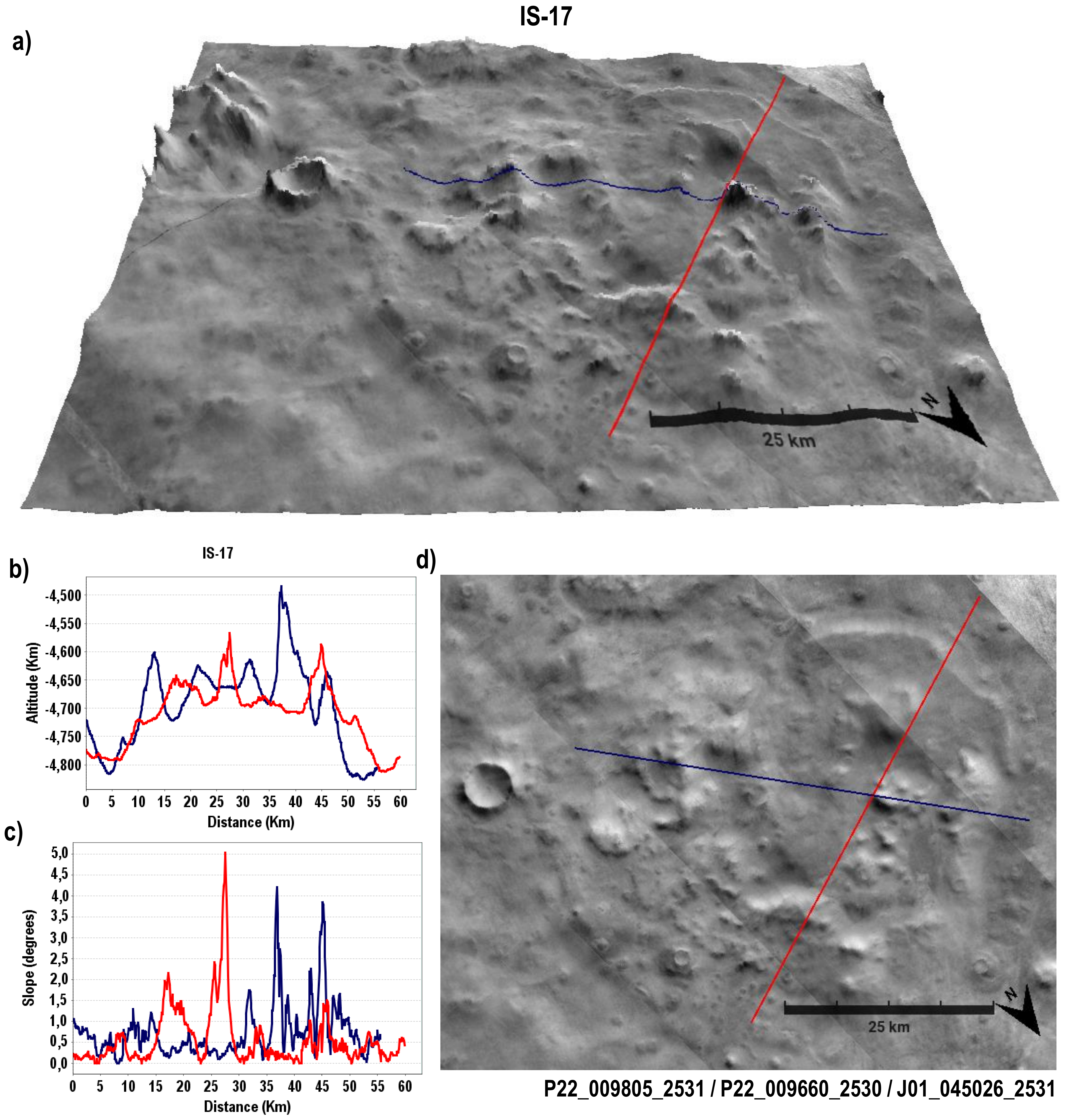
IS-17
a)
25 km
N
b)
IS-17
Altitude (Km)
-4,500
-4,550
-4,600
-4,650
-4,700
-4,750
-4,800
0 5 10 15 20 25 30 35 40 45 50 55 60
Distance (Km)
c)
Slope (degrees)
5,0
4,5
4,0
3,5
3,0
2,5
2,0
1,5
1,0
0,5
0,0
0 5 10 15 20 25 30 35 40 45 50 55 60
Distance (Km)
d)
25 km
N
P22_009805_2531 / P22_009660_2530 / J01_045026_2531

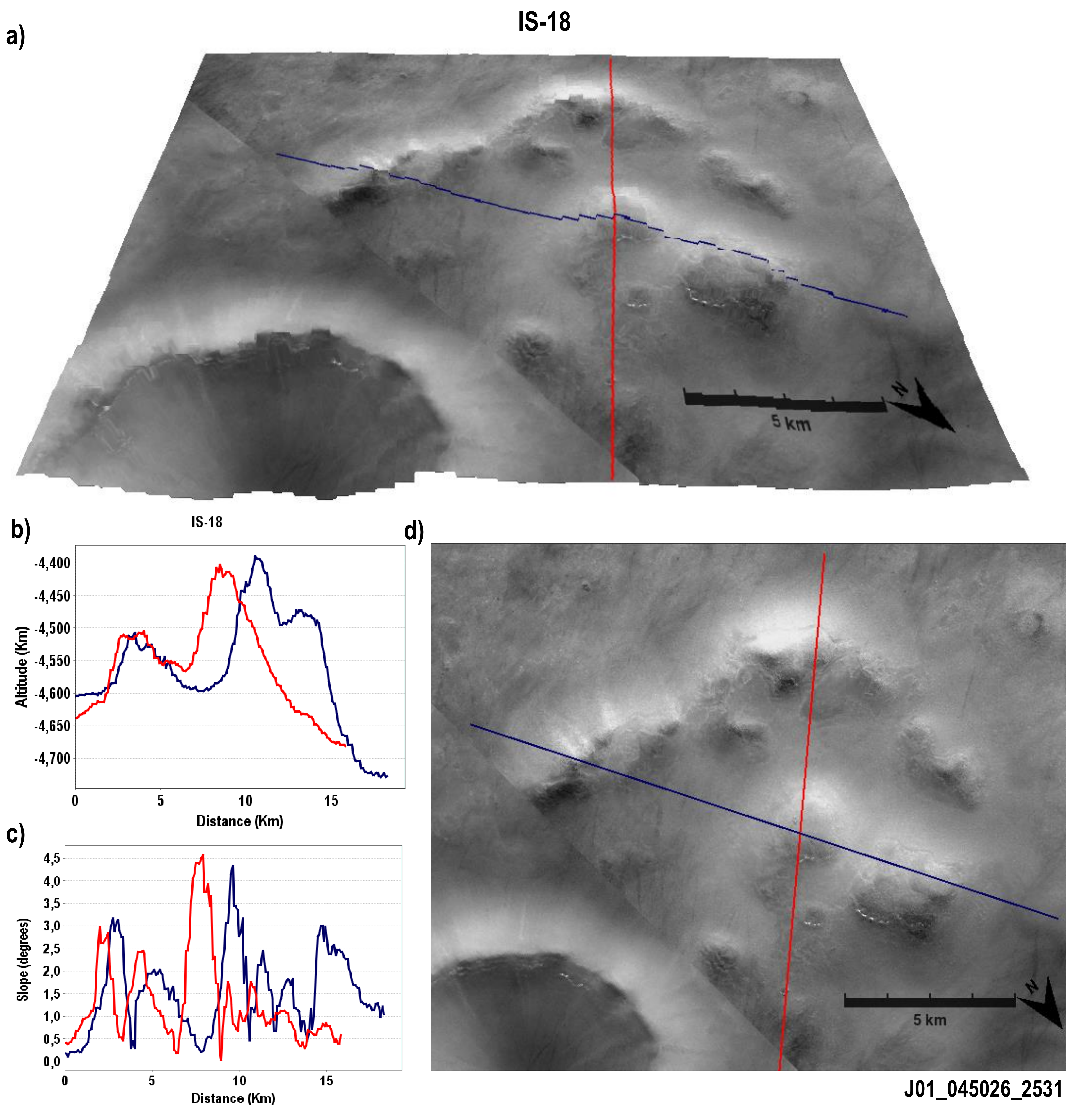
a)
IS-18
5 km
N
b)
IS-18
Altitude (Km)
-4,400
-4,450
-4,500
-4,550
-4,600
-4,650
-4,700
0
5
10
15
Distance (Km)
c)
Slope (degrees)
4,5
4,0
3,5
3,0
2,5
2,0
1,5
1,0
0,5
0,0
0
5
10
15
Distance (Km)
d)
5 km
N
J01_045026_2531

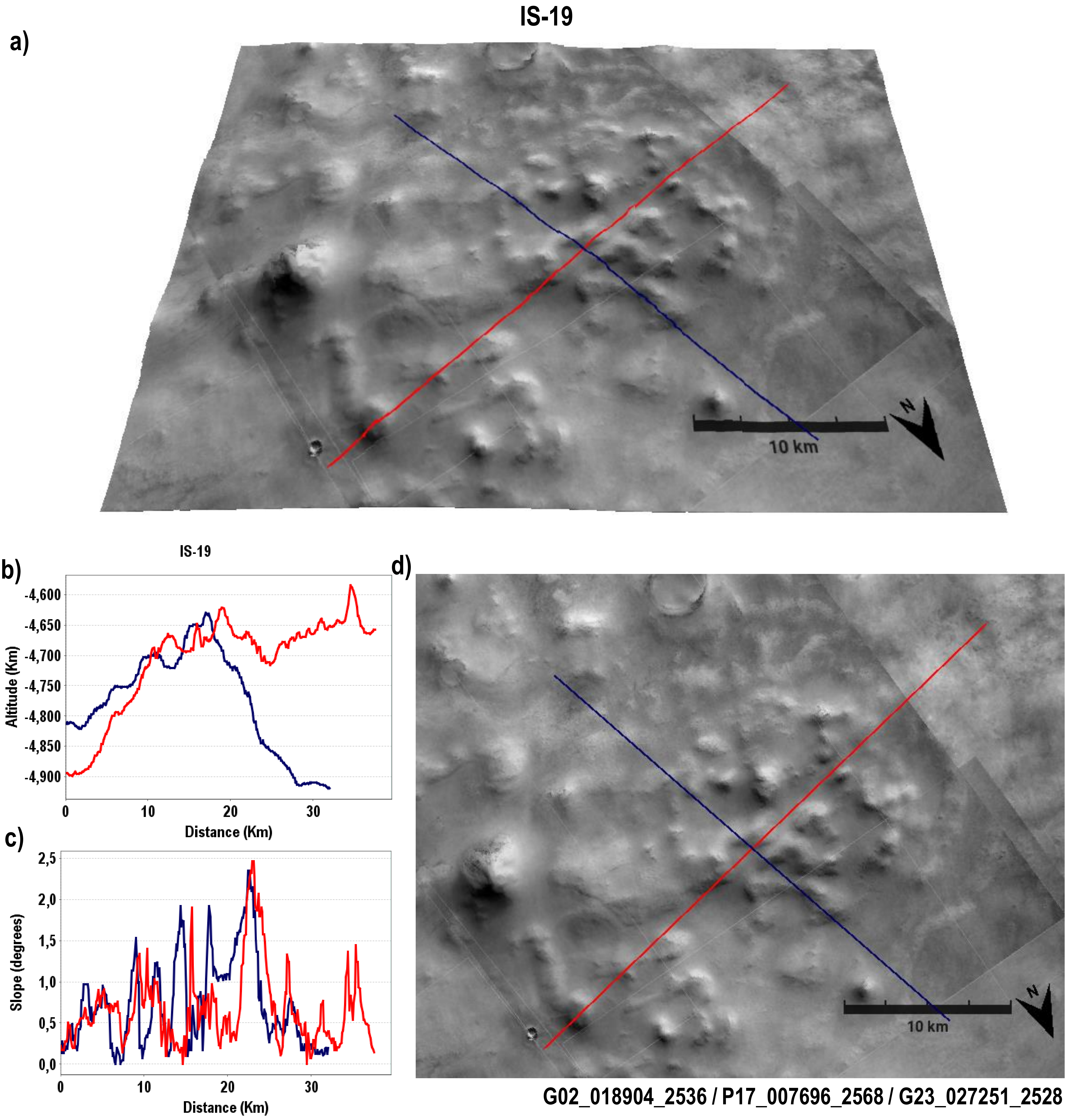
IS-19
a)
10 km
N
b)
IS-19
Altitude (Km)
-4,600
-4,650
-4,700
-4,750
-4,800
-4,850
-4,900
0
10
20
30
Distance (Km)
c)
Slope (degrees)
2,5
2,0
1,5
1,0
0,5
0,0
0
10
20
30
Distance (Km)
d)
10 km
N
G02_018904_2536 / P17_007696_2568 / G23_027251_2528

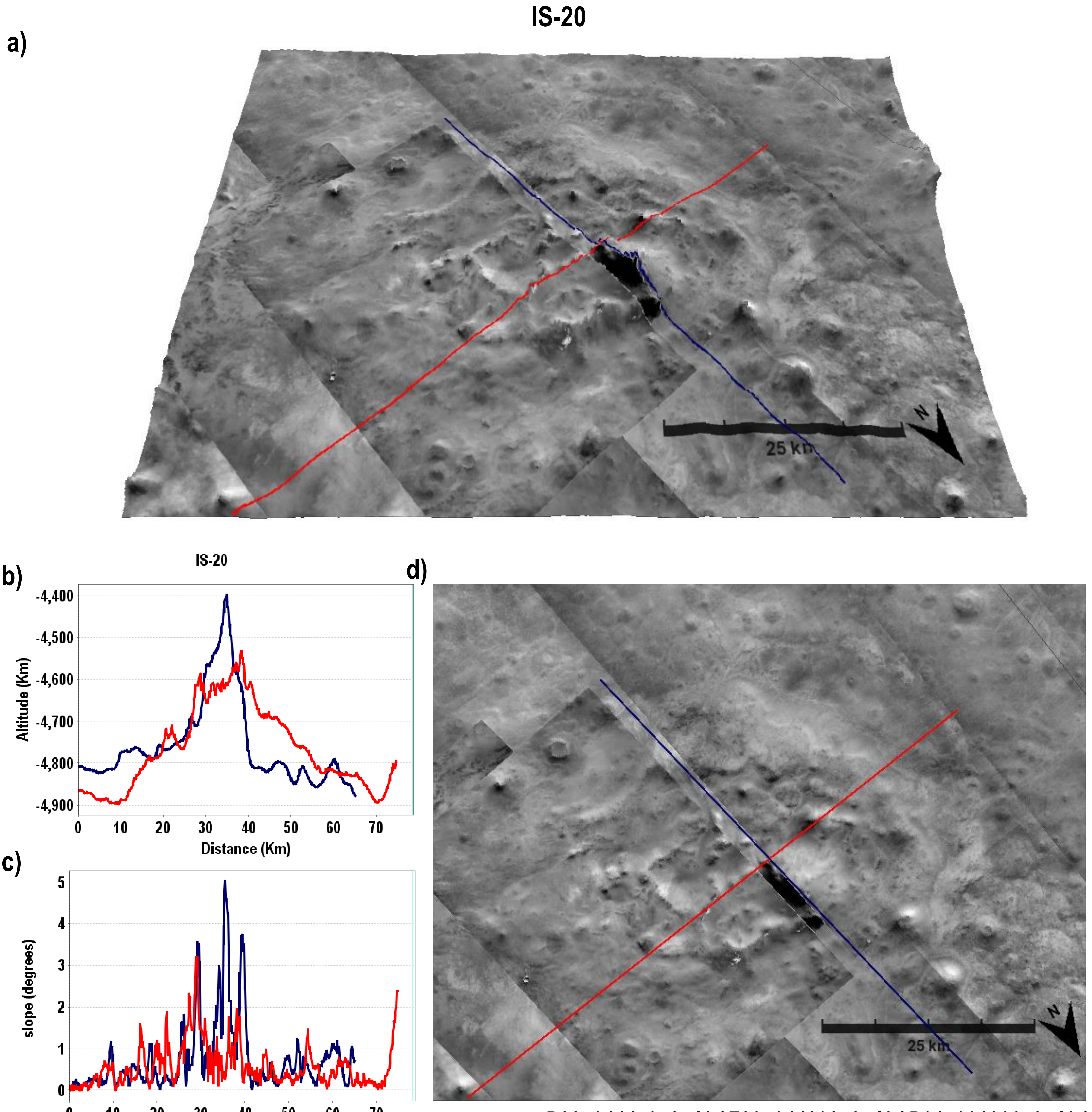

P22_044459_2546 / F23_044802_2548 / P01_001366_2546 / P22_009515_2535

IS-21

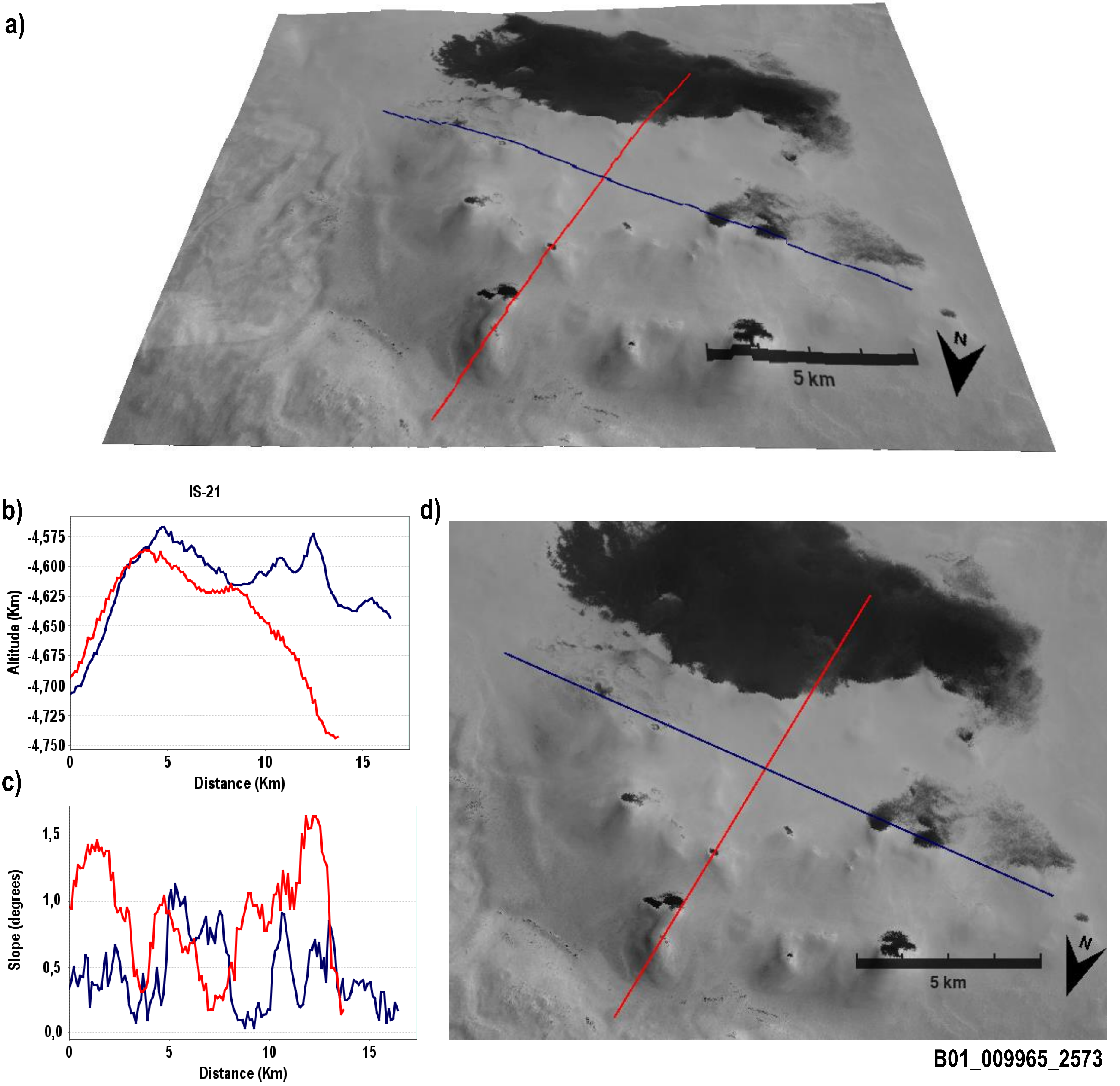

a)
5 km
N
b)
IS-21
-4,575
-4,600
-4,625
-4,650
-4,675
-4,700
-4,725
-4,750
Altitude (Km)
0
5
10
15
Distance (Km)
c)
1,5
1,0
0,5
0,0
Slope (degrees)
0
5
10
15
Distance (Km)
d)
5 km
N
B01_009965_2573

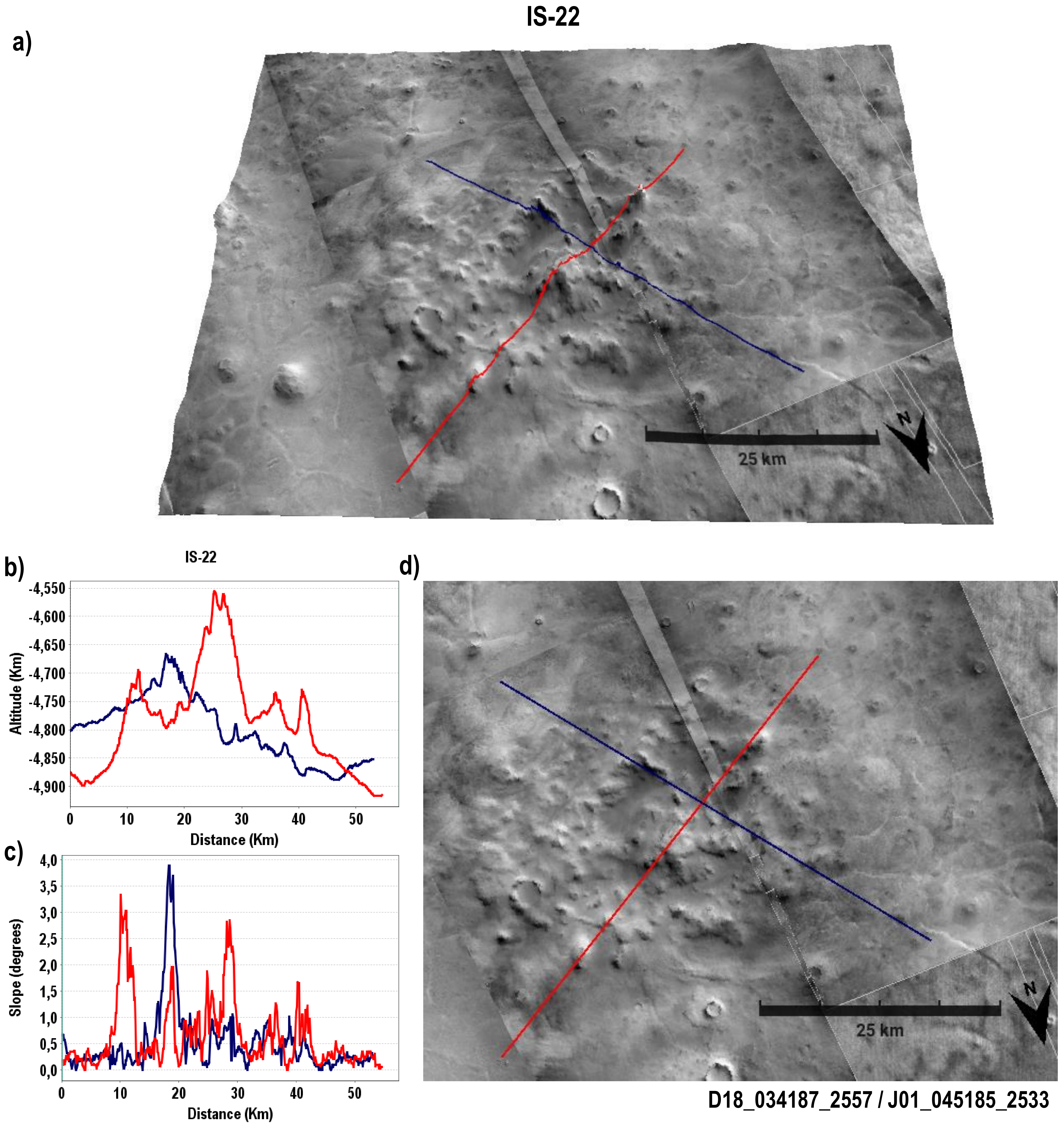
IS-22
a)
25 km
N
b)
IS-22
Altitude (Km)
-4,550
-4,600
-4,650
-4,700
-4,750
-4,800
-4,850
-4,900
0
10
20
30
40
50
Distance (Km)
c)
Slope (degrees)
4,0
3,5
3,0
2,5
2,0
1,5
1,0
0,5
0,0
0
10
20
30
40
50
Distance (Km)
d)
25 km
N
D18_034187_2557 / J01_045185_2533

IS-23

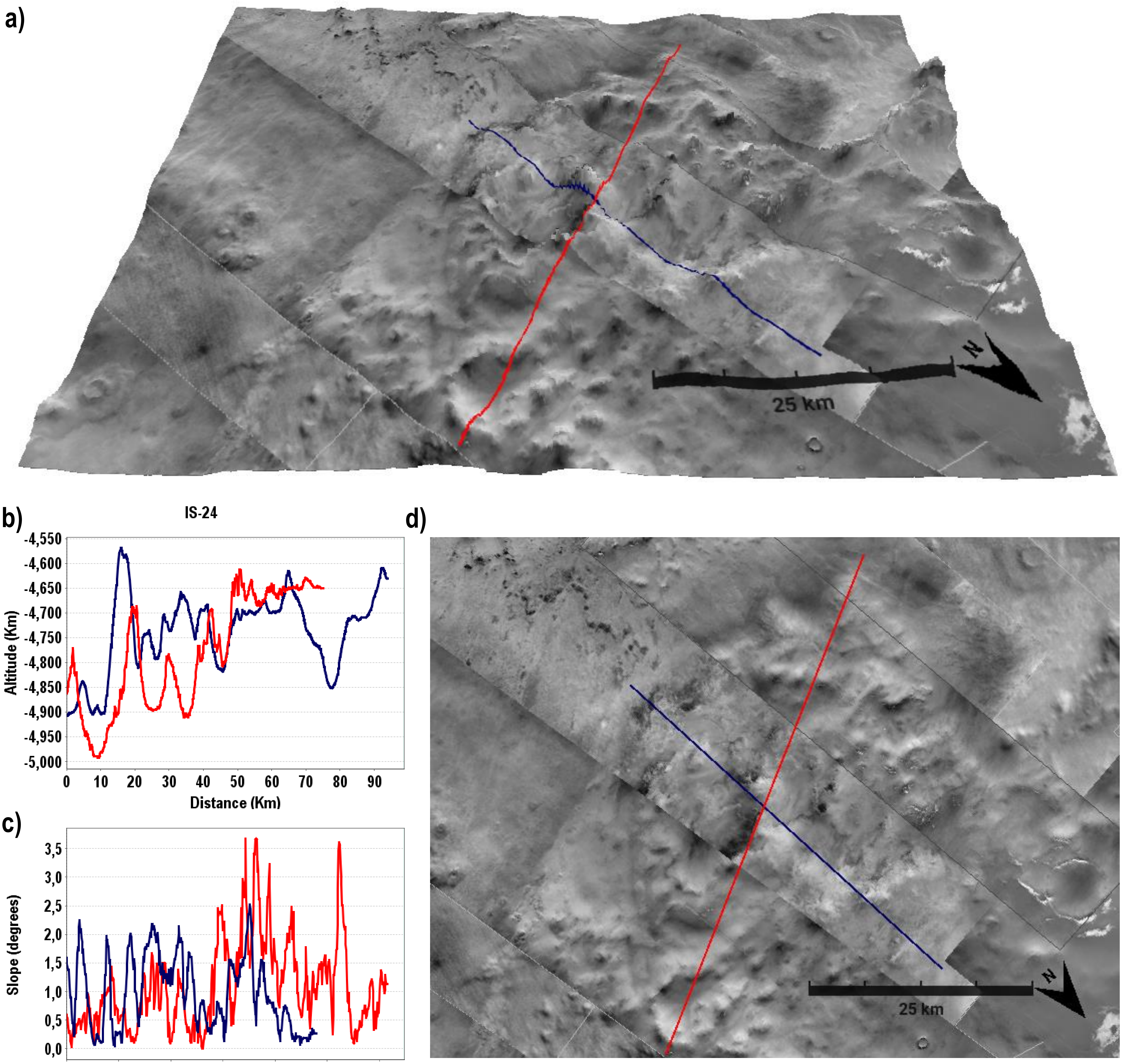

F22_044340_2551 / D22_035610_2546/ K03_054480_2549 / D22_035676_2547

IS-24

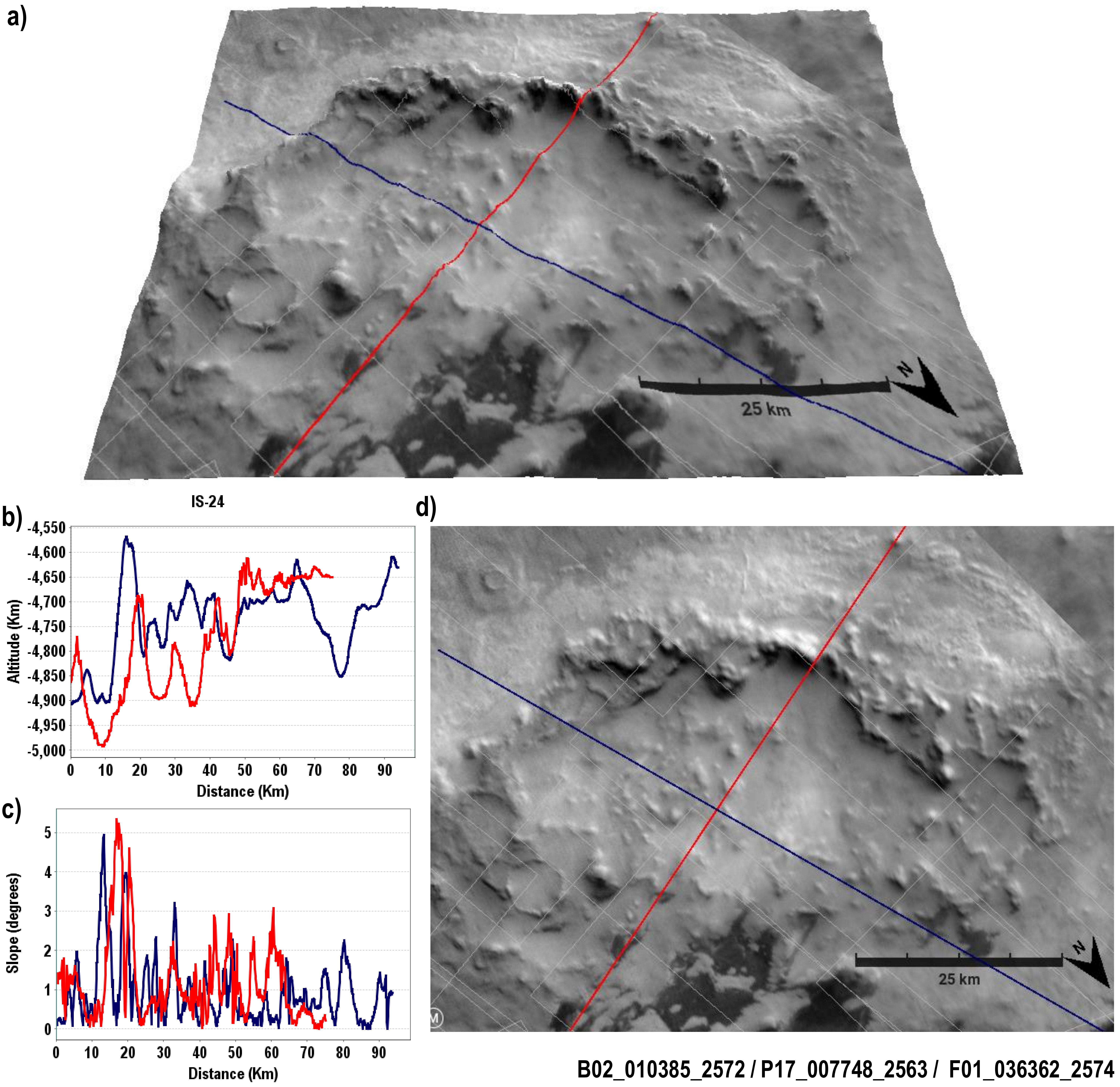

B02_010385_2572 / P17_007748_2563 / F01_036362_2574

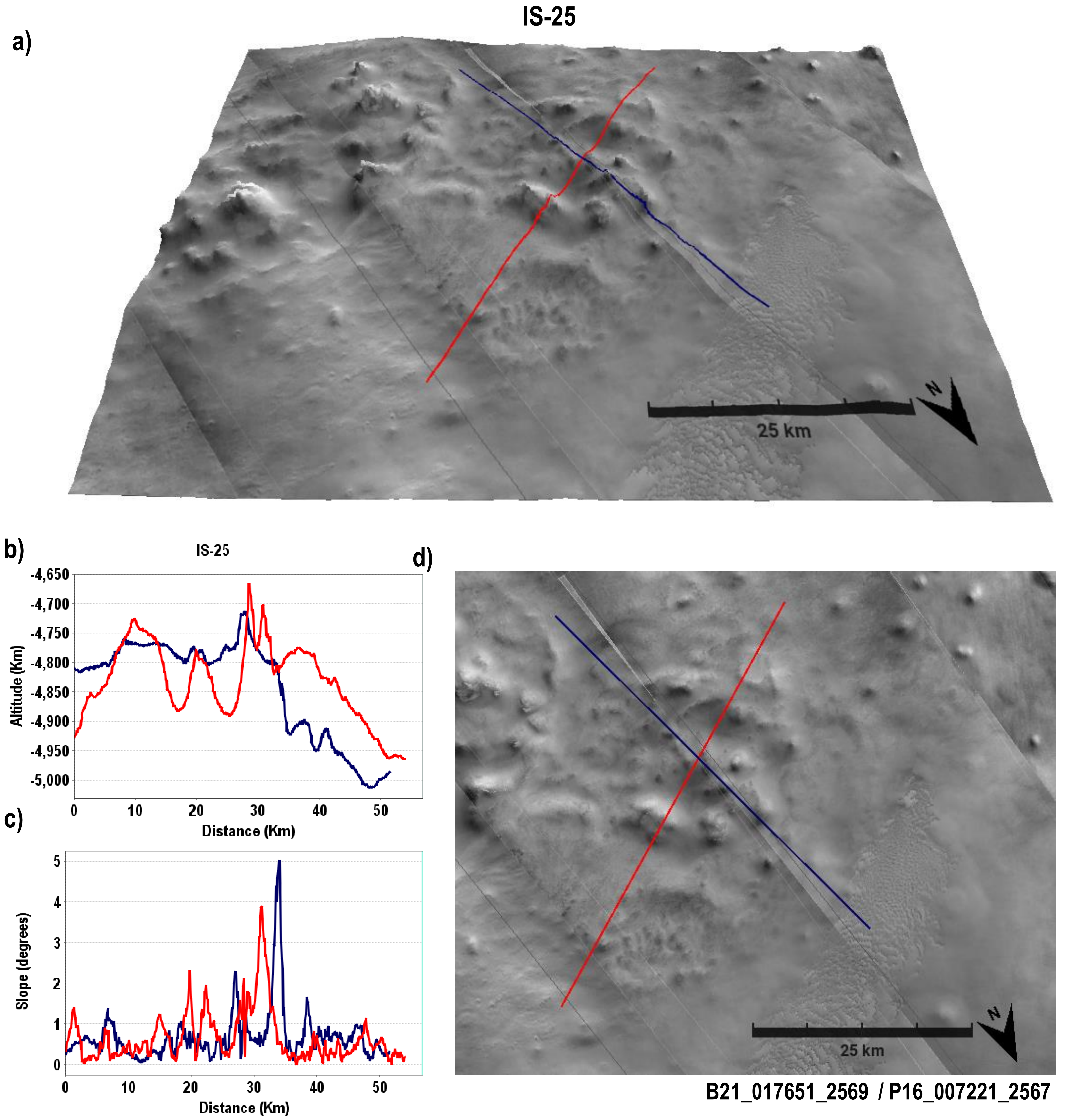

B21_017651_2569 / P16_007221_2567
